# Expanding causal genes for Parkinson’s disease via multi-omics analysis

**DOI:** 10.1038/s41531-023-00591-0

**Published:** 2023-10-21

**Authors:** Xiao-Jing Gu, Wei-Ming Su, Meng Dou, Zheng Jiang, Qing-Qing Duan, Kang-Fu Yin, Bei Cao, Yi Wang, Guo-Bo Li, Yong-Ping Chen

**Affiliations:** 1 Mental Health Center, West China Hospital, Sichuan University, Chengdu, Sichuan China; 2 Department of Neurology, West China Hospital, Sichuan University, Chengdu, Sichuan China; 3 Chengdu Institute of Computer Application, Chinese Academy of Sciences, Chengdu, Sichuan China; 4 Department of Pathophysiology, West China College of Basic Medical Sciences & Forensic Medicine, Sichuan University, Chengdu, China; 5 Key Laboratory of Drug Targeting and Drug Delivery System of Ministry of Education, West China School of Pharmacy, Sichuan University, Chengdu, China

**Keywords:** Medical genetics, Parkinson's disease, Target identification, Translational research

## Abstract

Genome‑wide association studies (GWASs) have revealed numerous loci associated with Parkinson’s disease (PD). However, some potential causal/risk genes were still not revealed and no etiological therapies are available. To find potential causal genes and explore genetically supported drug targets for PD is urgent. By integrating the expression quantitative trait loci (eQTL) and protein quantitative trait loci (pQTL) datasets from multiple tissues (blood, cerebrospinal fluid (CSF) and brain) and PD GWAS summary statistics, a pipeline combing Mendelian randomization (MR), Steiger filtering analysis, Bayesian colocalization, fine mapping, Protein-protein network and enrichment analysis were applied to identify potential causal genes for PD. As a result, GPNMB displayed a robust causal role for PD at the protein level in the blood, CSF and brain, and transcriptional level in the brain, while the protective role of CD38 (in brain pQTL and eQTL) was also identified. We also found inconsistent roles of DGKQ on PD between protein and mRNA levels. Another 9 proteins (CTSB, ARSA, SEC23IP, CD84, ENTPD1, FCGR2B, BAG3, SNCA, FCGR2A) were associated with the risk for PD based on only a single pQTL after multiple corrections. We also identified some proteins’ interactions with known PD causative genes and therapeutic targets. In conclusion, this study suggested GPNMB, CD38, and DGKQ may act in the pathogenesis of PD, but whether the other proteins involved in PD needs more evidence. These findings would help to uncover the genes underlying PD and prioritize targets for future therapeutic interventions.

## Introduction

Parkinson’s disease (PD) is the second most common progressive neurodegenerative disease, and a large proportion of the global health burden worldwide with aging, which is pathologically characterized by early prominent death of dopaminergic neurons in the substantia nigra pars compacta^1^. Although tremendous efforts, including understanding the nature of and relieving the symptoms, have been made, knowledge of disease mechanisms is still limited and no etiological therapies are available^2,3^.

The combination of genetic factors, environmental factors, and aging has contributed to the complex etiology of PD^4^. Among them, genetic factors have been proven to play a prominent role in the pathogenesis of PD. On the one hand, from the perspective of causative genes, rare variants in more than 20 genes have been reported to cause PD^5–7^. On the other, over 90 common variants identified are associated with PD risk, or modified phenotypes, such as age at onset, or progression via genome-wide association studies (GWASs)^8^. In the current study, we proposed to identify causal genes driving the genetic risk of PD with GWAS summary statistics.

Recent research has proposed gene expression quantitative trait loci (eQTL) or protein quantitative trait loci (pQTL) as functional intermediates to investigate the underlying biological mechanisms of genetics on neurodegenerative disorders^9–11^. eQTL and pQTL are the important multiple omics integration data to uncover genetic variants that explain variations in gene and protein expression levels. The measurement of these markers could reflect an individual’s health status and potentially provide novel insights into the effects of diseases. Monitoring expression changes of downstream genes and proteins is also critical for developing potential drug targets. Mendelian randomization (MR) is a genetic method that utilizes genetic variants to address causal questions about how modifiable exposures influence different outcomes^12^. It has been widely used in exploring the etiologies of complex diseases. Likewise, suppose we choose the single nucleotide polymorphisms (SNPs) associated with eQTL or pQTL as instrumental variables (IVs), in that case, we can infer the direct causal effect of the gene expression or protein level on PD, which will help discover the novel risk genes and the pathogenesis, and develop therapeutics based on gene targets since drugs with genetic support are more likely to succeed in clinical trials^13,14^.

Recent two studies analyzed the druggable genome in PD, mainly based on eQTL in blood and brain tissue^15^, or pQTL in blood^16^. Another study performed MR analysis of a genomic atlas based on pQTL in the brain, cerebrospinal fluid (CSF), and plasma to identify risk genes for PD^17^. Although genetic data from brain samples is optimal for research into neurodegenerative diseases, peripheral blood is deemed as a reasonable surrogate that avoids several limitations related to postmortem brain tissue^18^. Meanwhile, integrated analysis for multi-omics from multi-tissues would reduce the effect of false-positive or false-negative genetic data from single-omics or single tissues. Hence, in the current study, we applied a pipeline combing MR design, Steiger filtering analysis, Bayesian colocalization analysis, fine mapping and enrichment analysis to explore the novel causal genes for PD with eQTL and pQTL datasets from the human brain, CSF and blood; moreover, we also evaluated the safety of potential therapeutic targets, which could further provide the genetically-supported drug targets for managing PD.

## Results

### Proteins causally associated with PD in the brain

MR analysis of brain dorsolateral prefrontal cortex (dlPFC) pQTLs identified six genetically determined significant proteins on PD after multiple testing corrections (*P* < 8.55E-05 (0.05/585)). Specifically, the increased abundance of 3 proteins was significantly associated with an increased risk of PD, including GPNMB (OR:1.464, 95%CI: 1.280–1.675, *P* = 2.48E-08), SEC23IP (OR:7.880, 95%CI: 3.020–20.559, *P* = 2.45E-05) and ARSA (OR: 1.938,95%CI:1.394–2.696, *P* = 8.37E-05), while the increased protein abundance of the other 3 genes was significantly associated with a decreased risk of PD, namely CD38 (OR:0.319, 95%CI:0.237–0.431, *P* = 6.99E-14), DGKQ (OR:0.136 95%CI:0.071–0.261, *P* = 1.97E-09), and CTSB (OR:0.300, 95%CI: 0.092–0.433, *P* = 4.39E-05). Moreover, Steiger filtering analysis indicated the true causality (Table 1). After colocalization analysis, all five proteins, except for CTSB, shared a single variant with PD with PPH4 > 80% (Table 1). In addition, another 48 proteins met the suggestive differential expression by MR analysis (*P* < 0.05; Supplementary Table 1). Moreover, ARSA (OR:1.865, 95%CI:1.332–2.611, *P* = 2.86E-04) and CTSB (OR:0.279, 95%CI:0.145–0.460, *P* = 1.44E-04) were also validated to be causal genes for PD using Banner’s pQTL dataset.

**Table 1:** Candidate genes showing robust evidence (passed the Bonferroni correction) in the MR and Bayesian colocalization.

Tissue	Gene	Method	nsnp	*P*-value	OR (95% CI)	COLOC PPH4	Steiger *p*-value	Correct causal direction	Replicated in the banner’s pQTL (N = 152)	Replicated in Akbarian’s eQTL(N = 1866)	Replicated in the Sieberts’s eQTL (N = 1433)	FOCUS
Brain pQTL	*CD38*	Wald ratio	1	6.99E-14	0.319(0.237–0.431)	99.9%	4.199E-11	True	NA	Yes	Yes	Yes
	*CTSB*	Wald ratio	1	4.39E-05	0.201(0.093–0.433)	78.6%	5.978E-11	True	Yes	Suggestive	No	NA
	*DGKQ*	Wald ratio	1	1.97E-09	0.136(0.071–0.261)	99.4%	7.653E-12	True	NA	Suggestive	Suggestive	Yes
	*GPNMB*	Wald ratio	1	2.48E-08	1.465(1.281–1.675)	98.4%	4.508E-20	True	NA	Yes	Yes	Yes
	*ARSA*	Wald ratio	1	8.37E-05	1.938(1.394–2.696)	88.8%	6.730E-17	True	Yes	Suggestive	Suggestive	NA
	*SEC23IP*	Wald ratio	1	2.45E-05	7.880(3.020–20.559)	97.3%	2.057E-13	True	NA	No	NA	No
CSF pQTL	*CTSB*	Wald ratio	1	2.19E-05	0.191(0.089–0.410)	97.0%	5.565E-26	True	NA	NA	NA	NA
	*CD84*	Wald ratio	1	2.23E-05	0.053(0.014–0.207)	97.3%	9.094E-12	True	NA	NA	NA	NA
	*ENTPD1*	Wald ratio	1	3.16E-08	26.240(8.246–83.500)	99.8%	7.873E-10	True	NA	NA	NA	NA
	*GPNMB*	Wald ratio	1	3.95E-07	2.368(1.167–3.305)	95.0%	8.132E-67	True	NA	NA	NA	NA
	*FCGR2B*	Wald ratio	1	6.63E-05	1.346(1.163–1.557)	94.9%	1.865E-77	True	NA	NA	NA	NA
Blood pQTL	*SNCA*	Wald ratio	1	6.26E-25	0.418(0.354–0.494)	99.1%	1.143E-13	True	NA	Suggestive	NA	NA
	*BAG3*	Wald ratio	1	2.37E-05	1.588(1.282–1.969)	96.9%	5.334E-09	True	NA	No	NA	NA
	*FCGR2A*	IVW	3	1.76E-05	1.061(1.033–1.090)	76.2%	4.505E-233	True	NA	No	NA	NA
	*GPNMB*	Wald ratio	1	1.03E-07	1.642(1.368–1.972)	37.5%	2.142E-19	True	NA	Suggestive	NA	NA

“Yes” means the genes were replicated in the eQTL dataset and passed the Bonferroni correction (*p* < 0.05/the number of genes included in MR); “Suggestive” means the genes were replicated in the eQTL dataset (*p* < 0.05, but not passed the Bonferroni correction); *NA* Not Applicable, *FOCUS* fine mapping of the causal gene sets.

To provide an additional layer of insight into our identified genes at the protein level, we wondered whether the mRNA levels of those significant genes were also relevant to the risk for PD using the eQTL from human brain tissue **(**Supplementary Table 2). Notably, the causal effect of 2 genes, namely *GPNMB* and *CD38*, were replicated in the MR analysis and displayed the same direction of causal effect as in the pQTL (*GPNMB*: OR:1.465, *P* = 2.48E-08; *CD38*: OR:0.499, *P* = 1.20E-13), and also passed both the colocalization and Steiger filtering analysis (Table 1). Furthermore, at the brain eQTL level, 25 additional protein-coding genes, showed robust evidence for association with the risk for PD ((*P* < 0.05/8033, Supplementary Table 2). Interestingly, among them, 2 well-known genes, *MAPT* (OR:1.76, *P* = 6.89E-21) and *LRRK2* (OR:6.62, *P* = 3.16E-08) were identified to increase PD risk^19,20^. However, eQTL of the other 3 genes (*ARSA, DGKQ* and *CTSB*) only showed supportive evidence for their causal role in PD (0.05/8033 <*P* < 0.05). Moreover, *GPNMB* (OR:0.898, 95% CI: 0.864–0.932, *P* = 1.16E-08) and *CD38* (OR:1.256, 95%CI:1.182–1.335, *P* = 1.50E-13) were also validated to be causal genes for PD using another eQTL dataset.

### Proteins causally associated with PD in CSF

After multiple testing corrections (*P* < 0.05/585), the MR analysis identified 5 proteins in the CSF which had causal effects on PD (Table 1). Specifically, the increased abundance of 3 proteins was significantly associated with an increased risk of PD, namely ENTPD1 (OR:26.240, 95%CI:8.246–83.500, *P* = 3.16E-08), GPNMB (OR:2.368, 95%CI:1.167–3.305, *P* = 3.95E-07) and FCGR2B (OR:1.346, 95%CI:1.163–1.557, *P* = 6.63E-05), while the increased abundance of 3 proteins was significantly associated with a decreased risk of PD, namely CTSB (OR:0.191, 95%CI:0.089–0.410, *P* = 2.19E-05) and CD84 (OR:0.053,95%CI:0.014–0.207, *P* = 2.23E-05). Moreover, Steiger filtering analysis indicated direct causal associations from changes in protein to the development of PD. And in colocalization analysis, all these 5 significant proteins were found to share a single variant with PD with PPH4 > 80% (Table 1). Furthermore, an additional 13 proteins in the CSF showed a suggestive causal role for PD (0.05/585 < *P* < 0.05), with the increased abundance of eight proteins decreasing the risk for PD, and 5 proteins, including *ARSA*, increasing the risk for PD (Supplementary Table 3).

### Proteins causally associated with PD in the blood

After multiple testing corrections (*P* < 0.05/2051), the MR analysis identified 4 proteins in the blood which had causal effects on PD (Table 1). Specifically, 3 increased protein abundance was significantly associated with an increased risk of PD, namely *BAG3* (OR:1.588, 95% CI: 1.282–1.969, *P* = 2.37E-05), *GPNMB* (OR:1.642, 95% CI:1.368–1.972, *P* = 1.76E-05) and *FCGR2A* (OR:1.061, 95% CI:1.033–1.090, *P* = 1.76E-05), while the increased *SNCA* abundance significantly decreased PD risk (OR:0.418, 95% CI:0.354–0.494, *P* = 6.26E-25). In addition, Steiger filtering showed that all MR-identified proteins indicated direct causal associations from changes in protein to the development of PD. However, only *SNCA* and *BAG3* shared a single variant with PD with PPH4 > 80% in the colocalization analysis, (Table 1). Moreover, an additional 68 proteins in the blood showed a suggestive causal role for PD (*P* < 0.05), with the abundance of 32 proteins, including CTSB, decreasing the risk for PD, and 32 proteins increasing the risk for PD (Supplementary Table 4).

Notably, in the transcriptional level of blood genes, we failed to replicate the above 4 significant proteins on PD after multiple tests (*P* < 0.05/13514, Supplementary Table 5). However, *GPNMB* and *SNCA* showed supportive evidence (*P* < 0.05). Moreover, at the transcriptional level, 18 protein-coding genes were found to be associated with the risk for PD after multiple tests, with 8 genes decreasing the risk for PD, and 10 genes increasing the risk for PD (Supplementary Table 5).

### Results of the fine mapping

With the FOCUS method, CD38 (*P* = 5.83E-12, TWAS-Z = −7.06), DGKQ (*P* = 1.86E-22, TWAS-Z = 0.46) and GPNMB (*P* = 1.40E-12, TWAS-Z = 5.61) in the brain were validated to be causal genes for PD regardless of the linkage disequilibrium (LD) and pleiotropy.

### Summary findings

**Fig. 1:**
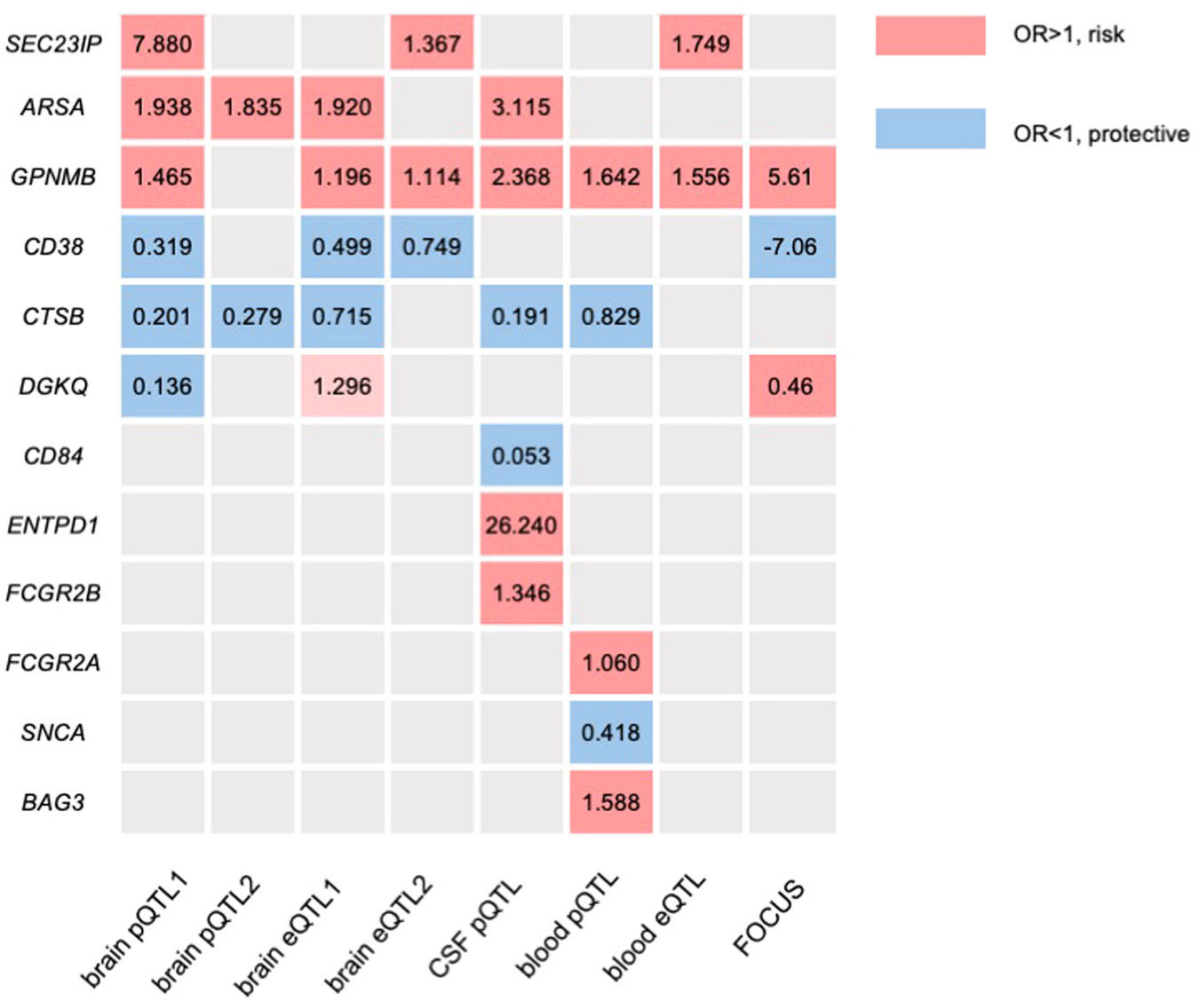
Heatmap of MR results. Heatmap showing the P values of the shared genes identified by PD GWAS, brain pQTL MR analysis, and eQTL MR analysis and blood eQTL MR analysis. This figure shows the specific pipline and the data set involved in this study.

Comparing the genes identified in pQTL and eQTL analysis (Fig. 1), we found that GPNMB displayed a robust causal role for PD at the transcriptional and protein level from blood, CSF, and brain, as well as validated by fine mapping. Likewise, the increased expression of CD38 shows a protective role towards PD was confirmed by brain pQTL and eQTL, which was also validated by fine mapping. Interestingly, although validated by fine mapping, pQTL and eQTL MR analyses of DGKQ showed inconsistent effects on PD, where the increased protein level of DGKQ in the brain was shown to be protective towards PD, while the increased mRNA level of DGKQ was leading to a higher risk of PD. Moreover, although not validated by fine mapping, the causal role of ARSA and SEC23IP were revealed by brain pQTL and suggested by other subsets, and the increased expression of CTSB shows a protective role towards PD was confirmed by the brain and CSF pQTL, and suggested by brain eQTL. Additionally, another 6 proteins (CD84, ENTPD1, FCGR2B, BAG3, SNCA, FCGR2A) were associated with the risk for PD based on a single pQTL subset, but are still worthy of note (Table 1, Fig. 1).

### Consistency comparison by correlation analysis

To further understand the correlation between brain-based, CSF-based and blood-based proteins and genes, we compared the MR effect estimates of the commonly identified proteins and genes. At the protein level, the MR effects between brain proteins and CSF proteins showed a robust positive correlation, and the correlation was strengthened when the limited *p*-value threshold to 0.05 (Supplementary Fig. 1a, b). The MR effects between blood and CSF proteins showed a robust positive correlation at no *p*-value threshold while increasing the p-value threshold to *p* < 0.05 led to no correlation (Supplementary Fig. 1c, d). Moreover, the MR effects between blood and brain proteins showed no correlation at either a p-value threshold or 0.05 threshold (Supplementary Fig. 1e, f). It can be seen that the brain and CSF have a stronger correlation than blood. The differential expression between those tissues and the presence of the blood–brain barrier may contribute to this phenomenon^21^.

At the transcriptional level, we found a robust positive correlation between the brain and blood MR effects. Increasing the p-value threshold resulted in a still robust and higher correlation between brain and blood eQTL (Supplementary Fig. 1g–j), which were consistent with the previous results^22^, and suggested that whole blood could be a proxy for gene identifications in brain-related traits^22^.

### Protein-protein interaction network and enrichment analysis

**Fig. 2:**
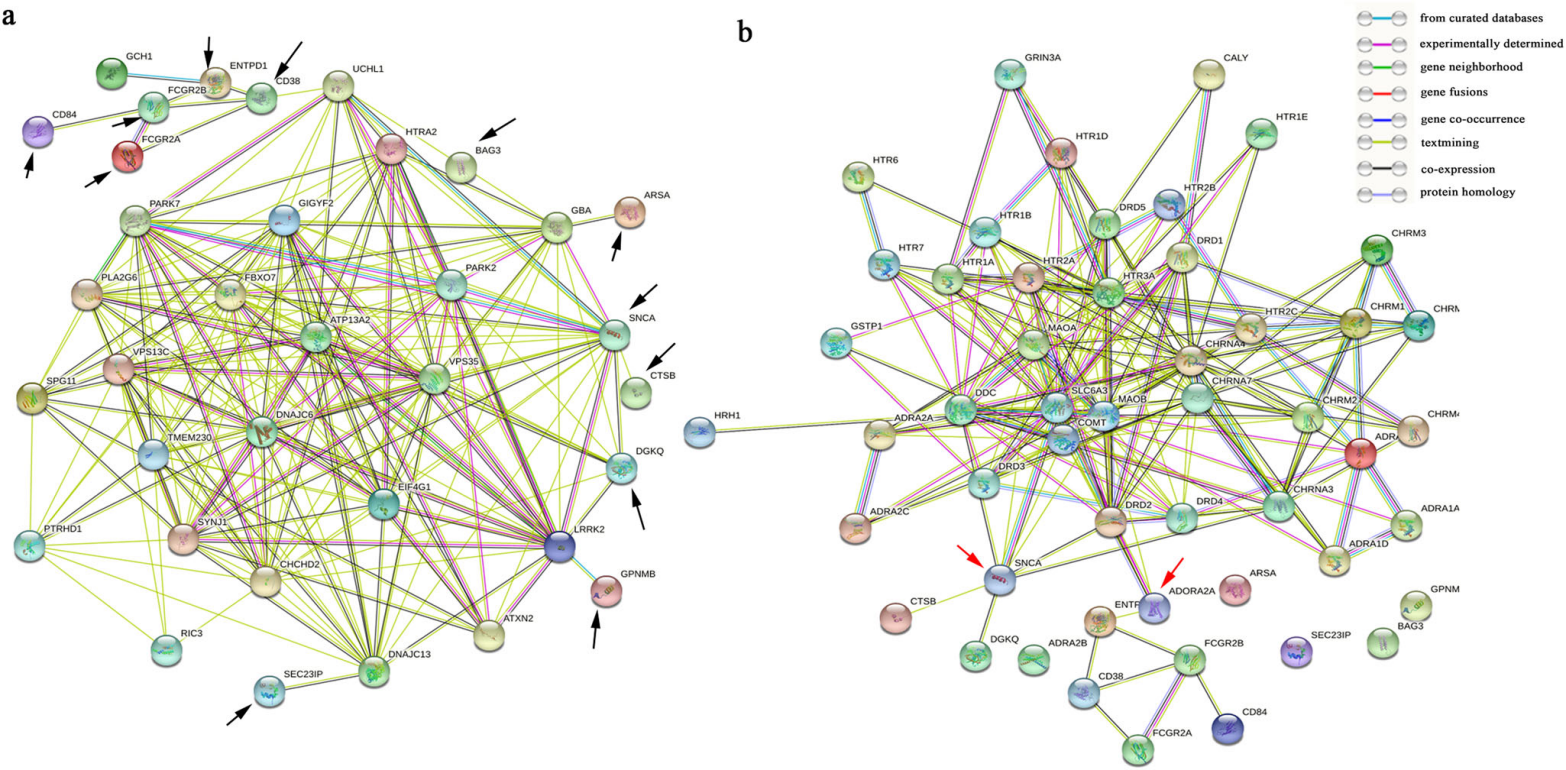
The Protein-protein interaction network using MR-identified proteins passed multiple corrections and PD causative/risk genes, or PD drug targets. **a** The Protein–protein interaction network using MR-identified proteins passed multiple correction and PD causative/risk genes. The black arrow means MR-identified proteins passed multiple corrections. **b** The Protein–protein interaction network using MR-identified proteins passed multiple correction and PD drug targets. The red arrow means Drug targets that interact with MR-identified proteins passed multiple corrections.

Using MR-identified suggestive proteins (*p* < 0.05) from the brain, CSF and blood via the STRING database, respectively, three Protein-protein interaction (PPI) networks were obtained. The statistical enrichment analysis incorporated in STRING revealed that the whole network was significantly enriched (P_Brain_ = 3.43E-05, P_CSF_ = 4.48E-05 and P_blood_ = 1.44E-15, respectively) (Supplementary Fig. 2–4). When comparing the three networks, we found some proteins could actively participate in the interaction network. Thus, we further conducted another PPI network using MR-identified proteins that passed multiple corrections based on all three pQTLs. We found the brain-based protein CD38 was interacted with by the CSF-based protein *FCGR2B* and *ENTPD1*, as well as the blood-based protein *FCGR2A*; brain-based *DGKQ* and *CTSB* both have interaction with blood-based protein *SNCA* (Supplementary figure 5). Moreover, by performing PPI with known PD-causative genes, we found that the top significant genes could interact with several known PD-causative genes, such as *GPNMB* with *LRRK2*, *SEC23IP* with *DNAJC13*, *ARSA* with *GBA* and *CD38* with *UCHL1* (Fig. 2a). On the other hand, by performing PPI with putative PD therapeutic targets, we found PPI network exists between significant risk genes and anti-parkinsonism drug targets, such as CTSB and DGKQ can interact with the dopamine network with SNCA, and CD38, CD84, FCGR2A, FCGR2B, and ENTPID could interact with the dopamine network ADORA2A (Fig. 2b). In the pathway enrichment analysis, we found that suggestive causal genes in the brain were enriched in the “protein dephosphorylation” pathway. Furthermore, in the cell-type-specific expression analysis, *CD38* was mostly expressed in astrocytes, *CTSB* was enriched in microglia, while *SEC23IP* and *DGKQ* were most expressed in the glutamatergic neuron (Supplementary Fig. 6).

### Safety evaluation of the potential therapeutic target

Because MR, colocalization and fine mapping analysis all supported CD38, GPNMB and DGKQ in the brain to be possible causal proteins for PD, we evaluated their safety as possible therapeutic targets. Overall, no significant side effects were identified (*P* < 0.05/782) by performing a broader MR screening of 782 non-PD traits (Supplementary Fig. 7, Supplementary Table 6–8). However, here we observed some trends. Higher CD38 levels may potentially contribute to some ischemic heart diseases and other disorders (Supplementary Table 6). In addition, higher levels of DKGQ may benefit dysmenorrhea (OR:0.118, *P* = 0.0014, Supplementary Table 7). Brain GPNMB levels were most likely associated with stricture of the artery (OR:2.112, *p* = 0.0001, Supplementary Table 8).

## Discussion

Although advances have been made in sequencing methods and bioinformatics tools, only a small proportion of PD patients can be identified with genetic causes. The current study applied a systematic pipeline via multi-omics analysis expanding causal proteins for PD. In summary, evidence from MR, colocalization and fine mapping supported that *GPNMB* showed a genetically causal role for PD, and *DGKQ* and *CD38* may have a protective function. However, another eight proteins were associated with the risk for PD only in one subset after multiple corrections (ARSA, SEC23IP, CD84, ENTPD1, FCGR2B, BAG3, SNCA, FCGR2A), except ARSA and SEC23IP also passed the suggestive *p*-value (0.05) in other subsets. Additionally, phenome-wide MR indicated that lowering the level of GPNMB in the brain and increasing the level of CD38 and DGKQ in the brain might be potential therapeutic targets for PD without significant side effects.

Despite the developments of new therapies over recent years, etiological or disease-modifying treatments for PD are still unavailable. Till now, some studies have been performed to find novel risk/causal genes and genetically supported drug targets for PD via MR of the druggable genome^15,23^, but linkage disequilibrium (LD) may confound the accurate identification of causal SNPs in both GWAS and QTL studies, and single QTL may result in exaggerated effect^24^. Compared with those previous studies^15–17,23,25–28^, our study has more additional value with more comprehensive and robust approaches (MR, colocalization, fine mapping, pathway enrichment, cell type expression analysis and druggable safety analysis), as well as more datasets to validate our findings (2 brain pQTLs, 2 brain eQTLs, 1 CSF pQTLs, 1 blood pQTLs, and 1 blood eQTLs). Therefore, there were consistent results between our and their results, while we also had novel findings. For example, Storm et al. applied MR of the blood- and brain-eQTL and found that the expression level of *CD38*, *CTSB*, *GPNMB* and *MAP3K12* have the most robust MR evidence for PD risk^15^, while only *CD38*, *CTSB* and *GPNMB* were replicated in our study. Png et al. applied the same blood pQTL and PD GWAS in our current study, and also found that blood GPNMB level was associated with an increased risk for PD^16^. Besides, Yang et al. found that plasma IDUA protein level was associated with an increased risk for PD^17^. While we did not identify IDUA protein as a risk factor for PD in any QTL after multiple testing. These inconsistencies might be caused by the different exposure and outcome datasets, and methods (Supplementary Table 9).

Of note, these findings may help to elucidate novel pathogenesis and biomarkers of PD. *GPNMB*, in concordance with previous researches^15,16,23,27,29^, showed robust evidence for PD risk in this study, which was detected in all tissues at both eQTL and pQTL levels. GPNMB is a transmembrane glycoprotein that releases a soluble signaling peptide when cleaved by ADAM10 or other extracellular proteases and was first identified as a risk locus for PD by a 2-stage meta-analysis^30^. Functional studies found that GPNMB protein is selectively elevated in the substantia nigra of PD patients and increased after lysosomal stress^31^. A recent study applied sing-cell sequencing for microglia of idiopathic PD patients and revealed a pro-inflammatory trajectory characterized by elevated levels of GPNMB^32^. Consistent with these studies, our data further suggest that an increased level of GPNMB could serve as a druggable target for PD.

Notably, we found a novel protein, ARSA in the brain, was causal for PD. Initially, homozygous or compound heterozygous mutations in *ARSA* can lead to metachromatic leukodystrophy, an autosomal recessive lysosomal storage disease^33^. ARSA can be detected in neurons, glial cells and blood cells within the blood vessels, but there were no significant differences between controls and PD patients’ neurons^34^. In plasma, the authors found that PD patients exhibited higher plasma ARSA levels than controls. ARSA depletion induces accumulation, secretion and propagation of a-synuclein aggregates by acting as an a-synuclein chaperone in vitro and vivo experiments^34^. However, after multiple testing, we detected no causal effect of it in the brain eQTL and CSF pQTL. It is largely unknown whether plasma ARSA levels or activity correlate with the respective ARSA levels or activity in the brain or CSF of PD patients and healthy controls^35^. The effect of intracranial ARSA may be inconsistent with its mechanism of it in plasma. Besides, plasma ARSA levels gradually decrease with PD progression^36^, while its changes in the brain and CSF need to be further studied. Together with our results, an increased level of ARSA in the brain but the deceased level in plasma might be a biomarker for PD progression, but, importantly, which effect on PD in different tissues needs further studies. In addition, another novel protein, SEC23IP in the brain was also found to be a risk protein for PD. SEC23IP, also known as p125^37^, is associated with spastic paraplegia 28 and nodular malignant melanoma^38,39^. However, previous studies have not suggested that it affects PD. An earlier meta-analysis of whole-exome sequencing data found mutation enrichment in SEC23IP has trends to act a role on PD, but failed to survive after multiple testing correction^40^, so did Nalls et al.^41^. Our study, from a big data, multi-method and multi-omics perspective, may be more useful in identifying novel potential PD-associated genes. Additionally, this protein encoded by *SEC23IP* is localized to endoplasmic reticulum exit sites and plays a critical role in ER-Golgi transport as part of the multimeric coat protein II complex, and is involved in cholesterol trafficking from the plasma membrane to mitochondria^42^. In PD, it is believed to be the consequence of an ER-Golgi transport imbalance and/or cytoskeleton alterations^43^. Therefore, it might contribute to the risk for PD via the vesicle trafficking pathway, and the more in-depth basic research is promising.

Moreover, our study, importantly, also detected that some genes might play a protective role in PD. We strongly recommended CTSB, consistent with previous studies finding that brain expression of CTSB likely decreased the risk for PD^15,27,44^. In addition, CTSB reduced the penetrance of PD patients with *GBA* variants^45^. What’s more, CTSB belongs to the cathepsin family, which is important in the lysosomal degradation of α-synuclein^46^. In addition, as for CD38, consistent with the results from the previous eQTL MR and TWAS study^15,27,47^, it was further found in pQTL in our study. It was reported that CD38 expression increases with aging, which is otherwise the primary risk associated with neurodegenerative diseases^48^. Several experimental data demonstrated that *CD38* knockout mice are protected from neurodegenerative and neuroinflammatory insults^49,50^. Overall, these results indicate that increasing the level of CTSB and CD38 in the brain might be a promising therapeutic target for PD.

However, we found inconsistent roles of DGKQ on PD, where the increased protein level of DGKQ in the brain was shown to be protective towards PD, while the increased mRNA level of DGKQ was leading to a higher risk of PD. DGKQ has been found to be a risk locus for PD by the previous GWAS^51^. It belongs to the diacylglycerol kinases family, which contain enzymes that catalyze the ATP-dependent phosphorylation of diacylglycerol (DAG) to phosphatidic acid (PtdOH)^52^. The function of DGKQ has been rarely studied, while it is localized to excitatory synapses where its kinase activity promotes retrieval of synaptic vesicles following neuronal activity^52^, which supports our findings that DGKQ protein level was protective in the brain. But for the inconsistent results between protein and mRNA levels, several reasons might account for this. Firstly, differences in equipment, reagents and statistical methods might contribute to inaccuracies in the QTL data. Secondly, the variance of sample sizes and the number of genes in the pQTL and eQTL datasets may also play a role. Moreover, these inconsistent roles between mRNA and protein levels might be caused by post-transcriptional modifications such as mRNA splicing and protein degradation^53^. Last but not least, the mRNA abundance in the eQTL dataset might be a specific DGKQ mRNA isoform that is targeted for degradation prior to translation. However, function studies were needed to ascertain DGKQ’s role in PD.

Our study would help to find novel drug targets for PD. In the PPI network analysis, we found that the identified proteins in our study can interact with the known PD causative genes, such as *GPNMB* with *LRRK2* and *CD38* with *UCHL1*. This evidence suggested that the top significant genes could involve in the pathogenesis of PD through known PD pathways. Furthermore, we found that although some top significant proteins (CTSB, DGKQ, SNCA, CD38, CD84, FCGR2A, FCGR2B, and ENTPID) could interact with the dopamine network and ADORA2A could interact with the known PD drug targets, while other proteins (GPNMB, ARSA, and BAG3) were unable to participate in the network. These findings shed new light on potential drug targets by linking genetic factors to disease and known targets. Moreover, in the pathway enrichment analysis, we found that PD causal proteins in the brain were enriched in the “protein dephosphorylation” pathway. PD is a neurodegenerative disease characterized by aberrant accumulation of misfolding a-synuclein in the brain. Phosphorylation at some residues such as Ser129 has been suggested to have a close relationship with a-synuclein degradation and aggregation, while it is still unclear whether phosphorylation promotes or prevents aggregation and toxicity^54^. Together with our results, the protein dephosphorylation pathway might be a therapeutic target for PD, while further studies were needed. Furthermore, in the cell-type-specific expression analysis, besides neurons, we found that PD-causal proteins were also expressed in astrocytes and microglia. These results supported previous findings that astrocytes and microglia play important roles in maintaining the microenvironment in the brain, and dysfunction of these glial cells has been implicated in the pathogenesis of PD^55^. Therefore, the cross-talk between neurons and glial cells in PD pathogenesis should get more attention.

With MR and fine mapping method, we found that an increased level of GPNMB in the brain was leading to a higher risk for PD, while increased levels of CD38 and DGKQ in the brain were leading to a lower risk of PD. These results indicated that lowering the level of GPNMB in the brain and increasing the level of CD38 and DGKQ in the brain might be potential therapeutic targets for PD. Therefore, we explored the potential side effects of lowering the level of GPNMB in the brain and increasing the level of CD38 and DGKQ in the brain. And no significant side effect was noted. These results suggested that CD38, DGKQ, and GPNMB might be promising therapeutic targets for PD, while further studies were needed.

The study also has some limitations. First, the brain tissue used in our study was limited to the human parietal lobes, while some other brain regions were found to be more relevant to PD, such as basal ganglia, basal ganglia work closely in concert with the cortex and cerebellum^56^. But the sample size of eQTL in basal ganglia was small^47^. Therefore, datasets from these regions of the brain would be needed. Second, there was no validation cohort to confirm the results, which might leverage false-positive findings. However, we applied the systematic multiple pipelines, including MR, Steiger filtering analysis, Bayesian colocalization analysis and fine mapping to confirm the results, and our results also replicate findings from some previous studies. Thirdly, the different sample sizes of the QTL datasets might result in variable statistical power for each study, leading to errors when comparing the results in different tissues. Last but not least, the current study focused on the genes conferring PD risk. However, attention also should be paid to genes responsible for PD progression, which might be targeted for disease-modifying therapies. Therefore, MR studies using gene profiles as exposures and PD progression^57^ as outcomes were needed in further studies.

In conclusion, with multi-omics from multiple tissues, our study identified 3 brain-based proteins (GPNMB, CD38, and DGKQ) to be associated with the risk for PD at the protein or transcriptional levels. These findings would help uncover the genes underlying PD and prioritize targets for future therapeutic interventions. However, further studies are needed to repeat this finding and explore the underlying biological mechanisms associated with the identified genes.

## Methods

### Datasets

**Fig. 3:**
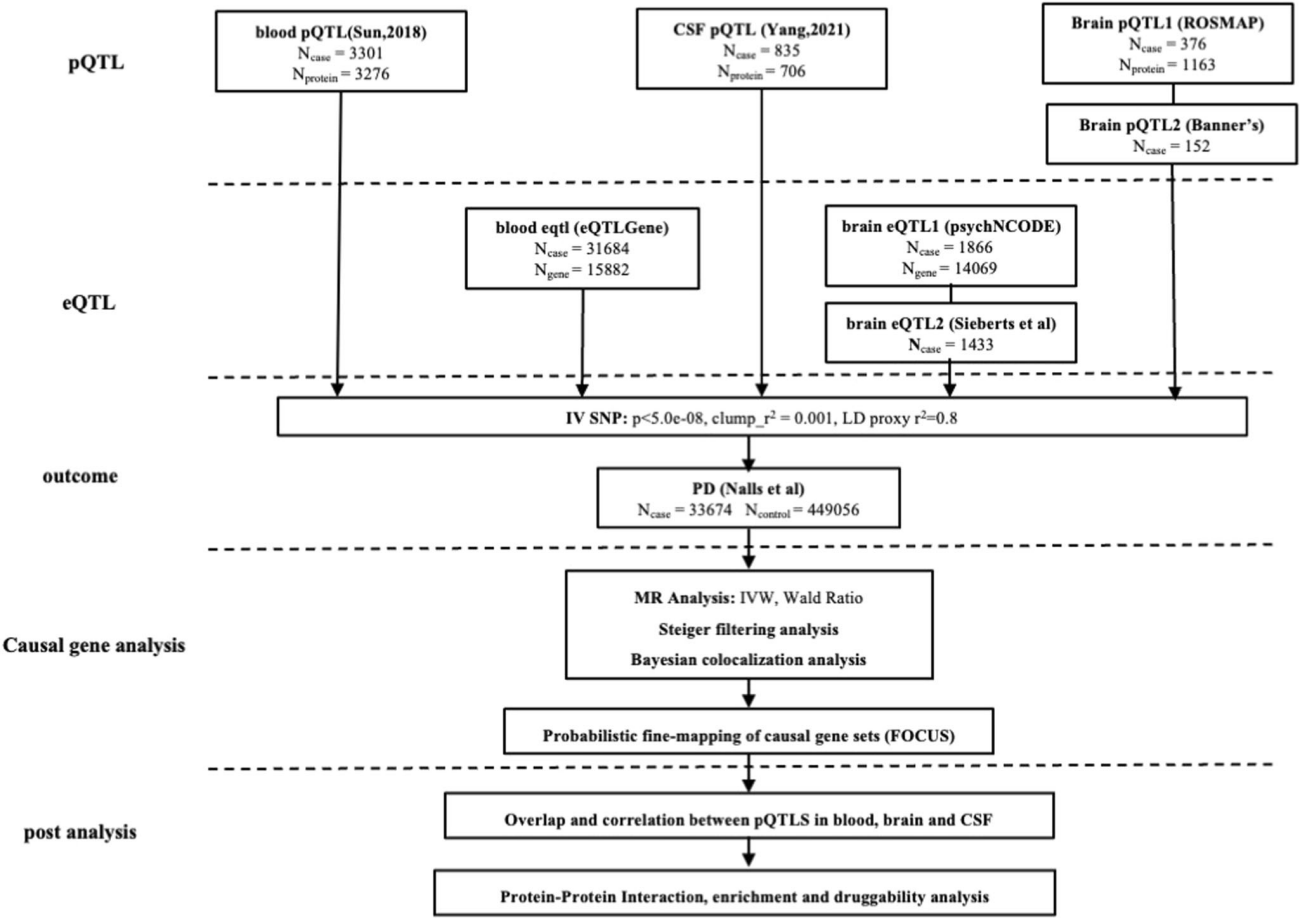
The design flow of the study.

The information about the datasets used in the current study is listed in Supplementary Table 10. The detailed flowchart of this study is illustrated in Fig. 3.

Human brain-tissue derived pQTL data (brain pQTL1) was obtained from a previous study^58^, which performed a proteome-wide association study (PWAS) by generating human brain proteomes from the dorsolateral prefrontal cortex (dlPFC) of postmortem brain samples donated by 400 participants of European ancestry of the Religious Orders Study/Memory and Aging Project (ROS/MAP) cohorts^58,59^. After quality control, 376 subjects and 1475 proteins were eligible for the PWAS analysis, and 607 proteins have significantly associated SNPs (*p* < 5E-08)^58^. Moreover, to strengthen our findings, we also validated the significant proteins in another dataset with 152 participants (brain pQTL2)^60^. The detailed information for the proteomic sequencing, genotyping and analyses were described in the original study^58^.

The brain eQTL data was obtained from the PsychENCODE Consortium (brain eQTL1), which collected data from the human brain of European ancestry (*n* = 1866) and covered 10,489 genes that have significantly associated SNPs with *p* < 5E-08^61^. Moreover, we also validated the significant genes in another dataset with 1433 participants (brain eQTL2)^62^.

The available CSF pQTL dataset measured the abundance of 1305 proteins using a multiplexed aptamer-based platform17 in CSF samples (*n* = 971), after quality control, 835 CSF samples and 713 proteins were eligible for subsequent analysis. The details on recruitment and quality control are available in the original publication^17^. Using this pQTL dataset, we found 217 CSF proteins which have significantly independent local pQTLs with *p* < 5E-08.

The plasma proteome was obtained from the INTERVAL study^63^, which performed genome-wide testing of 10.6 million imputed autosomal variants against levels of 2994 plasma proteins in 3301 individuals of European descent.

The whole blood eQTL data was from the eQTLGen consortium, consisting of 31,684 blood and peripheral blood mononuclear cell (PBMC) samples from 37 eQTLGen Consortium cohorts, and covering 19,942 genes^64^.

The PD dataset was obtained from the publicly available summary statistics from the latest and largest case-control genome-wide association meta-analysis of PD published in 2019 by the International Parkinson’s Disease Genomics Consortium (IPDGC) (excluding 23andMe data), which included 15056 PD cases, 18618 UK Biobank proxy-cases (i.e., subjects with a first degree relative with PD) and 449056 controls of European ancestry^26^.

### Two-sample mendelian randomization

MR analysis utilizes genome-wide significant SNPs as IVs to explore the causal effects of defined exposure on an outcome. It has been widely applied in identifying the genetic etiology of complex illnesses through integrating the quantitative trait loci data^21,65^. In this study, we used the QTL datasets as the exposure and PD GWAS as the outcome to identify novel causal genes and proteins for PD.

Three key assumptions must be met in selecting eligible IVs, which is the first and most important step to perform MR^66^. Assumption 1 (relevance assumption) requires the SNPs to be strongly associated with the exposure. Therefore, we adopted the genome-wide significance threshold *p* < 5E-08 to filter the SNPs in eQTLs and pQTLs. Assumption 2 (independence assumption) requires the IVs to not be associated with confounding factors, which can be calculated as pleiotropy in the post-MR analysis. Assumption 3 (exclusion assumption) requires the IVs to not be directly associated with the outcome. Therefore, to meet assumption 3, we searched the phenoscanner database to remove IVs that were directly associated with PD^67^.

Once the eligible IVs were selected, they were linkage disequilibrium (LD) clumped with *r*^2^ < 0.001 in a 10 megabase distance. And then the IVs were harmonized with the outcome. After clump and harmonization, there were usually 1 or 2 IVs for each exposure. Then, MR effects can be estimated. Wald ratio method was applied if only a single IV was available, inverse variance weighted (IVW) method was performed if 2 IVs were available^66^. The suggested threshold of *P*-value < 0.05 and Bonferroni correction thresholds (*P* < 0.05/number of genes or proteins analyzed) were used to prioritize genes for further follow-up. And because of the limited IV numbers, sensitivity analyses (including MR-Egger, weighted median mode and simple weighted median mode) and post-MR analysis (including pleiotropy test, outlier test and heterogeneity test) were unable to be performed. Moreover, the Steiger filtering analysis was applied to ensure that the effect of direction was from exposure to outcome, but not reverse^68^. The steps mentioned above were implemented using the “TwoSampleMR” R package (github.com/MRCIEU/ TwoSampleMR)^6^.

### Bayesian colocalization analysis

To avoid LD and pleiotropy and ensure the two independent GWAS association signals (pQTL/eQTL and PD) are consistent with a shared causal variant, Bayesian colocalization analysis was further applied^69^. Briefly, the colocalization analysis was performed with R package “coloc”, which provides the posterior probability for five hypotheses regarding whether a single variant is shared between two traits: (1) PPH0, no association with either trait; PPH1, a genetic variant only associated with the trait 1 (eQTL or pQTL), but not with the trait 2 (PD); PPH2, a genetic variant associated with the trait 2 (PD), but not with the trait 1 (eQTL or pQTL); PPH3, association with the trait 1 (eQTL or pQTL) and the trait 2 (PD), with different causal variants; PPH4, association with the trait 1 (eQTL or pQTL) and the trait 2 (PD), with a shared causal variant^69^. pQTL/eQTL and PD are considered to share the same variant if the posterior probability for PPH4 > 80%^21,70^.

### Probabilistic fine-mapping of causal gene sets

To disambiguate the potential of pleiotropy underlying genetic variants associated with the expression of multiple gene products in a given locus, and disambiguate which gene is most likely causal (e.g., looking at genes with high posterior inclusion probabilities), we applied fine-mapping of causal gene sets (FOCUS) to validate the genes in the brain discovered by MR. FOCUS takes as input GWAS summary data, expression prediction weights (as estimated from eQTL reference panels), and LD among all SNPs in the risk region, and estimates the probability for any given set of genes to explain the transcriptome-wide association study (TWAS) signal. We used the FOCUS weights and executed the code according to the manual provided by the original study (https://github.com/bogdanlab/focus)^71^.

### Pearson Correlation, protein-protein network, pathway enrichment and cell-type specific expression analysis

As previous study^21^, we wondered whether there would be correlations between the brain, CSF and blood-identified QTLs. Hence, we investigated the correlation between the shared QTLs identified in the brain, CSF and blood using effect estimates from the MR analysis by Pearson correlation analysis. In consideration that the number of pQTLs was much smaller than that of eQTLs, and no genes were shared between the brain, CSF, and blood pQTL at a threshold of *p* < 0.05, we set only no threshold for pQTLs and three for eQTLs (*P* < 0.05, 0.01 and 0.005) to ensure enough number of shared QTLs in the Pearson correlation analysis.

To investigate the interactions between the PD risk genes identified in this study and the known PD causative genes, we explored the protein-protein interaction (PPI) network for these proteins abundance (from pQTLs) was associated with PD risk in MR analysis (p-value passed the Bonferroni correction) and published PD known causative/risk genes (Supplementary Table 11)^5^, which was investigated by using the Search Tool for the Retrieval of Interacting Genes (STRING) database version 11.5 (https://string-db.org/)^72^. Moreover, to explore whether the PD-causal genes were enriched in certain pathways, we performed pathway enrichment of the suggestive PD causal proteins (*p* < 0.05) with the Metascape online software^73^. In addition, to investigate whether interactions exist between these identified risk genes and current PD therapeutic targets, we obtained 18 available PD medications from a previous review^74^ and corresponding drug targets based on the Drugbank database (https://www.drugbank.ca) (Supplementary Table 12)^75^. Moreover, we also explored the cell-type-specific expression of the causal genes in the brain. The cell-type-specific expression profile of the causal genes in the brain was downloaded from the human single-cell RNA-seq data from the cell types database (https://portal.brain-map.org/atlases-and-data/rnaseq). Cell-type expression specificity (CELLEX), a tool for computing cell-type expression specificity (ES) profiles, was applied to capture multiple aspects of ES^76^.

### Safety evaluation of the potential therapeutic targets by phenome-wide MR

As our previous study^77^, in order to assess the potential side effects of therapeutic targets, we utilized therapeutic target gene expression in the brain as the exposure factor and summary statistics of diseases in the UK Biobank cohort (*n* ≤ 408 961) as the outcomes for conducting phenome-wide MR. Disease GWASs from the UK Biobank were conducted using the Scalable and Accurate Implementation of Generalised Mixed Model (SAIGE V.0.29) approach in order to account for imbalanced case/control ratios^78^. Given the statistical power limitations, we selected 782 non-Parkinson’s disease traits (diseases) with over 500 cases for phenome-MR analyses (Supplementary Table 13). The summary statistics of disease-associated SNPs were obtained from the SAIGE GWAS (https://www.leelabsg.org/resources). More detailed information can be found in the publication^78^. Causal effects are considered statistically significant when *p* < 0.05/782 (after applying Bonferroni correction).

### Reporting Summary

Further information on research design is available in the Nature Research Reporting Summary linked to this article.

### Supplementary information

Supplementary materials

Related Manuscript File

## Data Availability

The data used in the current study were obtained from the published articles as referenced in the Methods section and listed in Supplementary Table 10. The analyzed datasets generated during the study are listed in Supplementary Table 1–8.

## Code availability

The code used in the current study is available from the corresponding author upon reasonable request.
