# Supplementary material for: Expanding causal genes for Parkinson’s disease via multi-omics analysis
Source: NPJ Parkinsons Dis. 2023 Oct 21;9:146. doi: 10.1038/s41531-023-00591-0 (PMC10590374; doi:10.1038/s41531-023-00591-0)
Supplement: Supplementary file 1 — Supplementary materials [file 41531_2023_591_MOESM1_ESM.pdf]

Supplementary Table 1 The result of the MR analysis between brain dlPFC pQTLs and PD

gene	outcome	method	nsnp	b	p-value	or	or_lci95	or_uci95
CD38	PD	Wald ratio	1	-1.14	6.99E-14	0.32	0.24	0.43
DGKQ	PD	Wald ratio	1	-1.99	1.97E-09	0.14	0.07	0.26
GPNMB	PD	Wald ratio	1	0.38	2.48E-08	1.46	1.28	1.67
SEC23IP	PD	Wald ratio	1	2.06	2.45E-05	7.88	3.02	20.56
CTSB	PD	Wald ratio	1	-1.61	4.39E-05	0.20	0.09	0.43
ARSA	PD	Wald ratio	1	0.66	8.37E-05	1.94	1.39	2.70
IDUA	PD	Wald ratio	1	0.69	0.000542	2.00	1.35	2.96
AMZ2	PD	Wald ratio	1	-0.52	0.000552	0.60	0.45	0.80
LMBRD1	PD	Wald ratio	1	-1.39	0.001202	0.25	0.11	0.58
ENPP4	PD	Wald ratio	1	-1.22	0.001288	0.30	0.14	0.62
PDLIM2	PD	Wald ratio	1	-0.83	0.00165	0.43	0.26	0.73
ULK3	PD	Wald ratio	1	-0.70	0.002271	0.50	0.32	0.78
SLC25A24	PD	Wald ratio	1	-0.53	0.002661	0.59	0.42	0.83
PPP3CC	PD	Wald ratio	1	0.99	0.00323	2.68	1.39	5.16
CAMLG	PD	Wald ratio	1	0.68	0.004632	1.96	1.23	3.13
FABP1	PD	Wald ratio	1	-0.23	0.004735	0.79	0.67	0.93
FARSB	PD	Wald ratio	1	-1.38	0.007103	0.25	0.09	0.69
SAMM50	PD	Wald ratio	1	1.31	0.007748	3.72	1.41	9.77
TIPRL	PD	Wald ratio	1	2.64	0.011061	14.05	1.83	107.94
RCSD1	PD	Wald ratio	1	-0.92	0.011347	0.40	0.20	0.81
LGALS3	PD	Wald ratio	1	0.53	0.012317	1.69	1.12	2.56
ZADH2	PD	Wald ratio	1	-0.93	0.012569	0.39	0.19	0.82
TXNRD2	PD	Inverse variance weighted	2	0.33	0.015108	1.39	1.07	1.81
SWAP70	PD	Wald ratio	1	-1.14	0.015864	0.32	0.13	0.81
LSS	PD	Wald ratio	1	0.30	0.016048	1.35	1.06	1.73
HBG2	PD	Wald ratio	1	-0.10	0.017279	0.91	0.84	0.98
COMTD1	PD	Wald ratio	1	-0.78	0.017695	0.46	0.24	0.87
GBASNIPSNAP2	PD	Wald ratio	1	-1.17	0.017972	0.31	0.12	0.82
VPS13C	PD	Wald ratio	1	1.52	0.018378	4.59	1.29	16.28
AAMDC	PD	Wald ratio	1	0.21	0.018594	1.24	1.04	1.48
APIP	PD	Wald ratio	1	-0.18	0.01885	0.84	0.72	0.97
ADHFE1	PD	Wald ratio	1	0.24	0.019354	1.27	1.04	1.56
GUF1	PD	Wald ratio	1	0.45	0.020869	1.57	1.07	2.30
FECH	PD	Wald ratio	1	0.69	0.023719	1.99	1.10	3.62
GART	PD	Wald ratio	1	-0.36	0.024522	0.70	0.51	0.96
PFKM	PD	Wald ratio	1	1.12	0.025078	3.05	1.15	8.10
SFXN4	PD	Wald ratio	1	-0.90	0.025243	0.40	0.18	0.89

BNIP1	PD	Wald ratio	1	0.95	0.025837	2.58	1.12	5.93
CCDC92	PD	Wald ratio	1	-1.37	0.027652	0.25	0.08	0.86
CSDC2	PD	Wald ratio	1	-0.33	0.029184	0.72	0.54	0.97
SLC15A2	PD	Wald ratio	1	0.37	0.033648	1.45	1.03	2.04
RASA1	PD	Wald ratio	1	-1.69	0.033964	0.18	0.04	0.88
DPYSL5	PD	Wald ratio	1	-0.97	0.035911	0.38	0.15	0.94
DPP3	PD	Wald ratio	1	1.20	0.036677	3.33	1.08	10.28
NUDCD1	PD	Wald ratio	1	1.04	0.038623	2.82	1.06	7.55
MICAL1	PD	Wald ratio	1	0.72	0.038949	2.05	1.04	4.04
KIAA1161MYORG	PD	Wald ratio	1	0.27	0.039511	1.31	1.01	1.70
GLRX5	PD	Wald ratio	1	0.33	0.039825	1.39	1.02	1.89
FAH	PD	Wald ratio	1	0.30	0.039853	1.36	1.01	1.81
ESD	PD	Wald ratio	1	-0.72	0.041126	0.48	0.24	0.97
GALC	PD	Wald ratio	1	0.20	0.041281	1.22	1.01	1.48
RCN1	PD	Wald ratio	1	0.63	0.042802	1.88	1.02	3.47
TOM1L2	PD	Wald ratio	1	1.10	0.046749	3.01	1.02	8.91
ADA	PD	Wald ratio	1	-0.38	0.047221	0.69	0.47	1.00
COQ7	PD	Wald ratio	1	-0.37	0.048011	0.69	0.48	1.00

Supplementary Table 2 The result of the MR analysis between brain eQTLs and PD

name	gene	outcome	method	nsnp	b	p-value	or	or_lci95	or_uci95
ENSG00000261575	*#N/A*	PD	Wald ratio	1	0.771	2.17E-21	2.16	1.84	2.53
ENSG00000265964	*#N/A*	PD	Wald ratio	1	0.930	2.49E-21	2.54	2.09	3.07
ENSG00000185294	*SPPL2C*	PD	Wald ratio	1	1.041	3.44E-21	2.83	2.28	3.51
ENSG00000186868	*MAPT*	PD	Wald ratio	1	0.565	6.89E-21	1.76	1.56	1.98
ENSG00000214425	*#N/A*	PD	Wald ratio	1	0.257	6.89E-21	1.29	1.23	1.36
ENSG00000204650	*LINC02210*	PD	Wald ratio	1	-1.508	1.35E-20	0.22	0.16	0.30
ENSG00000263142	*LRRC37A17P*	PD	Wald ratio	1	1.501	2.94E-20	4.48	3.26	6.17
ENSG00000262881	*#N/A*	PD	Wald ratio	1	0.955	4.79E-20	2.60	2.12	3.19
ENSG00000176681	*LRRC37A*	PD	Wald ratio	1	-0.259	1.62E-19	0.77	0.73	0.82
ENSG00000204652	*#N/A*	PD	Wald ratio	1	-0.538	7.80E-19	0.58	0.52	0.66
ENSG00000225190	*PLEKHM1*	PD	Wald ratio	1	1.209	5.59E-16	3.35	2.50	4.49
ENSG00000004468	*CD38*	PD	Wald ratio	1	-0.695	1.20E-13	0.50	0.42	0.60
ENSG00000138722	*MMRN1*	PD	Wald ratio	1	0.430	1.63E-12	1.54	1.36	1.73
ENSG00000265315	*RN7SL199P*	PD	Wald ratio	1	-0.464	3.53E-11	0.63	0.55	0.72
ENSG00000265411	*RN7SL656P*	PD	Wald ratio	1	-0.464	3.53E-11	0.63	0.55	0.72
ENSG00000263503	*#N/A*	PD	Wald ratio	1	-0.172	3.53E-11	0.84	0.80	0.89
ENSG00000262539	*#N/A*	PD	Wald ratio	1	-0.213	6.14E-11	0.81	0.76	0.86
ENSG00000267246	*#N/A*	PD	Wald ratio	1	-0.488	6.14E-11	0.61	0.53	0.71
ENSG00000120071	*KANSL1*	PD	Wald ratio	1	-0.380	6.14E-11	0.68	0.61	0.77
ENSG00000214401	*KANSL1-AS1*	PD	Wald ratio	1	-0.253	6.14E-11	0.78	0.72	0.84
ENSG00000120088	*CRHR1*	PD	Wald ratio	1	0.486	1.12E-09	1.63	1.39	1.90
ENSG00000264070	*#N/A*	PD	Wald ratio	1	0.487	1.12E-09	1.63	1.39	1.90
ENSG00000099377	*HSD3B7*	PD	Wald ratio	1	-0.676	4.25E-09	0.51	0.41	0.64
ENSG00000164182	*NDUFAF2*	PD	Wald ratio	1	1.128	2.08E-08	3.09	2.08	4.58
ENSG00000136235	*GPNMB*	PD	Wald ratio	1	0.179	2.48E-08	1.20	1.12	1.27
ENSG00000188906	*LRRK2*	PD	Wald ratio	1	1.890	3.16E-08	6.62	3.39	12.93
ENSG00000136243	*NUP42*	PD	Wald ratio	1	0.221	4.25E-08	1.25	1.15	1.35
ENSG00000226816	*#N/A*	PD	Wald ratio	1	-0.483	4.25E-08	0.62	0.52	0.73
ENSG00000262500	*MAPK8IP1P1*	PD	Wald ratio	1	0.612	1.72E-07	1.84	1.47	2.32
ENSG00000170425	*ADORA2B*	PD	Wald ratio	1	0.366	2.29E-07	1.44	1.25	1.66
ENSG00000141027	*NCOR1*	PD	Wald ratio	1	1.216	2.38E-07	3.37	2.13	5.35
ENSG00000260911	*#N/A*	PD	Wald ratio	1	-0.738	2.80E-07	0.48	0.36	0.63
ENSG00000103510	*KAT8*	PD	Wald ratio	1	-0.439	3.02E-07	0.64	0.54	0.76
ENSG00000103496	*STX4*	PD	Wald ratio	1	1.086	3.13E-07	2.96	1.95	4.49
ENSG00000103549	*RNF40*	PD	Wald ratio	1	-0.761	8.30E-07	0.47	0.35	0.63
ENSG00000196118	*CFAP119*	PD	Wald ratio	1	0.402	2.20E-06	1.50	1.27	1.77
ENSG00000214941	*ZSWIM7*	PD	Wald ratio	1	0.145	3.28E-06	1.16	1.09	1.23
ENSG00000163749	*CCDC158*	PD	Wald ratio	1	-0.324	4.46E-06	0.72	0.63	0.83
ENSG00000178226	*PRSS36*	PD	Wald ratio	1	-0.467	8.43E-06	0.63	0.51	0.77
ENSG00000198171	*DDRGK1*	PD	Wald ratio	1	0.947	2.14E-05	2.58	1.67	3.99
ENSG00000124613	*ZNF391*	PD	Wald ratio	1	0.311	4.07E-05	1.37	1.18	1.58

ENSG00000169242	*EFNA1*	PD	Wald ratio	1	0.740	4.24E-05	2.10	1.47	2.99
ENSG00000219881	*#N/A*	PD	Wald ratio	1	0.788	4.98E-05	2.20	1.50	3.22
ENSG00000169313	*P2RY12*	PD	Wald ratio	1	0.976	6.04E-05	2.65	1.65	4.28
ENSG00000166582	*CENPV*	PD	Wald ratio	1	0.184	8.76E-05	1.20	1.10	1.32
ENSG00000073969	*NSF*	PD	Wald ratio	1	-0.755	0.000106	0.47	0.32	0.69
ENSG00000108379	*WNT3*	PD	Wald ratio	1	-0.508	0.000106	0.60	0.47	0.78
ENSG00000131484	*#N/A*	PD	Wald ratio	1	-0.501	0.000106	0.61	0.47	0.78
ENSG00000185829	*ARL17A*	PD	Wald ratio	1	-0.194	0.000106	0.82	0.75	0.91
ENSG00000238083	*LRRC37A2*	PD	Wald ratio	1	-0.131	0.000106	0.88	0.82	0.94
ENSG00000232300	*#N/A*	PD	Wald ratio	1	-0.238	0.000106	0.79	0.70	0.89
ENSG00000164733	*CTSB*	PD	Wald ratio	1	-0.336	0.000124	0.71	0.60	0.85
ENSG00000162869	*PPP1R21*	PD	Wald ratio	1	-0.312	0.000164	0.73	0.62	0.86
ENSG00000181754	*AMIGO1*	PD	Wald ratio	1	-0.707	0.000174	0.49	0.34	0.71
ENSG00000138002	*IFT172*	PD	Wald ratio	1	0.979	0.000175	2.66	1.60	4.44
ENSG00000030582	*GRN*	PD	Wald ratio	1	-0.894	0.000183	0.41	0.26	0.65
ENSG00000268516	*ZNF8-DT*	PD	Wald ratio	1	-0.655	0.000189	0.52	0.37	0.73
ENSG00000271155	*#N/A*	PD	Wald ratio	1	0.471	0.000212	1.60	1.25	2.06
ENSG00000005884	*ITGA3*	PD	Wald ratio	1	0.708	0.000224	2.03	1.39	2.96
ENSG00000231170	*#N/A*	PD	Wald ratio	1	-0.188	0.000244	0.83	0.75	0.92
ENSG00000100075	*SLC25A1*	PD	Wald ratio	1	0.261	0.000249	1.30	1.13	1.49
ENSG00000111875	*ASF1A*	PD	Wald ratio	1	1.003	0.000264	2.73	1.59	4.67
ENSG00000115234	*SNX17*	PD	Wald ratio	1	1.349	0.000283	3.85	1.86	7.98
ENSG00000143194	*MAEL*	PD	Wald ratio	1	0.146	0.000284	1.16	1.07	1.25
ENSG00000069482	*GAL*	PD	Wald ratio	1	-0.248	0.000366	0.78	0.68	0.89
ENSG00000162552	*WNT4*	PD	Wald ratio	1	-0.489	0.000399	0.61	0.47	0.80
ENSG00000117533	*VAMP4*	PD	Wald ratio	1	0.725	0.000437	2.06	1.38	3.09
ENSG00000168297	*PXK*	PD	Wald ratio	1	-0.485	0.000451	0.62	0.47	0.81
ENSG00000170175	*CHRNB1*	PD	Wald ratio	1	0.347	0.000506	1.41	1.16	1.72
ENSG00000100403	*ZC3H7B*	PD	Wald ratio	1	-0.878	0.000511	0.42	0.25	0.68
ENSG00000066926	*FECH*	PD	Wald ratio	1	0.384	0.000523	1.47	1.18	1.82
ENSG00000261026	*#N/A*	PD	Wald ratio	1	0.499	0.00057	1.65	1.24	2.19
ENSG00000005981	*ASB4*	PD	Inverse variance weighted	2	-0.349	0.000574	0.71	0.58	0.86
ENSG00000162378	*ZYG11B*	PD	Wald ratio	1	0.916	0.000661	2.50	1.48	4.23
ENSG00000235289	*#N/A*	PD	Wald ratio	1	0.394	0.000733	1.48	1.18	1.86
ENSG00000126777	*KTN1*	PD	Wald ratio	1	0.817	0.000771	2.26	1.41	3.64
ENSG00000156958	*GALK2*	PD	Wald ratio	1	-0.805	0.000808	0.45	0.28	0.72
ENSG00000145700	*ANKRD31*	PD	Wald ratio	1	-0.373	0.000858	0.69	0.55	0.86
ENSG00000255046	*#N/A*	PD	Wald ratio	1	0.317	0.000885	1.37	1.14	1.65
ENSG00000101412	*E2F1*	PD	Wald ratio	1	0.651	0.001104	1.92	1.30	2.83
ENSG00000168216	*LMBRD1*	PD	Wald ratio	1	-0.501	0.001126	0.61	0.45	0.82
ENSG00000206053	*JPT2*	PD	Wald ratio	1	0.516	0.001128	1.68	1.23	2.29
ENSG00000247775	*SNCA-AS1*	PD	Wald ratio	1	-0.229	0.00119	0.80	0.69	0.91

ENSG00000115947	*ORC4*	PD	Wald ratio	1	0.532	0.001194	1.70	1.23	2.35
ENSG00000177234	*LINC01561*	PD	Wald ratio	1	-0.260	0.001204	0.77	0.66	0.90
ENSG00000180185	*FAHD1*	PD	Wald ratio	1	-0.156	0.001212	0.86	0.78	0.94
ENSG00000101311	*FERMT1*	PD	Wald ratio	1	-0.373	0.00124	0.69	0.55	0.86
ENSG00000116783	*TNNI3K*	PD	Wald ratio	1	-0.266	0.001288	0.77	0.65	0.90
ENSG00000120910	*PPP3CC*	PD	Wald ratio	1	0.563	0.001295	1.76	1.25	2.47
ENSG00000162971	*TYW5*	PD	Wald ratio	1	-0.288	0.001308	0.75	0.63	0.89
ENSG00000239556	*#N/A*	PD	Wald ratio	1	-0.361	0.001371	0.70	0.56	0.87
ENSG00000085788	*DDHD2*	PD	Wald ratio	1	0.357	0.001379	1.43	1.15	1.78
ENSG00000120913	*PDLIM2*	PD	Wald ratio	1	-0.329	0.00145	0.72	0.59	0.88
ENSG00000253200	*#N/A*	PD	Wald ratio	1	-0.321	0.001453	0.73	0.60	0.88
ENSG00000089041	*P2RX7*	PD	Inverse variance weighted	2	0.405	0.001472	1.50	1.17	1.92
ENSG00000258708	*SLC25A21-AS1*	PD	Wald ratio	1	0.373	0.001542	1.45	1.15	1.83
ENSG00000155252	*PI4K2A*	PD	Wald ratio	1	0.401	0.001554	1.49	1.16	1.92
ENSG00000271755	*#N/A*	PD	Wald ratio	1	0.276	0.001655	1.32	1.11	1.57
ENSG00000199545	*RNA5SP195*	PD	Wald ratio	1	-2.577	0.001663	0.08	0.02	0.38
ENSG00000172346	*CSDC2*	PD	Wald ratio	1	-0.239	0.001712	0.79	0.68	0.91
ENSG00000236430	*#N/A*	PD	Wald ratio	1	0.418	0.001745	1.52	1.17	1.97
ENSG00000184110	*EIF3C*	PD	Wald ratio	1	-0.161	0.001954	0.85	0.77	0.94
ENSG00000265242	*#N/A*	PD	Wald ratio	1	-0.459	0.001977	0.63	0.47	0.85
ENSG00000237096	*#N/A*	PD	Wald ratio	1	0.409	0.00205	1.50	1.16	1.95
ENSG00000087157	*PGS1*	PD	Wald ratio	1	0.350	0.002165	1.42	1.13	1.77
ENSG00000026297	*RNASET2*	PD	Wald ratio	1	0.485	0.002236	1.62	1.19	2.22
ENSG00000178922	*HYI*	PD	Wald ratio	1	-0.228	0.002258	0.80	0.69	0.92
ENSG00000085491	*SLC25A24*	PD	Wald ratio	1	-0.167	0.002263	0.85	0.76	0.94
ENSG00000143819	*EPHX1*	PD	Wald ratio	1	-0.800	0.002356	0.45	0.27	0.75
ENSG00000118407	*FILIP1*	PD	Wald ratio	1	-0.714	0.002441	0.49	0.31	0.78
ENSG00000168077	*SCARA3*	PD	Wald ratio	1	-0.675	0.002566	0.51	0.33	0.79
ENSG00000035499	*DEPDC1B*	PD	Wald ratio	1	-0.389	0.0026	0.68	0.53	0.87
ENSG00000063854	*HAGH*	PD	Wald ratio	1	-0.409	0.002608	0.66	0.51	0.87
ENSG00000125449	*ARMC7*	PD	Wald ratio	1	-0.893	0.002657	0.41	0.23	0.73
ENSG00000260879	*#N/A*	PD	Wald ratio	1	-0.378	0.002661	0.68	0.54	0.88
ENSG00000168350	*DEGS2*	PD	Wald ratio	1	-0.527	0.002772	0.59	0.42	0.83
ENSG00000105852	*PON3*	PD	Wald ratio	1	0.343	0.002861	1.41	1.12	1.77
ENSG00000167186	*COQ7*	PD	Inverse variance weighted	2	0.379	0.00287	1.46	1.14	1.87
ENSG00000162039	*MEIOB*	PD	Inverse variance weighted	2	0.143	0.002892	1.15	1.05	1.27
ENSG00000164070	*HSPA4L*	PD	Wald ratio	1	-0.933	0.003119	0.39	0.21	0.73

ENSG00000205609	*EIF3CL*	PD	Wald ratio	1	-0.226	0.003131	0.80	0.69	0.93
ENSG00000221933	*OR2A25*	PD	Wald ratio	1	-0.405	0.003146	0.67	0.51	0.87
ENSG00000112796	*ENPP5*	PD	Wald ratio	1	-0.362	0.003173	0.70	0.55	0.89
ENSG00000229402	*#N/A*	PD	Wald ratio	1	-0.527	0.003197	0.59	0.42	0.84
ENSG00000065054	*SLC9A3R2*	PD	Wald ratio	1	-0.334	0.003226	0.72	0.57	0.89
ENSG00000260793	*#N/A*	PD	Wald ratio	1	0.522	0.00323	1.69	1.19	2.39
ENSG00000172236	*TPSAB1*	PD	Wald ratio	1	-0.205	0.003302	0.81	0.71	0.93
ENSG00000251310	*#N/A*	PD	Wald ratio	1	-0.461	0.003322	0.63	0.46	0.86
ENSG00000213215	*OR2F1*	PD	Wald ratio	1	-0.342	0.003343	0.71	0.57	0.89
ENSG00000204869	*IGFL4*	PD	Wald ratio	1	0.306	0.003421	1.36	1.11	1.67
ENSG00000167778	*SPRYD3*	PD	Wald ratio	1	-0.787	0.003489	0.46	0.27	0.77
ENSG00000117280	*RAB29*	PD	Wald ratio	1	0.256	0.003521	1.29	1.09	1.54
ENSG00000182372	*CLN8*	PD	Wald ratio	1	0.340	0.003534	1.41	1.12	1.77
ENSG00000105339	*DENND3*	PD	Wald ratio	1	-0.677	0.003565	0.51	0.32	0.80
ENSG00000130768	*SMPDL3B*	PD	Wald ratio	1	0.299	0.003608	1.35	1.10	1.65
ENSG00000070061	*ELP1*	PD	Wald ratio	1	-0.418	0.003662	0.66	0.50	0.87
ENSG00000149311	*ATM*	PD	Wald ratio	1	0.954	0.003662	2.60	1.36	4.94
ENSG00000270005	*#N/A*	PD	Wald ratio	1	0.365	0.003691	1.44	1.13	1.84
ENSG00000271875	*#N/A*	PD	Wald ratio	1	-0.342	0.003732	0.71	0.56	0.89
ENSG00000249877	*#N/A*	PD	Wald ratio	1	-0.837	0.00385	0.43	0.25	0.76
ENSG00000185619	*PCGF3*	PD	Wald ratio	1	-0.360	0.00404	0.70	0.55	0.89
ENSG00000238084	*#N/A*	PD	Wald ratio	1	-0.196	0.004157	0.82	0.72	0.94
ENSG00000132530	*XAF1*	PD	Wald ratio	1	-0.446	0.004294	0.64	0.47	0.87
ENSG00000144792	*ZNF660*	PD	Wald ratio	1	-0.278	0.004345	0.76	0.63	0.92
ENSG00000110171	*TRIM3*	PD	Wald ratio	1	-0.650	0.004372	0.52	0.33	0.82
ENSG00000100299	*ARSA*	PD	Wald ratio	1	0.652	0.004395	1.92	1.23	3.01
ENSG00000271937	*#N/A*	PD	Wald ratio	1	-0.220	0.004455	0.80	0.69	0.93
ENSG00000128253	*RFPL2*	PD	Wald ratio	1	-0.191	0.004533	0.83	0.72	0.94
ENSG00000082898	*XPO1*	PD	Wald ratio	1	-0.860	0.004593	0.42	0.23	0.77
ENSG00000117020	*AKT3*	PD	Wald ratio	1	1.153	0.004593	3.17	1.43	7.03
ENSG00000213214	*ARHGEF35*	PD	Wald ratio	1	0.225	0.004607	1.25	1.07	1.46
ENSG00000221836	*OR2A5*	PD	Wald ratio	1	-0.157	0.004607	0.86	0.77	0.95
ENSG00000221938	*OR2A14*	PD	Wald ratio	1	-0.308	0.004607	0.74	0.59	0.91
ENSG00000221989	*OR2A2*	PD	Wald ratio	1	-0.188	0.004607	0.83	0.73	0.94
ENSG00000273234	*#N/A*	PD	Wald ratio	1	-0.146	0.004607	0.86	0.78	0.96
ENSG00000272368	*#N/A*	PD	Wald ratio	1	0.239	0.00462	1.27	1.08	1.50
ENSG00000197238	*H4C11*	PD	Wald ratio	1	-0.676	0.004864	0.51	0.32	0.81
ENSG00000138641	*HERC3*	PD	Wald ratio	1	0.314	0.004866	1.37	1.10	1.70
ENSG00000109576	*AADAT*	PD	Wald ratio	1	-0.281	0.004892	0.76	0.62	0.92
ENSG00000135541	*AHI1*	PD	Wald ratio	1	-0.526	0.004946	0.59	0.41	0.85
ENSG00000144460	*NYAP2*	PD	Wald ratio	1	0.781	0.004967	2.18	1.27	3.76
ENSG00000178467	*P4HTM*	PD	Wald ratio	1	0.435	0.005057	1.55	1.14	2.09
ENSG00000198380	*GFPT1*	PD	Wald ratio	1	-0.654	0.00511	0.52	0.33	0.82
ENSG00000197674	*#N/A*	PD	Wald ratio	1	0.393	0.00511	1.48	1.13	1.95

ENSG00000263488	*#N/A*	PD	Wald ratio	1	-0.466	0.005165	0.63	0.45	0.87
ENSG00000216316	*#N/A*	PD	Wald ratio	1	-0.415	0.005217	0.66	0.49	0.88
ENSG00000125388	*GRK4*	PD	Wald ratio	1	-0.333	0.005294	0.72	0.57	0.91
ENSG00000197635	*DPP4*	PD	Wald ratio	1	0.325	0.00535	1.38	1.10	1.74
ENSG00000177479	*ARIH2*	PD	Wald ratio	1	0.386	0.005611	1.47	1.12	1.93
ENSG00000087076	*HSD17B14*	PD	Wald ratio	1	-0.405	0.005646	0.67	0.50	0.89
ENSG00000255198	*SNHG9*	PD	Wald ratio	1	0.273	0.005681	1.31	1.08	1.60
ENSG00000267475	*#N/A*	PD	Wald ratio	1	-0.296	0.005708	0.74	0.60	0.92
ENSG00000101442	*ACTR5*	PD	Wald ratio	1	0.376	0.005719	1.46	1.12	1.90
ENSG00000203288	*TDRKH-AS1*	PD	Wald ratio	1	0.193	0.005774	1.21	1.06	1.39
ENSG00000273377	*OR2Q1P*	PD	Wald ratio	1	-0.335	0.005836	0.72	0.56	0.91
ENSG00000253982	*CLN8-AS1*	PD	Wald ratio	1	0.328	0.005857	1.39	1.10	1.75
ENSG00000206187	*LINC02346*	PD	Wald ratio	1	-0.575	0.00593	0.56	0.37	0.85
ENSG00000213626	*LBH*	PD	Wald ratio	1	0.852	0.00598	2.34	1.28	4.30
ENSG00000143157	*POGK*	PD	Wald ratio	1	-0.487	0.006028	0.61	0.43	0.87
ENSG00000132196	*HSD17B7*	PD	Wald ratio	1	-0.592	0.006044	0.55	0.36	0.84
ENSG00000100614	*PPM1A*	PD	Wald ratio	1	0.656	0.006059	1.93	1.21	3.08
ENSG00000157445	*CACNA2D3*	PD	Wald ratio	1	-0.771	0.006081	0.46	0.27	0.80
ENSG00000184009	*ACTG1*	PD	Wald ratio	1	-0.853	0.006163	0.43	0.23	0.78
ENSG00000164615	*CAMLG*	PD	Wald ratio	1	-0.119	0.006175	0.89	0.82	0.97
ENSG00000110218	*PANX1*	PD	Wald ratio	1	-0.404	0.006248	0.67	0.50	0.89
ENSG00000227593	*#N/A*	PD	Wald ratio	1	0.289	0.006248	1.34	1.09	1.64
ENSG00000174353	*TRIM74*	PD	Wald ratio	1	-0.498	0.006256	0.61	0.43	0.87
ENSG00000221553	*#N/A*	PD	Wald ratio	1	-0.767	0.006286	0.46	0.27	0.81
ENSG00000250091	*#N/A*	PD	Wald ratio	1	0.240	0.006327	1.27	1.07	1.51
ENSG00000270028	*#N/A*	PD	Wald ratio	1	0.227	0.006327	1.25	1.07	1.48
ENSG00000133835	*HSD17B4*	PD	Wald ratio	1	-0.473	0.006336	0.62	0.44	0.88
ENSG00000230316	*FEZF1-AS1*	PD	Wald ratio	1	-0.224	0.006349	0.80	0.68	0.94
ENSG00000124422	*USP22*	PD	Wald ratio	1	-0.496	0.006393	0.61	0.43	0.87
ENSG00000183122	*#N/A*	PD	Wald ratio	1	-0.173	0.00645	0.84	0.74	0.95
ENSG00000213672	*NCKIPSD*	PD	Wald ratio	1	0.291	0.006497	1.34	1.08	1.65
ENSG00000189129	*PLAC9*	PD	Wald ratio	1	-0.222	0.006533	0.80	0.68	0.94
ENSG00000101460	*MAP1LC3A*	PD	Wald ratio	1	0.283	0.006577	1.33	1.08	1.63
ENSG00000135636	*DYSF*	PD	Wald ratio	1	0.771	0.00677	2.16	1.24	3.78
ENSG00000224043	*#N/A*	PD	Wald ratio	1	0.286	0.006944	1.33	1.08	1.64
ENSG00000197774	*EME2*	PD	Wald ratio	1	-0.422	0.007005	0.66	0.48	0.89
ENSG00000228950	*#N/A*	PD	Wald ratio	1	-0.280	0.007044	0.76	0.62	0.93
ENSG00000100413	*POLR3H*	PD	Wald ratio	1	-0.206	0.007156	0.81	0.70	0.95
ENSG00000013288	*MAN2B2*	PD	Wald ratio	1	-0.453	0.007188	0.64	0.46	0.88
ENSG00000167588	*GPD1*	PD	Wald ratio	1	0.487	0.007191	1.63	1.14	2.32
ENSG00000152147	*GEMIN6*	PD	Wald ratio	1	-0.618	0.007458	0.54	0.34	0.85
ENSG00000261701	*HPR*	PD	Inverse variance weighted	3	-0.063	0.00747	0.94	0.90	0.98

ENSG00000173960	*UBXN2A*	PD	Wald ratio	1	0.408	0.007503	1.50	1.12	2.03
ENSG00000204876	*LOC389602*	PD	Wald ratio	1	0.145	0.007694	1.16	1.04	1.29
ENSG00000172273	*HINFP*	PD	Wald ratio	1	-0.420	0.007881	0.66	0.48	0.90
ENSG00000187010	*RHD*	PD	Inverse variance weighted	2	-0.099	0.008149	0.91	0.84	0.97
ENSG00000131981	*LGALS3*	PD	Wald ratio	1	0.524	0.008161	1.69	1.15	2.49
ENSG00000221813	*OR6B1*	PD	Wald ratio	1	-0.241	0.008327	0.79	0.66	0.94
ENSG00000248810	*LINC02432*	PD	Wald ratio	1	-0.305	0.008504	0.74	0.59	0.93
ENSG00000259160	*#N/A*	PD	Wald ratio	1	-1.162	0.008548	0.31	0.13	0.74
ENSG00000204789	*#N/A*	PD	Wald ratio	1	-0.526	0.008574	0.59	0.40	0.87
ENSG00000171476	*HOPX*	PD	Wald ratio	1	1.224	0.008646	3.40	1.36	8.49
ENSG00000171067	*C11orf24*	PD	Wald ratio	1	-0.242	0.008733	0.79	0.66	0.94
ENSG00000120756	*PLS1*	PD	Wald ratio	1	0.306	0.008739	1.36	1.08	1.71
ENSG00000152404	*CWF19L2*	PD	Wald ratio	1	0.806	0.00876	2.24	1.23	4.09
ENSG00000271662	*#N/A*	PD	Wald ratio	1	-0.179	0.008978	0.84	0.73	0.96
ENSG00000183726	*TMEM50A*	PD	Wald ratio	1	0.332	0.009008	1.39	1.09	1.79
ENSG00000228014	*#N/A*	PD	Wald ratio	1	-0.170	0.009037	0.84	0.74	0.96
ENSG00000143776	*CDC42BPA*	PD	Wald ratio	1	0.595	0.009163	1.81	1.16	2.84
ENSG00000204169	*#N/A*	PD	Wald ratio	1	-0.200	0.00918	0.82	0.70	0.95
ENSG00000214194	*SMIM30*	PD	Wald ratio	1	-1.208	0.009605	0.30	0.12	0.75
ENSG00000272195	*#N/A*	PD	Wald ratio	1	-0.236	0.009618	0.79	0.66	0.94
ENSG00000167515	*TRAPPC2L*	PD	Wald ratio	1	-0.295	0.009639	0.74	0.60	0.93
ENSG00000049449	*RCN1*	PD	Wald ratio	1	0.352	0.009696	1.42	1.09	1.86
ENSG00000272511	*#N/A*	PD	Wald ratio	1	-0.320	0.009754	0.73	0.57	0.93
ENSG00000137054	*POLR1E*	PD	Wald ratio	1	0.281	0.009857	1.32	1.07	1.64
ENSG00000173699	*SPATA3*	PD	Wald ratio	1	-0.229	0.01006	0.80	0.67	0.95
ENSG00000227345	*PARG*	PD	Wald ratio	1	-0.590	0.01006	0.55	0.35	0.87
ENSG00000169629	*RGPD8*	PD	Wald ratio	1	0.203	0.010067	1.23	1.05	1.43
ENSG00000143437	*ARNT*	PD	Wald ratio	1	-0.263	0.010403	0.77	0.63	0.94
ENSG00000160216	*AGPAT3*	PD	Wald ratio	1	0.550	0.010478	1.73	1.14	2.64
ENSG00000109534	*GAR1*	PD	Wald ratio	1	0.307	0.010508	1.36	1.07	1.72
ENSG00000227258	*SMIM2-AS1*	PD	Wald ratio	1	-0.128	0.010602	0.88	0.80	0.97
ENSG00000259358	*#N/A*	PD	Wald ratio	1	-0.666	0.010602	0.51	0.31	0.86
ENSG00000172640	*OR10AD1*	PD	Wald ratio	1	-0.417	0.010678	0.66	0.48	0.91
ENSG00000139865	*TTC6*	PD	Wald ratio	1	-0.345	0.010766	0.71	0.54	0.92
ENSG00000144036	*EXOC6B*	PD	Wald ratio	1	-0.827	0.010828	0.44	0.23	0.83
ENSG00000235296	*#N/A*	PD	Wald ratio	1	-0.110	0.010872	0.90	0.82	0.97
ENSG00000231806	*#N/A*	PD	Wald ratio	1	0.079	0.010886	1.08	1.02	1.15
ENSG00000145214	*DGKQ*	PD	Wald ratio	1	0.259	0.01101	1.30	1.06	1.58
ENSG00000088833	*NSFL1C*	PD	Wald ratio	1	-0.389	0.011063	0.68	0.50	0.91
ENSG00000078304	*PPP2R5C*	PD	Wald ratio	1	0.405	0.011068	1.50	1.10	2.05
ENSG00000254835	*#N/A*	PD	Wald ratio	1	0.154	0.011178	1.17	1.04	1.31
ENSG00000156990	*RPUSD3*	PD	Wald ratio	1	0.250	0.011271	1.28	1.06	1.56

ENSG00000217377	*AK4P5*	PD	Inverse variance weighted	2	-0.477	0.011393	0.62	0.43	0.90
ENSG00000228755	*#N/A*	PD	Wald ratio	1	0.213	0.011451	1.24	1.05	1.46
ENSG00000119242	*CCDC92*	PD	Wald ratio	1	-1.033	0.011601	0.36	0.16	0.79
ENSG00000083544	*TDRD3*	PD	Wald ratio	1	-0.576	0.011609	0.56	0.36	0.88
ENSG00000175749	*EIF3KP1*	PD	Wald ratio	1	-0.189	0.011678	0.83	0.71	0.96
ENSG00000182544	*MFSD5*	PD	Wald ratio	1	-0.573	0.011678	0.56	0.36	0.88
ENSG00000117399	*CDC20*	PD	Wald ratio	1	-0.341	0.011754	0.71	0.55	0.93
ENSG00000143452	*HORMAD1*	PD	Wald ratio	1	-0.159	0.011927	0.85	0.75	0.97
ENSG00000155158	*TTC39B*	PD	Wald ratio	1	1.074	0.011932	2.93	1.27	6.76
ENSG00000266999	*#N/A*	PD	Wald ratio	1	-0.453	0.012055	0.64	0.45	0.91
ENSG00000184779	*#N/A*	PD	Wald ratio	1	-0.386	0.01232	0.68	0.50	0.92
ENSG00000141012	*GALNS*	PD	Wald ratio	1	0.469	0.012419	1.60	1.11	2.31
ENSG00000104863	*LIN7B*	PD	Wald ratio	1	-0.363	0.012513	0.70	0.52	0.92
ENSG00000215146	*LOC441666*	PD	Wald ratio	1	0.097	0.012575	1.10	1.02	1.19
ENSG00000111912	*NCOA7*	PD	Wald ratio	1	0.865	0.01258	2.38	1.20	4.69
ENSG00000188056	*TREML4*	PD	Wald ratio	1	-0.114	0.012591	0.89	0.82	0.98
ENSG00000214743	*#N/A*	PD	Wald ratio	1	-0.744	0.012648	0.48	0.26	0.85
ENSG00000261338	*#N/A*	PD	Wald ratio	1	0.070	0.012713	1.07	1.02	1.13
ENSG00000258144	*LINC02406*	PD	Wald ratio	1	0.197	0.012867	1.22	1.04	1.42
ENSG00000213973	*ZNF99*	PD	Wald ratio	1	-0.417	0.013124	0.66	0.47	0.92
ENSG00000125445	*MRPS7*	PD	Wald ratio	1	-0.369	0.013255	0.69	0.52	0.93
ENSG00000251450	*RASGRF2-AS1*	PD	Wald ratio	1	0.186	0.013279	1.20	1.04	1.39
ENSG00000161798	*AQP5*	PD	Wald ratio	1	0.264	0.013442	1.30	1.06	1.61
ENSG00000147912	*FBXO10*	PD	Wald ratio	1	0.462	0.013523	1.59	1.10	2.29
ENSG00000124207	*CSE1L*	PD	Wald ratio	1	0.522	0.013539	1.68	1.11	2.55
ENSG00000198153	*ZNF849P*	PD	Wald ratio	1	-0.215	0.013597	0.81	0.68	0.96
ENSG00000134248	*LAMTOR5*	PD	Wald ratio	1	-0.440	0.013638	0.64	0.45	0.91
ENSG00000180011	*ZADH2*	PD	Wald ratio	1	-0.358	0.013659	0.70	0.53	0.93
ENSG00000268038	*LINC01785*	PD	Wald ratio	1	-0.978	0.013841	0.38	0.17	0.82
ENSG00000163827	*LRRC2*	PD	Inverse variance weighted	2	-0.105	0.013889	0.90	0.83	0.98
ENSG00000012223	*LTF*	PD	Wald ratio	1	0.120	0.014086	1.13	1.02	1.24
ENSG00000074706	*IPCEF1*	PD	Wald ratio	1	0.747	0.014218	2.11	1.16	3.83
ENSG00000096063	*SRPK1*	PD	Wald ratio	1	-0.496	0.014228	0.61	0.41	0.91
ENSG00000132749	*TESMIN*	PD	Wald ratio	1	0.269	0.014286	1.31	1.06	1.62
ENSG00000142185	*TRPM2*	PD	Wald ratio	1	0.411	0.014447	1.51	1.09	2.10
ENSG00000084764	*MAPRE3*	PD	Wald ratio	1	0.975	0.014533	2.65	1.21	5.80
ENSG00000256989	*#N/A*	PD	Wald ratio	1	-0.424	0.014621	0.65	0.47	0.92
ENSG00000226856	*THORLNC*	PD	Wald ratio	1	0.208	0.014841	1.23	1.04	1.45
ENSG00000172687	*ZNF738*	PD	Wald ratio	1	0.148	0.014993	1.16	1.03	1.31

ENSG00000170017	*ALCAM*	PD	Wald ratio	1	1.203	0.015002	3.33	1.26	8.78
ENSG00000138246	*DNAJC13*	PD	Wald ratio	1	0.418	0.015084	1.52	1.08	2.13
ENSG00000169018	*FEM1B*	PD	Wald ratio	1	1.006	0.015097	2.73	1.21	6.15
ENSG00000260657	*#N/A*	PD	Wald ratio	1	-0.180	0.015097	0.84	0.72	0.97
ENSG00000197256	*KANK2*	PD	Wald ratio	1	-0.317	0.015128	0.73	0.56	0.94
ENSG00000177398	*UMODL1*	PD	Wald ratio	1	-0.244	0.015414	0.78	0.64	0.95
ENSG00000223443	*USP17L2*	PD	Wald ratio	1	0.567	0.01582	1.76	1.11	2.79
ENSG00000226430	*USP17L7*	PD	Wald ratio	1	0.590	0.01582	1.80	1.12	2.91
ENSG00000239775	*#N/A*	PD	Wald ratio	1	-0.282	0.015825	0.75	0.60	0.95
ENSG00000152760	*DYNLT5*	PD	Wald ratio	1	0.120	0.01596	1.13	1.02	1.24
ENSG00000167528	*ZNF641*	PD	Wald ratio	1	-0.374	0.01605	0.69	0.51	0.93
ENSG00000105656	*ELL*	PD	Wald ratio	1	0.238	0.016124	1.27	1.05	1.54
ENSG00000250189	*#N/A*	PD	Wald ratio	1	-0.510	0.016177	0.60	0.40	0.91
ENSG00000158856	*DMTN*	PD	Wald ratio	1	0.815	0.016337	2.26	1.16	4.39
ENSG00000163565	*IFI16*	PD	Wald ratio	1	0.386	0.016509	1.47	1.07	2.02
ENSG00000130818	*ZNF426*	PD	Wald ratio	1	-0.620	0.01662	0.54	0.32	0.89
ENSG00000170837	*GPR27*	PD	Wald ratio	1	-0.356	0.016665	0.70	0.52	0.94
ENSG00000163938	*GNL3*	PD	Wald ratio	1	-0.478	0.016718	0.62	0.42	0.92
ENSG00000125629	*INSIG2*	PD	Wald ratio	1	0.540	0.01677	1.72	1.10	2.67
ENSG00000054282	*SDCCAG8*	PD	Wald ratio	1	0.706	0.01677	2.03	1.14	3.62
ENSG00000215032	*GNL3LP1*	PD	Wald ratio	1	-0.247	0.016789	0.78	0.64	0.96
ENSG00000125450	*NUP85*	PD	Wald ratio	1	0.228	0.016961	1.26	1.04	1.52
ENSG00000163082	*SGPP2*	PD	Wald ratio	1	0.254	0.017173	1.29	1.05	1.59
ENSG00000272143	*FGF14-AS2*	PD	Wald ratio	1	-0.314	0.017181	0.73	0.56	0.95
ENSG00000146005	*PSD2*	PD	Wald ratio	1	-0.829	0.017218	0.44	0.22	0.86
ENSG00000100583	*SAMD15*	PD	Wald ratio	1	-0.274	0.017374	0.76	0.61	0.95
ENSG00000259162	*#N/A*	PD	Wald ratio	1	0.980	0.017661	2.66	1.19	5.98
ENSG00000164199	*ADGRV1*	PD	Wald ratio	1	-0.616	0.017714	0.54	0.32	0.90
ENSG00000204524	*ZNF805*	PD	Wald ratio	1	-0.354	0.017853	0.70	0.52	0.94
ENSG00000146731	*CCT6A*	PD	Wald ratio	1	-0.635	0.017972	0.53	0.31	0.90
ENSG00000110427	*KIAA1549L*	PD	Wald ratio	1	-0.862	0.017976	0.42	0.21	0.86
ENSG00000167553	*TUBA1C*	PD	Wald ratio	1	0.167	0.017976	1.18	1.03	1.36
ENSG00000165272	*AQP3*	PD	Inverse variance weighted	2	-0.164	0.018627	0.85	0.74	0.97
ENSG00000161904	*LEMD2*	PD	Wald ratio	1	-0.481	0.018759	0.62	0.41	0.92
ENSG00000181039	*#N/A*	PD	Wald ratio	1	0.913	0.01885	2.49	1.16	5.34
ENSG00000197442	*MAP3K5*	PD	Wald ratio	1	0.836	0.019145	2.31	1.15	4.64
ENSG00000267454	*ZNF582-DT*	PD	Wald ratio	1	-0.227	0.01925	0.80	0.66	0.96
ENSG00000182768	*NGRN*	PD	Wald ratio	1	-0.792	0.019266	0.45	0.23	0.88
ENSG00000257075	*RPEP6*	PD	Wald ratio	1	0.644	0.019401	1.90	1.11	3.27
ENSG00000225764	*P3H2-AS1*	PD	Wald ratio	1	-0.234	0.019426	0.79	0.65	0.96
ENSG00000112357	*PEX7*	PD	Wald ratio	1	0.233	0.019428	1.26	1.04	1.53
ENSG00000008838	*MED24*	PD	Wald ratio	1	-0.289	0.019631	0.75	0.59	0.95

ENSG00000197753	*LHFPL5*	PD	Wald ratio	1	0.310	0.019631	1.36	1.05	1.77
ENSG00000269514	*#N/A*	PD	Wald ratio	1	-0.226	0.019631	0.80	0.66	0.96
ENSG00000224183	*#N/A*	PD	Inverse variance weighted	2	-0.097	0.01968	0.91	0.84	0.98
ENSG00000197915	*HRNR*	PD	Wald ratio	1	-0.226	0.0197	0.80	0.66	0.96
ENSG00000160588	*MPZL3*	PD	Wald ratio	1	-0.300	0.020086	0.74	0.58	0.95
ENSG00000258232	*#N/A*	PD	Wald ratio	1	-0.272	0.020108	0.76	0.61	0.96
ENSG00000119943	*PYROXD2*	PD	Wald ratio	1	0.172	0.02031	1.19	1.03	1.37
ENSG00000074266	*EED*	PD	Wald ratio	1	0.591	0.020351	1.81	1.10	2.97
ENSG00000152990	*ADGRA3*	PD	Wald ratio	1	0.370	0.020351	1.45	1.06	1.98
ENSG00000044459	*CNTLN*	PD	Wald ratio	1	-0.325	0.020439	0.72	0.55	0.95
ENSG00000180081	*#N/A*	PD	Wald ratio	1	-0.280	0.020549	0.76	0.60	0.96
ENSG00000076554	*TPD52*	PD	Wald ratio	1	0.578	0.020583	1.78	1.09	2.91
ENSG00000176236	*RPP38-DT*	PD	Inverse variance weighted	2	0.148	0.020879	1.16	1.02	1.31
ENSG00000258413	*#N/A*	PD	Wald ratio	1	0.086	0.020891	1.09	1.01	1.17
ENSG00000042286	*AIFM2*	PD	Wald ratio	1	0.137	0.021216	1.15	1.02	1.29
ENSG00000235241	*#N/A*	PD	Wald ratio	1	-0.585	0.021538	0.56	0.34	0.92
ENSG00000179562	*GCC1*	PD	Wald ratio	1	0.293	0.021563	1.34	1.04	1.72
ENSG00000181924	*COA4*	PD	Wald ratio	1	-0.488	0.021623	0.61	0.41	0.93
ENSG00000183605	*SFXN4*	PD	Wald ratio	1	0.268	0.021678	1.31	1.04	1.64
ENSG00000230487	*PSMG3-AS1*	PD	Wald ratio	1	0.405	0.021723	1.50	1.06	2.12
ENSG00000270948	*MTDHP1*	PD	Wald ratio	1	0.131	0.021745	1.14	1.02	1.28
ENSG00000075673	*ATP12A*	PD	Inverse variance weighted	2	0.195	0.021843	1.22	1.03	1.44
ENSG00000148384	*INPP5E*	PD	Wald ratio	1	-0.340	0.021925	0.71	0.53	0.95
ENSG00000166796	*LDHC*	PD	Wald ratio	1	0.046	0.022073	1.05	1.01	1.09
ENSG00000165949	*IFI27*	PD	Wald ratio	1	0.312	0.022121	1.37	1.05	1.79
ENSG00000259030	*FPGT-TNNI3K*	PD	Wald ratio	1	-0.209	0.022168	0.81	0.68	0.97
ENSG00000118194	*TNNT2*	PD	Wald ratio	1	0.381	0.022179	1.46	1.06	2.03
ENSG00000258017	*LOC105369760*	PD	Wald ratio	1	-0.291	0.022316	0.75	0.58	0.96
ENSG00000140450	*ARRDC4*	PD	Wald ratio	1	0.343	0.022416	1.41	1.05	1.89
ENSG00000242094	*#N/A*	PD	Wald ratio	1	-0.484	0.022472	0.62	0.41	0.93
ENSG00000113249	*HAVCR1*	PD	Wald ratio	1	0.199	0.022576	1.22	1.03	1.45
ENSG00000145850	*TIMD4*	PD	Wald ratio	1	0.259	0.022576	1.30	1.04	1.62
ENSG00000158486	*DNAH3*	PD	Wald ratio	1	-0.193	0.022783	0.82	0.70	0.97
ENSG00000165434	*PGM2L1*	PD	Wald ratio	1	-0.571	0.022804	0.56	0.35	0.92
ENSG00000163466	*ARPC2*	PD	Wald ratio	1	0.414	0.022865	1.51	1.06	2.16
ENSG00000272782	*#N/A*	PD	Wald ratio	1	0.122	0.023099	1.13	1.02	1.26

ENSG00000248712	*CCDC153*	PD	Wald ratio	1	0.215	0.023201	1.24	1.03	1.49
ENSG00000088298	*EDEM2*	PD	Wald ratio	1	-0.219	0.023208	0.80	0.67	0.97
ENSG00000133789	*SWAP70*	PD	Wald ratio	1	-0.218	0.023353	0.80	0.67	0.97
ENSG00000136143	*SUCLA2*	PD	Wald ratio	1	-0.639	0.023503	0.53	0.30	0.92
ENSG00000213462	*ERV3-1*	PD	Wald ratio	1	-0.077	0.023552	0.93	0.87	0.99
ENSG00000113734	*BNIP1*	PD	Wald ratio	1	0.212	0.023735	1.24	1.03	1.49
ENSG00000145358	*DDIT4L*	PD	Inverse variance weighted	2	0.209	0.023831	1.23	1.03	1.48
ENSG00000185963	*BICD2*	PD	Wald ratio	1	-0.240	0.024007	0.79	0.64	0.97
ENSG00000140284	*SLC27A2*	PD	Wald ratio	1	-0.261	0.024075	0.77	0.61	0.97
ENSG00000010072	*SPRTN*	PD	Wald ratio	1	-0.264	0.024091	0.77	0.61	0.97
ENSG00000231326	*LINC02662*	PD	Wald ratio	1	-0.906	0.024249	0.40	0.18	0.89
ENSG00000269558	*#N/A*	PD	Wald ratio	1	-0.304	0.024449	0.74	0.57	0.96
ENSG00000233058	*#N/A*	PD	Wald ratio	1	0.316	0.024516	1.37	1.04	1.81
ENSG00000162365	*CYP4A22*	PD	Wald ratio	1	-0.268	0.02454	0.76	0.61	0.97
ENSG00000119457	*SLC46A2*	PD	Wald ratio	1	0.160	0.024623	1.17	1.02	1.35
ENSG00000242539	*#N/A*	PD	Wald ratio	1	-0.618	0.02511	0.54	0.31	0.93
ENSG00000251320	*#N/A*	PD	Wald ratio	1	-0.441	0.025174	0.64	0.44	0.95
ENSG00000196152	*ZNF79*	PD	Wald ratio	1	-0.204	0.025307	0.82	0.68	0.98
ENSG00000152137	*HSPB8*	PD	Wald ratio	1	-0.936	0.025315	0.39	0.17	0.89
ENSG00000175662	*TOM1L2*	PD	Wald ratio	1	0.319	0.025376	1.38	1.04	1.82
ENSG00000264880	*#N/A*	PD	Wald ratio	1	-0.163	0.025388	0.85	0.74	0.98
ENSG00000218510	*LINC00339*	PD	Wald ratio	1	0.061	0.025432	1.06	1.01	1.12
ENSG00000254485	*#N/A*	PD	Wald ratio	1	-0.191	0.025717	0.83	0.70	0.98
ENSG00000178149	*DALRD3*	PD	Wald ratio	1	0.462	0.025747	1.59	1.06	2.38
ENSG00000205632	*LINC01310*	PD	Wald ratio	1	0.269	0.025857	1.31	1.03	1.66
ENSG00000182963	*GJC1*	PD	Wald ratio	1	-0.192	0.025913	0.83	0.70	0.98
ENSG00000174945	*AMZ1*	PD	Wald ratio	1	0.336	0.025966	1.40	1.04	1.88
ENSG00000100024	*UPB1*	PD	Wald ratio	1	-0.274	0.026015	0.76	0.60	0.97
ENSG00000272983	*#N/A*	PD	Inverse variance weighted	2	0.203	0.026299	1.22	1.02	1.46
ENSG00000163145	*C1QTNF7*	PD	Wald ratio	1	-0.163	0.026307	0.85	0.74	0.98
ENSG00000259984	*#N/A*	PD	Wald ratio	1	0.168	0.026341	1.18	1.02	1.37
ENSG00000106153	*CHCHD2*	PD	Inverse variance weighted	2	0.183	0.026419	1.20	1.02	1.41
ENSG00000099785	*MARCHF2*	PD	Wald ratio	1	-0.224	0.026884	0.80	0.66	0.97
ENSG00000117868	*ESYT2*	PD	Wald ratio	1	-0.491	0.026999	0.61	0.40	0.95
ENSG00000188672	*RHCE*	PD	Wald ratio	1	0.085	0.027006	1.09	1.01	1.17
ENSG00000196381	*ZNF781*	PD	Wald ratio	1	-0.082	0.027172	0.92	0.86	0.99
ENSG00000223496	*EXOSC6*	PD	Wald ratio	1	0.138	0.027238	1.15	1.02	1.30
ENSG00000175164	*ABO*	PD	Wald ratio	1	-0.178	0.027309	0.84	0.71	0.98

ENSG00000177981	*ASB8*	PD	Wald ratio	1	-0.122	0.027321	0.89	0.79	0.99
ENSG00000178252	*WDR6*	PD	Wald ratio	1	-0.216	0.027335	0.81	0.67	0.98
ENSG00000233232	*#N/A*	PD	Wald ratio	1	0.107	0.027335	1.11	1.01	1.22
ENSG00000269918	*#N/A*	PD	Wald ratio	1	-0.225	0.027493	0.80	0.65	0.98
ENSG00000109762	*SNX25*	PD	Wald ratio	1	-0.715	0.027874	0.49	0.26	0.93
ENSG00000165934	*CPSF2*	PD	Wald ratio	1	0.480	0.027948	1.62	1.05	2.48
ENSG00000248925	*#N/A*	PD	Wald ratio	1	-0.162	0.028117	0.85	0.74	0.98
ENSG00000126861	*OMG*	PD	Wald ratio	1	-0.487	0.028188	0.61	0.40	0.95
ENSG00000263327	*TAPT1-AS1*	PD	Wald ratio	1	-0.393	0.02823	0.67	0.47	0.96
ENSG00000124228	*DDX27*	PD	Wald ratio	1	-0.459	0.028657	0.63	0.42	0.95
ENSG00000089195	*TRMT6*	PD	Wald ratio	1	0.591	0.028672	1.81	1.06	3.07
ENSG00000151689	*INPP1*	PD	Wald ratio	1	0.190	0.028796	1.21	1.02	1.43
ENSG00000165863	*C10orf82*	PD	Wald ratio	1	0.252	0.028965	1.29	1.03	1.61
ENSG00000148798	*INA*	PD	Wald ratio	1	0.334	0.029046	1.40	1.03	1.88
ENSG00000249898	*#N/A*	PD	Inverse variance weighted	2	0.123	0.029135	1.13	1.01	1.26
ENSG00000095383	*TBC1D2*	PD	Wald ratio	1	0.189	0.029257	1.21	1.02	1.43
ENSG00000090861	*AARS1*	PD	Wald ratio	1	-0.488	0.029349	0.61	0.40	0.95
ENSG00000270424	*PAWRP2*	PD	Wald ratio	1	-0.179	0.029349	0.84	0.71	0.98
ENSG00000168301	*KCTD6*	PD	Wald ratio	1	-0.369	0.02962	0.69	0.50	0.96
ENSG00000186148	*LOC440895*	PD	Wald ratio	1	0.177	0.029982	1.19	1.02	1.40
ENSG00000034239	*EFCAB1*	PD	Wald ratio	1	0.081	0.029984	1.08	1.01	1.17
ENSG00000145725	*PPIP5K2*	PD	Wald ratio	1	0.759	0.030059	2.14	1.08	4.24
ENSG00000156299	*TIAM1*	PD	Wald ratio	1	-0.453	0.030102	0.64	0.42	0.96
ENSG00000273117	*#N/A*	PD	Wald ratio	1	-0.440	0.030184	0.64	0.43	0.96
ENSG00000226200	*#N/A*	PD	Wald ratio	1	0.192	0.030204	1.21	1.02	1.44
ENSG00000185290	*NUPR2*	PD	Wald ratio	1	0.157	0.03026	1.17	1.02	1.35
ENSG00000153574	*RPIA*	PD	Wald ratio	1	0.350	0.030326	1.42	1.03	1.95
ENSG00000259114	*#N/A*	PD	Wald ratio	1	0.226	0.03041	1.25	1.02	1.54
ENSG00000116031	*CD207*	PD	Wald ratio	1	-0.264	0.030513	0.77	0.60	0.98
ENSG00000152672	*CLEC4F*	PD	Wald ratio	1	-0.212	0.030513	0.81	0.67	0.98
ENSG00000258881	*#N/A*	PD	Wald ratio	1	-0.262	0.030513	0.77	0.61	0.98
ENSG00000136010	*ALDH1L2*	PD	Wald ratio	1	-0.348	0.030662	0.71	0.52	0.97
ENSG00000138640	*FAM13A*	PD	Wald ratio	1	-0.487	0.03104	0.61	0.39	0.96
ENSG00000261070	*LOC338694*	PD	Wald ratio	1	0.181	0.03108	1.20	1.02	1.41
ENSG00000178980	*SELENOW*	PD	Wald ratio	1	0.636	0.031113	1.89	1.06	3.37
ENSG00000121570	*DPPA4*	PD	Wald ratio	1	-0.176	0.031252	0.84	0.71	0.98
ENSG00000116857	*TMEM9*	PD	Wald ratio	1	0.305	0.031318	1.36	1.03	1.79
ENSG00000185880	*TRIM69*	PD	Wald ratio	1	0.094	0.031384	1.10	1.01	1.20
ENSG00000235076	*GAPDHP52*	PD	Wald ratio	1	-0.230	0.031487	0.79	0.64	0.98
ENSG00000160957	*RECQL4*	PD	Wald ratio	1	0.267	0.031601	1.31	1.02	1.67
ENSG00000037042	*TUBG2*	PD	Wald ratio	1	0.447	0.03165	1.56	1.04	2.35
ENSG00000103037	*SETD6*	PD	Wald ratio	1	0.217	0.031825	1.24	1.02	1.51

ENSG00000114544	*SLC41A3*	PD	Wald ratio	1	0.492	0.031832	1.63	1.04	2.56
ENSG00000156110	*ADK*	PD	Wald ratio	1	-0.427	0.031864	0.65	0.44	0.96
ENSG00000244215	*LINC02016*	PD	Wald ratio	1	-0.254	0.031886	0.78	0.61	0.98
ENSG00000114120	*SLC25A36*	PD	Wald ratio	1	-0.907	0.031902	0.40	0.18	0.92
ENSG00000271780	*#N/A*	PD	Wald ratio	1	-0.104	0.031989	0.90	0.82	0.99
ENSG00000272444	*#N/A*	PD	Wald ratio	1	-0.355	0.031989	0.70	0.51	0.97
ENSG00000214189	*#N/A*	PD	Wald ratio	1	-0.233	0.032479	0.79	0.64	0.98
ENSG00000128829	*EIF2AK4*	PD	Wald ratio	1	-0.339	0.0327	0.71	0.52	0.97
ENSG00000187553	*CYP26C1*	PD	Wald ratio	1	-0.144	0.032739	0.87	0.76	0.99
ENSG00000234585	*CCT6P3*	PD	Wald ratio	1	-0.058	0.032836	0.94	0.89	1.00
ENSG00000251379	*#N/A*	PD	Wald ratio	1	-0.144	0.032841	0.87	0.76	0.99
ENSG00000019505	*SYT13*	PD	Wald ratio	1	-0.641	0.032849	0.53	0.29	0.95
ENSG00000077942	*FBLN1*	PD	Wald ratio	1	0.380	0.032938	1.46	1.03	2.07
ENSG00000130592	*LSP1*	PD	Wald ratio	1	0.245	0.033022	1.28	1.02	1.60
ENSG00000114520	*SNX4*	PD	Wald ratio	1	0.493	0.033031	1.64	1.04	2.58
ENSG00000244234	*GMCL2*	PD	Wald ratio	1	0.284	0.033123	1.33	1.02	1.72
ENSG00000261310	*#N/A*	PD	Wald ratio	1	-0.542	0.033605	0.58	0.35	0.96
ENSG00000050426	*LETMD1*	PD	Wald ratio	1	0.221	0.03367	1.25	1.02	1.53
ENSG00000043462	*LCP2*	PD	Wald ratio	1	-0.280	0.03377	0.76	0.58	0.98
ENSG00000213542	*RPL7AP43*	PD	Wald ratio	1	0.179	0.033853	1.20	1.01	1.41
ENSG00000085871	*MGST2*	PD	Wald ratio	1	0.475	0.03389	1.61	1.04	2.49
ENSG00000187699	*C2orf88*	PD	Wald ratio	1	-0.445	0.033904	0.64	0.42	0.97
ENSG00000260361	*#N/A*	PD	Wald ratio	1	-0.303	0.033975	0.74	0.56	0.98
ENSG00000243414	*TICAM2*	PD	Wald ratio	1	-0.255	0.033988	0.77	0.61	0.98
ENSG00000066084	*DIP2B*	PD	Wald ratio	1	0.748	0.034064	2.11	1.06	4.22
ENSG00000196878	*LAMB3*	PD	Wald ratio	1	-0.244	0.034072	0.78	0.62	0.98
ENSG00000135424	*ITGA7*	PD	Wald ratio	1	0.273	0.034146	1.31	1.02	1.69
ENSG00000178445	*GLDC*	PD	Wald ratio	1	-0.319	0.034163	0.73	0.54	0.98
ENSG00000255529	*POLR2M*	PD	Wald ratio	1	-0.343	0.034267	0.71	0.52	0.97
ENSG00000270607	*#N/A*	PD	Wald ratio	1	-0.626	0.034294	0.53	0.30	0.95
ENSG00000167332	*OR51E2*	PD	Wald ratio	1	0.093	0.034583	1.10	1.01	1.20
ENSG00000161048	*NAPEPLD*	PD	Wald ratio	1	-0.479	0.034718	0.62	0.40	0.97
ENSG00000174600	*CMKLR1*	PD	Wald ratio	1	-0.281	0.034763	0.75	0.58	0.98
ENSG00000239332	*LINC01119*	PD	Wald ratio	1	-0.267	0.034847	0.77	0.60	0.98
ENSG00000108510	*MED13*	PD	Wald ratio	1	0.735	0.034871	2.08	1.05	4.12
ENSG00000147650	*LRP12*	PD	Wald ratio	1	0.556	0.035129	1.74	1.04	2.93
ENSG00000251532	*#N/A*	PD	Wald ratio	1	-0.204	0.035185	0.82	0.67	0.99
ENSG00000224097	*LOC401127*	PD	Wald ratio	1	0.172	0.035344	1.19	1.01	1.39
ENSG00000248019	*FAM13A-AS1*	PD	Wald ratio	1	-0.715	0.03548	0.49	0.25	0.95
ENSG00000163171	*CDC42EP3*	PD	Wald ratio	1	-0.528	0.035529	0.59	0.36	0.96
ENSG00000175324	*LSM1*	PD	Wald ratio	1	-0.373	0.03564	0.69	0.49	0.98
ENSG00000178965	*ERICH3*	PD	Wald ratio	1	0.468	0.03568	1.60	1.03	2.47
ENSG00000198467	*TPM2*	PD	Wald ratio	1	-0.173	0.035729	0.84	0.72	0.99
ENSG00000101546	*RBFA*	PD	Wald ratio	1	0.239	0.035891	1.27	1.02	1.59

ENSG00000140937	*CDH11*	PD	Wald ratio	1	0.930	0.036	2.53	1.06	6.04
ENSG00000207116	*RNU6-31P*	PD	Wald ratio	1	-1.300	0.03638	0.27	0.08	0.92
ENSG00000261758	*#N/A*	PD	Wald ratio	1	0.271	0.036418	1.31	1.02	1.69
ENSG00000247903	*#N/A*	PD	Wald ratio	1	0.314	0.036514	1.37	1.02	1.84
ENSG00000172469	*MANEA*	PD	Wald ratio	1	0.348	0.036536	1.42	1.02	1.96
ENSG00000204520	*MICA*	PD	Wald ratio	1	-0.155	0.036567	0.86	0.74	0.99
ENSG00000234745	*HLA-B*	PD	Wald ratio	1	-0.233	0.036567	0.79	0.64	0.99
ENSG00000272927	*#N/A*	PD	Wald ratio	1	-0.201	0.036595	0.82	0.68	0.99
ENSG00000087074	*PPP1R15A*	PD	Wald ratio	1	-0.331	0.036838	0.72	0.53	0.98
ENSG00000268081	*#N/A*	PD	Wald ratio	1	-0.083	0.036995	0.92	0.85	0.99
ENSG00000107745	*MICU1*	PD	Wald ratio	1	-0.399	0.037027	0.67	0.46	0.98
ENSG00000101391	*CDK5RAP1*	PD	Wald ratio	1	-0.305	0.037108	0.74	0.55	0.98
ENSG00000066056	*TIE1*	PD	Wald ratio	1	-0.247	0.037141	0.78	0.62	0.99
ENSG00000261349	*#N/A*	PD	Inverse variance weighted	2	-0.081	0.037224	0.92	0.85	1.00
ENSG00000132507	*EIF5A*	PD	Wald ratio	1	0.318	0.03731	1.37	1.02	1.85
ENSG00000214279	*SCART1*	PD	Wald ratio	1	0.126	0.037692	1.13	1.01	1.28
ENSG00000273133	*#N/A*	PD	Wald ratio	1	-0.153	0.037772	0.86	0.74	0.99
ENSG00000176381	*PRR18*	PD	Wald ratio	1	0.746	0.037943	2.11	1.04	4.27
ENSG00000257139	*#N/A*	PD	Wald ratio	1	0.134	0.038008	1.14	1.01	1.30
ENSG00000060982	*BCAT1*	PD	Wald ratio	1	0.349	0.038156	1.42	1.02	1.97
ENSG00000141564	*RPTOR*	PD	Wald ratio	1	0.480	0.038173	1.62	1.03	2.55
ENSG00000231633	*#N/A*	PD	Wald ratio	1	0.346	0.038289	1.41	1.02	1.96
ENSG00000173157	*ADAMTS20*	PD	Wald ratio	1	0.192	0.03845	1.21	1.01	1.45
ENSG00000236144	*TMEM147-AS1*	PD	Wald ratio	1	-0.220	0.038739	0.80	0.65	0.99
ENSG00000127220	*ABHD8*	PD	Wald ratio	1	0.630	0.038766	1.88	1.03	3.42
ENSG00000157470	*FAM81A*	PD	Wald ratio	1	0.495	0.038997	1.64	1.03	2.63
ENSG00000227544	*LOC100506725*	PD	Wald ratio	1	-0.865	0.03911	0.42	0.19	0.96
ENSG00000088256	*GNA11*	PD	Wald ratio	1	0.768	0.039242	2.16	1.04	4.48
ENSG00000164136	*IL15*	PD	Wald ratio	1	-0.373	0.039477	0.69	0.48	0.98
ENSG00000259150	*LINC00929*	PD	Wald ratio	1	0.306	0.039487	1.36	1.01	1.82
ENSG00000182512	*GLRX5*	PD	Wald ratio	1	0.432	0.039511	1.54	1.02	2.32
ENSG00000258390	*LINC02318*	PD	Wald ratio	1	-0.218	0.039511	0.80	0.65	0.99
ENSG00000142959	*BEST4*	PD	Wald ratio	1	-0.326	0.039581	0.72	0.53	0.98
ENSG00000127530	*OR7C1*	PD	Wald ratio	1	0.174	0.039771	1.19	1.01	1.41
ENSG00000188269	*OR7A5*	PD	Wald ratio	1	0.120	0.039771	1.13	1.01	1.26
ENSG00000138942	*RNF185*	PD	Wald ratio	1	0.194	0.039953	1.21	1.01	1.46
ENSG00000178449	*COX14*	PD	Wald ratio	1	-0.385	0.039999	0.68	0.47	0.98
ENSG00000224560	*LOC100419154*	PD	Wald ratio	1	-0.173	0.040221	0.84	0.71	0.99
ENSG00000056050	*HPF1*	PD	Wald ratio	1	0.206	0.040494	1.23	1.01	1.50

ENSG00000266379	*#N/A*	PD	Wald ratio	1	0.283	0.040656	1.33	1.01	1.74
ENSG00000065268	*WDR18*	PD	Wald ratio	1	0.130	0.040732	1.14	1.01	1.29
ENSG00000235421	*#N/A*	PD	Inverse variance weighted	2	-0.107	0.040821	0.90	0.81	1.00
ENSG00000259687	*#N/A*	PD	Wald ratio	1	0.180	0.04086	1.20	1.01	1.42
ENSG00000233087	*RAB6D*	PD	Wald ratio	1	0.213	0.04098	1.24	1.01	1.52
ENSG00000124713	*GNMT*	PD	Wald ratio	1	-0.059	0.041257	0.94	0.89	1.00
ENSG00000183876	*ARSI*	PD	Wald ratio	1	-0.178	0.041302	0.84	0.71	0.99
ENSG00000245384	*CXXC4-AS1*	PD	Wald ratio	1	0.397	0.041315	1.49	1.02	2.18
ENSG00000249087	*ZNF436-AS1*	PD	Wald ratio	1	0.283	0.041435	1.33	1.01	1.74
ENSG00000198056	*PRIM1*	PD	Wald ratio	1	-0.211	0.041492	0.81	0.66	0.99
ENSG00000134086	*VHL*	PD	Wald ratio	1	-0.304	0.041564	0.74	0.55	0.99
ENSG00000197181	*PIWIL2*	PD	Wald ratio	1	0.083	0.041685	1.09	1.00	1.18
ENSG00000135063	*FAM189A2*	PD	Wald ratio	1	-0.561	0.041842	0.57	0.33	0.98
ENSG00000259658	*LOC100128108*	PD	Wald ratio	1	-0.149	0.042341	0.86	0.75	0.99
ENSG00000186638	*KIF24*	PD	Wald ratio	1	-0.166	0.042402	0.85	0.72	0.99
ENSG00000236255	*#N/A*	PD	Wald ratio	1	-0.145	0.042438	0.86	0.75	1.00
ENSG00000115486	*GGCX*	PD	Wald ratio	1	-0.141	0.042451	0.87	0.76	1.00
ENSG00000160223	*ICOSLG*	PD	Wald ratio	1	0.330	0.042854	1.39	1.01	1.92
ENSG00000165891	*E2F7*	PD	Wald ratio	1	0.178	0.042857	1.19	1.01	1.42
ENSG00000145715	*RASA1*	PD	Inverse variance weighted	2	-0.336	0.042917	0.71	0.52	0.99
ENSG00000123352	*SPATS2*	PD	Wald ratio	1	0.468	0.042947	1.60	1.01	2.51
ENSG00000197013	*ZNF429*	PD	Wald ratio	1	-0.273	0.043122	0.76	0.58	0.99
ENSG00000175279	*CENPS*	PD	Wald ratio	1	-0.483	0.043143	0.62	0.39	0.99
ENSG00000160539	*PLPP7*	PD	Wald ratio	1	-0.615	0.043352	0.54	0.30	0.98
ENSG00000197586	*ENTPD6*	PD	Wald ratio	1	0.717	0.04377	2.05	1.02	4.12
ENSG00000140326	*CDAN1*	PD	Wald ratio	1	-0.291	0.043935	0.75	0.56	0.99
ENSG00000196350	*ZNF729*	PD	Wald ratio	1	-0.281	0.044139	0.76	0.57	0.99
ENSG00000122642	*FKBP9*	PD	Wald ratio	1	0.298	0.044173	1.35	1.01	1.80
ENSG00000258548	*LINC00645*	PD	Wald ratio	1	0.151	0.04424	1.16	1.00	1.35
ENSG00000144908	*ALDH1L1*	PD	Wald ratio	1	0.474	0.044553	1.61	1.01	2.55
ENSG00000053900	*ANAPC4*	PD	Wald ratio	1	-0.268	0.04489	0.77	0.59	0.99
ENSG00000168792	*ABHD15*	PD	Wald ratio	1	0.204	0.044941	1.23	1.00	1.50
ENSG00000138074	*SLC5A6*	PD	Wald ratio	1	-0.349	0.044963	0.71	0.50	0.99
ENSG00000138085	*ATRAID*	PD	Wald ratio	1	0.355	0.044963	1.43	1.01	2.02
ENSG00000138604	*GLCE*	PD	Wald ratio	1	-0.514	0.0455	0.60	0.36	0.99
ENSG00000163820	*FYCO1*	PD	Wald ratio	1	-0.430	0.0455	0.65	0.43	0.99
ENSG00000265798	*#N/A*	PD	Wald ratio	1	0.159	0.045842	1.17	1.00	1.37
ENSG00000231133	*HAR1B*	PD	Wald ratio	1	-0.275	0.045905	0.76	0.58	1.00
ENSG00000175538	*KCNE3*	PD	Wald ratio	1	-0.179	0.04597	0.84	0.70	1.00

ENSG00000267160	*#N/A*	PD	Wald ratio	1	-0.452	0.046072	0.64	0.41	0.99
ENSG00000259917	*#N/A*	PD	Wald ratio	1	0.245	0.046105	1.28	1.00	1.63
ENSG00000167550	*RHEBL1*	PD	Wald ratio	1	0.349	0.046135	1.42	1.01	2.00
ENSG00000261790	*#N/A*	PD	Wald ratio	1	0.153	0.046439	1.17	1.00	1.36
ENSG00000226824	*#N/A*	PD	Wald ratio	1	0.062	0.046778	1.06	1.00	1.13
ENSG00000165406	*MARCHF8*	PD	Wald ratio	1	0.293	0.046782	1.34	1.00	1.79
ENSG00000271215	*#N/A*	PD	Wald ratio	1	-1.316	0.04679	0.27	0.07	0.98
ENSG00000101407	*TTI1*	PD	Wald ratio	1	-0.534	0.046935	0.59	0.35	0.99
ENSG00000114547	*ROPN1B*	PD	Wald ratio	1	-0.090	0.046966	0.91	0.84	1.00
ENSG00000198131	*ZNF544*	PD	Wald ratio	1	-0.188	0.046993	0.83	0.69	1.00
ENSG00000206192	*#N/A*	PD	Inverse variance weighted	2	0.159	0.047081	1.17	1.00	1.37
ENSG00000233297	*#N/A*	PD	Inverse variance weighted	2	0.093	0.04715	1.10	1.00	1.20
ENSG00000160285	*LSS*	PD	Wald ratio	1	-0.297	0.047181	0.74	0.55	1.00
ENSG00000133265	*HSPBP1*	PD	Wald ratio	1	-0.906	0.047205	0.40	0.17	0.99
ENSG00000172992	*DCAKD*	PD	Wald ratio	1	-0.148	0.047232	0.86	0.75	1.00
ENSG00000057663	*ATG5*	PD	Wald ratio	1	0.847	0.047516	2.33	1.01	5.39
ENSG00000168883	*USP39*	PD	Wald ratio	1	0.307	0.047822	1.36	1.00	1.84
ENSG00000100216	*TOMM22*	PD	Wald ratio	1	0.278	0.047873	1.32	1.00	1.74
ENSG00000143466	*#N/A*	PD	Wald ratio	1	-0.127	0.047988	0.88	0.78	1.00
ENSG00000162888	*#N/A*	PD	Wald ratio	1	-0.204	0.047988	0.82	0.67	1.00
ENSG00000151466	*SCLT1*	PD	Wald ratio	1	-0.701	0.048071	0.50	0.25	0.99
ENSG00000130764	*LRRC47*	PD	Wald ratio	1	0.620	0.048222	1.86	1.00	3.44
ENSG00000238286	*#N/A*	PD	Wald ratio	1	-0.114	0.048304	0.89	0.80	1.00
ENSG00000250158	*#N/A*	PD	Wald ratio	1	-0.248	0.048406	0.78	0.61	1.00
ENSG00000032742	*IFT88*	PD	Wald ratio	1	-0.189	0.048458	0.83	0.69	1.00
ENSG00000219545	*UMAD1*	PD	Wald ratio	1	0.450	0.048471	1.57	1.00	2.45
ENSG00000184277	*TM2D3*	PD	Wald ratio	1	0.242	0.048499	1.27	1.00	1.62
ENSG00000169251	*NMD3*	PD	Wald ratio	1	0.158	0.048515	1.17	1.00	1.37
ENSG00000161896	*IP6K3*	PD	Wald ratio	1	0.339	0.048555	1.40	1.00	1.96
ENSG00000241935	*HOGA1*	PD	Inverse variance weighted	2	0.160	0.048569	1.17	1.00	1.37
ENSG00000271918	*#N/A*	PD	Wald ratio	1	-0.245	0.048647	0.78	0.61	1.00
ENSG00000232053	*#N/A*	PD	Wald ratio	1	0.215	0.048789	1.24	1.00	1.54
ENSG00000218416	*LOC100130449*	PD	Wald ratio	1	0.376	0.048888	1.46	1.00	2.12
ENSG00000105404	*RABAC1*	PD	Wald ratio	1	-0.559	0.04901	0.57	0.33	1.00
ENSG00000149273	*RPS3*	PD	Wald ratio	1	0.294	0.049401	1.34	1.00	1.80
ENSG00000164049	*FBXW12*	PD	Wald ratio	1	-0.191	0.049472	0.83	0.68	1.00
ENSG00000213689	*TREX1*	PD	Wald ratio	1	0.096	0.049472	1.10	1.00	1.21

ENSG00000244380	*#N/A*	PD	Wald ratio	1	-0.164	0.049472	0.85	0.72	1.00
ENSG00000160460	*SPTBN4*	PD	Wald ratio	1	0.555	0.049583	1.74	1.00	3.03
ENSG00000161265	*U2AF1L4*	PD	Wald ratio	1	-0.275	0.04964	0.76	0.58	1.00
ENSG00000164039	*BDH2*	PD	Wald ratio	1	-0.275	0.049744	0.76	0.58	1.00
ENSG00000237684	*RPSAP35*	PD	Wald ratio	1	0.324	0.049835	1.38	1.00	1.91
ENSG00000247157	*LINC01252*	PD	Wald ratio	1	0.167	0.049891	1.18	1.00	1.40
ENSG00000256537	*SMIM10L1*	PD	Wald ratio	1	0.161	0.049891	1.18	1.00	1.38

Supplementary Table 3 The result of the MR analysis between CSF pQTLs and PD.

gene	outcome	method	nsnp	b	p-value	or	or_lci95	or_uci95
ENTPD1	PD	Wald ratio	1	3.27	3.16E-08	26.24	8.25	83.50
GPNMB	PD	Wald ratio	1	0.86	3.95E-07	2.37	1.70	3.31
CTSB	PD	Wald ratio	1	-1.66	2.19E-05	0.19	0.09	0.41
CD84	PD	Wald ratio	1	-2.93	2.23E-05	0.05	0.01	0.21
FCGR2B	PD	Wald ratio	1	0.30	6.63E-05	1.35	1.16	1.56
ARSA	PD	Wald ratio	1	1.14	0.001185	3.12	1.57	6.19
PGD	PD	Wald ratio	1	1.07	0.001217	2.92	1.53	5.59
ICAM1	PD	Wald ratio	1	-0.29	0.003739	0.75	0.62	0.91
COLEC11	PD	Wald ratio	1	-0.40	0.010088	0.67	0.50	0.91
ICOSLG	PD	Wald ratio	1	-1.11	0.012961	0.33	0.14	0.79
IL1R1	PD	Wald ratio	1	-1.28	0.015971	0.28	0.10	0.79
PCSK7	PD	Wald ratio	1	-1.05	0.018285	0.35	0.15	0.84
EPHA1	PD	Wald ratio	1	0.58	0.025929	1.79	1.07	2.99
PLG	PD	Wald ratio	1	1.10	0.028549	3.01	1.12	8.07
GHR	PD	Inverse variance weighted	2	0.74	0.028627	2.09	1.08	4.05
TIE1	PD	Wald ratio	1	-1.39	0.032611	0.25	0.07	0.89
ESD	PD	Wald ratio	1	-0.29	0.036075	0.75	0.57	0.98
CTSA	PD	Wald ratio	1	-1.21	0.045005	0.30	0.09	0.97

Supplementary Table 4 The result of the MR analysis between Blood pQTLs and PD

gene	outcome	method	nsnp	b	pval	or	or_lci95	or_uci95
SNCA	PD	Wald ratio	1	-0.87	6.26E-25	0.42	0.35	0.49
GPNMB	PD	Wald ratio	1	0.50	1.03E-07	1.64	1.37	1.97
FCGR2A	PD	Inverse variance weighted	3	0.06	1.76E-05	1.06	1.03	1.09
BAG3	PD	Wald ratio	1	0.46	2.37E-05	1.59	1.28	1.97
CTSB	PD	Wald ratio	1	-0.19	0.00019	0.83	0.75	0.91
ATF6	PD	Inverse variance weighted	4	0.14	0.000395	1.15	1.06	1.24
UAP1	PD	Wald ratio	1	0.42	0.001096	1.52	1.18	1.96
ENPP5	PD	Inverse variance weighted	2	-0.07	0.003071	0.94	0.90	0.98
EGF	PD	Inverse variance weighted	2	-0.14	0.00347	0.87	0.79	0.95
ICAM1	PD	Wald ratio	1	-0.04	0.003739	0.96	0.93	0.99
ASIP	PD	Wald ratio	1	-0.11	0.004238	0.89	0.83	0.97
SERPINE2	PD	Inverse variance weighted	2	-0.16	0.004707	0.85	0.76	0.95
IDUA	PD	Inverse variance weighted	2	-0.09	0.00476	0.92	0.86	0.97
HAVCR2	PD	Wald ratio	1	-0.09	0.004847	0.91	0.86	0.97
CCNH	PD	Wald ratio	1	0.23	0.00812	1.25	1.06	1.48
FARS2	PD	Wald ratio	1	-0.17	0.008422	0.84	0.74	0.96
EMILIN3	PD	Wald ratio	1	0.20	0.008924	1.22	1.05	1.42
TBXAS1	PD	Inverse variance weighted	2	0.28	0.009234	1.32	1.07	1.62
CREG1	PD	Inverse variance weighted	2	-0.16	0.010294	0.85	0.75	0.96
NTRK1	PD	Wald ratio	1	0.46	0.012179	1.58	1.10	2.26
B3GAT3	PD	Inverse variance weighted	2	0.10	0.012498	1.11	1.02	1.20
SURF1	PD	Inverse variance weighted	3	-0.11	0.012686	0.90	0.82	0.98
MBD4	PD	Wald ratio	1	0.43	0.014076	1.54	1.09	2.17
IL1B	PD	Inverse variance weighted	2	0.18	0.015933	1.20	1.03	1.39
RXFP1	PD	Wald ratio	1	-0.38	0.016837	0.68	0.50	0.93
FCGR2B	PD	Weighted median	3	0.05	0.017572	1.05	1.01	1.09
MGAT2	PD	Inverse variance weighted	2	-0.18	0.018373	0.83	0.71	0.97
CMPK1	PD	Inverse variance weighted	2	0.25	0.01839	1.28	1.04	1.58
RELL1	PD	Wald ratio	1	-0.37	0.018406	0.69	0.50	0.94
PAPPA2	PD	Wald ratio	1	-0.27	0.018976	0.76	0.61	0.96
EPHA10	PD	Wald ratio	1	0.09	0.019106	1.09	1.01	1.17
APOA5	PD	Wald ratio	1	-0.22	0.019416	0.81	0.67	0.97
LOC652493	PD	Wald ratio	1	0.25	0.019416	1.29	1.04	1.60
NXPH3	PD	Wald ratio	1	0.26	0.019416	1.30	1.04	1.62
BSG	PD	Wald ratio	1	0.36	0.020875	1.44	1.06	1.95
CRABP2	PD	Wald ratio	1	0.37	0.020875	1.44	1.06	1.97
FKBP14	PD	Wald ratio	1	0.25	0.020875	1.29	1.04	1.60
GDA	PD	Wald ratio	1	0.32	0.020875	1.37	1.05	1.80
IL19	PD	Wald ratio	1	0.09	0.020875	1.09	1.01	1.17
ISOC1	PD	Wald ratio	1	0.30	0.020875	1.35	1.05	1.74

NSDHL	PD	Wald ratio	1	0.18	0.020875	1.20	1.03	1.40
SF1	PD	Wald ratio	1	0.25	0.020875	1.29	1.04	1.60
SIGLEC14	PD	Wald ratio	1	0.15	0.020875	1.16	1.02	1.31
EFNA5	PD	Wald ratio	1	0.41	0.021098	1.51	1.06	2.14
CTSF	PD	Inverse variance weighted	3	-0.19	0.021581	0.83	0.70	0.97
GLCE	PD	Wald ratio	1	0.05	0.023594	1.06	1.01	1.11
FGFR3	PD	Inverse variance weighted	2	0.20	0.023981	1.22	1.03	1.44
GABBR2	PD	Wald ratio	1	-0.27	0.024934	0.76	0.60	0.97
STC2	PD	Wald ratio	1	0.29	0.026001	1.33	1.04	1.72
MFAP1	PD	Wald ratio	1	-0.35	0.02822	0.70	0.51	0.96
COLEC10	PD	Inverse variance weighted	2	-0.15	0.028228	0.86	0.76	0.98
NRXN3	PD	Inverse variance weighted	2	0.20	0.028724	1.22	1.02	1.45
KIAA1161	PD	Wald ratio	1	0.12	0.029123	1.13	1.01	1.27
CCL15	PD	Inverse variance weighted	2	-0.05	0.031046	0.95	0.91	1.00
PLAU	PD	Wald ratio	1	-0.22	0.031301	0.80	0.66	0.98
FAM171B	PD	Wald ratio	1	0.17	0.03247	1.19	1.01	1.39
PLG	PD	Inverse variance weighted	3	-0.13	0.034403	0.88	0.78	0.99
TCN2	PD	Inverse variance weighted	5	-0.04	0.036774	0.96	0.92	1.00
DNAJA4	PD	Wald ratio	1	0.29	0.03759	1.34	1.02	1.76
FAH	PD	Wald ratio	1	0.06	0.039853	1.07	1.00	1.13
ARL1	PD	Wald ratio	1	-0.24	0.040188	0.78	0.62	0.99
SEMA3G	PD	Inverse variance weighted	2	0.14	0.040396	1.15	1.01	1.31
GNMT	PD	Wald ratio	1	-0.09	0.041842	0.91	0.84	1.00
PARK2	PD	Wald ratio	1	-0.35	0.043418	0.71	0.50	0.99
PI3	PD	Wald ratio	1	-0.11	0.04601	0.89	0.80	1.00
RACGAP1	PD	Inverse variance weighted	3	0.16	0.046166	1.17	1.00	1.37
RBP4	PD	Wald ratio	1	0.24	0.046585	1.27	1.00	1.62
TXNDC5	PD	Inverse variance weighted	4	-0.08	0.048858	0.92	0.85	1.00
DHX8	PD	Inverse variance weighted	2	-0.05	0.049158	0.95	0.91	1.00
STIM1	PD	Inverse variance weighted	4	-0.10	0.049309	0.90	0.81	1.00
CBLN4	PD	Inverse variance weighted	3	-0.16	0.049768	0.86	0.73	1.00

Supplementary Table 5 The result of the MR analysis between blood eQTLs and PD.

exposure name	gene	outcome	method	nsnp	b	p-value	or	or_lci95	or_uci95
ENSG00000172465	TCEAL1	PD	Wald ratio	1	3.01	4.42E-21	20.24	10.83	37.84
ENSG00000266644	#N/A	PD	Wald ratio	1	1.35	8.59E-21	3.87	2.92	5.14
ENSG00000174547	MRPL11	PD	Wald ratio	1	2.84	8.95E-21	17.17	9.46	31.17
ENSG00000264070	#N/A	PD	Inverse variance weighted	2	-0.33	3.43E-17	0.72	0.66	0.77
ENSG00000225190	PLEKHM1	PD	Wald ratio	1	-0.73	2.65E-16	0.48	0.41	0.58
ENSG00000264057	#N/A	PD	Wald ratio	1	-0.79	1.37E-15	0.46	0.38	0.55
ENSG00000263503	#N/A	PD	Inverse variance weighted	3	-0.26	1.64E-10	0.77	0.72	0.84
ENSG00000214401	KANSL1-AS1	PD	Inverse variance weighted	4	-0.23	2.91E-10	0.80	0.74	0.85
ENSG00000214846	RPL10AP7	PD	Inverse variance weighted	2	-0.25	1.67E-09	0.78	0.72	0.85
ENSG00000167394	ZNF668	PD	Inverse variance weighted	2	0.23	3.52E-09	1.26	1.17	1.36
ENSG00000156928	MALSU1	PD	Wald ratio	1	1.03	6.50E-09	2.81	1.98	3.99
ENSG00000243368	#N/A	PD	Wald ratio	1	-1.54	1.77E-08	0.22	0.13	0.37
ENSG00000226816	#N/A	PD	Wald ratio	1	-0.22	3.86E-08	0.80	0.74	0.87
ENSG00000254918	#N/A	PD	Wald ratio	1	-0.70	4.84E-08	0.50	0.39	0.64
ENSG00000131470	PSMC3IP	PD	Inverse variance weighted	2	-0.54	1.07E-07	0.58	0.48	0.71
ENSG00000262539	#N/A	PD	Inverse variance weighted	2	-0.45	1.69E-07	0.64	0.54	0.75
ENSG00000262766	#N/A	PD	Wald ratio	1	-0.95	1.85E-07	0.39	0.27	0.55
ENSG00000230042	AK3P3	PD	Wald ratio	1	0.41	5.11E-07	1.50	1.28	1.76
ENSG00000214425	#N/A	PD	Inverse variance weighted	6	0.24	5.60E-07	1.27	1.16	1.40
ENSG00000049167	ERCC8	PD	Inverse variance weighted	2	0.24	6.10E-07	1.27	1.16	1.40
ENSG00000164182	NDUFAF2	PD	Wald ratio	1	0.52	7.23E-07	1.67	1.37	2.05
ENSG00000167397	VKORC1	PD	Wald ratio	1	0.58	9.39E-07	1.79	1.42	2.27
ENSG00000167395	ZNF646	PD	Wald ratio	1	-0.58	1.07E-06	0.56	0.45	0.71
ENSG00000267198	#N/A	PD	Wald ratio	1	0.28	1.62E-06	1.33	1.18	1.49
ENSG00000103507	BCKDK	PD	Inverse variance weighted	6	0.23	1.69E-06	1.25	1.14	1.37
ENSG00000225342	#N/A	PD	Wald ratio	1	-0.58	1.89E-06	0.56	0.44	0.71
ENSG00000073969	NSF	PD	Inverse variance weighted	3	-0.30	2.13E-06	0.74	0.65	0.84
ENSG00000127415	IDUA	PD	Inverse variance weighted	3	-0.37	2.27E-06	0.69	0.60	0.81
ENSG00000156858	PRR14	PD	Wald ratio	1	0.55	2.32E-06	1.74	1.38	2.18
ENSG00000188725	SMIM15	PD	Wald ratio	1	-1.43	2.40E-06	0.24	0.13	0.43
ENSG00000257410	#N/A	PD	Wald ratio	1	0.66	7.49E-06	1.94	1.45	2.59
ENSG00000260911	#N/A	PD	Inverse variance weighted	3	-0.24	9.17E-06	0.79	0.71	0.88
ENSG00000160766	GBAP1	PD	Inverse variance weighted	6	0.15	1.69E-05	1.16	1.08	1.24
ENSG00000163686	ABHD6	PD	Wald ratio	1	-0.37	1.93E-05	0.69	0.59	0.82
ENSG00000143740	SNAP47	PD	Inverse variance weighted	2	0.32	2.38E-05	1.38	1.19	1.60
ENSG00000260943	#N/A	PD	Inverse variance weighted	6	0.15	2.53E-05	1.16	1.08	1.24
ENSG00000261716	H2BC20P	PD	Inverse variance weighted	4	0.28	2.90E-05	1.32	1.16	1.50
ENSG00000115216	NRBP1	PD	Wald ratio	1	-0.20	3.32E-05	0.81	0.74	0.90
ENSG00000124613	ZNF391	PD	Wald ratio	1	0.20	3.98E-05	1.22	1.11	1.34

ENSG00000184348	#N/A	PD	Inverse variance weighted	2	0.67	4.11E-05	1.96	1.42	2.71
ENSG00000188987	#N/A	PD	Inverse variance weighted	2	0.48	4.11E-05	1.61	1.28	2.03
ENSG00000197697	#N/A	PD	Inverse variance weighted	2	0.43	4.11E-05	1.54	1.25	1.90
ENSG00000217275	RPS10P1	PD	Inverse variance weighted	2	0.54	4.11E-05	1.72	1.33	2.23
ENSG00000184678	H2BC21	PD	Inverse variance weighted	4	0.39	4.47E-05	1.47	1.22	1.77
ENSG00000224515	#N/A	PD	Wald ratio	1	0.61	4.73E-05	1.85	1.37	2.48
ENSG00000266305	MIR4518	PD	Wald ratio	1	1.10	5.08E-05	3.01	1.77	5.13
ENSG00000178226	PRSS36	PD	Wald ratio	1	-0.42	5.69E-05	0.66	0.53	0.81
ENSG00000141027	NCOR1	PD	Wald ratio	1	0.15	6.49E-05	1.17	1.08	1.26
ENSG00000096654	ZNF184	PD	Inverse variance weighted	2	-0.30	7.32E-05	0.74	0.64	0.86
ENSG00000180596	H2BC4	PD	Inverse variance weighted	4	0.24	7.47E-05	1.27	1.13	1.42
ENSG00000155438	NIFK	PD	Inverse variance weighted	3	-0.19	9.22E-05	0.83	0.75	0.91
ENSG00000177565	TBL1XR1	PD	Wald ratio	1	-0.30	0.000128	0.74	0.64	0.87
ENSG00000185022	MAFF	PD	Wald ratio	1	-0.78	0.000128	0.46	0.31	0.68
ENSG00000157992	KRTCAP3	PD	Wald ratio	1	0.17	0.000132	1.19	1.09	1.30
ENSG00000187837	H1-2	PD	Inverse variance weighted	6	0.13	0.000154	1.14	1.06	1.22
ENSG00000168216	LMBRD1	PD	Wald ratio	1	0.56	0.000159	1.76	1.31	2.36
ENSG00000138760	SCARB2	PD	Inverse variance weighted	4	0.26	0.000201	1.29	1.13	1.48
ENSG00000180901	KCTD2	PD	Inverse variance weighted	3	0.16	0.000229	1.17	1.08	1.28
ENSG00000107651	SEC23IP	PD	Wald ratio	1	0.56	0.00023	1.75	1.30	2.35
ENSG00000133627	ACTR3B	PD	Wald ratio	1	0.75	0.000242	2.12	1.42	3.17
ENSG00000174680	#N/A	PD	Wald ratio	1	0.56	0.000258	1.76	1.30	2.38
ENSG00000211803	TRAV23DV6	PD	Inverse variance weighted	2	0.33	0.000271	1.39	1.16	1.66
ENSG00000224805	LINC00853	PD	Inverse variance weighted	3	0.26	0.000294	1.30	1.13	1.49
ENSG00000205609	EIF3CL	PD	Inverse variance weighted	4	-0.21	0.000296	0.81	0.72	0.91
ENSG00000168291	PDHB	PD	Inverse variance weighted	3	-0.16	0.00031	0.85	0.78	0.93
ENSG00000108784	NAGLU	PD	Inverse variance weighted	2	0.20	0.000323	1.22	1.09	1.35
ENSG00000174946	GPR171	PD	Wald ratio	1	-0.71	0.000357	0.49	0.33	0.73
ENSG00000160714	UBE2Q1	PD	Inverse variance weighted	2	-0.33	0.000364	0.72	0.60	0.86
ENSG00000213693	SEC14L1P1	PD	Wald ratio	1	-0.99	0.000368	0.37	0.21	0.64
ENSG00000157540	DYRK1A	PD	Wald ratio	1	0.51	0.000404	1.66	1.25	2.20
ENSG00000109743	BST1	PD	Inverse variance weighted	6	-0.12	0.000411	0.88	0.82	0.95
ENSG00000173473	SMARCC1	PD	Wald ratio	1	0.92	0.000418	2.52	1.51	4.20
ENSG00000168763	CNNM3	PD	Inverse variance weighted	3	0.47	0.000425	1.60	1.23	2.09
ENSG00000173068	BNC2	PD	Inverse variance weighted	3	-0.13	0.000432	0.88	0.82	0.94
ENSG00000204650	LINC02210	PD	Inverse variance weighted	4	-0.14	0.000433	0.87	0.81	0.94
ENSG00000184110	EIF3C	PD	Inverse variance weighted	2	-0.27	0.00044	0.77	0.66	0.89
ENSG00000172974	#N/A	PD	Wald ratio	1	-1.14	0.000442	0.32	0.17	0.60
ENSG00000268201	#N/A	PD	Inverse variance weighted	2	-0.40	0.000467	0.67	0.54	0.84
ENSG00000099377	HSD3B7	PD	Inverse variance weighted	2	0.32	0.000491	1.37	1.15	1.64
ENSG00000053900	ANAPC4	PD	Inverse variance weighted	6	-0.13	0.000508	0.88	0.82	0.94
ENSG00000123600	METTL8	PD	Wald ratio	1	0.22	0.000526	1.24	1.10	1.41
ENSG00000130939	UBE4B	PD	Inverse variance weighted	3	0.23	0.000575	1.26	1.11	1.44

ENSG00000163519	TRAT1	PD	Inverse variance weighted	5	0.15	0.000586	1.17	1.07	1.27
ENSG00000139218	SCAF11	PD	Wald ratio	1	0.99	0.000595	2.70	1.53	4.75
ENSG00000249790	#N/A	PD	Inverse variance weighted	3	0.25	0.000602	1.28	1.11	1.48
ENSG00000179921	GPBAR1	PD	Inverse variance weighted	11	-0.11	0.000609	0.89	0.84	0.95
ENSG00000230068	#N/A	PD	Wald ratio	1	-0.99	0.000615	0.37	0.21	0.66
ENSG00000165863	C10orf82	PD	Wald ratio	1	1.04	0.000633	2.82	1.56	5.11
ENSG00000172375	C2CD2L	PD	Inverse variance weighted	3	-0.12	0.000639	0.88	0.82	0.95
ENSG00000147853	AK3	PD	Inverse variance weighted	5	0.15	0.000686	1.16	1.06	1.26
ENSG00000172269	DPAGT1	PD	Wald ratio	1	-0.22	0.000703	0.80	0.71	0.91
ENSG00000134193	REG4	PD	Wald ratio	1	-0.72	0.000716	0.49	0.32	0.74
ENSG00000244682	#N/A	PD	Inverse variance weighted	6	0.21	0.000732	1.23	1.09	1.38
ENSG00000267937	#N/A	PD	Wald ratio	1	0.98	0.000746	2.67	1.51	4.72
ENSG00000162616	DNAJB4	PD	Wald ratio	1	0.22	0.000758	1.25	1.10	1.42
ENSG00000095906	NUBP2	PD	Wald ratio	1	0.41	0.000826	1.50	1.18	1.91
ENSG00000175556	LONRF3	PD	Wald ratio	1	-0.38	0.000828	0.68	0.55	0.85
ENSG00000110090	CPT1A	PD	Inverse variance weighted	2	0.16	0.000855	1.17	1.07	1.29
ENSG00000114316	USP4	PD	Inverse variance weighted	3	-0.43	0.000857	0.65	0.51	0.84
ENSG00000147439	BIN3	PD	Inverse variance weighted	3	0.13	0.000884	1.13	1.05	1.22
ENSG00000188486	H2AX	PD	Wald ratio	1	0.48	0.000899	1.62	1.22	2.15
ENSG00000178977	LINC00324	PD	Inverse variance weighted	4	0.13	0.000918	1.14	1.06	1.24
ENSG00000167074	TEF	PD	Inverse variance weighted	4	-0.12	0.00094	0.89	0.83	0.95
ENSG00000180185	FAHD1	PD	Inverse variance weighted	3	-0.13	0.000947	0.88	0.81	0.95
ENSG00000170356	OR2A20P	PD	Inverse variance weighted	4	0.19	0.000956	1.21	1.08	1.36
ENSG00000035141	FAM136A	PD	Inverse variance weighted	2	-0.21	0.00099	0.81	0.72	0.92
ENSG00000183751	TBL3	PD	Wald ratio	1	0.66	0.001029	1.94	1.31	2.87
ENSG00000113319	RASGRF2	PD	Inverse variance weighted	2	-0.31	0.001034	0.74	0.61	0.88
ENSG00000189079	ARID2	PD	Wald ratio	1	-0.72	0.001059	0.49	0.31	0.75
ENSG00000172995	ARPP21	PD	Wald ratio	1	0.93	0.001085	2.52	1.45	4.40
ENSG00000206053	JPT2	PD	Wald ratio	1	0.47	0.001091	1.61	1.21	2.13
ENSG00000198554	WDHD1	PD	Wald ratio	1	-0.73	0.00112	0.48	0.31	0.75
ENSG00000147548	NSD3	PD	Wald ratio	1	-0.24	0.001121	0.78	0.68	0.91
ENSG00000131061	ZNF341	PD	Wald ratio	1	0.44	0.001181	1.55	1.19	2.02
ENSG00000197774	EME2	PD	Wald ratio	1	-0.23	0.001216	0.80	0.70	0.91
ENSG00000253200	#N/A	PD	Wald ratio	1	-0.42	0.001321	0.66	0.51	0.85
ENSG00000239556	#N/A	PD	Wald ratio	1	-0.18	0.001371	0.84	0.75	0.93
ENSG00000088836	SLC4A11	PD	Wald ratio	1	-0.41	0.00138	0.67	0.52	0.85
ENSG00000231062	#N/A	PD	Wald ratio	1	-0.44	0.001398	0.64	0.49	0.84
ENSG00000229390	#N/A	PD	Inverse variance weighted	4	-0.14	0.001465	0.87	0.80	0.95
ENSG00000196296	ATP2A1	PD	Wald ratio	1	-0.41	0.001517	0.66	0.51	0.85
ENSG00000115350	POLE4	PD	Inverse variance weighted	3	-0.39	0.001522	0.68	0.54	0.86
ENSG00000180573	H2AC6	PD	Inverse variance weighted	6	0.18	0.001529	1.20	1.07	1.34
ENSG00000235290	#N/A	PD	Inverse variance weighted	5	-0.17	0.001545	0.85	0.76	0.94
ENSG00000103160	HSDL1	PD	Inverse variance weighted	4	0.09	0.001549	1.10	1.04	1.17
ENSG00000173226	IQCB1	PD	Inverse variance weighted	4	-0.09	0.001572	0.92	0.87	0.97

ENSG00000158158	CNNM4	PD	Wald ratio	1	-0.18	0.001573	0.83	0.74	0.93
ENSG00000164615	CAMLG	PD	Inverse variance weighted	4	-0.12	0.001581	0.89	0.83	0.96
ENSG00000251692	PTX4	PD	Wald ratio	1	0.67	0.001605	1.95	1.29	2.95
ENSG00000188467	SLC24A5	PD	Inverse variance weighted	2	-0.11	0.001663	0.90	0.84	0.96
ENSG00000178971	CTC1	PD	Inverse variance weighted	4	0.10	0.001686	1.10	1.04	1.17
ENSG00000170175	CHRNB1	PD	Inverse variance weighted	2	0.13	0.001704	1.14	1.05	1.23
ENSG00000181754	AMIGO1	PD	Wald ratio	1	-0.17	0.001713	0.85	0.76	0.94
ENSG00000187778	MCRS1	PD	Wald ratio	1	-0.89	0.001727	0.41	0.23	0.72
ENSG00000139726	DENR	PD	Wald ratio	1	-0.25	0.001774	0.78	0.66	0.91
ENSG00000251417	#N/A	PD	Wald ratio	1	0.29	0.001809	1.33	1.11	1.59
ENSG00000169696	ASPSCR1	PD	Wald ratio	1	0.19	0.001814	1.20	1.07	1.35
ENSG00000230358	SPDYE21	PD	Wald ratio	1	0.40	0.001853	1.50	1.16	1.93
ENSG00000178188	SH2B1	PD	Wald ratio	1	0.62	0.001917	1.86	1.26	2.74
ENSG00000249042	#N/A	PD	Wald ratio	1	0.56	0.001923	1.75	1.23	2.50
ENSG00000101445	PPP1R16B	PD	Inverse variance weighted	2	0.27	0.001939	1.30	1.10	1.54
ENSG00000211776	TRAV2	PD	Inverse variance weighted	2	0.51	0.001943	1.67	1.21	2.31
ENSG00000158480	SPATA2	PD	Wald ratio	1	-0.70	0.001974	0.50	0.32	0.77
ENSG00000188612	SUMO2	PD	Wald ratio	1	-0.36	0.001977	0.70	0.55	0.88
ENSG00000174456	C12orf76	PD	Inverse variance weighted	6	-0.16	0.001991	0.85	0.77	0.94
ENSG00000082898	XPO1	PD	Wald ratio	1	-0.69	0.002004	0.50	0.32	0.78
ENSG00000108312	UBTF	PD	Wald ratio	1	-0.38	0.002013	0.68	0.54	0.87
ENSG00000160678	S100A1	PD	Inverse variance weighted	2	0.31	0.002057	1.36	1.12	1.66
ENSG00000178464	RPL10P16	PD	Wald ratio	1	-0.54	0.002091	0.58	0.41	0.82
ENSG00000188033	ZNF490	PD	Wald ratio	1	0.38	0.002091	1.46	1.15	1.85
ENSG00000145217	SLC26A1	PD	Wald ratio	1	0.22	0.002133	1.25	1.08	1.45
ENSG00000205352	PRR13	PD	Inverse variance weighted	3	0.18	0.002138	1.19	1.07	1.33
ENSG00000136628	EPRS1	PD	Inverse variance weighted	2	-0.13	0.002174	0.88	0.81	0.96
ENSG00000136819	C9orf78	PD	Inverse variance weighted	10	-0.08	0.002232	0.93	0.88	0.97
ENSG00000124201	ZNFX1	PD	Inverse variance weighted	2	0.18	0.002254	1.19	1.07	1.34
ENSG00000130511	SSBP4	PD	Wald ratio	1	0.44	0.002301	1.55	1.17	2.06
ENSG00000152128	TMEM163	PD	Wald ratio	1	0.17	0.002327	1.18	1.06	1.32
ENSG00000183682	BMP8A	PD	Inverse variance weighted	3	0.23	0.002388	1.26	1.09	1.46
ENSG00000163930	BAP1	PD	Wald ratio	1	-0.65	0.002491	0.52	0.34	0.80
ENSG00000236296	GUSBP5	PD	Inverse variance weighted	6	-0.11	0.002524	0.89	0.83	0.96
ENSG00000235027	#N/A	PD	Inverse variance weighted	2	-0.34	0.002533	0.71	0.57	0.89
ENSG00000182158	CREB3L2	PD	Wald ratio	1	0.54	0.002551	1.72	1.21	2.44
ENSG00000173960	UBXN2A	PD	Inverse variance weighted	3	-0.13	0.002631	0.88	0.80	0.95
ENSG00000184445	KNTC1	PD	Wald ratio	1	-0.22	0.002636	0.80	0.70	0.93
ENSG00000197646	PDCD1LG2	PD	Inverse variance weighted	2	-0.22	0.002672	0.80	0.69	0.93
ENSG00000155755	TMEM237	PD	Inverse variance weighted	3	0.21	0.002846	1.23	1.07	1.42
ENSG00000234456	MAGI2-AS3	PD	Inverse variance weighted	3	-0.15	0.002851	0.86	0.78	0.95
ENSG00000212719	LINC02693	PD	Wald ratio	1	0.31	0.002868	1.37	1.11	1.68
ENSG00000222365	SNORD12B	PD	Wald ratio	1	0.12	0.002875	1.12	1.04	1.21

ENSG00000262500	MAPK8IP1P1	PD	Inverse variance weighted	2	-0.56	0.002913	0.57	0.39	0.82
ENSG00000185721	DRG1	PD	Inverse variance weighted	2	0.29	0.002947	1.33	1.10	1.61
ENSG00000162817	C1orf115	PD	Inverse variance weighted	6	0.11	0.003003	1.12	1.04	1.20
ENSG00000138768	USO1	PD	Wald ratio	1	-0.19	0.003063	0.83	0.73	0.94
ENSG00000078967	UBE2D4	PD	Wald ratio	1	-0.28	0.003101	0.75	0.63	0.91
ENSG00000163823	CCR1	PD	Inverse variance weighted	9	-0.10	0.003101	0.90	0.85	0.97
ENSG00000267049	#N/A	PD	Wald ratio	1	-0.55	0.003196	0.58	0.40	0.83
ENSG00000140995	DEF8	PD	Inverse variance weighted	4	0.19	0.003233	1.21	1.07	1.38
ENSG00000259030	FPGT-TNNI3K	PD	Wald ratio	1	-0.43	0.00327	0.65	0.49	0.87
ENSG00000244932	#N/A	PD	Wald ratio	1	0.44	0.003309	1.56	1.16	2.09
ENSG00000198408	OGA	PD	Wald ratio	1	-0.73	0.003392	0.48	0.30	0.79
ENSG00000182372	CLN8	PD	Inverse variance weighted	2	0.16	0.003393	1.18	1.06	1.31
ENSG00000179195	ZNF664	PD	Inverse variance weighted	2	-0.19	0.003422	0.82	0.72	0.94
ENSG00000163812	ZDHHC3	PD	Wald ratio	1	0.34	0.003456	1.40	1.12	1.75
ENSG00000162929	SANBR	PD	Inverse variance weighted	6	-0.10	0.003472	0.90	0.85	0.97
ENSG00000138246	DNAJC13	PD	Inverse variance weighted	3	0.20	0.003502	1.22	1.07	1.39
ENSG00000267214	#N/A	PD	Wald ratio	1	-0.93	0.003542	0.40	0.21	0.74
ENSG00000164776	PHKG1	PD	Inverse variance weighted	2	-0.25	0.003619	0.78	0.66	0.92
ENSG00000268804	#N/A	PD	Wald ratio	1	-0.19	0.003636	0.83	0.73	0.94
ENSG00000226252	#N/A	PD	Wald ratio	1	-0.86	0.003689	0.42	0.24	0.76
ENSG00000136235	GPNMB	PD	Inverse variance weighted	2	0.44	0.003694	1.56	1.15	2.10
ENSG00000143157	POGK	PD	Wald ratio	1	-0.22	0.003726	0.80	0.69	0.93
ENSG00000163126	ANKRD23	PD	Wald ratio	1	0.45	0.003797	1.57	1.16	2.14
ENSG00000132359	RAP1GAP2	PD	Inverse variance weighted	4	-0.26	0.003867	0.77	0.64	0.92
ENSG00000090861	AARS1	PD	Inverse variance weighted	3	-0.25	0.003878	0.78	0.66	0.92
ENSG00000163214	DHX57	PD	Wald ratio	1	0.15	0.003909	1.16	1.05	1.28
ENSG00000182109	#N/A	PD	Inverse variance weighted	4	0.17	0.003938	1.18	1.05	1.32
ENSG00000135919	SERPINE2	PD	Inverse variance weighted	6	-0.11	0.003939	0.89	0.83	0.96
ENSG00000196576	PLXNB2	PD	Inverse variance weighted	4	0.15	0.003942	1.16	1.05	1.28
ENSG00000096063	SRPK1	PD	Inverse variance weighted	8	-0.10	0.003952	0.90	0.84	0.97
ENSG00000228107	#N/A	PD	Wald ratio	1	0.51	0.00396	1.67	1.18	2.37
ENSG00000176597	B3GNT5	PD	Inverse variance weighted	2	0.16	0.003969	1.18	1.05	1.31
ENSG00000257167	TMPO-AS1	PD	Wald ratio	1	-0.93	0.003974	0.39	0.21	0.74
ENSG00000163913	IFT122	PD	Wald ratio	1	0.41	0.004047	1.51	1.14	2.01
ENSG00000198216	CACNA1E	PD	Inverse variance weighted	4	-0.20	0.004091	0.82	0.72	0.94
ENSG00000167861	HID1	PD	Wald ratio	1	-0.51	0.004099	0.60	0.43	0.85
ENSG00000104219	ZDHHC2	PD	Inverse variance weighted	4	0.13	0.004148	1.14	1.04	1.24
ENSG00000265242	#N/A	PD	Wald ratio	1	-0.54	0.004165	0.58	0.40	0.84
ENSG00000166908	PIP4K2C	PD	Wald ratio	1	0.31	0.004265	1.36	1.10	1.69
ENSG00000255046	#N/A	PD	Wald ratio	1	0.16	0.00429	1.18	1.05	1.32
ENSG00000160691	SHC1	PD	Inverse variance weighted	2	0.12	0.004304	1.12	1.04	1.22
ENSG00000078747	ITCH	PD	Wald ratio	1	0.19	0.004308	1.20	1.06	1.37

ENSG00000019995	ZRANB1	PD	Inverse variance weighted	4	0.10	0.004325	1.11	1.03	1.19
ENSG00000171067	C11orf24	PD	Wald ratio	1	-0.34	0.004326	0.71	0.57	0.90
ENSG00000005884	ITGA3	PD	Inverse variance weighted	2	0.32	0.004351	1.38	1.11	1.72
ENSG00000136044	APPL2	PD	Inverse variance weighted	3	-0.12	0.004435	0.88	0.81	0.96
ENSG00000205482	SPDYE18	PD	Wald ratio	1	-0.34	0.004472	0.71	0.57	0.90
ENSG00000152382	TADA1	PD	Wald ratio	1	0.29	0.004542	1.33	1.09	1.63
ENSG00000205746	PKD1P4	PD	Inverse variance weighted	3	0.50	0.004643	1.64	1.16	2.31
ENSG00000206535	LNP1	PD	Wald ratio	1	-0.39	0.004709	0.67	0.51	0.89
ENSG00000100401	RANGAP1	PD	Wald ratio	1	0.33	0.004726	1.39	1.11	1.75
ENSG00000155066	PROM2	PD	Wald ratio	1	0.50	0.004789	1.64	1.16	2.32
ENSG00000155252	PI4K2A	PD	Wald ratio	1	-0.33	0.004806	0.72	0.57	0.90
ENSG00000123908	AGO2	PD	Inverse variance weighted	4	0.21	0.004854	1.24	1.07	1.43
ENSG00000260793	#N/A	PD	Wald ratio	1	0.43	0.004864	1.54	1.14	2.09
ENSG00000113621	TXNDC15	PD	Inverse variance weighted	2	-0.11	0.004877	0.89	0.83	0.97
ENSG00000222057	RNU4-62P	PD	Wald ratio	1	-0.71	0.004881	0.49	0.30	0.81
ENSG00000177479	ARIH2	PD	Wald ratio	1	0.21	0.00497	1.23	1.06	1.42
ENSG00000172059	KLF11	PD	Inverse variance weighted	4	-0.14	0.004988	0.87	0.79	0.96
ENSG00000158201	ABHD3	PD	Inverse variance weighted	6	-0.20	0.004997	0.82	0.71	0.94
ENSG00000243544	RN7SL172P	PD	Wald ratio	1	-0.20	0.005044	0.82	0.72	0.94
ENSG00000134900	TPP2	PD	Inverse variance weighted	4	0.11	0.005089	1.12	1.03	1.21
ENSG00000124571	XPO5	PD	Inverse variance weighted	2	-0.43	0.005101	0.65	0.48	0.88
ENSG00000178467	P4HTM	PD	Wald ratio	1	0.12	0.005236	1.13	1.04	1.23
ENSG00000149308	NPAT	PD	Wald ratio	1	-0.22	0.005279	0.80	0.68	0.94
ENSG00000132570	PCBD2	PD	Wald ratio	1	-0.65	0.005295	0.52	0.33	0.82
ENSG00000115827	DCAF17	PD	Wald ratio	1	0.49	0.005302	1.63	1.16	2.30
ENSG00000148396	SEC16A	PD	Inverse variance weighted	2	-0.12	0.005327	0.88	0.81	0.96
ENSG00000168101	NUDT16L1	PD	Wald ratio	1	0.48	0.005357	1.61	1.15	2.25
ENSG00000174792	ODAPH	PD	Wald ratio	1	0.49	0.005404	1.63	1.16	2.31
ENSG00000122729	ACO1	PD	Inverse variance weighted	4	0.21	0.005459	1.23	1.06	1.42
ENSG00000262468	#N/A	PD	Inverse variance weighted	2	0.26	0.005533	1.30	1.08	1.56
ENSG00000162366	PDZK1IP1	PD	Inverse variance weighted	14	-0.06	0.005615	0.94	0.90	0.98
ENSG00000145949	MYLK4	PD	Inverse variance weighted	2	-0.15	0.005712	0.86	0.77	0.96
ENSG00000104643	MTMR9	PD	Inverse variance weighted	2	-0.29	0.005742	0.75	0.61	0.92
ENSG00000213214	ARHGEF35	PD	Inverse variance weighted	2	0.12	0.005852	1.12	1.03	1.22
ENSG00000125458	NT5C	PD	Inverse variance weighted	2	0.13	0.005852	1.14	1.04	1.26
ENSG00000253239	IGLVI-70	PD	Inverse variance weighted	2	-0.40	0.005869	0.67	0.50	0.89
ENSG00000159403	C1R	PD	Wald ratio	1	-0.33	0.005894	0.72	0.56	0.91
ENSG00000176490	DIRAS1	PD	Wald ratio	1	0.19	0.005894	1.21	1.06	1.39
ENSG00000140403	DNAJA4	PD	Inverse variance weighted	5	0.11	0.005966	1.12	1.03	1.21
ENSG00000119630	PGF	PD	Wald ratio	1	-0.26	0.006119	0.77	0.64	0.93
ENSG00000183864	TOB2	PD	Wald ratio	1	0.35	0.006153	1.42	1.10	1.81
ENSG00000135052	GOLM1	PD	Inverse variance weighted	8	-0.12	0.006222	0.89	0.81	0.97
ENSG00000255198	SNHG9	PD	Inverse variance weighted	3	0.09	0.006285	1.09	1.03	1.17

ENSG00000115504	EHBP1	PD	Wald ratio	1	0.68	0.006355	1.97	1.21	3.19
ENSG00000174944	P2RY14	PD	Inverse variance weighted	6	-0.15	0.00637	0.86	0.77	0.96
ENSG00000224043	#N/A	PD	Wald ratio	1	0.45	0.006378	1.56	1.13	2.16
ENSG00000137876	RSL24D1	PD	Inverse variance weighted	2	-0.17	0.006384	0.84	0.74	0.95
ENSG00000159256	MORC3	PD	Wald ratio	1	0.11	0.006476	1.12	1.03	1.21
ENSG00000213672	NCKIPSD	PD	Wald ratio	1	0.15	0.006497	1.16	1.04	1.29
ENSG00000257698	GIHCG	PD	Wald ratio	1	0.20	0.006533	1.23	1.06	1.42
ENSG00000176946	THAP4	PD	Wald ratio	1	-0.39	0.006655	0.68	0.51	0.90
ENSG00000181350	LRRC75A	PD	Inverse variance weighted	4	0.17	0.006779	1.18	1.05	1.34
ENSG00000241627	#N/A	PD	Wald ratio	1	0.49	0.006799	1.62	1.14	2.31
ENSG00000148090	AUH	PD	Wald ratio	1	-0.48	0.006816	0.62	0.44	0.88
ENSG00000179388	EGR3	PD	Wald ratio	1	0.71	0.006888	2.04	1.22	3.43
ENSG00000171121	KCNMB3	PD	Wald ratio	1	0.42	0.007021	1.52	1.12	2.05
ENSG00000162650	ATXN7L2	PD	Wald ratio	1	-0.41	0.007159	0.66	0.49	0.89
ENSG00000250334	LINC00989	PD	Inverse variance weighted	4	0.13	0.007224	1.14	1.04	1.25
ENSG00000250506	CDK3	PD	Wald ratio	1	0.61	0.007237	1.84	1.18	2.86
ENSG00000162928	PEX13	PD	Wald ratio	1	-0.42	0.007289	0.66	0.49	0.89
ENSG00000173208	ABCD2	PD	Wald ratio	1	-0.22	0.007349	0.80	0.69	0.94
ENSG00000148356	LRSAM1	PD	Wald ratio	1	-0.28	0.007416	0.75	0.61	0.93
ENSG00000176681	LRRC37A	PD	Inverse variance weighted	3	-0.18	0.007519	0.83	0.73	0.95
ENSG00000238083	LRRC37A2	PD	Inverse variance weighted	4	-0.34	0.007682	0.71	0.55	0.91
ENSG00000171631	P2RY6	PD	Wald ratio	1	-0.74	0.007689	0.48	0.28	0.82
ENSG00000215424	MCM3AP-AS1	PD	Inverse variance weighted	2	-0.17	0.00775	0.84	0.74	0.96
ENSG00000140598	EFL1	PD	Inverse variance weighted	2	-0.38	0.007753	0.68	0.52	0.90
ENSG00000181904	C5orf24	PD	Wald ratio	1	-0.71	0.007773	0.49	0.29	0.83
ENSG00000186868	MAPT	PD	Wald ratio	1	0.64	0.00786	1.90	1.18	3.04
ENSG00000232300	#N/A	PD	Wald ratio	1	-0.17	0.00786	0.85	0.75	0.96
ENSG00000171160	MORN4	PD	Inverse variance weighted	2	0.14	0.007888	1.15	1.04	1.28
ENSG00000196396	PTPN1	PD	Wald ratio	1	-0.17	0.007905	0.84	0.75	0.96
ENSG00000163827	LRRC2	PD	Inverse variance weighted	2	-0.37	0.007975	0.69	0.53	0.91
ENSG00000147912	FBXO10	PD	Wald ratio	1	-0.33	0.008022	0.72	0.56	0.92
ENSG00000109084	TMEM97	PD	Inverse variance weighted	6	-0.10	0.008093	0.91	0.84	0.98
ENSG00000204564	C6orf136	PD	Inverse variance weighted	2	0.26	0.008099	1.30	1.07	1.59
ENSG00000134480	CCNH	PD	Wald ratio	1	-0.44	0.00812	0.65	0.47	0.89
ENSG00000260398	#N/A	PD	Wald ratio	1	-0.44	0.00812	0.64	0.46	0.89
ENSG00000241852	C8orf58	PD	Inverse variance weighted	2	0.16	0.008196	1.17	1.04	1.32
ENSG00000171236	LRG1	PD	Inverse variance weighted	5	-0.27	0.00822	0.76	0.62	0.93
ENSG00000156958	GALK2	PD	Wald ratio	1	0.17	0.008321	1.19	1.05	1.35
ENSG00000233695	#N/A	PD	Inverse variance weighted	2	-0.13	0.008385	0.87	0.79	0.97
ENSG00000167220	HDHD2	PD	Inverse variance weighted	2	0.12	0.008386	1.13	1.03	1.24
ENSG00000242247	ARFGAP3	PD	Inverse variance weighted	2	-0.15	0.008389	0.86	0.77	0.96
ENSG00000163281	GNPDA2	PD	Inverse variance weighted	2	-0.21	0.008438	0.81	0.69	0.95
ENSG00000149136	SSRP1	PD	Inverse variance weighted	2	0.23	0.008489	1.25	1.06	1.48

ENSG00000173809	TDRD12	PD	Wald ratio	1	-0.35	0.008492	0.71	0.55	0.92
ENSG00000225985	#N/A	PD	Wald ratio	1	0.83	0.008528	2.29	1.23	4.23
ENSG00000150990	DHX37	PD	Wald ratio	1	-0.43	0.008618	0.65	0.47	0.90
ENSG00000245213	GALNT7-DT	PD	Inverse variance weighted	3	0.27	0.008753	1.31	1.07	1.61
ENSG00000214194	SMIM30	PD	Inverse variance weighted	2	-0.28	0.008756	0.76	0.62	0.93
ENSG00000040633	PHF23	PD	Inverse variance weighted	3	-0.15	0.0088	0.86	0.77	0.96
ENSG00000261644	#N/A	PD	Wald ratio	1	-0.21	0.008818	0.81	0.70	0.95
ENSG00000269918	#N/A	PD	Inverse variance weighted	3	-0.13	0.008858	0.88	0.79	0.97
ENSG00000269772	#N/A	PD	Wald ratio	1	0.44	0.008879	1.55	1.12	2.14
ENSG00000163637	PRICKLE2	PD	Wald ratio	1	-0.57	0.008904	0.57	0.37	0.87
ENSG00000146085	MMUT	PD	Inverse variance weighted	7	0.09	0.008921	1.09	1.02	1.17
ENSG00000268568	#N/A	PD	Wald ratio	1	0.11	0.008931	1.12	1.03	1.21
ENSG00000241370	RPP21	PD	Wald ratio	1	-0.37	0.009001	0.69	0.53	0.91
ENSG00000171408	PDE7B	PD	Wald ratio	1	-0.55	0.00905	0.58	0.38	0.87
ENSG00000172005	MAL	PD	Inverse variance weighted	5	-0.12	0.009061	0.89	0.81	0.97
ENSG00000112972	HMGCS1	PD	Inverse variance weighted	2	-0.48	0.009072	0.62	0.43	0.89
ENSG00000188234	AGAP4	PD	Inverse variance weighted	4	-0.08	0.009106	0.93	0.87	0.98
ENSG00000104133	SPG11	PD	Wald ratio	1	-0.38	0.009109	0.68	0.51	0.91
ENSG00000164855	TMEM184A	PD	Wald ratio	1	-0.25	0.009197	0.78	0.64	0.94
ENSG00000130529	TRPM4	PD	Inverse variance weighted	10	-0.09	0.009216	0.91	0.85	0.98
ENSG00000243224	TWF2-DT	PD	Inverse variance weighted	2	-0.12	0.009387	0.88	0.81	0.97
ENSG00000216775	LOC730101	PD	Wald ratio	1	0.20	0.009516	1.22	1.05	1.41
ENSG00000229431	#N/A	PD	Wald ratio	1	-0.40	0.009609	0.67	0.49	0.91
ENSG00000177646	ACAD9	PD	Inverse variance weighted	4	0.20	0.009651	1.22	1.05	1.41
ENSG00000145723	GIN1	PD	Wald ratio	1	0.22	0.009664	1.24	1.05	1.46
ENSG00000173406	DAB1	PD	Inverse variance weighted	2	0.10	0.009684	1.11	1.03	1.20
ENSG00000258521	#N/A	PD	Wald ratio	1	-0.31	0.009689	0.73	0.58	0.93
ENSG00000229474	PATL2	PD	Inverse variance weighted	7	-0.11	0.009778	0.90	0.83	0.97
ENSG00000260051	#N/A	PD	Wald ratio	1	0.16	0.010063	1.18	1.04	1.34
ENSG00000169629	RGPD8	PD	Wald ratio	1	0.18	0.010067	1.19	1.04	1.36
ENSG00000255987	#N/A	PD	Inverse variance weighted	3	-0.23	0.010103	0.79	0.66	0.95
ENSG00000145113	MUC4	PD	Inverse variance weighted	2	-0.17	0.010108	0.84	0.74	0.96
ENSG00000183397	C19orf71	PD	Inverse variance weighted	3	-0.25	0.010176	0.78	0.64	0.94
ENSG00000162723	SLAMF9	PD	Wald ratio	1	-0.65	0.010182	0.52	0.32	0.86
ENSG00000259976	#N/A	PD	Wald ratio	1	0.86	0.010196	2.36	1.23	4.53
ENSG00000237149	ZNF503-AS2	PD	Wald ratio	1	0.42	0.010215	1.52	1.10	2.08
ENSG00000172653	#N/A	PD	Wald ratio	1	0.26	0.010236	1.29	1.06	1.58
ENSG00000243566	UPK3B	PD	Inverse variance weighted	3	-0.10	0.010251	0.91	0.84	0.98
ENSG00000247400	DNAJC3-DT	PD	Wald ratio	1	-0.21	0.010352	0.81	0.70	0.95
ENSG00000261338	#N/A	PD	Inverse variance weighted	4	0.11	0.010393	1.11	1.03	1.20

ENSG00000156113	KCNMA1	PD	Inverse variance weighted	4	0.10	0.010523	1.11	1.02	1.20
ENSG00000225217	#N/A	PD	Inverse variance weighted	2	0.18	0.010526	1.19	1.04	1.37
ENSG00000242797	#N/A	PD	Wald ratio	1	-0.31	0.010602	0.73	0.58	0.93
ENSG00000245317	LOC100996419	PD	Wald ratio	1	0.52	0.010637	1.68	1.13	2.50
ENSG00000199370	#N/A	PD	Wald ratio	1	-0.24	0.010855	0.79	0.66	0.95
ENSG00000205269	TMEM170B	PD	Inverse variance weighted	5	-0.15	0.010882	0.86	0.77	0.97
ENSG00000186834	HEXIM1	PD	Wald ratio	1	0.44	0.010883	1.55	1.11	2.17
ENSG00000167207	NOD2	PD	Inverse variance weighted	8	0.05	0.010892	1.05	1.01	1.09
ENSG00000269676	#N/A	PD	Wald ratio	1	-0.20	0.01099	0.82	0.70	0.96
ENSG00000089169	RPH3A	PD	Inverse variance weighted	6	-0.10	0.011018	0.90	0.84	0.98
ENSG00000101391	CDK5RAP1	PD	Wald ratio	1	0.47	0.011072	1.61	1.11	2.32
ENSG00000163932	PRKCD	PD	Inverse variance weighted	5	0.18	0.011117	1.19	1.04	1.37
ENSG00000156990	RPUSD3	PD	Wald ratio	1	0.16	0.011271	1.17	1.04	1.32
ENSG00000171223	JUNB	PD	Inverse variance weighted	2	-0.42	0.011296	0.66	0.48	0.91
ENSG00000138738	PRDM5	PD	Inverse variance weighted	4	-0.11	0.011341	0.89	0.82	0.97
ENSG00000088298	EDEM2	PD	Inverse variance weighted	2	-0.10	0.011358	0.90	0.83	0.98
ENSG00000237797	#N/A	PD	Wald ratio	1	0.77	0.011396	2.15	1.19	3.91
ENSG00000167515	TRAPPC2L	PD	Wald ratio	1	-0.15	0.011425	0.86	0.77	0.97
ENSG00000135541	AHI1	PD	Inverse variance weighted	4	-0.08	0.01147	0.92	0.87	0.98
ENSG00000130348	QRSL1	PD	Inverse variance weighted	3	-0.10	0.01148	0.90	0.83	0.98
ENSG00000169926	KLF13	PD	Wald ratio	1	-0.36	0.01149	0.70	0.53	0.92
ENSG00000241782	RPL21P95	PD	Wald ratio	1	0.35	0.011514	1.43	1.08	1.88
ENSG00000100393	EP300	PD	Wald ratio	1	-0.13	0.011527	0.88	0.79	0.97
ENSG00000148671	ADIRF	PD	Wald ratio	1	-0.35	0.011581	0.71	0.54	0.93
ENSG00000136827	TOR1A	PD	Inverse variance weighted	4	-0.11	0.011594	0.90	0.83	0.98
ENSG00000110075	PPP6R3	PD	Wald ratio	1	-0.22	0.011678	0.80	0.68	0.95
ENSG00000132964	CDK8	PD	Wald ratio	1	-0.36	0.01177	0.70	0.53	0.92
ENSG00000126264	HCST	PD	Inverse variance weighted	4	0.50	0.011816	1.65	1.12	2.43
ENSG00000234694	#N/A	PD	Inverse variance weighted	2	-0.20	0.011961	0.82	0.70	0.96
ENSG00000086062	B4GALT1	PD	Inverse variance weighted	3	0.33	0.011964	1.38	1.07	1.78
ENSG00000170027	YWHAG	PD	Inverse variance weighted	2	-0.11	0.011991	0.90	0.82	0.98
ENSG00000184863	RBM33	PD	Wald ratio	1	0.80	0.012074	2.23	1.19	4.18
ENSG00000169599	NFU1	PD	Wald ratio	1	-0.16	0.012125	0.85	0.75	0.96
ENSG00000169018	FEM1B	PD	Wald ratio	1	0.34	0.012152	1.40	1.08	1.82
ENSG00000197912	SPG7	PD	Wald ratio	1	0.15	0.012212	1.16	1.03	1.31
ENSG00000164484	TMEM200A	PD	Inverse variance weighted	2	0.15	0.012279	1.17	1.03	1.32
ENSG00000255310	#N/A	PD	Inverse variance weighted	3	-0.13	0.012329	0.88	0.80	0.97
ENSG00000143971	ETAA1	PD	Inverse variance weighted	2	0.26	0.012391	1.29	1.06	1.58
ENSG00000176809	LRRC37A3	PD	Wald ratio	1	-0.28	0.012419	0.76	0.61	0.94
ENSG00000257743	MGAM2	PD	Wald ratio	1	0.19	0.012419	1.21	1.04	1.41
ENSG00000164609	SLU7	PD	Inverse variance weighted	2	0.52	0.012543	1.69	1.12	2.54

ENSG00000234585	CCT6P3	PD	Inverse variance weighted	3	-0.10	0.012551	0.91	0.84	0.98
ENSG00000176720	BOK	PD	Inverse variance weighted	3	0.11	0.012584	1.12	1.02	1.22
ENSG00000225450	#N/A	PD	Wald ratio	1	0.19	0.012616	1.21	1.04	1.41
ENSG00000243970	PPIEL	PD	Inverse variance weighted	3	0.19	0.012651	1.21	1.04	1.41
ENSG00000124198	ARFGEF2	PD	Wald ratio	1	0.18	0.012678	1.20	1.04	1.38
ENSG00000230953	#N/A	PD	Wald ratio	1	-0.44	0.012713	0.64	0.46	0.91
ENSG00000185499	MUC1	PD	Inverse variance weighted	5	0.14	0.012762	1.15	1.03	1.28
ENSG00000175203	DCTN2	PD	Wald ratio	1	0.71	0.012774	2.04	1.16	3.58
ENSG00000138172	CALHM2	PD	Inverse variance weighted	3	0.09	0.012975	1.09	1.02	1.17
ENSG00000172367	PDZD3	PD	Inverse variance weighted	2	-0.15	0.012992	0.86	0.77	0.97
ENSG00000236540	#N/A	PD	Inverse variance weighted	2	0.24	0.013137	1.27	1.05	1.53
ENSG00000196968	FUT11	PD	Wald ratio	1	0.30	0.013152	1.35	1.06	1.71
ENSG00000180776	ZDHHC20	PD	Inverse variance weighted	2	-0.18	0.013275	0.83	0.72	0.96
ENSG00000270120	#N/A	PD	Inverse variance weighted	3	0.09	0.013344	1.09	1.02	1.17
ENSG00000128271	ADORA2A	PD	Wald ratio	1	0.20	0.013489	1.23	1.04	1.44
ENSG00000132749	TESMIN	PD	Wald ratio	1	0.11	0.013489	1.11	1.02	1.21
ENSG00000238142	LOC105376805	PD	Inverse variance weighted	2	-0.18	0.01353	0.84	0.72	0.96
ENSG00000177303	CASKIN2	PD	Wald ratio	1	0.33	0.013534	1.39	1.07	1.81
ENSG00000237914	SIRPG-AS1	PD	Inverse variance weighted	4	0.10	0.013562	1.10	1.02	1.19
ENSG00000228137	#N/A	PD	Wald ratio	1	-0.63	0.013565	0.53	0.32	0.88
ENSG00000133805	AMPD3	PD	Inverse variance weighted	5	0.16	0.013573	1.17	1.03	1.33
ENSG00000138593	SECISBP2L	PD	Wald ratio	1	0.45	0.013688	1.56	1.10	2.23
ENSG00000143379	SETDB1	PD	Wald ratio	1	0.24	0.013767	1.27	1.05	1.53
ENSG00000123427	EEF1AKMT3	PD	Inverse variance weighted	5	-0.08	0.013812	0.92	0.86	0.98
ENSG00000237883	DGUOK-AS1	PD	Inverse variance weighted	2	0.55	0.013851	1.74	1.12	2.69
ENSG00000144504	ANKMY1	PD	Inverse variance weighted	4	0.09	0.013866	1.10	1.02	1.18
ENSG00000269947	#N/A	PD	Wald ratio	1	0.45	0.013876	1.56	1.10	2.23
ENSG00000090565	RAB11FIP3	PD	Wald ratio	1	0.46	0.013939	1.58	1.10	2.28
ENSG00000130201	#N/A	PD	Wald ratio	1	-0.45	0.013966	0.64	0.45	0.91
ENSG00000143776	CDC42BPA	PD	Inverse variance weighted	2	-0.24	0.014065	0.79	0.65	0.95
ENSG00000128438	#N/A	PD	Wald ratio	1	0.41	0.014077	1.51	1.09	2.11
ENSG00000172766	NAA16	PD	Inverse variance weighted	2	-0.16	0.01412	0.85	0.75	0.97
ENSG00000143457	GOLPH3L	PD	Inverse variance weighted	3	-0.25	0.014154	0.78	0.64	0.95
ENSG00000096092	TMEM14A	PD	Wald ratio	1	0.21	0.014165	1.24	1.04	1.47
ENSG00000167543	TP53I13	PD	Inverse variance weighted	2	-0.19	0.01442	0.82	0.71	0.96
ENSG00000207110	RNVU1-32	PD	Wald ratio	1	0.29	0.014436	1.34	1.06	1.69
ENSG00000239415	#N/A	PD	Inverse variance weighted	2	-0.12	0.014445	0.89	0.81	0.98
ENSG00000187166	H1-7	PD	Wald ratio	1	-0.20	0.014508	0.82	0.70	0.96
ENSG00000266631	#N/A	PD	Wald ratio	1	-0.58	0.014581	0.56	0.35	0.89
ENSG00000148848	ADAM12	PD	Inverse variance weighted	2	0.24	0.014592	1.28	1.05	1.55

ENSG00000204745	LOC102724642	PD	Inverse variance weighted	2	-0.24	0.014695	0.78	0.64	0.95
ENSG00000139636	LMBR1L	PD	Wald ratio	1	-0.42	0.014705	0.66	0.47	0.92
ENSG00000230487	PSMG3-AS1	PD	Inverse variance weighted	4	0.15	0.01473	1.16	1.03	1.31
ENSG00000182636	NDN	PD	Inverse variance weighted	4	-0.10	0.015045	0.90	0.83	0.98
ENSG00000178974	FBXO34	PD	Wald ratio	1	-0.16	0.015056	0.86	0.75	0.97
ENSG00000104881	PPP1R13L	PD	Wald ratio	1	0.62	0.01507	1.86	1.13	3.08
ENSG00000163466	ARPC2	PD	Wald ratio	1	0.30	0.015094	1.35	1.06	1.72
ENSG00000123684	LPGAT1	PD	Inverse variance weighted	2	-0.15	0.015142	0.86	0.76	0.97
ENSG00000197442	MAP3K5	PD	Inverse variance weighted	3	-0.09	0.015181	0.91	0.84	0.98
ENSG00000127837	AAMP	PD	Wald ratio	1	0.32	0.015192	1.37	1.06	1.77
ENSG00000099282	TSPAN15	PD	Inverse variance weighted	2	-0.23	0.01527	0.79	0.66	0.96
ENSG00000123416	TUBA1B	PD	Wald ratio	1	0.37	0.015286	1.45	1.07	1.97
ENSG00000255561	FDXACB1	PD	Inverse variance weighted	2	-0.48	0.015335	0.62	0.42	0.91
ENSG00000178719	GRINA	PD	Inverse variance weighted	3	-0.09	0.015437	0.91	0.84	0.98
ENSG00000168209	DDIT4	PD	Wald ratio	1	-0.52	0.015466	0.60	0.39	0.91
ENSG00000151014	NOCT	PD	Inverse variance weighted	2	-0.43	0.01547	0.65	0.46	0.92
ENSG00000115355	CCDC88A	PD	Inverse variance weighted	2	0.17	0.015683	1.19	1.03	1.37
ENSG00000156671	SAMD8	PD	Wald ratio	1	0.53	0.015744	1.70	1.11	2.62
ENSG00000163938	GNL3	PD	Wald ratio	1	-0.20	0.01577	0.82	0.70	0.96
ENSG00000157322	CLEC18A	PD	Inverse variance weighted	4	0.08	0.015788	1.08	1.02	1.16
ENSG00000172273	HINFP	PD	Inverse variance weighted	3	0.10	0.015815	1.10	1.02	1.20
ENSG00000157514	TSC22D3	PD	Wald ratio	1	0.73	0.016018	2.08	1.15	3.78
ENSG00000242607	RPS3AP34	PD	Wald ratio	1	-0.84	0.016043	0.43	0.22	0.86
ENSG00000152672	CLEC4F	PD	Inverse variance weighted	7	-0.05	0.016054	0.95	0.91	0.99
ENSG00000216895	LOC100506302	PD	Wald ratio	1	-0.24	0.016083	0.78	0.64	0.96
ENSG00000151806	GUF1	PD	Inverse variance weighted	2	-0.12	0.01618	0.89	0.80	0.98
ENSG00000205726	ITSN1	PD	Inverse variance weighted	5	0.08	0.01621	1.09	1.02	1.16
ENSG00000135766	EGLN1	PD	Inverse variance weighted	3	0.13	0.016334	1.13	1.02	1.26
ENSG00000225992	#N/A	PD	Inverse variance weighted	2	-0.26	0.016423	0.77	0.63	0.95
ENSG00000132953	XPO4	PD	Wald ratio	1	0.27	0.016643	1.30	1.05	1.62
ENSG00000148516	ZEB1	PD	Wald ratio	1	0.61	0.016669	1.84	1.12	3.03
ENSG00000258839	MC1R	PD	Inverse variance weighted	3	-0.09	0.016851	0.92	0.85	0.98
ENSG00000139428	MMAB	PD	Wald ratio	1	0.15	0.01687	1.16	1.03	1.32
ENSG00000167657	DAPK3	PD	Inverse variance weighted	2	0.28	0.016886	1.33	1.05	1.67
ENSG00000067596	DHX8	PD	Wald ratio	1	0.24	0.017016	1.28	1.04	1.56
ENSG00000157181	ODR4	PD	Wald ratio	1	-0.35	0.017016	0.71	0.53	0.94
ENSG00000212093	#N/A	PD	Wald ratio	1	-0.19	0.017026	0.83	0.71	0.97
ENSG00000270028	#N/A	PD	Wald ratio	1	0.40	0.017047	1.50	1.07	2.09
ENSG00000253341	#N/A	PD	Wald ratio	1	-0.39	0.017049	0.68	0.49	0.93
ENSG00000165959	CLMN	PD	Inverse variance weighted	4	-0.12	0.017139	0.89	0.80	0.98
ENSG00000148773	MKI67	PD	Wald ratio	1	-0.60	0.017157	0.55	0.33	0.90

ENSG00000213626	LBH	PD	Inverse variance weighted	4	-0.13	0.017206	0.88	0.79	0.98
ENSG00000197586	ENTPD6	PD	Inverse variance weighted	3	-0.18	0.017238	0.84	0.72	0.97
ENSG00000270175	#N/A	PD	Wald ratio	1	-0.30	0.017292	0.74	0.58	0.95
ENSG00000255449	#N/A	PD	Wald ratio	1	0.28	0.017316	1.33	1.05	1.67
ENSG00000258867	LINC01146	PD	Wald ratio	1	-0.27	0.017319	0.76	0.61	0.95
ENSG00000125148	MT2A	PD	Inverse variance weighted	8	0.09	0.017423	1.09	1.02	1.18
ENSG00000179163	FUCA1	PD	Inverse variance weighted	7	-0.09	0.017456	0.91	0.84	0.98
ENSG00000270107	#N/A	PD	Inverse variance weighted	2	0.21	0.017506	1.24	1.04	1.47
ENSG00000198879	SFMBT2	PD	Inverse variance weighted	2	-0.16	0.01753	0.85	0.75	0.97
ENSG00000205763	RP9P	PD	Wald ratio	1	-0.53	0.017549	0.59	0.38	0.91
ENSG00000235560	ZNF747-DT	PD	Inverse variance weighted	2	0.19	0.017615	1.21	1.03	1.41
ENSG00000154175	ABI3BP	PD	Inverse variance weighted	2	-0.30	0.017634	0.74	0.58	0.95
ENSG00000182544	MFSD5	PD	Inverse variance weighted	3	-0.11	0.017698	0.90	0.82	0.98
ENSG00000096968	JAK2	PD	Inverse variance weighted	3	0.16	0.017712	1.17	1.03	1.34
ENSG00000267724	#N/A	PD	Wald ratio	1	0.48	0.017761	1.62	1.09	2.40
ENSG00000225331	#N/A	PD	Inverse variance weighted	2	-0.18	0.017789	0.84	0.73	0.97
ENSG00000143315	PIGM	PD	Inverse variance weighted	3	-0.13	0.017791	0.88	0.79	0.98
ENSG00000165555	NOXRED1	PD	Wald ratio	1	0.37	0.01794	1.45	1.07	1.98
ENSG00000138709	LARP1B	PD	Inverse variance weighted	2	0.22	0.017964	1.24	1.04	1.48
ENSG00000146731	CCT6A	PD	Wald ratio	1	-0.13	0.017972	0.87	0.78	0.98
ENSG00000206838	SNORA5A	PD	Wald ratio	1	-0.62	0.018022	0.54	0.32	0.90
ENSG00000244242	IFITM10	PD	Wald ratio	1	0.21	0.018052	1.24	1.04	1.48
ENSG00000225213	#N/A	PD	Wald ratio	1	-0.41	0.018057	0.66	0.47	0.93
ENSG00000180228	PRKRA	PD	Inverse variance weighted	2	0.16	0.018123	1.18	1.03	1.35
ENSG00000134291	TMEM106C	PD	Inverse variance weighted	6	0.09	0.018128	1.09	1.01	1.17
ENSG00000167797	CDK2AP2	PD	Inverse variance weighted	5	0.26	0.018152	1.30	1.05	1.61
ENSG00000232613	#N/A	PD	Wald ratio	1	-0.40	0.018282	0.67	0.48	0.93
ENSG00000244515	KRT18P34	PD	Wald ratio	1	0.31	0.018434	1.37	1.05	1.77
ENSG00000211640	IGLV6-57	PD	Wald ratio	1	-0.73	0.018519	0.48	0.26	0.88
ENSG00000198015	MRPL42	PD	Inverse variance weighted	2	-0.09	0.018589	0.91	0.85	0.99
ENSG00000259658	LOC100128108	PD	Wald ratio	1	-0.21	0.018639	0.81	0.68	0.97
ENSG00000124659	TBCC	PD	Wald ratio	1	0.42	0.018816	1.53	1.07	2.17
ENSG00000186468	RPS23	PD	Inverse variance weighted	5	0.08	0.018916	1.08	1.01	1.15
ENSG00000252645	RNU7-111P	PD	Inverse variance weighted	2	-0.12	0.019001	0.88	0.80	0.98
ENSG00000119777	TMEM214	PD	Wald ratio	1	-0.55	0.019046	0.58	0.36	0.91
ENSG00000125779	PANK2	PD	Inverse variance weighted	2	0.15	0.019047	1.16	1.02	1.31
ENSG00000185928	#N/A	PD	Wald ratio	1	-0.46	0.019055	0.63	0.43	0.93
ENSG00000203288	TDRKH-AS1	PD	Wald ratio	1	0.27	0.019058	1.31	1.04	1.64
ENSG00000198833	UBE2J1	PD	Inverse variance weighted	6	0.12	0.019225	1.13	1.02	1.25
ENSG00000230149	#N/A	PD	Wald ratio	1	0.64	0.019258	1.90	1.11	3.26
ENSG00000160294	MCM3AP	PD	Inverse variance weighted	2	-0.08	0.019261	0.93	0.87	0.99

ENSG00000249396	LINC02212	PD	Wald ratio	1	-0.68	0.019406	0.51	0.29	0.90
ENSG00000136383	ALPK3	PD	Wald ratio	1	0.40	0.019428	1.49	1.07	2.09
ENSG00000105011	ASF1B	PD	Inverse variance weighted	4	-0.25	0.019488	0.78	0.63	0.96
ENSG00000232874	LINC02924	PD	Wald ratio	1	-0.79	0.019498	0.45	0.23	0.88
ENSG00000261556	SMG1P7	PD	Inverse variance weighted	3	0.10	0.019499	1.10	1.02	1.19
ENSG00000140854	KATNB1	PD	Inverse variance weighted	2	0.22	0.019572	1.25	1.04	1.51
ENSG00000169957	ZNF768	PD	Wald ratio	1	0.46	0.019631	1.58	1.08	2.32
ENSG00000259684	#N/A	PD	Wald ratio	1	-0.21	0.019729	0.81	0.69	0.97
ENSG00000162378	ZYG11B	PD	Inverse variance weighted	5	0.10	0.019819	1.10	1.02	1.19
ENSG00000269220	LINC00528	PD	Inverse variance weighted	2	0.25	0.01983	1.28	1.04	1.57
ENSG00000116898	MRPS15	PD	Inverse variance weighted	2	0.20	0.019888	1.22	1.03	1.43
ENSG00000164669	INTS4P1	PD	Wald ratio	1	0.32	0.019934	1.37	1.05	1.80
ENSG00000261229	#N/A	PD	Wald ratio	1	0.79	0.019938	2.20	1.13	4.27
ENSG00000129993	CBFA2T3	PD	Inverse variance weighted	3	0.25	0.020033	1.28	1.04	1.57
ENSG00000259211	#N/A	PD	Wald ratio	1	0.48	0.020142	1.61	1.08	2.41
ENSG00000204314	PRRT1	PD	Wald ratio	1	0.39	0.020144	1.48	1.06	2.06
ENSG00000212127	TAS2R14	PD	Wald ratio	1	0.09	0.020317	1.10	1.01	1.19
ENSG00000155090	KLF10	PD	Wald ratio	1	-0.32	0.020397	0.73	0.56	0.95
ENSG00000168906	MAT2A	PD	Inverse variance weighted	3	-0.18	0.020559	0.83	0.71	0.97
ENSG00000163684	RPP14	PD	Wald ratio	1	0.27	0.02057	1.31	1.04	1.65
ENSG00000012779	ALOX5	PD	Inverse variance weighted	6	-0.16	0.020661	0.85	0.74	0.98
ENSG00000248593	DSTNP2	PD	Wald ratio	1	0.63	0.020679	1.88	1.10	3.22
ENSG00000171862	PTEN	PD	Wald ratio	1	0.63	0.020696	1.88	1.10	3.20
ENSG00000106245	BUD31	PD	Inverse variance weighted	3	-0.22	0.020827	0.80	0.67	0.97
ENSG00000144746	ARL6IP5	PD	Inverse variance weighted	3	0.20	0.020852	1.22	1.03	1.44
ENSG00000228409	#N/A	PD	Wald ratio	1	0.12	0.020869	1.13	1.02	1.24
ENSG00000261707	#N/A	PD	Inverse variance weighted	2	0.20	0.021002	1.22	1.03	1.44
ENSG00000131484	#N/A	PD	Inverse variance weighted	2	0.61	0.021102	1.84	1.10	3.09
ENSG00000225171	DUTP6	PD	Inverse variance weighted	2	0.10	0.021111	1.11	1.02	1.21
ENSG00000203772	SPRN	PD	Wald ratio	1	-0.63	0.021128	0.53	0.31	0.91
ENSG00000206503	HLA-A	PD	Inverse variance weighted	6	-0.07	0.021254	0.93	0.87	0.99
ENSG00000160803	UBQLN4	PD	Wald ratio	1	-0.14	0.021287	0.87	0.77	0.98
ENSG00000156172	CFAP418	PD	Wald ratio	1	-0.35	0.021415	0.71	0.53	0.95
ENSG00000204241	LINC02731	PD	Inverse variance weighted	2	0.20	0.021617	1.23	1.03	1.46
ENSG00000139977	NAA30	PD	Wald ratio	1	0.24	0.021692	1.27	1.04	1.56
ENSG00000198964	SGMS1	PD	Wald ratio	1	-0.59	0.021852	0.56	0.34	0.92
ENSG00000170017	ALCAM	PD	Inverse variance weighted	6	-0.10	0.021897	0.91	0.83	0.99
ENSG00000142185	TRPM2	PD	Inverse variance weighted	2	-0.16	0.021924	0.86	0.75	0.98
ENSG00000175806	MSRA	PD	Inverse variance weighted	8	0.10	0.022177	1.10	1.01	1.20
ENSG00000234127	TRIM26	PD	Inverse variance weighted	2	0.16	0.022185	1.18	1.02	1.35
ENSG00000135390	ATP5MC2	PD	Inverse variance weighted	2	0.16	0.022207	1.17	1.02	1.34
ENSG00000198182	ZNF607	PD	Inverse variance weighted	2	0.21	0.022561	1.23	1.03	1.47
ENSG00000256087	ZNF432	PD	Wald ratio	1	0.51	0.022628	1.66	1.07	2.57
ENSG00000185507	IRF7	PD	Inverse variance weighted	5	0.13	0.02265	1.14	1.02	1.27

ENSG00000099860	GADD45B	PD	Inverse variance weighted	5	-0.19	0.022658	0.83	0.71	0.97
ENSG00000173320	STOX2	PD	Wald ratio	1	0.43	0.022686	1.54	1.06	2.22
ENSG00000256294	ZNF225	PD	Wald ratio	1	-0.51	0.022709	0.60	0.39	0.93
ENSG00000077458	FAM76B	PD	Inverse variance weighted	3	0.10	0.022724	1.11	1.01	1.21
ENSG00000042286	AIFM2	PD	Inverse variance weighted	2	0.14	0.022794	1.15	1.02	1.29
ENSG00000143418	CERS2	PD	Inverse variance weighted	3	-0.13	0.02285	0.88	0.79	0.98
ENSG00000233850	#N/A	PD	Wald ratio	1	-0.45	0.022854	0.64	0.43	0.94
ENSG00000189159	JPT1	PD	Inverse variance weighted	2	-0.25	0.022892	0.78	0.62	0.97
ENSG00000198218	QRICH1	PD	Wald ratio	1	-0.12	0.022906	0.89	0.81	0.98
ENSG00000228923	#N/A	PD	Wald ratio	1	-0.19	0.023106	0.83	0.70	0.97
ENSG00000262152	#N/A	PD	Wald ratio	1	-0.40	0.02311	0.67	0.47	0.95
ENSG00000262362	#N/A	PD	Wald ratio	1	0.56	0.02311	1.75	1.08	2.84
ENSG00000077235	GTF3C1	PD	Wald ratio	1	-0.77	0.023201	0.46	0.24	0.90
ENSG00000262745	#N/A	PD	Wald ratio	1	0.58	0.023254	1.78	1.08	2.93
ENSG00000196262	PPIA	PD	Wald ratio	1	0.13	0.023324	1.14	1.02	1.27
ENSG00000163660	CCNL1	PD	Wald ratio	1	-0.09	0.023349	0.92	0.85	0.99
ENSG00000143756	FBXO28	PD	Inverse variance weighted	5	-0.08	0.023449	0.93	0.87	0.99
ENSG00000133111	RFXAP	PD	Wald ratio	1	0.67	0.023462	1.96	1.09	3.50
ENSG00000172183	ISG20	PD	Inverse variance weighted	10	-0.08	0.023548	0.92	0.86	0.99
ENSG00000164237	CMBL	PD	Inverse variance weighted	7	-0.07	0.023596	0.93	0.87	0.99
ENSG00000225762	#N/A	PD	Wald ratio	1	0.56	0.023664	1.76	1.08	2.87
ENSG00000178803	ADORA2A-AS1	PD	Wald ratio	1	0.52	0.023907	1.69	1.07	2.66
ENSG00000200235	#N/A	PD	Wald ratio	1	0.35	0.023919	1.42	1.05	1.93
ENSG00000144231	POLR2D	PD	Inverse variance weighted	2	-0.14	0.023938	0.87	0.77	0.98
ENSG00000184677	ZBTB40	PD	Wald ratio	1	-0.12	0.023942	0.89	0.80	0.98
ENSG00000158445	KCNB1	PD	Inverse variance weighted	2	0.28	0.023973	1.33	1.04	1.70
ENSG00000131126	TEX101	PD	Inverse variance weighted	5	0.13	0.023986	1.14	1.02	1.27
ENSG00000261118	#N/A	PD	Wald ratio	1	0.45	0.024187	1.56	1.06	2.30
ENSG00000149577	SIDT2	PD	Inverse variance weighted	17	-0.05	0.024197	0.95	0.91	0.99
ENSG00000188672	RHCE	PD	Wald ratio	1	0.31	0.024267	1.37	1.04	1.80
ENSG00000179314	WSCD1	PD	Wald ratio	1	0.62	0.024326	1.85	1.08	3.17
ENSG00000044459	CNTLN	PD	Wald ratio	1	-0.21	0.024449	0.81	0.68	0.97
ENSG00000228696	ARL17A	PD	Inverse variance weighted	3	0.14	0.024499	1.15	1.02	1.31
ENSG00000152939	MARVELD2	PD	Wald ratio	1	-0.60	0.02452	0.55	0.32	0.93
ENSG00000261451	#N/A	PD	Wald ratio	1	-0.44	0.0246	0.65	0.44	0.95
ENSG00000096401	CDC5L	PD	Inverse variance weighted	2	0.32	0.024602	1.37	1.04	1.81
ENSG00000178209	PLEC	PD	Wald ratio	1	-0.11	0.024624	0.90	0.82	0.99
ENSG00000144724	PTPRG	PD	Wald ratio	1	-0.65	0.024646	0.52	0.30	0.92
ENSG00000162444	RBP7	PD	Inverse variance weighted	9	0.11	0.024648	1.12	1.01	1.23
ENSG00000139343	SNRPF	PD	Wald ratio	1	0.36	0.024744	1.44	1.05	1.98
ENSG00000134183	GNAT2	PD	Wald ratio	1	0.37	0.024782	1.45	1.05	2.01
ENSG00000171798	KNDC1	PD	Inverse variance weighted	4	-0.11	0.024917	0.89	0.81	0.99

ENSG00000142583	SLC2A5	PD	Inverse variance weighted	6	0.12	0.025027	1.12	1.01	1.24
ENSG00000155629	PIK3AP1	PD	Inverse variance weighted	3	-0.21	0.025041	0.81	0.68	0.97
ENSG00000197019	SERTAD1	PD	Inverse variance weighted	2	-0.16	0.025047	0.85	0.74	0.98
ENSG00000259984	#N/A	PD	Wald ratio	1	-0.39	0.025091	0.67	0.48	0.95
ENSG00000242539	#N/A	PD	Wald ratio	1	-0.67	0.02511	0.51	0.28	0.92
ENSG00000168994	PXDC1	PD	Wald ratio	1	0.36	0.02516	1.43	1.05	1.95
ENSG00000249249	#N/A	PD	Wald ratio	1	0.39	0.025183	1.48	1.05	2.07
ENSG00000226986	#N/A	PD	Wald ratio	1	-0.12	0.025344	0.89	0.80	0.99
ENSG00000228305	#N/A	PD	Wald ratio	1	-0.16	0.025344	0.86	0.75	0.98
ENSG00000167077	MEI1	PD	Inverse variance weighted	4	-0.12	0.025483	0.89	0.80	0.99
ENSG00000186141	POLR3C	PD	Wald ratio	1	-0.61	0.025503	0.55	0.32	0.93
ENSG00000171044	XKR6	PD	Wald ratio	1	0.20	0.025517	1.23	1.03	1.46
ENSG00000105656	ELL	PD	Inverse variance weighted	5	-0.07	0.025677	0.93	0.87	0.99
ENSG00000138111	MFSD13A	PD	Inverse variance weighted	5	-0.08	0.025745	0.92	0.85	0.99
ENSG00000173917	HOXB2	PD	Inverse variance weighted	11	0.05	0.025763	1.05	1.01	1.09
ENSG00000145029	NICN1	PD	Inverse variance weighted	3	-0.08	0.025766	0.92	0.86	0.99
ENSG00000129925	PGAP6	PD	Inverse variance weighted	5	0.06	0.025769	1.06	1.01	1.12
ENSG00000254691	#N/A	PD	Wald ratio	1	0.23	0.025842	1.26	1.03	1.55
ENSG00000228399	RPS20P6	PD	Inverse variance weighted	2	0.37	0.025889	1.45	1.05	2.02
ENSG00000198646	NCOA6	PD	Inverse variance weighted	3	-0.17	0.025919	0.85	0.73	0.98
ENSG00000203780	FANK1	PD	Inverse variance weighted	2	0.16	0.026146	1.17	1.02	1.35
ENSG00000238246	#N/A	PD	Wald ratio	1	0.53	0.02615	1.70	1.06	2.71
ENSG00000235888	#N/A	PD	Wald ratio	1	0.47	0.026183	1.61	1.06	2.44
ENSG00000149292	TTC12	PD	Inverse variance weighted	5	0.09	0.026211	1.09	1.01	1.18
ENSG00000146700	SSC4D	PD	Wald ratio	1	-0.23	0.026268	0.79	0.64	0.97
ENSG00000238086	PPP1R26P1	PD	Wald ratio	1	-0.41	0.02635	0.66	0.46	0.95
ENSG00000152760	DYNLT5	PD	Inverse variance weighted	2	0.09	0.026426	1.09	1.01	1.18
ENSG00000172071	EIF2AK3	PD	Wald ratio	1	0.31	0.02647	1.36	1.04	1.79
ENSG00000246375	PPM1K-DT	PD	Wald ratio	1	-0.21	0.026482	0.81	0.68	0.98
ENSG00000105559	PLEKHA4	PD	Wald ratio	1	-0.24	0.026486	0.79	0.64	0.97
ENSG00000139180	NDUFA9	PD	Wald ratio	1	0.31	0.026542	1.37	1.04	1.81
ENSG00000121281	ADCY7	PD	Inverse variance weighted	4	-0.07	0.026642	0.93	0.87	0.99
ENSG00000198276	UCKL1	PD	Inverse variance weighted	3	0.12	0.026701	1.12	1.01	1.24
ENSG00000250982	#N/A	PD	Wald ratio	1	-0.62	0.026789	0.54	0.31	0.93
ENSG00000125817	CENPB	PD	Inverse variance weighted	2	0.15	0.026965	1.16	1.02	1.32
ENSG00000165688	PMPCA	PD	Wald ratio	1	0.54	0.02697	1.72	1.06	2.79
ENSG00000122861	PLAU	PD	Inverse variance weighted	2	-0.15	0.026997	0.86	0.75	0.98
ENSG00000161547	SRSF2	PD	Wald ratio	1	-0.55	0.027052	0.58	0.36	0.94
ENSG00000109066	TMEM104	PD	Wald ratio	1	0.41	0.027069	1.50	1.05	2.16
ENSG00000257696	#N/A	PD	Wald ratio	1	0.27	0.027079	1.31	1.03	1.65
ENSG00000139597	N4BP2L1	PD	Inverse variance weighted	2	0.31	0.027192	1.36	1.04	1.80
ENSG00000258581	#N/A	PD	Wald ratio	1	-0.13	0.027193	0.87	0.78	0.99
ENSG00000148297	MED22	PD	Wald ratio	1	0.35	0.027309	1.42	1.04	1.93
ENSG00000139546	TARBP2	PD	Wald ratio	1	-0.47	0.027312	0.62	0.41	0.95

ENSG00000077420	APBB1IP	PD	Inverse variance weighted	5	-0.14	0.027368	0.87	0.76	0.98
ENSG00000163376	KBTBD8	PD	Wald ratio	1	0.41	0.027429	1.51	1.05	2.18
ENSG00000095574	IKZF5	PD	Wald ratio	1	0.51	0.027433	1.67	1.06	2.64
ENSG00000181631	P2RY13	PD	Inverse variance weighted	8	-0.12	0.027655	0.89	0.80	0.99
ENSG00000128191	DGCR8	PD	Inverse variance weighted	6	-0.07	0.027657	0.93	0.87	0.99
ENSG00000136861	CDK5RAP2	PD	Inverse variance weighted	3	-0.07	0.027722	0.93	0.88	0.99
ENSG00000166507	NDST2	PD	Inverse variance weighted	3	0.11	0.027729	1.12	1.01	1.24
ENSG00000234284	ZNF879	PD	Wald ratio	1	0.15	0.027736	1.16	1.02	1.32
ENSG00000198546	ZNF511	PD	Inverse variance weighted	2	-0.29	0.02789	0.75	0.58	0.97
ENSG00000114120	SLC25A36	PD	Wald ratio	1	0.44	0.027945	1.55	1.05	2.30
ENSG00000188283	ZNF383	PD	Wald ratio	1	0.37	0.027955	1.45	1.04	2.02
ENSG00000161956	SENP3	PD	Wald ratio	1	0.30	0.027958	1.35	1.03	1.76
ENSG00000152117	SMPD4BP	PD	Inverse variance weighted	2	0.10	0.027968	1.10	1.01	1.21
ENSG00000251215	#N/A	PD	Wald ratio	1	0.36	0.028012	1.44	1.04	1.98
ENSG00000169180	XPO6	PD	Inverse variance weighted	4	0.15	0.02802	1.16	1.02	1.33
ENSG00000233411	#N/A	PD	Wald ratio	1	0.14	0.028034	1.15	1.02	1.31
ENSG00000178149	DALRD3	PD	Wald ratio	1	0.20	0.028093	1.23	1.02	1.47
ENSG00000101236	RNF24	PD	Inverse variance weighted	4	0.13	0.028121	1.14	1.01	1.28
ENSG00000255139	#N/A	PD	Wald ratio	1	0.46	0.028163	1.59	1.05	2.41
ENSG00000228989	#N/A	PD	Inverse variance weighted	2	-0.25	0.028212	0.78	0.63	0.97
ENSG00000136206	SPDYE1	PD	Wald ratio	1	0.27	0.028256	1.32	1.03	1.68
ENSG00000148204	CRB2	PD	Wald ratio	1	0.32	0.028277	1.38	1.03	1.84
ENSG00000258794	DUX4L27	PD	Inverse variance weighted	2	0.53	0.028341	1.70	1.06	2.73
ENSG00000168522	FNTA	PD	Wald ratio	1	-0.57	0.028381	0.57	0.34	0.94
ENSG00000262943	ALOX12P2	PD	Wald ratio	1	-0.42	0.028439	0.66	0.45	0.96
ENSG00000160213	CSTB	PD	Inverse variance weighted	4	-0.07	0.028827	0.94	0.88	0.99
ENSG00000256612	CYP2B7P	PD	Inverse variance weighted	2	-0.11	0.028893	0.90	0.82	0.99
ENSG00000131788	PIAS3	PD	Wald ratio	1	-0.50	0.028969	0.61	0.39	0.95
ENSG00000225523	IGKV6D-21	PD	Inverse variance weighted	2	0.44	0.02909	1.56	1.05	2.32
ENSG00000140612	SEC11A	PD	Wald ratio	1	0.16	0.029123	1.17	1.02	1.35
ENSG00000175054	ATR	PD	Wald ratio	1	-0.56	0.029123	0.57	0.35	0.94
ENSG00000225978	#N/A	PD	Inverse variance weighted	2	-0.10	0.029162	0.91	0.83	0.99
ENSG00000189144	ZNF573	PD	Wald ratio	1	-0.10	0.029199	0.90	0.83	0.99
ENSG00000033050	ABCF2	PD	Wald ratio	1	0.13	0.029208	1.14	1.01	1.28
ENSG00000166582	CENPV	PD	Inverse variance weighted	2	0.19	0.029231	1.21	1.02	1.43
ENSG00000143147	GPR161	PD	Wald ratio	1	0.37	0.029319	1.45	1.04	2.02
ENSG00000158186	MRAS	PD	Inverse variance weighted	6	0.17	0.029335	1.18	1.02	1.38
ENSG00000100075	SLC25A1	PD	Inverse variance weighted	2	0.12	0.029336	1.13	1.01	1.26
ENSG00000172738	TMEM217	PD	Wald ratio	1	0.46	0.02934	1.58	1.05	2.40
ENSG00000141391	PRELID3A	PD	Wald ratio	1	0.30	0.029429	1.35	1.03	1.76
ENSG00000268852	#N/A	PD	Wald ratio	1	-0.37	0.029431	0.69	0.49	0.96
ENSG00000080546	SESN1	PD	Inverse variance weighted	5	-0.07	0.029493	0.93	0.87	0.99
ENSG00000211934	IGHV1-2	PD	Wald ratio	1	0.89	0.029511	2.44	1.09	5.47
ENSG00000253394	LINC00534	PD	Inverse variance weighted	2	0.23	0.029522	1.26	1.02	1.56

ENSG00000124214	STAU1	PD	Wald ratio	1	-0.29	0.029545	0.75	0.58	0.97
ENSG00000124207	CSE1L	PD	Inverse variance weighted	2	0.10	0.029626	1.11	1.01	1.21
ENSG00000144550	CPNE9	PD	Wald ratio	1	0.63	0.029748	1.88	1.06	3.32
ENSG00000005102	MEOX1	PD	Inverse variance weighted	4	-0.10	0.029873	0.91	0.83	0.99
ENSG00000163958	ZDHHC19	PD	Inverse variance weighted	7	-0.13	0.029893	0.88	0.79	0.99
ENSG00000204149	AGAP6	PD	Inverse variance weighted	2	0.08	0.030043	1.09	1.01	1.17
ENSG00000260027	HOXB7	PD	Wald ratio	1	0.31	0.030065	1.36	1.03	1.80
ENSG00000129245	FXR2	PD	Wald ratio	1	0.52	0.030086	1.67	1.05	2.67
ENSG00000198673	TAFA2	PD	Inverse variance weighted	3	0.09	0.030153	1.10	1.01	1.19
ENSG00000129472	RAB2B	PD	Inverse variance weighted	6	-0.12	0.030192	0.89	0.80	0.99
ENSG00000118900	UBN1	PD	Inverse variance weighted	4	0.23	0.030441	1.26	1.02	1.55
ENSG00000166012	TAF1D	PD	Inverse variance weighted	2	-0.22	0.030446	0.81	0.66	0.98
ENSG00000130270	ATP8B3	PD	Inverse variance weighted	2	0.16	0.030455	1.18	1.02	1.36
ENSG00000103502	CDIPT	PD	Wald ratio	1	-0.32	0.030469	0.73	0.54	0.97
ENSG00000135503	ACVR1B	PD	Wald ratio	1	0.18	0.030534	1.20	1.02	1.42
ENSG00000237851	#N/A	PD	Wald ratio	1	-0.37	0.030746	0.69	0.49	0.97
ENSG00000164406	LEAP2	PD	Wald ratio	1	-0.33	0.030813	0.72	0.54	0.97
ENSG00000181963	OR52K2	PD	Inverse variance weighted	5	-0.10	0.031034	0.91	0.83	0.99
ENSG00000109065	NAT9	PD	Wald ratio	1	-0.32	0.031162	0.73	0.54	0.97
ENSG00000241127	YAE1	PD	Wald ratio	1	0.47	0.031162	1.59	1.04	2.43
ENSG00000229715	#N/A	PD	Inverse variance weighted	3	-0.11	0.031297	0.90	0.81	0.99
ENSG00000162642	C1orf52	PD	Wald ratio	1	-0.38	0.031304	0.69	0.49	0.97
ENSG00000175970	UNC119B	PD	Inverse variance weighted	2	0.10	0.03151	1.11	1.01	1.22
ENSG00000261662	#N/A	PD	Wald ratio	1	-0.51	0.031523	0.60	0.38	0.96
ENSG00000135926	TMBIM1	PD	Inverse variance weighted	5	0.11	0.031614	1.12	1.01	1.24
ENSG00000099904	ZDHHC8	PD	Wald ratio	1	0.19	0.031614	1.21	1.02	1.45
ENSG00000198728	LDB1	PD	Wald ratio	1	-0.15	0.031713	0.86	0.75	0.99
ENSG00000182362	YBEY	PD	Inverse variance weighted	9	0.06	0.031928	1.06	1.01	1.13
ENSG00000232706	#N/A	PD	Wald ratio	1	0.63	0.031974	1.88	1.06	3.36
ENSG00000132507	EIF5A	PD	Wald ratio	1	0.12	0.032125	1.12	1.01	1.25
ENSG00000115520	COQ10B	PD	Wald ratio	1	-0.35	0.032173	0.70	0.51	0.97
ENSG00000171084	FAM86JP	PD	Wald ratio	1	0.28	0.032241	1.32	1.02	1.70
ENSG00000100029	PES1	PD	Wald ratio	1	-0.18	0.03225	0.84	0.71	0.99
ENSG00000232151	#N/A	PD	Wald ratio	1	-1.51	0.032309	0.22	0.06	0.88
ENSG00000171105	INSR	PD	Inverse variance weighted	4	0.24	0.032341	1.28	1.02	1.60
ENSG00000167107	ACSF2	PD	Wald ratio	1	-0.60	0.03238	0.55	0.32	0.95
ENSG00000234198	#N/A	PD	Wald ratio	1	-0.51	0.032485	0.60	0.38	0.96
ENSG00000151689	INPP1	PD	Inverse variance weighted	2	0.10	0.03256	1.11	1.01	1.22
ENSG00000131153	GINS2	PD	Inverse variance weighted	2	-0.12	0.032658	0.89	0.80	0.99
ENSG00000256633	#N/A	PD	Inverse variance weighted	2	-0.13	0.032671	0.88	0.78	0.99
ENSG00000197409	H3C4	PD	Inverse variance weighted	5	0.18	0.032715	1.19	1.01	1.40
ENSG00000151690	MFSD6	PD	Inverse variance weighted	6	0.06	0.032762	1.06	1.01	1.13
ENSG00000139055	ERP27	PD	Inverse variance weighted	5	0.04	0.032839	1.04	1.00	1.08
ENSG00000136147	PHF11	PD	Inverse variance weighted	2	-0.30	0.032867	0.74	0.56	0.98

ENSG00000234028	EIF2AK3-DT	PD	Wald ratio	1	0.33	0.032868	1.39	1.03	1.89
ENSG00000166164	BRD7	PD	Inverse variance weighted	4	-0.06	0.032912	0.94	0.89	1.00
ENSG00000111325	OGFOD2	PD	Wald ratio	1	-0.39	0.032949	0.68	0.47	0.97
ENSG00000251992	#N/A	PD	Inverse variance weighted	2	-0.37	0.032966	0.69	0.49	0.97
ENSG00000127838	PNKD	PD	Inverse variance weighted	11	0.07	0.033022	1.07	1.01	1.15
ENSG00000179562	GCC1	PD	Wald ratio	1	0.12	0.033187	1.13	1.01	1.26
ENSG00000125304	TM9SF2	PD	Inverse variance weighted	2	0.21	0.033302	1.23	1.02	1.49
ENSG00000204256	BRD2	PD	Inverse variance weighted	3	0.16	0.033461	1.17	1.01	1.36
ENSG00000137161	CNPY3	PD	Wald ratio	1	0.19	0.033467	1.21	1.01	1.44
ENSG00000013288	MAN2B2	PD	Inverse variance weighted	3	-0.07	0.033496	0.93	0.88	0.99
ENSG00000100138	SNU13	PD	Inverse variance weighted	2	0.11	0.033536	1.12	1.01	1.24
ENSG00000130309	COLGALT1	PD	Inverse variance weighted	3	-0.08	0.033589	0.93	0.87	0.99
ENSG00000100417	PMM1	PD	Inverse variance weighted	2	0.10	0.033646	1.11	1.01	1.22
ENSG00000108389	MTMR4	PD	Wald ratio	1	0.38	0.033699	1.46	1.03	2.08
ENSG00000251364	LOC100506258	PD	Inverse variance weighted	2	-0.15	0.033851	0.86	0.74	0.99
ENSG00000156398	SFXN2	PD	Inverse variance weighted	2	0.10	0.033967	1.10	1.01	1.21
ENSG00000182118	FAM89A	PD	Inverse variance weighted	4	0.08	0.033993	1.08	1.01	1.17
ENSG00000105968	H2AZ2	PD	Inverse variance weighted	2	0.27	0.034023	1.32	1.02	1.70
ENSG00000257924	#N/A	PD	Inverse variance weighted	2	0.18	0.034041	1.19	1.01	1.40
ENSG00000144445	KANSL1L	PD	Wald ratio	1	-0.53	0.034043	0.59	0.36	0.96
ENSG00000188641	DPYD	PD	Inverse variance weighted	5	-0.08	0.034052	0.92	0.85	0.99
ENSG00000178999	AURKB	PD	Wald ratio	1	0.17	0.034076	1.19	1.01	1.39
ENSG00000243926	#N/A	PD	Wald ratio	1	0.55	0.034106	1.74	1.04	2.90
ENSG00000050405	LIMA1	PD	Inverse variance weighted	3	-0.09	0.034167	0.91	0.84	0.99
ENSG00000137100	DCTN3	PD	Inverse variance weighted	2	-0.18	0.03421	0.83	0.71	0.99
ENSG00000170835	CEL	PD	Wald ratio	1	-0.24	0.034227	0.79	0.63	0.98
ENSG00000198933	TBKBP1	PD	Inverse variance weighted	4	0.08	0.03425	1.08	1.01	1.16
ENSG00000234009	RPL5P34	PD	Wald ratio	1	-0.47	0.034311	0.62	0.40	0.97
ENSG00000179152	TCAIM	PD	Wald ratio	1	0.36	0.034339	1.43	1.03	1.99
ENSG00000125449	ARMC7	PD	Inverse variance weighted	2	-0.19	0.034362	0.83	0.70	0.99
ENSG00000137747	TMPRSS13	PD	Inverse variance weighted	2	-0.29	0.034415	0.75	0.57	0.98
ENSG00000204922	UQCC3	PD	Wald ratio	1	0.30	0.034516	1.35	1.02	1.77
ENSG00000176171	BNIP3	PD	Wald ratio	1	-0.38	0.034575	0.68	0.48	0.97
ENSG00000145725	PPIP5K2	PD	Inverse variance weighted	4	0.07	0.034637	1.07	1.01	1.15
ENSG00000123810	B9D2	PD	Inverse variance weighted	4	0.15	0.034687	1.17	1.01	1.34
ENSG00000143622	RIT1	PD	Inverse variance weighted	2	0.12	0.034807	1.13	1.01	1.26
ENSG00000178199	ZC3H12D	PD	Wald ratio	1	0.19	0.034838	1.21	1.01	1.45
ENSG00000033327	GAB2	PD	Wald ratio	1	-0.19	0.034844	0.82	0.69	0.99
ENSG00000126353	CCR7	PD	Inverse variance weighted	5	0.19	0.034915	1.21	1.01	1.45
ENSG00000064490	RFXANK	PD	Inverse variance weighted	3	-0.10	0.034993	0.90	0.82	0.99
ENSG00000108691	CCL2	PD	Wald ratio	1	-0.58	0.03507	0.56	0.32	0.96

ENSG00000233165	#N/A	PD	Inverse variance weighted	2	-0.19	0.035141	0.83	0.69	0.99
ENSG00000232615	#N/A	PD	Wald ratio	1	-0.20	0.035185	0.82	0.68	0.99
ENSG00000217325	PRELID1P1	PD	Inverse variance weighted	2	-0.06	0.035192	0.94	0.88	1.00
ENSG00000105197	TIMM50	PD	Wald ratio	1	0.49	0.035243	1.64	1.03	2.59
ENSG00000007402	CACNA2D2	PD	Wald ratio	1	-0.27	0.035404	0.77	0.60	0.98
ENSG00000153093	ACOXL	PD	Inverse variance weighted	2	0.34	0.03542	1.41	1.02	1.94
ENSG00000186152	#N/A	PD	Inverse variance weighted	2	0.24	0.035441	1.28	1.02	1.60
ENSG00000211630	#N/A	PD	Wald ratio	1	-0.11	0.035474	0.89	0.80	0.99
ENSG00000240834	#N/A	PD	Wald ratio	1	0.19	0.035474	1.21	1.01	1.45
ENSG00000198643	FAM3D	PD	Wald ratio	1	0.17	0.035504	1.19	1.01	1.40
ENSG00000232372	#N/A	PD	Wald ratio	1	-0.44	0.035534	0.64	0.42	0.97
ENSG00000188707	ZBED6CL	PD	Inverse variance weighted	2	-0.12	0.035604	0.89	0.80	0.99
ENSG00000232686	#N/A	PD	Wald ratio	1	0.47	0.035634	1.60	1.03	2.49
ENSG00000174720	LARP7	PD	Inverse variance weighted	2	0.20	0.035653	1.22	1.01	1.47
ENSG00000153495	TEX29	PD	Wald ratio	1	0.32	0.035672	1.38	1.02	1.86
ENSG00000175066	GK5	PD	Inverse variance weighted	2	0.28	0.035745	1.32	1.02	1.71
ENSG00000166126	AMN	PD	Wald ratio	1	0.53	0.035771	1.69	1.04	2.77
ENSG00000158161	EYA3	PD	Wald ratio	1	-0.10	0.035889	0.90	0.82	0.99
ENSG00000186130	ZBTB6	PD	Wald ratio	1	0.26	0.035908	1.29	1.02	1.64
ENSG00000141574	SECTM1	PD	Inverse variance weighted	5	-0.16	0.035919	0.85	0.73	0.99
ENSG00000084754	HADHA	PD	Inverse variance weighted	2	0.10	0.035931	1.11	1.01	1.22
ENSG00000164713	BRI3	PD	Inverse variance weighted	3	-0.13	0.036178	0.88	0.78	0.99
ENSG00000100804	PSMB5	PD	Inverse variance weighted	3	-0.12	0.036188	0.89	0.79	0.99
ENSG00000229539	#N/A	PD	Wald ratio	1	-0.19	0.036246	0.83	0.69	0.99
ENSG00000187037	GPR141	PD	Inverse variance weighted	3	-0.12	0.03628	0.89	0.79	0.99
ENSG00000141526	SLC16A3	PD	Inverse variance weighted	3	-0.08	0.036355	0.92	0.85	0.99
ENSG00000227370	#N/A	PD	Inverse variance weighted	2	0.19	0.036392	1.20	1.01	1.43
ENSG00000172346	CSDC2	PD	Wald ratio	1	-0.26	0.036424	0.77	0.61	0.98
ENSG00000160404	TOR2A	PD	Inverse variance weighted	2	0.25	0.036448	1.29	1.02	1.63
ENSG00000136159	NUDT15	PD	Inverse variance weighted	2	-0.48	0.036461	0.62	0.40	0.97
ENSG00000100644	HIF1A	PD	Inverse variance weighted	4	-0.16	0.036483	0.85	0.73	0.99
ENSG00000267244	LOC100288123	PD	Wald ratio	1	-0.14	0.036536	0.87	0.76	0.99
ENSG00000255045	#N/A	PD	Wald ratio	1	-0.51	0.036658	0.60	0.37	0.97
ENSG00000143452	HORMAD1	PD	Inverse variance weighted	2	-0.10	0.036707	0.91	0.83	0.99
ENSG00000259143	#N/A	PD	Inverse variance weighted	4	-0.16	0.036717	0.85	0.74	0.99
ENSG00000230189	#N/A	PD	Inverse variance weighted	2	-0.26	0.036735	0.77	0.61	0.98
ENSG00000198130	HIBCH	PD	Inverse variance weighted	4	-0.08	0.036751	0.92	0.85	0.99
ENSG00000204619	PPP1R11	PD	Inverse variance weighted	2	0.19	0.036859	1.21	1.01	1.46
ENSG00000168386	FILIP1L	PD	Wald ratio	1	-0.56	0.036921	0.57	0.34	0.97
ENSG00000126787	DLGAP5	PD	Wald ratio	1	-0.39	0.036935	0.68	0.47	0.98
ENSG00000138074	SLC5A6	PD	Inverse variance weighted	4	-0.07	0.036965	0.93	0.87	1.00
ENSG00000213967	ZNF726	PD	Wald ratio	1	0.56	0.037175	1.75	1.03	2.97

ENSG00000260186	#N/A	PD	Wald ratio	1	-0.73	0.037191	0.48	0.24	0.96
ENSG00000149809	TM7SF2	PD	Inverse variance weighted	2	0.12	0.037272	1.13	1.01	1.27
ENSG00000121413	ZSCAN18	PD	Inverse variance weighted	4	0.09	0.037292	1.09	1.01	1.19
ENSG00000146530	VWDE	PD	Inverse variance weighted	5	0.06	0.037298	1.06	1.00	1.13
ENSG00000133256	PDE6B	PD	Wald ratio	1	0.15	0.037303	1.16	1.01	1.34
ENSG00000166889	PATL1	PD	Wald ratio	1	-0.27	0.03738	0.76	0.59	0.98
ENSG00000250786	SNHG18	PD	Inverse variance weighted	2	0.29	0.037386	1.33	1.02	1.75
ENSG00000262944	#N/A	PD	Wald ratio	1	0.45	0.037435	1.57	1.03	2.40
ENSG00000166037	CEP57	PD	Wald ratio	1	-0.09	0.037526	0.92	0.84	0.99
ENSG00000163820	FYCO1	PD	Inverse variance weighted	2	-0.15	0.037584	0.86	0.74	0.99
ENSG00000152904	GGPS1	PD	Inverse variance weighted	3	0.10	0.037607	1.10	1.01	1.21
ENSG00000197723	#N/A	PD	Wald ratio	1	0.47	0.037622	1.60	1.03	2.49
ENSG00000250548	LINC01303	PD	Wald ratio	1	0.18	0.037675	1.19	1.01	1.41
ENSG00000132286	TIMM10B	PD	Wald ratio	1	0.16	0.03773	1.18	1.01	1.37
ENSG00000215458	AATBC	PD	Inverse variance weighted	3	-0.09	0.038017	0.91	0.84	1.00
ENSG00000010322	NISCH	PD	Wald ratio	1	-0.32	0.038092	0.73	0.54	0.98
ENSG00000153214	TMEM87B	PD	Inverse variance weighted	4	-0.10	0.038124	0.90	0.82	0.99
ENSG00000237927	#N/A	PD	Inverse variance weighted	2	0.11	0.038214	1.12	1.01	1.25
ENSG00000187474	FPR3	PD	Inverse variance weighted	6	0.11	0.038227	1.11	1.01	1.23
ENSG00000150456	EEF1AKMT1	PD	Wald ratio	1	-0.32	0.038319	0.73	0.54	0.98
ENSG00000106948	AKNA	PD	Inverse variance weighted	2	0.12	0.038351	1.13	1.01	1.27
ENSG00000114631	PODXL2	PD	Inverse variance weighted	4	-0.12	0.038432	0.89	0.80	0.99
ENSG00000132514	CLEC10A	PD	Inverse variance weighted	8	-0.07	0.038435	0.94	0.88	1.00
ENSG00000167286	CD3D	PD	Inverse variance weighted	4	-0.18	0.038457	0.84	0.71	0.99
ENSG00000132906	CASP9	PD	Inverse variance weighted	3	-0.08	0.038606	0.93	0.86	1.00
ENSG00000204390	HSPA1L	PD	Inverse variance weighted	3	-0.15	0.038617	0.86	0.75	0.99
ENSG00000267344	#N/A	PD	Wald ratio	1	0.21	0.038634	1.23	1.01	1.50
ENSG00000132383	RPA1	PD	Inverse variance weighted	5	-0.08	0.038745	0.93	0.86	1.00
ENSG00000163923	RPL39L	PD	Wald ratio	1	-0.20	0.038766	0.82	0.68	0.99
ENSG00000170037	CNTROB	PD	Wald ratio	1	0.59	0.038812	1.81	1.03	3.18
ENSG00000143401	ANP32E	PD	Wald ratio	1	-0.22	0.038822	0.81	0.66	0.99
ENSG00000196917	HCAR1	PD	Wald ratio	1	-0.14	0.038835	0.87	0.76	0.99
ENSG00000147650	LRP12	PD	Wald ratio	1	0.19	0.038982	1.20	1.01	1.44
ENSG00000226508	LINC01918	PD	Wald ratio	1	0.68	0.038993	1.98	1.04	3.79
ENSG00000204590	GNL1	PD	Wald ratio	1	-0.46	0.039034	0.63	0.41	0.98
ENSG00000128944	KNSTRN	PD	Inverse variance weighted	3	-0.08	0.039048	0.92	0.86	1.00
ENSG00000163882	POLR2H	PD	Wald ratio	1	-0.32	0.039131	0.72	0.53	0.98
ENSG00000260467	#N/A	PD	Inverse variance weighted	2	-0.23	0.039282	0.80	0.64	0.99
ENSG00000238975	#N/A	PD	Wald ratio	1	-0.50	0.039341	0.61	0.38	0.98
ENSG00000231890	DARS1-AS1	PD	Wald ratio	1	0.21	0.03939	1.23	1.01	1.50
ENSG00000176155	CCDC57	PD	Wald ratio	1	-0.08	0.039511	0.92	0.85	1.00
ENSG00000170322	NFRKB	PD	Inverse variance weighted	5	0.16	0.039613	1.17	1.01	1.36

ENSG00000150459	SAP18	PD	Wald ratio	1	-0.22	0.039658	0.80	0.65	0.99
ENSG00000141002	TCF25	PD	Wald ratio	1	-0.40	0.039672	0.67	0.45	0.98
ENSG00000248925	#N/A	PD	Wald ratio	1	-0.21	0.039732	0.81	0.66	0.99
ENSG00000223959	AFG3L1P	PD	Inverse variance weighted	3	0.14	0.039743	1.15	1.01	1.31
ENSG00000167904	TMEM68	PD	Wald ratio	1	0.23	0.039909	1.26	1.01	1.58
ENSG00000229961	#N/A	PD	Inverse variance weighted	2	0.41	0.039909	1.51	1.02	2.23
ENSG00000178295	GEN1	PD	Wald ratio	1	-0.16	0.040017	0.85	0.73	0.99
ENSG00000136856	SLC2A8	PD	Inverse variance weighted	2	0.08	0.040109	1.08	1.00	1.16
ENSG00000151715	TMEM45B	PD	Inverse variance weighted	5	0.06	0.040128	1.06	1.00	1.12
ENSG00000130244	FAM98C	PD	Inverse variance weighted	2	0.17	0.040181	1.18	1.01	1.39
ENSG00000181450	ZNF678	PD	Wald ratio	1	-0.54	0.040188	0.58	0.35	0.98
ENSG00000261542	#N/A	PD	Inverse variance weighted	3	-0.35	0.040215	0.71	0.51	0.98
ENSG00000142039	CCDC97	PD	Inverse variance weighted	2	0.31	0.040249	1.37	1.01	1.84
ENSG00000259418	#N/A	PD	Wald ratio	1	0.46	0.040312	1.59	1.02	2.48
ENSG00000175161	CADM2	PD	Wald ratio	1	0.48	0.04034	1.61	1.02	2.54
ENSG00000269397	#N/A	PD	Wald ratio	1	0.68	0.040364	1.97	1.03	3.78
ENSG00000147799	ARHGAP39	PD	Wald ratio	1	0.22	0.040391	1.25	1.01	1.55
ENSG00000229127	#N/A	PD	Wald ratio	1	-0.28	0.040418	0.76	0.58	0.99
ENSG00000197279	ZNF165	PD	Inverse variance weighted	3	0.22	0.040485	1.24	1.01	1.53
ENSG00000160214	RRP1	PD	Inverse variance weighted	2	-0.22	0.040511	0.80	0.65	0.99
ENSG00000135655	USP15	PD	Inverse variance weighted	2	0.11	0.040546	1.12	1.00	1.24
ENSG00000198839	ZNF277	PD	Wald ratio	1	0.51	0.040636	1.67	1.02	2.72
ENSG00000111912	NCOA7	PD	Inverse variance weighted	2	-0.11	0.040688	0.89	0.80	1.00
ENSG00000170425	ADORA2B	PD	Inverse variance weighted	2	0.19	0.040729	1.21	1.01	1.46
ENSG00000198156	NPIPB6	PD	Inverse variance weighted	2	-0.26	0.040799	0.77	0.60	0.99
ENSG00000169710	FASN	PD	Wald ratio	1	0.10	0.040835	1.11	1.00	1.22
ENSG00000129194	SOX15	PD	Wald ratio	1	-0.38	0.04086	0.68	0.47	0.98
ENSG00000129255	MPDU1	PD	Wald ratio	1	0.18	0.04086	1.19	1.01	1.41
ENSG00000226403	#N/A	PD	Inverse variance weighted	2	-0.31	0.040971	0.73	0.55	0.99
ENSG00000168734	PKIG	PD	Wald ratio	1	0.16	0.041022	1.18	1.01	1.38
ENSG00000166896	ATP23	PD	Inverse variance weighted	4	0.10	0.041031	1.11	1.00	1.22
ENSG00000222267	RNU6-892P	PD	Wald ratio	1	0.23	0.041036	1.25	1.01	1.56
ENSG00000241106	HLA-DOB	PD	Inverse variance weighted	4	-0.08	0.041093	0.93	0.86	1.00
ENSG00000165102	HGSNAT	PD	Inverse variance weighted	2	0.36	0.041143	1.43	1.01	2.01
ENSG00000214413	BBIP1	PD	Inverse variance weighted	2	0.26	0.041211	1.30	1.01	1.66
ENSG00000153922	CHD1	PD	Wald ratio	1	0.60	0.041225	1.82	1.02	3.25
ENSG00000033627	ATP6V0A1	PD	Inverse variance weighted	4	-0.16	0.041236	0.85	0.73	0.99
ENSG00000185432	METTL7A	PD	Inverse variance weighted	3	0.09	0.041311	1.09	1.00	1.19
ENSG00000186777	ZNF732	PD	Wald ratio	1	0.75	0.041334	2.12	1.03	4.35
ENSG00000160783	PMF1	PD	Inverse variance weighted	2	0.22	0.041351	1.24	1.01	1.54
ENSG00000181511	#N/A	PD	Wald ratio	1	0.27	0.041444	1.31	1.01	1.69
ENSG00000166704	ZNF606	PD	Inverse variance weighted	3	0.17	0.041728	1.19	1.01	1.40
ENSG00000117318	ID3	PD	Inverse variance weighted	2	-0.22	0.041733	0.80	0.65	0.99

ENSG00000268230	#N/A	PD	Wald ratio	1	-0.27	0.041788	0.77	0.59	0.99
ENSG00000075391	RASAL2	PD	Wald ratio	1	0.48	0.041807	1.62	1.02	2.59
ENSG00000269001	#N/A	PD	Inverse variance weighted	2	-0.22	0.041814	0.80	0.65	0.99
ENSG00000124713	GNMT	PD	Wald ratio	1	-0.08	0.041842	0.92	0.85	1.00
ENSG00000163617	CCDC191	PD	Wald ratio	1	0.17	0.041886	1.19	1.01	1.40
ENSG00000264964	#N/A	PD	Wald ratio	1	0.28	0.041888	1.32	1.01	1.73
ENSG00000131686	CA6	PD	Inverse variance weighted	2	-0.11	0.041924	0.90	0.81	1.00
ENSG00000198848	CES1	PD	Inverse variance weighted	5	-0.13	0.041958	0.88	0.77	1.00
ENSG00000054267	ARID4B	PD	Wald ratio	1	0.30	0.042056	1.35	1.01	1.82
ENSG00000197982	C1orf122	PD	Wald ratio	1	0.10	0.042128	1.11	1.00	1.23
ENSG00000107736	CDH23	PD	Inverse variance weighted	8	-0.05	0.042245	0.95	0.91	1.00
ENSG00000172164	SNTB1	PD	Inverse variance weighted	4	0.18	0.042371	1.20	1.01	1.43
ENSG00000165406	MARCHF8	PD	Inverse variance weighted	3	0.15	0.042394	1.16	1.01	1.33
ENSG00000254946	LINC02751	PD	Wald ratio	1	-0.51	0.042502	0.60	0.37	0.98
ENSG00000137815	RTF1	PD	Wald ratio	1	-0.14	0.042535	0.87	0.75	1.00
ENSG00000198483	ANKRD35	PD	Wald ratio	1	-0.08	0.042535	0.92	0.85	1.00
ENSG00000236778	INTS6-AS1	PD	Inverse variance weighted	2	-0.16	0.042621	0.85	0.73	0.99
ENSG00000128951	DUT	PD	Inverse variance weighted	4	-0.10	0.042644	0.90	0.82	1.00
ENSG00000136274	NACAD	PD	Inverse variance weighted	2	0.17	0.042669	1.19	1.01	1.40
ENSG00000186940	#N/A	PD	Wald ratio	1	-0.21	0.042684	0.81	0.66	0.99
ENSG00000215006	#N/A	PD	Wald ratio	1	-0.35	0.042684	0.70	0.50	0.99
ENSG00000140988	RPS2	PD	Wald ratio	1	0.37	0.042722	1.44	1.01	2.06
ENSG00000140990	NDUFB10	PD	Wald ratio	1	-0.17	0.042722	0.84	0.71	0.99
ENSG00000164244	PRRC1	PD	Inverse variance weighted	2	-0.15	0.042801	0.86	0.75	1.00
ENSG00000139291	TMEM19	PD	Inverse variance weighted	2	-0.21	0.042955	0.81	0.66	0.99
ENSG00000184304	PRKD1	PD	Inverse variance weighted	2	0.06	0.043019	1.06	1.00	1.12
ENSG00000114353	GNAI2	PD	Wald ratio	1	-0.37	0.043023	0.69	0.48	0.99
ENSG00000005206	SPPL2B	PD	Wald ratio	1	-0.44	0.043124	0.64	0.42	0.99
ENSG00000142798	HSPG2	PD	Inverse variance weighted	3	0.13	0.04314	1.14	1.00	1.30
ENSG00000182919	C11orf54	PD	Inverse variance weighted	2	0.09	0.043171	1.09	1.00	1.19
ENSG00000105229	PIAS4	PD	Wald ratio	1	0.18	0.043179	1.19	1.01	1.41
ENSG00000165633	VSTM4	PD	Wald ratio	1	0.59	0.043279	1.81	1.02	3.21
ENSG00000164011	ZNF691	PD	Wald ratio	1	0.51	0.043297	1.67	1.02	2.74
ENSG00000125810	CD93	PD	Inverse variance weighted	12	0.07	0.043387	1.07	1.00	1.14
ENSG00000187239	FNBP1	PD	Inverse variance weighted	2	0.09	0.043418	1.09	1.00	1.19
ENSG00000163386	#N/A	PD	Inverse variance weighted	2	-0.37	0.043559	0.69	0.48	0.99
ENSG00000235241	#N/A	PD	Wald ratio	1	-0.37	0.043589	0.69	0.48	0.99
ENSG00000184811	TRARG1	PD	Wald ratio	1	-0.29	0.043663	0.75	0.56	0.99
ENSG00000103723	AP3B2	PD	Inverse variance weighted	2	-0.09	0.043669	0.91	0.83	1.00
ENSG00000143761	ARF1	PD	Wald ratio	1	-0.41	0.04367	0.67	0.45	0.99
ENSG00000198574	SH2D1B	PD	Inverse variance weighted	7	0.16	0.043756	1.17	1.00	1.36
ENSG00000188786	MTF1	PD	Wald ratio	1	0.25	0.043777	1.28	1.01	1.63
ENSG00000143751	SDE2	PD	Inverse variance weighted	2	-0.28	0.043802	0.76	0.58	0.99
ENSG00000151466	SCLT1	PD	Inverse variance weighted	3	0.15	0.04391	1.17	1.00	1.36

ENSG00000249601	#N/A	PD	Inverse variance weighted	3	-0.21	0.043936	0.81	0.66	0.99
ENSG00000231234	#N/A	PD	Wald ratio	1	-0.40	0.044146	0.67	0.45	0.99
ENSG00000254685	FPGT	PD	Inverse variance weighted	2	-0.09	0.044243	0.91	0.83	1.00
ENSG00000165630	PRPF18	PD	Wald ratio	1	0.32	0.044256	1.38	1.01	1.88
ENSG00000243667	DNAAF10	PD	Wald ratio	1	0.14	0.044287	1.15	1.00	1.31
ENSG00000251453	#N/A	PD	Wald ratio	1	0.25	0.044287	1.29	1.01	1.65
ENSG00000157617	C2CD2	PD	Inverse variance weighted	2	-0.20	0.04441	0.82	0.68	1.00
ENSG00000188848	BEND4	PD	Wald ratio	1	0.41	0.04454	1.51	1.01	2.25
ENSG00000206965	RNU6-5P	PD	Wald ratio	1	-0.53	0.044608	0.59	0.35	0.99
ENSG00000188603	CLN3	PD	Inverse variance weighted	2	0.13	0.044628	1.14	1.00	1.30
ENSG00000203602	#N/A	PD	Wald ratio	1	0.29	0.045027	1.34	1.01	1.78
ENSG00000128482	RNF112	PD	Wald ratio	1	-0.11	0.045058	0.90	0.81	1.00
ENSG00000166484	MAPK7	PD	Wald ratio	1	0.25	0.045058	1.28	1.01	1.63
ENSG00000269969	#N/A	PD	Inverse variance weighted	2	0.34	0.045068	1.40	1.01	1.94
ENSG00000188739	RBM34	PD	Wald ratio	1	-0.42	0.04509	0.66	0.44	0.99
ENSG00000170153	RNF150	PD	Inverse variance weighted	6	-0.05	0.045211	0.95	0.90	1.00
ENSG00000159840	ZYX	PD	Inverse variance weighted	5	-0.06	0.045237	0.94	0.89	1.00
ENSG00000197037	ZSCAN25	PD	Wald ratio	1	-0.28	0.045316	0.76	0.58	0.99
ENSG00000125818	PSMF1	PD	Inverse variance weighted	7	0.10	0.045433	1.11	1.00	1.22
ENSG00000260698	#N/A	PD	Wald ratio	1	-0.36	0.0455	0.70	0.49	0.99
ENSG00000139926	FRMD6	PD	Inverse variance weighted	2	0.27	0.04564	1.31	1.01	1.70
ENSG00000160588	MPZL3	PD	Inverse variance weighted	3	-0.10	0.045662	0.91	0.82	1.00
ENSG00000064225	ST3GAL6	PD	Inverse variance weighted	4	0.08	0.045679	1.08	1.00	1.17
ENSG00000137992	DBT	PD	Wald ratio	1	0.80	0.04575	2.22	1.02	4.86
ENSG00000136643	RPS6KC1	PD	Wald ratio	1	-0.31	0.045954	0.73	0.54	0.99
ENSG00000136938	ANP32B	PD	Wald ratio	1	-0.21	0.04603	0.81	0.66	1.00
ENSG00000168282	MGAT2	PD	Wald ratio	1	-0.17	0.046051	0.84	0.71	1.00
ENSG00000125388	GRK4	PD	Inverse variance weighted	2	-0.07	0.046065	0.93	0.87	1.00
ENSG00000205038	PKHD1L1	PD	Inverse variance weighted	5	0.10	0.046247	1.11	1.00	1.22
ENSG00000240399	#N/A	PD	Inverse variance weighted	2	0.09	0.046269	1.09	1.00	1.19
ENSG00000158435	CNOT11	PD	Inverse variance weighted	2	0.19	0.046301	1.21	1.00	1.46
ENSG00000248019	FAM13A-AS1	PD	Inverse variance weighted	3	-0.13	0.046418	0.87	0.77	1.00
ENSG00000171867	PRNP	PD	Inverse variance weighted	2	-0.16	0.046602	0.85	0.73	1.00
ENSG00000259488	#N/A	PD	Inverse variance weighted	2	-0.19	0.046716	0.83	0.68	1.00
ENSG00000223825	#N/A	PD	Wald ratio	1	-0.39	0.046741	0.67	0.46	0.99
ENSG00000157014	TATDN2	PD	Inverse variance weighted	3	0.08	0.046844	1.08	1.00	1.17
ENSG00000197880	MDS2	PD	Inverse variance weighted	2	0.22	0.047297	1.25	1.00	1.56
ENSG00000177606	JUN	PD	Inverse variance weighted	8	0.11	0.047372	1.11	1.00	1.23
ENSG00000135842	NIBAN1	PD	Inverse variance weighted	4	-0.11	0.047374	0.89	0.80	1.00
ENSG00000211807	TRAV26-1	PD	Inverse variance weighted	2	0.27	0.047464	1.31	1.00	1.72
ENSG00000174446	SNAPC5	PD	Inverse variance weighted	3	-0.16	0.047506	0.85	0.73	1.00
ENSG00000247199	FBXO38-DT	PD	Wald ratio	1	0.38	0.047749	1.46	1.00	2.12

ENSG00000134255	CEPT1	PD	Inverse variance weighted	4	0.15	0.04781	1.16	1.00	1.35
ENSG00000145416	MARCHF1	PD	Inverse variance weighted	4	0.07	0.047917	1.08	1.00	1.16
ENSG00000211637	IGLV4-69	PD	Wald ratio	1	-0.28	0.048125	0.75	0.57	1.00
ENSG00000211648	IGLV1-47	PD	Wald ratio	1	-0.57	0.048125	0.57	0.32	1.00
ENSG00000254030	IGLC5	PD	Wald ratio	1	0.22	0.048125	1.25	1.00	1.55
ENSG00000159713	TPPP3	PD	Inverse variance weighted	9	0.10	0.048147	1.11	1.00	1.23
ENSG00000204574	ABCF1	PD	Inverse variance weighted	2	-0.17	0.048151	0.85	0.72	1.00
ENSG00000151490	PTPRO	PD	Inverse variance weighted	2	0.24	0.048206	1.27	1.00	1.62
ENSG00000153786	ZDHHC7	PD	Inverse variance weighted	7	-0.06	0.048283	0.94	0.89	1.00
ENSG00000125454	SLC25A19	PD	Inverse variance weighted	2	0.25	0.048361	1.29	1.00	1.66
ENSG00000143353	LYPLAL1	PD	Inverse variance weighted	2	0.11	0.04846	1.12	1.00	1.24
ENSG00000135686	KLHL36	PD	Inverse variance weighted	2	0.08	0.048473	1.08	1.00	1.16
ENSG00000140284	SLC27A2	PD	Wald ratio	1	-0.21	0.048482	0.81	0.66	1.00
ENSG00000218186	KRT8P43	PD	Wald ratio	1	-0.46	0.048485	0.63	0.40	1.00
ENSG00000169251	NMD3	PD	Wald ratio	1	-0.12	0.048515	0.88	0.78	1.00
ENSG00000178718	RPP25	PD	Wald ratio	1	-0.46	0.048652	0.63	0.40	1.00
ENSG00000187796	CARD9	PD	Inverse variance weighted	6	0.06	0.048683	1.06	1.00	1.13
ENSG00000178252	WDR6	PD	Inverse variance weighted	4	-0.06	0.048718	0.95	0.89	1.00
ENSG00000135245	HILPDA	PD	Wald ratio	1	0.36	0.048788	1.43	1.00	2.05
ENSG00000232053	#N/A	PD	Wald ratio	1	0.38	0.048789	1.46	1.00	2.12
ENSG00000180747	SMG1P3	PD	Inverse variance weighted	4	0.38	0.048999	1.46	1.00	2.14
ENSG00000100802	C14orf93	PD	Inverse variance weighted	2	-0.31	0.04916	0.74	0.54	1.00
ENSG00000204791	#N/A	PD	Wald ratio	1	-0.19	0.049222	0.83	0.69	1.00
ENSG00000004975	DVL2	PD	Wald ratio	1	-0.20	0.049309	0.82	0.67	1.00
ENSG00000170502	NUDT9	PD	Inverse variance weighted	2	-0.16	0.04931	0.85	0.72	1.00
ENSG00000246596	#N/A	PD	Wald ratio	1	-0.52	0.049386	0.60	0.36	1.00
ENSG00000203760	CENPW	PD	Wald ratio	1	-0.28	0.049401	0.75	0.57	1.00
ENSG00000111254	AKAP3	PD	Wald ratio	1	-0.14	0.049435	0.87	0.75	1.00
ENSG00000232112	TMA7	PD	Wald ratio	1	0.22	0.049472	1.25	1.00	1.55
ENSG00000101447	FAM83D	PD	Wald ratio	1	0.21	0.049557	1.23	1.00	1.52
ENSG00000163171	CDC42EP3	PD	Inverse variance weighted	4	0.12	0.049647	1.13	1.00	1.28
ENSG00000143952	VPS54	PD	Inverse variance weighted	2	0.30	0.049665	1.35	1.00	1.83
ENSG00000137462	TLR2	PD	Inverse variance weighted	3	0.15	0.049752	1.16	1.00	1.35
ENSG00000234477	#N/A	PD	Wald ratio	1	0.13	0.049792	1.14	1.00	1.31
ENSG00000255328	#N/A	PD	Inverse variance weighted	6	-0.07	0.049805	0.93	0.87	1.00
ENSG00000115486	GGCX	PD	Inverse variance weighted	2	-0.07	0.049836	0.93	0.86	1.00
ENSG00000248367	#N/A	PD	Inverse variance weighted	2	0.13	0.049848	1.14	1.00	1.30
ENSG00000269932	#N/A	PD	Inverse variance weighted	2	0.42	0.04995	1.51	1.00	2.29

Supplementary Table 6: Phenome-wide MR results for brain CD38.

outcome	exposure	method	nsnp	b	se	pval	or	or_lci95	or_uci95
Other acute and subacute forms of ischemic heart disease	CD38	Wald ratio	1	1.34	0.38	3.99E-04	3.83	1.82	8.06
Other disorders of gallbladder	CD38	Wald ratio	1	-1.21	0.34	4.65E-04	0.30	0.15	0.59
Chronic renal failure [CKD]	CD38	Wald ratio	1	0.86	0.25	7.16E-04	2.36	1.43	3.87
Ovarian cyst	CD38	Wald ratio	1	0.60	0.19	1.65E-03	1.82	1.25	2.64
Other specified cardiac dysrhythmias	CD38	Wald ratio	1	0.69	0.23	2.63E-03	1.99	1.27	3.11
Poisoning by anticonvulsants and anti-Parkinsonism drugs	CD38	Wald ratio	1	-1.71	0.57	0.003	0.18	0.06	0.56
Sleep disorders	CD38	Wald ratio	1	0.49	0.18	0.006	1.64	1.15	2.34
Anaphylactic shock NOS	CD38	Wald ratio	1	1.44	0.55	0.009	4.23	1.43	12.50
Diseases and other conditions of the tongue	CD38	Wald ratio	1	0.95	0.37	0.010	2.59	1.26	5.34
Sleep apnea	CD38	Wald ratio	1	0.50	0.20	0.010	1.66	1.13	2.43
Hypothyroidism	CD38	Wald ratio	1	0.28	0.11	0.011	1.33	1.07	1.66
Noninflammatory disorders of ovary, fallopian tube, and broad ligament	CD38	Wald ratio	1	1.19	0.48	0.012	3.30	1.29	8.43
Known or suspected fetal abnormality affecting management of mother	CD38	Wald ratio	1	-0.51	0.21	0.017	0.60	0.40	0.91
Sciatica	CD38	Wald ratio	1	0.64	0.27	0.017	1.89	1.12	3.18
Disorders of sweat glands	CD38	Wald ratio	1	-1.07	0.45	0.018	0.34	0.14	0.83
Hypothyroidism NOS	CD38	Wald ratio	1	0.27	0.12	0.018	1.31	1.05	1.65
Infertility, female	CD38	Wald ratio	1	-0.83	0.36	0.021	0.44	0.22	0.88
Other local infections of skin and subcutaneous tissue	CD38	Wald ratio	1	-0.44	0.19	0.021	0.64	0.44	0.94
Carbuncle and furuncle	CD38	Wald ratio	1	-0.62	0.27	0.022	0.54	0.32	0.91
Other headache syndromes	CD38	Wald ratio	1	0.34	0.15	0.022	1.40	1.05	1.87
Cervical cancer	CD38	Wald ratio	1	0.73	0.32	0.022	2.07	1.11	3.88

Symptoms involving nervous and musculoskeletal systems	CD38	Wald ratio	1	0.68	0.30	0.024	1.96	1.09	3.53
Peritoneal or intestinal adhesions	CD38	Wald ratio	1	1.01	0.45	0.024	2.74	1.14	6.58
Abnormal heart sounds	CD38	Wald ratio	1	0.89	0.40	0.025	2.45	1.12	5.35
Irregular menstrual cycle/bleeding	CD38	Wald ratio	1	0.25	0.11	0.025	1.28	1.03	1.59
Occlusion and stenosis of precerebral arteries	CD38	Wald ratio	1	-0.83	0.37	0.026	0.43	0.21	0.91
Psoriatic arthropathy	CD38	Wald ratio	1	1.07	0.48	0.027	2.92	1.13	7.54
Uterine/Uterovaginal prolapse	CD38	Wald ratio	1	0.39	0.18	0.030	1.48	1.04	2.10
Cerebral ischemia	CD38	Wald ratio	1	0.51	0.24	0.033	1.67	1.04	2.68
Symptoms affecting skin	CD38	Wald ratio	1	0.37	0.17	0.034	1.44	1.03	2.03
Stiffness of joint	CD38	Wald ratio	1	1.10	0.52	0.035	3.00	1.08	8.31
Phobia	CD38	Wald ratio	1	1.21	0.58	0.036	3.34	1.08	10.32
Retention of urine	CD38	Wald ratio	1	0.34	0.16	0.036	1.40	1.02	1.92
Adverse effects of sedatives or other central nervous system depressants and anesthetics	CD38	Wald ratio	1	1.12	0.53	0.036	3.05	1.07	8.67
Umbilical hernia	CD38	Wald ratio	1	0.45	0.21	0.037	1.56	1.03	2.38
Ectropion or entropion	CD38	Wald ratio	1	0.82	0.40	0.039	2.27	1.04	4.94
Open wounds of head; neck; and trunk	CD38	Wald ratio	1	0.43	0.21	0.039	1.54	1.02	2.32
Renal failure	CD38	Wald ratio	1	0.32	0.16	0.041	1.37	1.01	1.86
Memory loss	CD38	Wald ratio	1	0.99	0.49	0.043	2.69	1.03	7.01
Hyperhidrosis	CD38	Wald ratio	1	-1.07	0.53	0.043	0.34	0.12	0.97
Contracture of palmar fascia [Dupuytren's disease]	CD38	Wald ratio	1	-0.45	0.22	0.043	0.64	0.41	0.99
GERD	CD38	Wald ratio	1	0.22	0.11	0.045	1.25	1.01	1.56
Tachycardia NOS	CD38	Wald ratio	1	0.55	0.28	0.048	1.73	1.01	2.98
Noninflammatory disorders of cervix	CD38	Wald ratio	1	-0.48	0.24	0.048	0.62	0.38	1.00
Other biliary tract disease	CD38	Wald ratio	1	-0.41	0.21	0.049	0.66	0.44	1.00
Cystitis and urethritis	CD38	Wald ratio	1	0.45	0.23	0.053	1.57	0.99	2.49

Congestive heart failure; nonhypertensive	CD38	Wald ratio	1	0.34	0.18	0.054	1.41	0.99	2.00
Hypopotassemia	CD38	Wald ratio	1	0.66	0.34	0.055	1.93	0.99	3.76
Disturbance of skin sensation	CD38	Wald ratio	1	0.46	0.24	0.055	1.58	0.99	2.53
Otitis media and Eustachian tube disorders	CD38	Wald ratio	1	-0.52	0.27	0.056	0.59	0.35	1.01
Hyperlipidemia	CD38	Wald ratio	1	0.15	0.08	0.056	1.16	1.00	1.34
Diseases of esophagus	CD38	Wald ratio	1	0.14	0.07	0.057	1.15	1.00	1.33
Cystitis	CD38	Wald ratio	1	0.45	0.24	0.058	1.57	0.98	2.52
Precordial pain	CD38	Wald ratio	1	-0.41	0.22	0.060	0.66	0.43	1.02
Disorders of iron metabolism	CD38	Wald ratio	1	0.93	0.50	0.061	2.55	0.96	6.76
Disorders of lipoid metabolism	CD38	Wald ratio	1	0.14	0.08	0.061	1.15	0.99	1.34
Blindness and low vision	CD38	Wald ratio	1	0.89	0.48	0.063	2.44	0.95	6.26
Injury, NOS	CD38	Wald ratio	1	0.26	0.14	0.066	1.30	0.98	1.72
Osteoarthrosis, generalized	CD38	Wald ratio	1	0.97	0.53	0.066	2.64	0.94	7.45
Phlebitis and thrombophlebitis	CD38	Wald ratio	1	0.38	0.21	0.068	1.47	0.97	2.21
Disorders of muscle, ligament, and fascia	CD38	Wald ratio	1	-0.36	0.20	0.068	0.70	0.47	1.03
Normal delivery	CD38	Wald ratio	1	-0.55	0.30	0.068	0.58	0.32	1.04
Asthma	CD38	Wald ratio	1	0.16	0.09	0.069	1.17	0.99	1.38
Viral hepatitis	CD38	Wald ratio	1	0.68	0.37	0.069	1.96	0.95	4.07
Hypertensive chronic kidney disease	CD38	Wald ratio	1	0.60	0.33	0.069	1.82	0.95	3.48
Irregular menstrual cycle	CD38	Wald ratio	1	0.52	0.29	0.072	1.69	0.95	2.99
Late effects of cerebrovascular disease	CD38	Wald ratio	1	0.65	0.36	0.075	1.91	0.94	3.90
Heart failure NOS	CD38	Wald ratio	1	0.36	0.20	0.075	1.43	0.96	2.12
Fever of unknown origin	CD38	Wald ratio	1	-0.37	0.21	0.076	0.69	0.46	1.04
Bronchitis	CD38	Wald ratio	1	0.91	0.51	0.078	2.48	0.90	6.77
Hypercholesterolemia	CD38	Wald ratio	1	0.14	0.08	0.079	1.15	0.98	1.34
Fracture of neck of femur	CD38	Wald ratio	1	-0.54	0.30	0.079	0.58	0.32	1.06

Cancer of stomach	CD38	Wald ratio	1	0.96	0.55	0.079	2.62	0.89	7.66
Hyposmolality and/or hyponatremia	CD38	Wald ratio	1	0.53	0.30	0.080	1.70	0.94	3.09
Gangrene	CD38	Wald ratio	1	0.96	0.55	0.080	2.62	0.89	7.69
Other derangement of joint	CD38	Wald ratio	1	-0.50	0.29	0.081	0.60	0.34	1.06
Hemoptysis	CD38	Wald ratio	1	0.49	0.28	0.084	1.63	0.94	2.85
Excessive or frequent menstruation	CD38	Wald ratio	1	0.24	0.14	0.085	1.27	0.97	1.66
Hallux valgus (Bunion)	CD38	Wald ratio	1	0.28	0.16	0.086	1.32	0.96	1.82
Dysmenorrhea	CD38	Wald ratio	1	0.53	0.31	0.087	1.70	0.93	3.11
Osteoporosis	CD38	Wald ratio	1	0.28	0.16	0.087	1.32	0.96	1.83
Tobacco use disorder	CD38	Wald ratio	1	0.17	0.10	0.087	1.18	0.98	1.43
Duodenal ulcer	CD38	Wald ratio	1	0.41	0.24	0.088	1.50	0.94	2.40
Anorexia	CD38	Wald ratio	1	-0.76	0.45	0.089	0.47	0.19	1.12
Fracture of clavicle or scapula	CD38	Wald ratio	1	0.56	0.33	0.089	1.76	0.92	3.36
Fetal distress and abnormal forces of labor	CD38	Wald ratio	1	-0.49	0.29	0.090	0.61	0.35	1.08
Duodenitis	CD38	Wald ratio	1	0.25	0.15	0.090	1.29	0.96	1.73
Poisoning by psychotropic agents	CD38	Wald ratio	1	-0.50	0.29	0.091	0.61	0.34	1.08
Symptoms and disorders of the joints	CD38	Wald ratio	1	0.36	0.21	0.092	1.43	0.94	2.18
Other and unspecified disc disorder	CD38	Wald ratio	1	0.33	0.20	0.092	1.39	0.95	2.04
Jaundice (not of newborn)	CD38	Wald ratio	1	0.73	0.43	0.092	2.07	0.89	4.83
Enthesopathy	CD38	Wald ratio	1	0.23	0.13	0.092	1.25	0.96	1.63
Benign neoplasm of other endocrine glands and related structures	CD38	Wald ratio	1	0.73	0.44	0.093	2.08	0.88	4.90
Disorders of refraction and accommodation; blindness and low vision	CD38	Wald ratio	1	0.44	0.26	0.094	1.55	0.93	2.59
Fasciitis	CD38	Wald ratio	1	-0.35	0.21	0.097	0.70	0.46	1.07
Genital prolapse	CD38	Wald ratio	1	0.20	0.12	0.100	1.23	0.96	1.56
Atrioventricular [AV] block	CD38	Wald ratio	1	-0.46	0.28	0.100	0.63	0.36	1.09
Nonspecific abnormal findings in stool contents	CD38	Wald ratio	1	0.52	0.32	0.101	1.68	0.90	3.13

Peptic ulcer (excl. esophageal)	CD38	Wald ratio	1	0.25	0.15	0.105	1.28	0.95	1.73
Cardiac dysrhythmias	CD38	Wald ratio	1	0.14	0.09	0.106	1.15	0.97	1.37
Ulcerative colitis (chronic)	CD38	Wald ratio	1	0.90	0.56	0.108	2.45	0.82	7.30
Calculus of kidney	CD38	Wald ratio	1	0.37	0.23	0.111	1.44	0.92	2.27
Electrolyte imbalance	CD38	Wald ratio	1	0.32	0.20	0.113	1.38	0.93	2.06
Mechanical complication of unspecified genitourinary device, implant, and graft	CD38	Wald ratio	1	0.58	0.37	0.114	1.79	0.87	3.69
Anemia of chronic disease	CD38	Wald ratio	1	0.77	0.49	0.114	2.16	0.83	5.62
Cardiomegaly	CD38	Wald ratio	1	0.40	0.26	0.115	1.50	0.91	2.48
Other disorders of circulatory system	CD38	Wald ratio	1	0.16	0.10	0.116	1.18	0.96	1.45
Polymyalgia Rheumatica	CD38	Wald ratio	1	0.60	0.38	0.119	1.82	0.86	3.84
Malignant neoplasm of ovary and other uterine adnexa	CD38	Wald ratio	1	0.44	0.28	0.119	1.55	0.89	2.70
Poisoning by other anti-infectives	CD38	Wald ratio	1	0.57	0.37	0.120	1.77	0.86	3.62
Other symptoms/disorders or the urinary system	CD38	Wald ratio	1	0.13	0.09	0.120	1.14	0.97	1.35
Multiple sclerosis	CD38	Wald ratio	1	-0.55	0.35	0.121	0.58	0.29	1.16
Abnormal sputum	CD38	Wald ratio	1	0.43	0.28	0.122	1.54	0.89	2.65
Alcoholic liver damage	CD38	Wald ratio	1	0.71	0.46	0.122	2.02	0.83	4.95
Peripheral vascular disease	CD38	Wald ratio	1	0.32	0.21	0.123	1.38	0.92	2.08
Abnormal results of function study of liver	CD38	Wald ratio	1	0.34	0.22	0.123	1.41	0.91	2.17
Appendicitis	CD38	Wald ratio	1	0.35	0.23	0.124	1.42	0.91	2.23
Cholelithiasis with other cholecystitis	CD38	Wald ratio	1	0.27	0.18	0.125	1.31	0.93	1.86
Otitis media	CD38	Wald ratio	1	-0.47	0.30	0.125	0.63	0.35	1.14
Otitis externa	CD38	Wald ratio	1	0.86	0.56	0.128	2.36	0.78	7.12
Diabetic retinopathy	CD38	Wald ratio	1	0.54	0.35	0.129	1.71	0.86	3.42
Transient cerebral ischemia	CD38	Wald ratio	1	0.42	0.28	0.129	1.53	0.88	2.64

Swelling, mass, or lump in head and neck [Space-occupying lesion, intracranial NOS]	CD38	Wald ratio	1	0.65	0.43	0.130	1.91	0.83	4.43
Spondylosis without myelopathy	CD38	Wald ratio	1	-0.28	0.18	0.130	0.76	0.53	1.09
Portal hypertension	CD38	Wald ratio	1	0.84	0.56	0.132	2.33	0.78	6.99
Renal failure NOS	CD38	Wald ratio	1	0.52	0.34	0.133	1.68	0.85	3.29
Dysphagia	CD38	Wald ratio	1	0.24	0.16	0.137	1.28	0.93	1.76
Migraine	CD38	Wald ratio	1	0.36	0.24	0.137	1.44	0.89	2.31
Disorders of menstruation and other abnormal bleeding from female genital tract	CD38	Wald ratio	1	0.15	0.10	0.138	1.17	0.95	1.43
Gram negative septicemia	CD38	Wald ratio	1	0.67	0.45	0.138	1.94	0.81	4.68
Psoriasis	CD38	Wald ratio	1	0.40	0.27	0.142	1.50	0.87	2.56
Malignant neoplasm of ovary	CD38	Wald ratio	1	0.42	0.28	0.142	1.52	0.87	2.65
Other inflammatory spondylopathies	CD38	Wald ratio	1	0.47	0.32	0.142	1.59	0.86	2.97
Pulmonary heart disease	CD38	Wald ratio	1	0.29	0.20	0.142	1.34	0.91	1.98
Type 2 diabetes with ophthalmic manifestations	CD38	Wald ratio	1	0.53	0.36	0.143	1.70	0.84	3.44
Other disorders of synovium, tendon, and bursa	CD38	Wald ratio	1	0.22	0.15	0.144	1.24	0.93	1.67
Osteoarthrosis NOS	CD38	Wald ratio	1	0.17	0.12	0.146	1.19	0.94	1.51
Other disorders of bladder	CD38	Wald ratio	1	0.19	0.13	0.146	1.21	0.93	1.57
Adverse drug events and drug allergies	CD38	Wald ratio	1	0.71	0.49	0.150	2.03	0.77	5.31
Other disorders of middle ear and mastoid	CD38	Wald ratio	1	-0.64	0.45	0.152	0.53	0.22	1.27
Psoriasis and related disorders	CD38	Wald ratio	1	0.39	0.27	0.152	1.47	0.87	2.51
Circulatory disease NEC	CD38	Wald ratio	1	0.15	0.11	0.155	1.16	0.94	1.43
Aseptic necrosis of bone	CD38	Wald ratio	1	-0.79	0.55	0.155	0.45	0.15	1.35
Other acquired musculoskeletal deformity	CD38	Wald ratio	1	0.42	0.29	0.155	1.52	0.85	2.71
Seborrheic keratosis	CD38	Wald ratio	1	0.33	0.23	0.156	1.39	0.88	2.20
Alcohol-related disorders	CD38	Wald ratio	1	0.17	0.12	0.157	1.18	0.94	1.49

Poisoning/allergy of sulfonamides	CD38	Wald ratio	1	0.62	0.44	0.157	1.86	0.79	4.38
Other complications of pregnancy NEC	CD38	Wald ratio	1	-0.40	0.28	0.157	0.67	0.38	1.17
Acute appendicitis	CD38	Wald ratio	1	0.36	0.25	0.158	1.43	0.87	2.36
Peripheral enthesopathies and allied syndromes	CD38	Wald ratio	1	0.15	0.11	0.159	1.17	0.94	1.44
Hallux rigidus	CD38	Wald ratio	1	0.46	0.33	0.160	1.59	0.83	3.03
Esophagitis, GERD and related diseases	CD38	Wald ratio	1	0.11	0.08	0.162	1.12	0.96	1.30
Secondary hypothyroidism	CD38	Wald ratio	1	0.54	0.39	0.162	1.72	0.80	3.68
Phlebitis and thrombophlebitis of lower extremities	CD38	Wald ratio	1	0.30	0.22	0.163	1.36	0.88	2.08
Complication of colostomy or enterostomy	CD38	Wald ratio	1	0.80	0.58	0.164	2.23	0.72	6.93
Acquired foot deformities	CD38	Wald ratio	1	0.19	0.13	0.164	1.21	0.93	1.57
Other unspecified back disorders	CD38	Wald ratio	1	0.45	0.32	0.165	1.56	0.83	2.94
Dyspareunia	CD38	Wald ratio	1	0.52	0.38	0.167	1.68	0.80	3.51
Hypertension complicating pregnancy, childbirth, and the puerperium	CD38	Wald ratio	1	-0.55	0.40	0.168	0.58	0.27	1.26
Rash and other nonspecific skin eruption	CD38	Wald ratio	1	0.38	0.28	0.168	1.47	0.85	2.53
Benign mammary dysplasias	CD38	Wald ratio	1	-0.31	0.22	0.170	0.73	0.47	1.14
Gastritis and duodenitis	CD38	Wald ratio	1	0.11	0.08	0.171	1.12	0.95	1.31
Dysuria	CD38	Wald ratio	1	0.51	0.37	0.173	1.66	0.80	3.45
Derangement of joint, non-traumatic	CD38	Wald ratio	1	-0.33	0.24	0.173	0.72	0.45	1.15
Other symptoms of respiratory system	CD38	Wald ratio	1	0.19	0.14	0.175	1.21	0.92	1.58
Other chronic ischemic heart disease, unspecified	CD38	Wald ratio	1	0.15	0.11	0.176	1.17	0.93	1.46
Malignant neoplasm of bladder	CD38	Wald ratio	1	-0.38	0.28	0.177	0.68	0.39	1.19
Other congenital musculoskeletal anomalies	CD38	Wald ratio	1	-0.71	0.53	0.181	0.49	0.17	1.39
Diseases of the jaws	CD38	Wald ratio	1	0.55	0.42	0.183	1.74	0.77	3.94
Other conditions of brain	CD38	Wald ratio	1	-0.45	0.34	0.184	0.63	0.32	1.24
Cervical cancer and dysplasia	CD38	Wald ratio	1	0.29	0.22	0.185	1.33	0.87	2.03
Benign neoplasm of brain and other parts of nervous system	CD38	Wald ratio	1	-0.60	0.45	0.185	0.55	0.23	1.33

Complications of cardiac/vascular device, implant, and graft	CD38	Wald ratio	1	-0.41	0.31	0.185	0.67	0.37	1.21
Infection/inflammation of internal prosthetic device; implant; and graft	CD38	Wald ratio	1	0.34	0.26	0.185	1.41	0.85	2.34
Benign neoplasm of brain, cranial nerves, meninges	CD38	Wald ratio	1	-0.62	0.47	0.185	0.54	0.22	1.34
Malposition and malpresentation of fetus or obstruction	CD38	Wald ratio	1	-0.43	0.32	0.185	0.65	0.35	1.23
Abdominal aortic aneurysm	CD38	Wald ratio	1	0.57	0.43	0.186	1.77	0.76	4.13
Pyelonephritis	CD38	Wald ratio	1	0.46	0.36	0.192	1.59	0.79	3.20
Retinal vascular changes and abnomalities	CD38	Wald ratio	1	-0.58	0.44	0.193	0.56	0.24	1.34
Burns	CD38	Wald ratio	1	-0.70	0.54	0.194	0.50	0.17	1.43
Personal history of diseases of digestive system	CD38	Wald ratio	1	0.14	0.11	0.196	1.15	0.93	1.42
Diseases of the oral soft tissues, excluding lesions specific for gingiva and tongue	CD38	Wald ratio	1	0.27	0.21	0.197	1.31	0.87	1.96
Osteoporosis, osteopenia and pathological fracture	CD38	Wald ratio	1	0.20	0.15	0.198	1.22	0.90	1.64
Right bundle branch block	CD38	Wald ratio	1	-0.43	0.33	0.199	0.65	0.34	1.25
Cancer of other female genital organs	CD38	Wald ratio	1	0.34	0.26	0.201	1.40	0.84	2.34
Symptoms involving head and neck	CD38	Wald ratio	1	0.30	0.24	0.202	1.35	0.85	2.15
Disturbances in tooth eruption	CD38	Wald ratio	1	-0.34	0.27	0.202	0.71	0.42	1.20
Intestinal infection due to C. difficile	CD38	Wald ratio	1	-0.65	0.51	0.202	0.52	0.19	1.41
Nephritis; nephrosis; renal sclerosis	CD38	Wald ratio	1	0.42	0.33	0.204	1.53	0.79	2.93
Other disorders of stomach and duodenum	CD38	Wald ratio	1	0.28	0.22	0.205	1.33	0.86	2.06
Pathologic fracture	CD38	Wald ratio	1	0.72	0.57	0.205	2.06	0.67	6.29
Primary/intrinsic cardiomyopathies	CD38	Wald ratio	1	-0.47	0.37	0.207	0.62	0.30	1.30
Gout	CD38	Wald ratio	1	0.29	0.23	0.208	1.34	0.85	2.11
Benign neoplasm of colon	CD38	Wald ratio	1	0.12	0.10	0.210	1.13	0.93	1.36
Abnormality of gait	CD38	Wald ratio	1	0.40	0.32	0.212	1.50	0.79	2.82

Malignant neoplasm of other and ill-defined sites within the digestive organs and peritoneum	CD38	Wald ratio	1	0.22	0.18	0.214	1.24	0.88	1.75
Osteopenia or other disorder of bone and cartilage	CD38	Wald ratio	1	-0.56	0.45	0.214	0.57	0.24	1.38
Bursitis	CD38	Wald ratio	1	0.55	0.44	0.215	1.73	0.73	4.10
Skin cancer	CD38	Wald ratio	1	-0.14	0.11	0.219	0.87	0.69	1.09
Osteoporosis NOS	CD38	Wald ratio	1	0.22	0.18	0.220	1.24	0.88	1.76
Cholesteatoma	CD38	Wald ratio	1	-0.64	0.52	0.221	0.53	0.19	1.47
Inflammation of the eye	CD38	Wald ratio	1	0.28	0.23	0.222	1.32	0.84	2.08
Irritable Bowel Syndrome	CD38	Wald ratio	1	-0.22	0.18	0.222	0.81	0.57	1.14
Colon cancer	CD38	Wald ratio	1	0.29	0.24	0.223	1.33	0.84	2.12
Other specified nonpsychotic and/or transient mental disorders	CD38	Wald ratio	1	0.62	0.51	0.225	1.86	0.68	5.06
Acute tonsillitis	CD38	Wald ratio	1	0.63	0.52	0.227	1.88	0.68	5.21
Chronic pharyngitis and nasopharyngitis	CD38	Wald ratio	1	0.50	0.42	0.227	1.65	0.73	3.74
Other chronic nonalcoholic liver disease	CD38	Wald ratio	1	-0.38	0.32	0.230	0.68	0.37	1.27
Non-Hodgkins lymphoma	CD38	Wald ratio	1	0.37	0.31	0.231	1.44	0.79	2.63
Postinflammatory pulmonary fibrosis	CD38	Wald ratio	1	0.52	0.43	0.231	1.68	0.72	3.94
Cerebrovascular disease	CD38	Wald ratio	1	0.17	0.14	0.231	1.18	0.90	1.56
Hypovolemia	CD38	Wald ratio	1	0.29	0.24	0.231	1.34	0.83	2.16
Sepsis	CD38	Wald ratio	1	-0.29	0.24	0.231	0.75	0.46	1.20
Sepsis and SIRS	CD38	Wald ratio	1	-0.29	0.24	0.231	0.75	0.46	1.20
Cardiac shunt/ heart septal defect	CD38	Wald ratio	1	-0.64	0.53	0.232	0.53	0.19	1.50
Other benign neoplasm of connective and other soft tissue	CD38	Wald ratio	1	0.46	0.39	0.232	1.59	0.74	3.39
Other disorders of bone and cartilage	CD38	Wald ratio	1	-0.25	0.21	0.232	0.78	0.51	1.18
Noninflammatory female genital disorders	CD38	Wald ratio	1	-0.16	0.14	0.233	0.85	0.65	1.11
Diplopia and disorders of binocular vision	CD38	Wald ratio	1	-0.57	0.48	0.234	0.57	0.22	1.45
Other acquired deformities of limbs	CD38	Wald ratio	1	-0.38	0.32	0.237	0.69	0.37	1.28

Dizziness and giddiness (Light-headedness and vertigo)	CD38	Wald ratio	1	0.23	0.19	0.238	1.25	0.86	1.82
Cardiomyopathy	CD38	Wald ratio	1	-0.43	0.37	0.239	0.65	0.32	1.33
Appendiceal conditions	CD38	Wald ratio	1	0.26	0.22	0.242	1.30	0.84	2.01
Paralytic ileus	CD38	Wald ratio	1	0.66	0.56	0.243	1.93	0.64	5.84
Dementias	CD38	Wald ratio	1	0.49	0.42	0.244	1.63	0.72	3.69
Intestinal malabsorption (non-celiac)	CD38	Wald ratio	1	-0.33	0.28	0.247	0.72	0.41	1.26
Other and unspecified disorders of back	CD38	Wald ratio	1	0.33	0.28	0.247	1.39	0.80	2.42
Disorders of fluid, electrolyte, and acid-base balance	CD38	Wald ratio	1	0.18	0.15	0.248	1.19	0.88	1.61
Disorders of external ear	CD38	Wald ratio	1	0.39	0.34	0.250	1.48	0.76	2.88
Rupture of synovium	CD38	Wald ratio	1	0.61	0.53	0.251	1.84	0.65	5.19
Disorders of tooth development	CD38	Wald ratio	1	-0.30	0.26	0.252	0.74	0.44	1.24
Cholelithiasis	CD38	Wald ratio	1	0.13	0.11	0.253	1.14	0.91	1.43
Secondary malignancy of lymph nodes	CD38	Wald ratio	1	0.20	0.18	0.253	1.23	0.86	1.74
Fracture of unspecified bones	CD38	Wald ratio	1	0.41	0.36	0.256	1.50	0.75	3.03
Postoperative infection	CD38	Wald ratio	1	0.22	0.20	0.256	1.25	0.85	1.83
Hemorrhage or hematoma complicating a procedure	CD38	Wald ratio	1	0.20	0.18	0.258	1.22	0.86	1.74
Diaphragmatic hernia	CD38	Wald ratio	1	0.10	0.08	0.258	1.10	0.93	1.30
Acute and chronic tonsillitis	CD38	Wald ratio	1	0.33	0.29	0.259	1.39	0.79	2.44
Congenital anomalies of genital organs	CD38	Wald ratio	1	-0.54	0.48	0.260	0.58	0.23	1.49
Cancer of bladder	CD38	Wald ratio	1	-0.30	0.26	0.261	0.74	0.44	1.25
Ulceration of the lower GI tract	CD38	Wald ratio	1	0.44	0.40	0.262	1.56	0.72	3.39
Other disorders of liver	CD38	Wald ratio	1	0.19	0.17	0.266	1.21	0.87	1.69
Diseases of nail, NOS	CD38	Wald ratio	1	-0.40	0.36	0.267	0.67	0.33	1.36
Disorders of mineral metabolism	CD38	Wald ratio	1	0.31	0.28	0.269	1.37	0.79	2.37
Other open wound of head and face	CD38	Wald ratio	1	0.27	0.24	0.269	1.30	0.81	2.09
Hypertensive heart and/or renal disease	CD38	Wald ratio	1	0.35	0.31	0.269	1.41	0.77	2.61

Prolapse of vaginal vault after hysterectomy	CD38	Wald ratio	1	0.63	0.57	0.270	1.88	0.61	5.75
Allergy/adverse effect of penicillin	CD38	Wald ratio	1	0.12	0.11	0.274	1.12	0.91	1.38
Melanomas of skin, dx or hx	CD38	Wald ratio	1	-0.27	0.25	0.274	0.76	0.46	1.24
Melanomas of skin	CD38	Wald ratio	1	-0.27	0.25	0.274	0.76	0.46	1.24
Other disorders of metabolism	CD38	Wald ratio	1	0.38	0.34	0.275	1.46	0.74	2.86
Degeneration of intervertebral disc	CD38	Wald ratio	1	-0.27	0.24	0.276	0.77	0.48	1.24
Cyst of kidney, acquired	CD38	Wald ratio	1	0.40	0.36	0.277	1.49	0.73	3.03
Nonrheumatic mitral valve disorders	CD38	Wald ratio	1	0.26	0.24	0.277	1.30	0.81	2.09
Other arthropathies	CD38	Wald ratio	1	0.08	0.07	0.278	1.08	0.94	1.25
Late pregnancy and failed induction	CD38	Wald ratio	1	0.40	0.37	0.278	1.50	0.72	3.11
Alcoholism	CD38	Wald ratio	1	0.15	0.14	0.278	1.16	0.88	1.53
Celiac disease	CD38	Wald ratio	1	-0.33	0.30	0.281	0.72	0.40	1.31
Injuries to the nervous system	CD38	Wald ratio	1	0.38	0.36	0.282	1.47	0.73	2.95
Other tests	CD38	Wald ratio	1	0.18	0.17	0.282	1.20	0.86	1.67
Acidosis	CD38	Wald ratio	1	-0.43	0.40	0.284	0.65	0.30	1.43
Type 2 diabetes with neurological manifestations	CD38	Wald ratio	1	0.58	0.54	0.286	1.78	0.62	5.11
Osteoarthrosis, localized, primary	CD38	Wald ratio	1	0.15	0.14	0.287	1.16	0.88	1.52
Pain in limb	CD38	Wald ratio	1	0.18	0.17	0.289	1.19	0.86	1.65
Simple and unspecified goiter	CD38	Wald ratio	1	0.56	0.53	0.289	1.75	0.62	4.90
Ischemic Heart Disease	CD38	Wald ratio	1	0.09	0.08	0.289	1.09	0.93	1.28
Pain and other symptoms associated with female genital organs	CD38	Wald ratio	1	0.25	0.24	0.291	1.29	0.81	2.05
Anxiety disorder	CD38	Wald ratio	1	0.17	0.16	0.294	1.19	0.86	1.64
Nasal polyps	CD38	Wald ratio	1	0.24	0.23	0.298	1.27	0.81	1.98
Intestinal infection	CD38	Wald ratio	1	-0.14	0.14	0.298	0.87	0.66	1.13
Abnormal glucose	CD38	Wald ratio	1	0.51	0.49	0.299	1.67	0.64	4.38
Erythematous conditions	CD38	Wald ratio	1	0.27	0.26	0.301	1.31	0.78	2.19

Unspecified polyarthropathy or polyarthritis	CD38	Wald ratio	1	0.23	0.22	0.302	1.26	0.82	1.93
Gout and other crystal arthropathies	CD38	Wald ratio	1	0.22	0.21	0.303	1.25	0.82	1.89
Traumatic arthropathy	CD38	Wald ratio	1	0.58	0.56	0.303	1.78	0.59	5.35
Inflammation of eyelids	CD38	Wald ratio	1	0.27	0.26	0.304	1.31	0.78	2.20
Joint effusions	CD38	Wald ratio	1	0.35	0.34	0.305	1.42	0.73	2.78
Symptoms concerning nutrition, metabolism, and development	CD38	Wald ratio	1	-0.18	0.18	0.305	0.83	0.59	1.18
Anal and rectal polyp	CD38	Wald ratio	1	0.16	0.15	0.306	1.17	0.87	1.58
Mood disorders	CD38	Wald ratio	1	0.12	0.12	0.306	1.13	0.89	1.43
Urinary calculus	CD38	Wald ratio	1	0.17	0.16	0.307	1.18	0.86	1.62
Ascites (non malignant)	CD38	Wald ratio	1	0.34	0.33	0.307	1.40	0.73	2.66
Bladder neck obstruction	CD38	Wald ratio	1	0.30	0.29	0.307	1.35	0.76	2.39
Delirium due to conditions classified elsewhere	CD38	Wald ratio	1	0.51	0.50	0.312	1.67	0.62	4.48
Cholelithiasis and cholecystitis	CD38	Wald ratio	1	0.11	0.11	0.313	1.11	0.90	1.37
Irregular menstrual bleeding	CD38	Wald ratio	1	0.21	0.21	0.313	1.24	0.82	1.88
Subjective visual disturbances	CD38	Wald ratio	1	0.52	0.52	0.316	1.69	0.61	4.67
Symptoms involving digestive system	CD38	Wald ratio	1	0.10	0.10	0.317	1.11	0.90	1.36
Superficial injury without mention of infection	CD38	Wald ratio	1	0.20	0.20	0.317	1.22	0.83	1.79
Inflammatory disease of cervix, vagina, and vulva	CD38	Wald ratio	1	0.24	0.24	0.323	1.27	0.79	2.05
Suppurative and unspecified otitis media	CD38	Wald ratio	1	-0.43	0.44	0.326	0.65	0.27	1.54
Crushing or internal injury to organs	CD38	Wald ratio	1	0.40	0.41	0.327	1.49	0.67	3.30
Other diseases of blood and blood-forming organs	CD38	Wald ratio	1	0.20	0.20	0.328	1.22	0.82	1.81
Hypotension NOS	CD38	Wald ratio	1	-0.21	0.22	0.330	0.81	0.53	1.24
Hypertension	CD38	Wald ratio	1	0.06	0.06	0.333	1.06	0.94	1.19
Cervicitis and endocervicitis	CD38	Wald ratio	1	0.34	0.36	0.334	1.41	0.70	2.84
Abnormal function study of cardiovascular system	CD38	Wald ratio	1	0.54	0.56	0.335	1.72	0.57	5.21
Chronic periodontitis	CD38	Wald ratio	1	0.52	0.54	0.335	1.69	0.58	4.91

Voice disturbance	CD38	Wald ratio	1	0.38	0.39	0.336	1.46	0.68	3.15
Cellulitis and abscess of fingers/toes	CD38	Wald ratio	1	-0.52	0.54	0.336	0.59	0.20	1.72
Open-angle glaucoma	CD38	Wald ratio	1	0.39	0.40	0.337	1.47	0.67	3.23
Inflammatory and toxic neuropathy	CD38	Wald ratio	1	0.33	0.35	0.338	1.40	0.71	2.76
Vaginal enterocele, congenital or acquired	CD38	Wald ratio	1	0.48	0.50	0.338	1.61	0.61	4.25
Anal and rectal conditions	CD38	Wald ratio	1	0.10	0.11	0.338	1.11	0.90	1.36
Benign neoplasm of breast	CD38	Wald ratio	1	-0.31	0.33	0.339	0.73	0.38	1.39
Unstable angina (intermediate coronary syndrome)	CD38	Wald ratio	1	-0.18	0.18	0.339	0.84	0.59	1.20
Contusion	CD38	Wald ratio	1	0.32	0.33	0.340	1.38	0.71	2.65
Cancer within the respiratory system	CD38	Wald ratio	1	0.24	0.25	0.341	1.27	0.78	2.07
Pelvic peritoneal adhesions, female (postoperative) (postinfection)	CD38	Wald ratio	1	0.25	0.26	0.341	1.28	0.77	2.12
Cardiac and circulatory congenital anomalies	CD38	Wald ratio	1	-0.23	0.25	0.343	0.79	0.49	1.28
Secondary malignancy of respiratory organs	CD38	Wald ratio	1	-0.26	0.28	0.343	0.77	0.45	1.32
Gastric ulcer	CD38	Wald ratio	1	0.19	0.20	0.344	1.21	0.81	1.81
Symptoms involving skin and other integumentary tissue	CD38	Wald ratio	1	-0.29	0.30	0.344	0.75	0.41	1.36
Intracerebral hemorrhage	CD38	Wald ratio	1	-0.46	0.49	0.344	0.63	0.24	1.64
Raynaud's syndrome	CD38	Wald ratio	1	0.36	0.38	0.344	1.43	0.68	3.03
Aphakia and other disorders of lens	CD38	Wald ratio	1	-0.29	0.30	0.347	0.75	0.41	1.36
Inflammatory diseases of female pelvic organs	CD38	Wald ratio	1	0.15	0.17	0.352	1.17	0.84	1.62
Other disorders of peritoneum	CD38	Wald ratio	1	0.21	0.23	0.354	1.23	0.79	1.92
Atrial fibrillation and flutter	CD38	Wald ratio	1	0.10	0.11	0.354	1.11	0.89	1.38
Cyst or abscess of Bartholin's gland	CD38	Wald ratio	1	0.43	0.47	0.354	1.54	0.62	3.87
Hemiplegia	CD38	Wald ratio	1	0.31	0.33	0.357	1.36	0.71	2.62
Noninflammatory disorders of vulva and perineum	CD38	Wald ratio	1	-0.32	0.35	0.357	0.72	0.36	1.44
Diseases of the larynx and vocal cords	CD38	Wald ratio	1	0.23	0.25	0.357	1.26	0.77	2.07
Atherosclerosis of the extremities	CD38	Wald ratio	1	0.42	0.46	0.358	1.52	0.62	3.71

Agorophobia, social phobia, and panic disorder	CD38	Wald ratio	1	0.45	0.49	0.359	1.56	0.60	4.05
Glomerulonephritis	CD38	Wald ratio	1	0.37	0.40	0.359	1.45	0.66	3.19
Hematemesis	CD38	Wald ratio	1	0.27	0.29	0.359	1.31	0.74	2.32
Other specified osteoporosis	CD38	Wald ratio	1	0.51	0.56	0.361	1.67	0.56	5.01
Nausea and vomiting	CD38	Wald ratio	1	-0.11	0.12	0.362	0.89	0.70	1.14
Visual disturbances	CD38	Wald ratio	1	0.21	0.23	0.364	1.23	0.79	1.91
Vertiginous syndromes and other disorders of vestibular system	CD38	Wald ratio	1	0.15	0.17	0.364	1.16	0.84	1.61
Hammer toe (acquired)	CD38	Wald ratio	1	-0.27	0.30	0.366	0.77	0.43	1.37
Other anemias	CD38	Wald ratio	1	0.11	0.12	0.368	1.11	0.88	1.40
Effects radiation NOS	CD38	Wald ratio	1	0.21	0.23	0.369	1.23	0.78	1.94
Nontoxic nodular goiter	CD38	Wald ratio	1	0.34	0.38	0.370	1.41	0.66	2.99
Endocarditis	CD38	Wald ratio	1	-0.44	0.49	0.372	0.64	0.25	1.69
First degree AV block	CD38	Wald ratio	1	-0.37	0.41	0.372	0.69	0.31	1.55
Primary open angle glaucoma	CD38	Wald ratio	1	0.36	0.40	0.372	1.43	0.65	3.16
Neurological disorders	CD38	Wald ratio	1	0.17	0.19	0.373	1.18	0.82	1.72
Essential hypertension	CD38	Wald ratio	1	0.05	0.06	0.374	1.05	0.94	1.18
Calculus of ureter	CD38	Wald ratio	1	0.24	0.26	0.375	1.26	0.75	2.13
Bacterial enteritis	CD38	Wald ratio	1	-0.22	0.25	0.376	0.80	0.50	1.30
Diseases of hard tissues of teeth	CD38	Wald ratio	1	0.21	0.23	0.377	1.23	0.78	1.95
Depression	CD38	Wald ratio	1	0.11	0.12	0.379	1.11	0.88	1.41
Fracture of foot	CD38	Wald ratio	1	0.26	0.30	0.381	1.30	0.72	2.36
Abdominal hernia	CD38	Wald ratio	1	0.06	0.06	0.381	1.06	0.93	1.20
Intestinal obstruction without mention of hernia	CD38	Wald ratio	1	0.18	0.21	0.383	1.20	0.80	1.79
Peritoneal adhesions (postoperative) (postinfection)	CD38	Wald ratio	1	0.20	0.23	0.386	1.22	0.78	1.92
Degenerative skin conditions and other dermatoses	CD38	Wald ratio	1	0.15	0.18	0.389	1.16	0.82	1.64
Delirium dementia and amnestic and other cognitive disorders	CD38	Wald ratio	1	0.25	0.29	0.390	1.29	0.73	2.28

Myalgia and myositis unspecified	CD38	Wald ratio	1	0.44	0.51	0.391	1.55	0.57	4.21
Malaise and fatigue	CD38	Wald ratio	1	-0.19	0.22	0.391	0.83	0.53	1.28
Open wounds of extremities	CD38	Wald ratio	1	0.17	0.20	0.392	1.19	0.80	1.76
Degeneration of macula and posterior pole of retina	CD38	Wald ratio	1	0.24	0.28	0.394	1.27	0.74	2.18
Carcinoma in situ of skin	CD38	Wald ratio	1	0.43	0.50	0.396	1.53	0.57	4.09
Pneumonitis due to inhalation of food or vomitus	CD38	Wald ratio	1	0.45	0.53	0.399	1.57	0.55	4.48
Macular degeneration (senile) of retina NOS	CD38	Wald ratio	1	0.23	0.28	0.399	1.26	0.73	2.18
Pleurisy; pleural effusion	CD38	Wald ratio	1	0.14	0.16	0.399	1.15	0.83	1.57
Decubitus ulcer	CD38	Wald ratio	1	0.36	0.43	0.403	1.43	0.62	3.30
Fracture of pelvis	CD38	Wald ratio	1	0.40	0.48	0.403	1.49	0.58	3.84
Other disorders of the kidney and ureters	CD38	Wald ratio	1	0.19	0.23	0.406	1.21	0.78	1.87
Heart valve disorders	CD38	Wald ratio	1	0.17	0.20	0.410	1.18	0.80	1.75
Heartburn	CD38	Wald ratio	1	0.23	0.28	0.410	1.26	0.73	2.19
Synovitis and tenosynovitis	CD38	Wald ratio	1	0.20	0.25	0.414	1.22	0.75	1.98
Superficial cellulitis and abscess	CD38	Wald ratio	1	-0.12	0.15	0.419	0.88	0.66	1.19
Osteoarthrosis	CD38	Wald ratio	1	0.07	0.08	0.420	1.07	0.91	1.26
Liver abscess and sequelae of chronic liver disease	CD38	Wald ratio	1	0.34	0.42	0.420	1.40	0.62	3.20
Pelvic inflammatory disease (PID)	CD38	Wald ratio	1	0.34	0.42	0.421	1.41	0.61	3.22
Malignant neoplasm of rectum, rectosigmoid junction, and anus	CD38	Wald ratio	1	-0.23	0.28	0.424	0.80	0.46	1.39
Obstetrical/birth trauma	CD38	Wald ratio	1	0.16	0.20	0.426	1.17	0.79	1.72
Other diseases of respiratory system, not elsewhere classified	CD38	Wald ratio	1	0.11	0.13	0.426	1.11	0.86	1.44
Noninfectious gastroenteritis	CD38	Wald ratio	1	0.08	0.11	0.426	1.09	0.88	1.34
Degenerative disease of the spinal cord	CD38	Wald ratio	1	-0.24	0.30	0.427	0.78	0.43	1.43
Abnormal findings on exam of gastrointestinal tract/ abdominal area	CD38	Wald ratio	1	0.25	0.32	0.427	1.29	0.69	2.40
Epiphora	CD38	Wald ratio	1	0.34	0.43	0.427	1.41	0.60	3.28
Other dyspnea	CD38	Wald ratio	1	0.34	0.43	0.428	1.40	0.61	3.25

Neoplasm of uncertain behavior	CD38	Wald ratio	1	0.27	0.34	0.429	1.31	0.67	2.57
Fracture of humerus	CD38	Wald ratio	1	0.25	0.31	0.430	1.28	0.69	2.37
Perforation of tympanic membrane	CD38	Wald ratio	1	-0.32	0.40	0.431	0.73	0.33	1.60
Elevated blood pressure reading without diagnosis of hypertension	CD38	Wald ratio	1	-0.26	0.34	0.432	0.77	0.40	1.48
Peripheral vascular disease, unspecified	CD38	Wald ratio	1	0.20	0.26	0.432	1.22	0.74	2.03
Ankylosing spondylitis	CD38	Wald ratio	1	0.41	0.52	0.432	1.50	0.54	4.16
Allergic rhinitis	CD38	Wald ratio	1	0.31	0.40	0.433	1.37	0.63	2.97
Polyarteritis nodosa and allied conditions	CD38	Wald ratio	1	-0.35	0.45	0.436	0.70	0.29	1.70
Fracture of upper limb	CD38	Wald ratio	1	0.11	0.14	0.436	1.12	0.84	1.48
Myocardial infarction	CD38	Wald ratio	1	0.10	0.13	0.437	1.10	0.86	1.41
Hydronephrosis	CD38	Wald ratio	1	0.23	0.29	0.439	1.25	0.71	2.22
Foreign body injury	CD38	Wald ratio	1	-0.29	0.37	0.439	0.75	0.36	1.56
Dental caries	CD38	Wald ratio	1	0.18	0.24	0.440	1.20	0.76	1.90
Arthropathy NOS	CD38	Wald ratio	1	0.06	0.07	0.440	1.06	0.92	1.22
Manlignant and unknown neoplasms of brain and nervous system	CD38	Wald ratio	1	-0.39	0.50	0.442	0.68	0.25	1.82
Colorectal cancer	CD38	Wald ratio	1	0.15	0.19	0.443	1.16	0.79	1.70
Megaloblastic anemia	CD38	Wald ratio	1	0.30	0.39	0.444	1.35	0.62	2.93
Other aneurysm	CD38	Wald ratio	1	0.23	0.30	0.446	1.26	0.69	2.29
Postmenopausal atrophic vaginitis	CD38	Wald ratio	1	-0.30	0.39	0.446	0.74	0.35	1.60
Complication of internal orthopedic device	CD38	Wald ratio	1	0.18	0.23	0.447	1.19	0.76	1.87
Other non-epithelial cancer of skin	CD38	Wald ratio	1	-0.10	0.13	0.449	0.91	0.71	1.17
Endometrial hyperplasia	CD38	Wald ratio	1	0.29	0.38	0.455	1.33	0.63	2.83
Genitourinary congenital anomalies	CD38	Wald ratio	1	-0.24	0.32	0.455	0.79	0.42	1.48
Other diseases of the teeth and supporting structures	CD38	Wald ratio	1	0.19	0.25	0.457	1.20	0.74	1.96
Other disorders of intestine	CD38	Wald ratio	1	0.15	0.20	0.458	1.16	0.78	1.72
Iron deficiency anemias, unspecified or not due to blood loss	CD38	Wald ratio	1	-0.11	0.15	0.460	0.89	0.66	1.20

Fracture of vertebral column without mention of spinal cord injury	CD38	Wald ratio	1	0.26	0.35	0.461	1.30	0.65	2.58
Cardiac pacemaker/device in situ	CD38	Wald ratio	1	-0.19	0.26	0.462	0.83	0.50	1.38
Diabetes mellitus	CD38	Wald ratio	1	0.07	0.10	0.463	1.07	0.89	1.30
Decreased white blood cell count	CD38	Wald ratio	1	-0.17	0.23	0.463	0.85	0.54	1.32
Neutropenia	CD38	Wald ratio	1	-0.17	0.23	0.463	0.85	0.54	1.32
Viral warts & HPV	CD38	Wald ratio	1	0.30	0.41	0.464	1.35	0.61	2.99
Scar conditions and fibrosis of skin	CD38	Wald ratio	1	0.19	0.27	0.465	1.21	0.72	2.04
Obstruction of bile duct	CD38	Wald ratio	1	-0.34	0.46	0.470	0.71	0.29	1.78
Urinary tract infection	CD38	Wald ratio	1	-0.08	0.12	0.470	0.92	0.73	1.16
Premature beats	CD38	Wald ratio	1	0.40	0.56	0.471	1.49	0.50	4.46
Chronic glomerulonephritis, NOS	CD38	Wald ratio	1	0.32	0.45	0.472	1.38	0.58	3.30
Extrapyramidal disease and abnormal movement disorders	CD38	Wald ratio	1	-0.31	0.43	0.472	0.73	0.31	1.71
Cellulitis and abscess of trunk	CD38	Wald ratio	1	-0.38	0.53	0.473	0.69	0.24	1.92
Iron deficiency anemias	CD38	Wald ratio	1	-0.11	0.15	0.476	0.90	0.67	1.20
Bacterial pneumonia	CD38	Wald ratio	1	-0.11	0.16	0.477	0.89	0.65	1.22
Acquired spondylolisthesis	CD38	Wald ratio	1	0.23	0.33	0.480	1.26	0.66	2.42
Lipoma of skin and subcutaneous tissue	CD38	Wald ratio	1	0.14	0.19	0.480	1.14	0.79	1.67
Varicose veins	CD38	Wald ratio	1	-0.09	0.12	0.480	0.92	0.72	1.17
Varicose veins of lower extremity, symptomtic	CD38	Wald ratio	1	0.36	0.51	0.481	1.44	0.52	3.93
Reflux esophagitis	CD38	Wald ratio	1	0.09	0.13	0.481	1.09	0.85	1.41
Atopic/contact dermatitis due to other or unspecified	CD38	Wald ratio	1	-0.20	0.28	0.481	0.82	0.47	1.43
Streptococcus infection	CD38	Wald ratio	1	-0.22	0.32	0.482	0.80	0.43	1.49
Hyperparathyroidism	CD38	Wald ratio	1	-0.33	0.46	0.482	0.72	0.29	1.79
Cancer of brain and nervous system	CD38	Wald ratio	1	-0.39	0.56	0.483	0.67	0.22	2.02
Varicose veins of lower extremity	CD38	Wald ratio	1	-0.09	0.12	0.483	0.92	0.72	1.17
Psychogenic and somatoform disorders	CD38	Wald ratio	1	0.39	0.56	0.489	1.47	0.49	4.43

Shortness of breath	CD38	Wald ratio	1	0.12	0.17	0.489	1.13	0.81	1.57
Aortic valve disease	CD38	Wald ratio	1	0.25	0.36	0.491	1.29	0.63	2.63
Hemorrhage of rectum and anus	CD38	Wald ratio	1	-0.08	0.11	0.491	0.92	0.74	1.16
Neoplasm of unspecified nature of digestive system	CD38	Wald ratio	1	0.27	0.40	0.493	1.31	0.60	2.86
Anxiety disorders	CD38	Wald ratio	1	0.11	0.16	0.494	1.11	0.82	1.52
Fracture of ankle and foot	CD38	Wald ratio	1	0.18	0.27	0.495	1.20	0.71	2.03
Other venous embolism and thrombosis	CD38	Wald ratio	1	0.37	0.55	0.498	1.45	0.50	4.22
Secondary malignancy of bone	CD38	Wald ratio	1	-0.19	0.28	0.498	0.83	0.48	1.43
Tinnitus	CD38	Wald ratio	1	-0.38	0.57	0.500	0.68	0.22	2.08
Angina pectoris	CD38	Wald ratio	1	0.07	0.11	0.500	1.08	0.87	1.33
Fracture of hand or wrist	CD38	Wald ratio	1	0.15	0.22	0.500	1.16	0.75	1.80
Unspecified monoarthritis	CD38	Wald ratio	1	-0.07	0.11	0.500	0.93	0.75	1.15
Cardiac congenital anomalies	CD38	Wald ratio	1	-0.17	0.25	0.502	0.84	0.51	1.39
Benign neoplasm of ovary	CD38	Wald ratio	1	0.22	0.34	0.505	1.25	0.65	2.42
Abnormal findings on examination of urine	CD38	Wald ratio	1	-0.14	0.22	0.506	0.87	0.57	1.32
Cancer of esophagus	CD38	Wald ratio	1	0.32	0.48	0.507	1.38	0.53	3.55
Pneumococcal pneumonia	CD38	Wald ratio	1	-0.11	0.17	0.507	0.89	0.64	1.25
Benign neoplasm of other parts of digestive system	CD38	Wald ratio	1	0.12	0.18	0.510	1.13	0.79	1.60
Curvature of spine	CD38	Wald ratio	1	-0.25	0.38	0.511	0.78	0.37	1.65
Carditis	CD38	Wald ratio	1	-0.19	0.29	0.511	0.83	0.47	1.46
Cholecystitis without cholelithiasis	CD38	Wald ratio	1	0.16	0.25	0.512	1.18	0.72	1.91
Edema	CD38	Wald ratio	1	-0.21	0.32	0.513	0.81	0.43	1.52
Problems associated with amniotic cavity and membranes	CD38	Wald ratio	1	-0.23	0.35	0.515	0.79	0.40	1.59
Femoral hernia	CD38	Wald ratio	1	-0.33	0.51	0.517	0.72	0.27	1.95
Esophageal bleeding (varices/hemorrhage)	CD38	Wald ratio	1	0.20	0.32	0.518	1.23	0.66	2.28
Skull and face fracture and other intercranial injury	CD38	Wald ratio	1	0.15	0.24	0.518	1.17	0.73	1.86

Postmenopausal bleeding	CD38	Wald ratio	1	0.09	0.14	0.519	1.09	0.83	1.44
Abnormal movement	CD38	Wald ratio	1	0.16	0.25	0.522	1.17	0.72	1.90
Chronic cystitis	CD38	Wald ratio	1	0.27	0.43	0.526	1.32	0.56	3.07
Opiates and related narcotics causing adverse effects in therapeutic use	CD38	Wald ratio	1	-0.24	0.38	0.528	0.79	0.37	1.66
Disorders of uterus, NEC	CD38	Wald ratio	1	-0.14	0.23	0.530	0.87	0.56	1.35
Prolapse of vaginal walls	CD38	Wald ratio	1	0.10	0.15	0.532	1.10	0.81	1.49
Myopia	CD38	Wald ratio	1	0.23	0.37	0.532	1.26	0.61	2.57
Disorders of calcium/phosphorus metabolism	CD38	Wald ratio	1	-0.23	0.37	0.532	0.79	0.38	1.64
Hemorrhage of gastrointestinal tract	CD38	Wald ratio	1	0.11	0.18	0.532	1.12	0.79	1.59
Other intestinal obstruction	CD38	Wald ratio	1	0.14	0.22	0.534	1.15	0.74	1.78
Chronic pancreatitis	CD38	Wald ratio	1	-0.35	0.57	0.536	0.70	0.23	2.15
Rheumatism, unspecified and fibrositis	CD38	Wald ratio	1	0.25	0.41	0.538	1.29	0.58	2.86
Urinary incontinence	CD38	Wald ratio	1	0.09	0.14	0.539	1.09	0.83	1.44
Vitamin deficiency	CD38	Wald ratio	1	0.23	0.37	0.544	1.25	0.60	2.60
Other diseases of respiratory system, NEC	CD38	Wald ratio	1	0.08	0.14	0.544	1.09	0.83	1.42
Rheumatic disease of the heart valves	CD38	Wald ratio	1	0.11	0.19	0.544	1.12	0.78	1.62
Schizophrenia and other psychotic disorders	CD38	Wald ratio	1	0.27	0.44	0.545	1.31	0.55	3.12
Pericarditis	CD38	Wald ratio	1	-0.22	0.36	0.547	0.80	0.39	1.64
Acid-base balance disorder	CD38	Wald ratio	1	-0.23	0.38	0.547	0.80	0.38	1.67
Retinal detachment with retinal defect	CD38	Wald ratio	1	0.21	0.35	0.550	1.23	0.62	2.43
Chronic bronchitis	CD38	Wald ratio	1	-0.14	0.24	0.550	0.87	0.54	1.39
Disorders of esophageal motility	CD38	Wald ratio	1	-0.31	0.51	0.550	0.74	0.27	2.01
Poisoning by antibiotics	CD38	Wald ratio	1	0.06	0.10	0.550	1.06	0.87	1.29
Cancer of other lymphoid, histiocytic tissue	CD38	Wald ratio	1	0.16	0.27	0.551	1.18	0.69	2.01
Hypoglycemia	CD38	Wald ratio	1	-0.25	0.42	0.551	0.78	0.34	1.78

Mucous polyp of cervix	CD38	Wald ratio	1	0.13	0.22	0.551	1.14	0.74	1.76
Aortic aneurysm	CD38	Wald ratio	1	0.21	0.35	0.553	1.23	0.62	2.43
Septal Deviations/Turbinate Hypertrophy	CD38	Wald ratio	1	-0.11	0.19	0.555	0.90	0.62	1.29
Other disorders of cervical region	CD38	Wald ratio	1	-0.33	0.57	0.555	0.72	0.24	2.17
Other disorders of pancreatic internal secretion	CD38	Wald ratio	1	-0.25	0.42	0.557	0.78	0.34	1.78
Other retinal disorders	CD38	Wald ratio	1	-0.12	0.21	0.557	0.88	0.59	1.33
Occlusion of cerebral arteries	CD38	Wald ratio	1	0.12	0.20	0.559	1.13	0.76	1.68
Pain in joint	CD38	Wald ratio	1	0.09	0.15	0.561	1.09	0.82	1.45
Calculus of lower urinary tract	CD38	Wald ratio	1	-0.27	0.46	0.561	0.76	0.31	1.90
Hematuria	CD38	Wald ratio	1	0.06	0.10	0.564	1.06	0.87	1.30
Pulmonary collapse; interstitial and compensatory emphysema	CD38	Wald ratio	1	0.17	0.29	0.565	1.18	0.67	2.08
Acute upper respiratory infections of multiple or unspecified sites	CD38	Wald ratio	1	0.15	0.27	0.568	1.16	0.69	1.96
Benign neoplasm of lip, oral cavity, and pharynx	CD38	Wald ratio	1	-0.23	0.41	0.569	0.79	0.35	1.77
Renal colic	CD38	Wald ratio	1	-0.16	0.29	0.570	0.85	0.49	1.49
Congestive heart failure (CHF) NOS	CD38	Wald ratio	1	0.16	0.29	0.570	1.18	0.67	2.08
Psoriasis vulgaris	CD38	Wald ratio	1	0.18	0.32	0.571	1.20	0.64	2.22
Menopausal and postmenopausal disorders	CD38	Wald ratio	1	0.07	0.13	0.571	1.08	0.84	1.39
Other deficiency anemia	CD38	Wald ratio	1	0.22	0.38	0.575	1.24	0.58	2.64
Bacterial infection NOS	CD38	Wald ratio	1	0.07	0.12	0.575	1.07	0.85	1.35
Meniere's disease	CD38	Wald ratio	1	-0.30	0.54	0.577	0.74	0.26	2.13
Other specified gastritis	CD38	Wald ratio	1	0.08	0.15	0.582	1.08	0.81	1.44
Prurigo and Lichen	CD38	Wald ratio	1	-0.25	0.46	0.584	0.78	0.31	1.92
Nonspecific findings on examination of blood	CD38	Wald ratio	1	-0.09	0.16	0.584	0.91	0.67	1.26
Osteomyelitis, periostitis, and other infections involving bone	CD38	Wald ratio	1	-0.28	0.52	0.584	0.75	0.27	2.09
Regional enteritis	CD38	Wald ratio	1	0.17	0.31	0.586	1.18	0.64	2.18
Functional digestive disorders	CD38	Wald ratio	1	0.05	0.09	0.589	1.05	0.88	1.25

Cholelithiasis with acute cholecystitis	CD38	Wald ratio	1	0.18	0.33	0.589	1.20	0.62	2.31
Cardiac pacemaker in situ	CD38	Wald ratio	1	-0.15	0.27	0.589	0.86	0.51	1.47
Noninfectious disorders of lymphatic channels	CD38	Wald ratio	1	0.26	0.49	0.590	1.30	0.50	3.38
Cellulitis and abscess of face/neck	CD38	Wald ratio	1	0.30	0.56	0.592	1.35	0.45	4.09
Mitral valve disease	CD38	Wald ratio	1	0.13	0.24	0.593	1.14	0.71	1.81
Hyperpotassemia	CD38	Wald ratio	1	-0.22	0.41	0.595	0.80	0.36	1.81
Ptosis of eyelid	CD38	Wald ratio	1	-0.16	0.30	0.597	0.85	0.47	1.55
Diseases of pulp and periapical tissues	CD38	Wald ratio	1	-0.16	0.30	0.598	0.85	0.47	1.55
Other abnormal blood chemistry	CD38	Wald ratio	1	-0.09	0.16	0.599	0.92	0.66	1.27
Disorders of the pituitary gland and its hypothalamic control	CD38	Wald ratio	1	-0.26	0.49	0.600	0.77	0.30	2.02
Other specified benign mammary dysplasias	CD38	Wald ratio	1	-0.26	0.49	0.602	0.77	0.30	2.02
Respiratory failure	CD38	Wald ratio	1	-0.15	0.29	0.602	0.86	0.49	1.51
Cardiac conduction disorders	CD38	Wald ratio	1	-0.08	0.16	0.605	0.92	0.68	1.26
Palpitations	CD38	Wald ratio	1	0.11	0.21	0.607	1.12	0.74	1.69
Chronic fatigue syndrome	CD38	Wald ratio	1	-0.27	0.53	0.612	0.76	0.27	2.16
Acute renal failure	CD38	Wald ratio	1	-0.10	0.19	0.614	0.91	0.62	1.33
Sicca syndrome	CD38	Wald ratio	1	-0.29	0.57	0.616	0.75	0.24	2.30
Malunion and nonunion of fracture	CD38	Wald ratio	1	0.18	0.36	0.621	1.20	0.59	2.44
Secondary malignancy of brain/spine	CD38	Wald ratio	1	-0.22	0.46	0.623	0.80	0.33	1.95
Nontoxic multinodular goiter	CD38	Wald ratio	1	0.24	0.50	0.623	1.28	0.48	3.38
Missed abortion/Hydatidiform mole	CD38	Wald ratio	1	-0.18	0.37	0.623	0.83	0.40	1.73
Cough	CD38	Wald ratio	1	0.12	0.24	0.626	1.13	0.70	1.81
Ulcerative colitis	CD38	Wald ratio	1	-0.11	0.23	0.627	0.89	0.57	1.41
Fracture of radius and ulna	CD38	Wald ratio	1	-0.09	0.18	0.628	0.92	0.64	1.31
Arterial embolism and thrombosis	CD38	Wald ratio	1	0.20	0.43	0.632	1.23	0.53	2.83
Other disorders of male genital organs	CD38	Wald ratio	1	-0.12	0.24	0.633	0.89	0.55	1.44

Displacement of intervertebral disc	CD38	Wald ratio	1	0.27	0.57	0.636	1.31	0.43	4.01
Bronchiectasis	CD38	Wald ratio	1	-0.14	0.30	0.636	0.87	0.48	1.56
Abnormal findings on mammogram or breast exam	CD38	Wald ratio	1	-0.15	0.33	0.637	0.86	0.45	1.63
Diverticulosis and diverticulitis	CD38	Wald ratio	1	0.04	0.09	0.637	1.04	0.88	1.23
Stricture and stenosis of esophagus	CD38	Wald ratio	1	-0.14	0.30	0.638	0.87	0.48	1.56
Ingrowing nail	CD38	Wald ratio	1	-0.19	0.42	0.640	0.82	0.36	1.86
Left bundle branch block	CD38	Wald ratio	1	0.14	0.31	0.641	1.16	0.63	2.12
Functional disorders of bladder	CD38	Wald ratio	1	0.15	0.32	0.641	1.16	0.62	2.16
Other disorders of eyelids	CD38	Wald ratio	1	0.08	0.17	0.644	1.08	0.77	1.52
Diverticulosis	CD38	Wald ratio	1	0.04	0.09	0.644	1.04	0.88	1.23
Other peripheral nerve disorders	CD38	Wald ratio	1	-0.05	0.12	0.644	0.95	0.75	1.19
Unspecified diffuse connective tissue disease	CD38	Wald ratio	1	0.11	0.25	0.647	1.12	0.69	1.83
Subarachnoid hemorrhage	CD38	Wald ratio	1	-0.21	0.45	0.648	0.81	0.33	1.98
Pilonidal cyst	CD38	Wald ratio	1	0.24	0.53	0.648	1.28	0.45	3.64
Disorder of skin and subcutaneous tissue NOS	CD38	Wald ratio	1	0.08	0.17	0.649	1.08	0.77	1.51
Uterine leiomyoma	CD38	Wald ratio	1	0.06	0.13	0.649	1.06	0.82	1.37
Strabismus (not specified as paralytic)	CD38	Wald ratio	1	0.19	0.43	0.650	1.21	0.53	2.80
Protein-calorie malnutrition	CD38	Wald ratio	1	-0.18	0.40	0.650	0.84	0.38	1.82
Chondrocalcinosis	CD38	Wald ratio	1	-0.25	0.55	0.651	0.78	0.27	2.28
Hearing loss	CD38	Wald ratio	1	-0.09	0.20	0.652	0.91	0.62	1.35
Empyema and pneumothorax	CD38	Wald ratio	1	-0.17	0.38	0.652	0.84	0.40	1.77
Paroxysmal tachycardia, unspecified	CD38	Wald ratio	1	0.10	0.23	0.652	1.11	0.71	1.74
Kyphoscoliosis and scoliosis	CD38	Wald ratio	1	-0.18	0.40	0.653	0.84	0.38	1.82
Malignant neoplasm, other	CD38	Wald ratio	1	0.05	0.10	0.654	1.05	0.85	1.29
Sarcoidosis	CD38	Wald ratio	1	-0.25	0.55	0.656	0.78	0.27	2.31
Other ill-defined and unknown causes of morbidity and mortality	CD38	Wald ratio	1	0.05	0.11	0.658	1.05	0.85	1.29

Benign neoplasm of skin	CD38	Wald ratio	1	0.07	0.15	0.659	1.07	0.80	1.43
Bundle branch block	CD38	Wald ratio	1	-0.10	0.23	0.663	0.91	0.58	1.41
Periodontitis (acute or chronic)	CD38	Wald ratio	1	0.16	0.37	0.664	1.17	0.57	2.42
Fracture of patella	CD38	Wald ratio	1	0.20	0.47	0.669	1.22	0.49	3.05
Respiratory insufficiency	CD38	Wald ratio	1	-0.13	0.31	0.671	0.88	0.48	1.61
Cancer of urinary organs (incl. kidney and bladder)	CD38	Wald ratio	1	-0.09	0.20	0.674	0.92	0.62	1.37
Ganglion and cyst of synovium, tendon, and bursa	CD38	Wald ratio	1	0.10	0.23	0.676	1.10	0.70	1.73
Paroxysmal supraventricular tachycardia	CD38	Wald ratio	1	0.11	0.27	0.677	1.12	0.66	1.89
Cataract	CD38	Wald ratio	1	-0.04	0.10	0.677	0.96	0.80	1.16
Crystal arthropathies	CD38	Wald ratio	1	-0.22	0.52	0.680	0.81	0.29	2.24
Nonspecific chest pain	CD38	Wald ratio	1	0.03	0.08	0.681	1.03	0.89	1.20
Spinal stenosis	CD38	Wald ratio	1	0.09	0.21	0.683	1.09	0.72	1.66
Hemangioma and lymphangioma, any site	CD38	Wald ratio	1	0.13	0.32	0.688	1.14	0.60	2.14
Other disorders of eye	CD38	Wald ratio	1	-0.08	0.21	0.688	0.92	0.61	1.38
Abnormal findings examination of lungs	CD38	Wald ratio	1	0.10	0.26	0.690	1.11	0.67	1.84
Type 2 diabetes	CD38	Wald ratio	1	0.04	0.10	0.691	1.04	0.85	1.27
Breast conditions, congenital or relating to hormones	CD38	Wald ratio	1	-0.17	0.42	0.693	0.85	0.37	1.93
Atrioventricular block, complete	CD38	Wald ratio	1	-0.21	0.53	0.696	0.81	0.29	2.31
Complications of labor and delivery NEC	CD38	Wald ratio	1	-0.11	0.27	0.699	0.90	0.53	1.53
Diseases of spleen	CD38	Wald ratio	1	0.22	0.57	0.699	1.25	0.41	3.81
Cellulitis and abscess of arm/hand	CD38	Wald ratio	1	-0.07	0.18	0.700	0.93	0.66	1.32
Other signs and symptoms in breast	CD38	Wald ratio	1	-0.19	0.49	0.702	0.83	0.32	2.16
Malignant neoplasm of female breast	CD38	Wald ratio	1	-0.05	0.12	0.702	0.95	0.75	1.22
Gingival and periodontal diseases	CD38	Wald ratio	1	0.12	0.31	0.703	1.13	0.61	2.06
Chronic pulmonary heart disease	CD38	Wald ratio	1	0.20	0.53	0.704	1.22	0.43	3.47
Cerebral artery occlusion, with cerebral infarction	CD38	Wald ratio	1	0.13	0.33	0.704	1.14	0.59	2.19

Other symptoms	CD38	Wald ratio	1	-0.14	0.37	0.705	0.87	0.42	1.79
Torus fracture	CD38	Wald ratio	1	-0.12	0.32	0.707	0.89	0.48	1.65
Retinal detachments and defects	CD38	Wald ratio	1	0.09	0.23	0.708	1.09	0.70	1.71
Other symptoms involving abdomen and pelvis	CD38	Wald ratio	1	0.09	0.23	0.708	1.09	0.70	1.71
Abnormal involuntary movements	CD38	Wald ratio	1	-0.16	0.43	0.709	0.85	0.36	1.99
Other disorders of tympanic membrane	CD38	Wald ratio	1	-0.13	0.35	0.710	0.88	0.44	1.75
Large cell lymphoma	CD38	Wald ratio	1	-0.20	0.54	0.710	0.82	0.28	2.35
Flatulence	CD38	Wald ratio	1	0.12	0.31	0.710	1.12	0.61	2.08
Fracture of ribs	CD38	Wald ratio	1	0.17	0.46	0.711	1.19	0.48	2.95
Cardiac arrest	CD38	Wald ratio	1	0.16	0.42	0.713	1.17	0.51	2.68
Symptoms involving female genital tract	CD38	Wald ratio	1	-0.06	0.16	0.716	0.94	0.69	1.30
Thrombocytopenia	CD38	Wald ratio	1	-0.12	0.33	0.716	0.89	0.47	1.68
Spondylosis and allied disorders	CD38	Wald ratio	1	-0.05	0.15	0.716	0.95	0.71	1.27
Vascular insufficiency of intestine	CD38	Wald ratio	1	-0.19	0.54	0.720	0.82	0.29	2.37
Fracture of lower limb	CD38	Wald ratio	1	-0.06	0.15	0.720	0.95	0.70	1.28
Obstructive chronic bronchitis	CD38	Wald ratio	1	-0.09	0.25	0.721	0.91	0.56	1.50
Cancer, suspected or other	CD38	Wald ratio	1	0.04	0.10	0.722	1.04	0.85	1.27
Diseases of lips	CD38	Wald ratio	1	0.18	0.49	0.722	1.19	0.45	3.14
Lymphoid leukemia	CD38	Wald ratio	1	-0.19	0.54	0.722	0.83	0.29	2.37
Actinic keratosis	CD38	Wald ratio	1	0.09	0.25	0.725	1.09	0.66	1.80
Diffuse diseases of connective tissue	CD38	Wald ratio	1	0.08	0.22	0.726	1.08	0.70	1.67
Breast cancer [female]	CD38	Wald ratio	1	-0.04	0.12	0.727	0.96	0.76	1.22
Arrhythmia (cardiac) NOS	CD38	Wald ratio	1	-0.15	0.43	0.728	0.86	0.37	1.99
Aphasia/speech disturbance	CD38	Wald ratio	1	0.12	0.33	0.729	1.12	0.58	2.16
Coagulation defects	CD38	Wald ratio	1	0.15	0.42	0.730	1.16	0.51	2.65
Hypotension	CD38	Wald ratio	1	-0.06	0.17	0.730	0.94	0.68	1.32

Ventral hernia	CD38	Wald ratio	1	-0.08	0.22	0.730	0.93	0.60	1.43
Hypertrophy of breast (Gynecomastia)	CD38	Wald ratio	1	-0.15	0.44	0.731	0.86	0.36	2.05
Benign neoplasm of uterus	CD38	Wald ratio	1	0.04	0.13	0.736	1.04	0.81	1.35
Other disorders of biliary tract	CD38	Wald ratio	1	0.14	0.40	0.737	1.14	0.52	2.52
Myeloproliferative disease	CD38	Wald ratio	1	-0.14	0.41	0.737	0.87	0.39	1.95
Coronary atherosclerosis	CD38	Wald ratio	1	0.03	0.10	0.739	1.03	0.85	1.26
Lump or mass in breast	CD38	Wald ratio	1	-0.11	0.33	0.739	0.90	0.47	1.72
Lymphoid leukemia, chronic	CD38	Wald ratio	1	0.19	0.57	0.739	1.21	0.39	3.73
Nonspecific abnormal findings on radiological and other examination of musculoskeletal system	CD38	Wald ratio	1	-0.16	0.49	0.741	0.85	0.33	2.22
Cellulitis and abscess of leg, except foot	CD38	Wald ratio	1	-0.06	0.18	0.741	0.94	0.67	1.33
Atherosclerosis	CD38	Wald ratio	1	0.12	0.36	0.741	1.13	0.56	2.27
Cancer of mouth	CD38	Wald ratio	1	-0.16	0.51	0.747	0.85	0.31	2.30
Other disorders of arteries and arterioles	CD38	Wald ratio	1	-0.11	0.35	0.747	0.89	0.44	1.79
Lipoma	CD38	Wald ratio	1	0.05	0.16	0.748	1.05	0.77	1.45
Acquired toe deformities	CD38	Wald ratio	1	0.06	0.18	0.749	1.06	0.74	1.52
Multiple myeloma	CD38	Wald ratio	1	0.18	0.55	0.750	1.19	0.41	3.49
Polyp of female genital organs	CD38	Wald ratio	1	0.04	0.13	0.750	1.04	0.81	1.34
Stricture/obstruction of ureter	CD38	Wald ratio	1	0.14	0.43	0.750	1.15	0.50	2.65
Chemotherapy	CD38	Wald ratio	1	0.03	0.09	0.756	1.03	0.86	1.23
Frequency of urination and polyuria	CD38	Wald ratio	1	0.06	0.21	0.756	1.07	0.71	1.59
Acute gastritis	CD38	Wald ratio	1	-0.12	0.38	0.760	0.89	0.43	1.86
Diseases of white blood cells	CD38	Wald ratio	1	-0.06	0.21	0.768	0.94	0.62	1.42
Disorders of conjunctiva	CD38	Wald ratio	1	-0.14	0.47	0.770	0.87	0.35	2.18
Stricture of artery	CD38	Wald ratio	1	-0.13	0.44	0.770	0.88	0.37	2.08
Noninflammatory disorders of vagina	CD38	Wald ratio	1	0.09	0.31	0.771	1.09	0.60	2.00

Infection of the eye	CD38	Wald ratio	1	-0.16	0.55	0.772	0.85	0.29	2.49
Inflammatory bowel disease and other gastroenteritis and colitis	CD38	Wald ratio	1	-0.06	0.20	0.773	0.95	0.64	1.39
Vascular hamartomas and non-neoplastic nevi	CD38	Wald ratio	1	-0.15	0.51	0.774	0.86	0.32	2.34
Acute pancreatitis	CD38	Wald ratio	1	-0.08	0.29	0.774	0.92	0.52	1.63
Facial nerve disorders [CN7]	CD38	Wald ratio	1	0.12	0.44	0.775	1.13	0.48	2.66
Aneurysm and dissection of heart	CD38	Wald ratio	1	0.14	0.49	0.776	1.15	0.44	2.99
Peritonitis and retroperitoneal infections	CD38	Wald ratio	1	-0.12	0.43	0.780	0.89	0.38	2.07
Malignant neoplasm of uterus	CD38	Wald ratio	1	-0.10	0.36	0.782	0.90	0.44	1.84
Effects of other external causes	CD38	Wald ratio	1	-0.06	0.20	0.783	0.95	0.64	1.40
stress incontinence, female	CD38	Wald ratio	1	-0.05	0.17	0.784	0.95	0.68	1.34
Pancreatic cancer	CD38	Wald ratio	1	0.14	0.53	0.786	1.16	0.41	3.28
Secondary malignant neoplasm	CD38	Wald ratio	1	0.04	0.14	0.788	1.04	0.80	1.35
Dislocation	CD38	Wald ratio	1	0.07	0.28	0.794	1.08	0.62	1.87
Disorders of vitreous body	CD38	Wald ratio	1	-0.09	0.35	0.795	0.91	0.46	1.81
Cancer of bronchus; lung	CD38	Wald ratio	1	0.07	0.28	0.796	1.08	0.62	1.87
Staphylococcus infections	CD38	Wald ratio	1	0.06	0.23	0.799	1.06	0.67	1.67
Emphysema	CD38	Wald ratio	1	-0.08	0.31	0.803	0.93	0.50	1.71
Obesity	CD38	Wald ratio	1	0.03	0.13	0.804	1.03	0.80	1.33
Osteomyelitis	CD38	Wald ratio	1	-0.13	0.54	0.805	0.87	0.30	2.53
Osteoarthritis; localized	CD38	Wald ratio	1	0.02	0.10	0.809	1.02	0.84	1.25
Ill-defined descriptions and complications of heart disease	CD38	Wald ratio	1	0.11	0.46	0.810	1.12	0.46	2.74
Respiratory abnormalities	CD38	Wald ratio	1	0.13	0.52	0.811	1.13	0.41	3.16
Secondary malignant neoplasm of liver	CD38	Wald ratio	1	0.06	0.25	0.811	1.06	0.65	1.75
Neuralgia, neuritis, and radiculitis NOS	CD38	Wald ratio	1	-0.09	0.38	0.811	0.91	0.44	1.91
Back pain	CD38	Wald ratio	1	-0.03	0.13	0.812	0.97	0.76	1.24
Labyrinthitis	CD38	Wald ratio	1	-0.11	0.47	0.814	0.90	0.36	2.24

Internal derangement of knee	CD38	Wald ratio	1	0.03	0.11	0.815	1.03	0.83	1.27
Hemorrhoids	CD38	Wald ratio	1	-0.02	0.09	0.815	0.98	0.82	1.16
Schizophrenia	CD38	Wald ratio	1	0.13	0.54	0.816	1.13	0.39	3.27
Cellulitis and abscess of foot, toe	CD38	Wald ratio	1	-0.04	0.18	0.816	0.96	0.68	1.36
Gastrointestinal hemorrhage	CD38	Wald ratio	1	-0.02	0.09	0.817	0.98	0.82	1.17
Hemorrhage from gastrointestinal ulcer	CD38	Wald ratio	1	-0.12	0.52	0.818	0.89	0.32	2.46
Breast cancer	CD38	Wald ratio	1	-0.03	0.12	0.824	0.97	0.77	1.23
Chronic liver disease and cirrhosis	CD38	Wald ratio	1	-0.05	0.24	0.826	0.95	0.59	1.52
Senile cataract	CD38	Wald ratio	1	-0.03	0.15	0.828	0.97	0.73	1.29
Hemorrhage in early pregnancy	CD38	Wald ratio	1	0.09	0.40	0.830	1.09	0.50	2.38
Other disorders of urethra and urinary tract	CD38	Wald ratio	1	0.04	0.20	0.830	1.04	0.70	1.56
Diseases of pancreas	CD38	Wald ratio	1	-0.05	0.25	0.830	0.95	0.58	1.55
Primary angle-closure glaucoma	CD38	Wald ratio	1	0.10	0.49	0.831	1.11	0.43	2.89
Cancer of larynx, pharynx, nasal cavities	CD38	Wald ratio	1	0.11	0.51	0.831	1.12	0.41	3.06
Pruritus and related conditions	CD38	Wald ratio	1	-0.10	0.46	0.832	0.91	0.37	2.24
Nonspecific abnormal findings on radiological and other examination of other intrathoracic organs (echocardiogram, etc)	CD38	Wald ratio	1	-0.11	0.54	0.834	0.89	0.31	2.59
Heart valve replaced	CD38	Wald ratio	1	0.07	0.33	0.840	1.07	0.56	2.06
Blood in stool	CD38	Wald ratio	1	-0.05	0.25	0.840	0.95	0.58	1.56
Cancer of kidney and renal pelvis	CD38	Wald ratio	1	-0.08	0.40	0.842	0.92	0.42	2.02
Cardiac arrest and ventricular fibrillation	CD38	Wald ratio	1	0.08	0.38	0.843	1.08	0.51	2.29
Complication due to other implant and internal device	CD38	Wald ratio	1	-0.05	0.23	0.843	0.96	0.61	1.49
Diseases of sebaceous glands	CD38	Wald ratio	1	-0.03	0.14	0.844	0.97	0.74	1.28
Chronic sinusitis	CD38	Wald ratio	1	0.05	0.25	0.845	1.05	0.64	1.73
Hemorrhage during pregnancy; childbirth and postpartum	CD38	Wald ratio	1	-0.06	0.30	0.846	0.94	0.52	1.70
Inflammatory disease of breast	CD38	Wald ratio	1	0.09	0.48	0.846	1.10	0.43	2.82

Nevus, non-neoplastic	CD38	Wald ratio	1	-0.10	0.53	0.846	0.90	0.32	2.55
Malignant neoplasm of testis	CD38	Wald ratio	1	-0.05	0.24	0.847	0.95	0.60	1.53
Diseases of the salivary glands	CD38	Wald ratio	1	-0.09	0.48	0.847	0.91	0.35	2.35
Altered mental status	CD38	Wald ratio	1	-0.05	0.28	0.847	0.95	0.55	1.63
Cystic mastopathy	CD38	Wald ratio	1	0.08	0.43	0.848	1.09	0.47	2.50
Arterial embolism and thrombosis of lower extremity artery	CD38	Wald ratio	1	0.10	0.55	0.851	1.11	0.38	3.23
Digestive congenital anomalies	CD38	Wald ratio	1	-0.09	0.49	0.853	0.91	0.35	2.37
Other paralytic syndromes	CD38	Wald ratio	1	-0.09	0.50	0.854	0.91	0.34	2.44
Glaucoma	CD38	Wald ratio	1	0.03	0.20	0.861	1.03	0.70	1.52
Musculoskeletal symptoms referable to limbs	CD38	Wald ratio	1	-0.03	0.18	0.862	0.97	0.69	1.37
Pneumonia	CD38	Wald ratio	1	-0.02	0.13	0.863	0.98	0.76	1.27
Syncope and collapse	CD38	Wald ratio	1	-0.02	0.14	0.866	0.98	0.75	1.28
Sebaceous cyst	CD38	Wald ratio	1	-0.02	0.14	0.867	0.98	0.74	1.28
Miscarriage; stillbirth	CD38	Wald ratio	1	-0.03	0.19	0.868	0.97	0.67	1.40
Epistaxis or throat hemorrhage	CD38	Wald ratio	1	-0.04	0.26	0.869	0.96	0.57	1.60
Urethral stricture (not specified as infectious)	CD38	Wald ratio	1	-0.04	0.23	0.869	0.96	0.62	1.50
Ileostomy status	CD38	Wald ratio	1	0.05	0.32	0.870	1.05	0.56	1.97
Intracranial hemorrhage (injury)	CD38	Wald ratio	1	0.09	0.57	0.872	1.10	0.36	3.37
Umbilical cord complications during labor and delivery	CD38	Wald ratio	1	-0.09	0.57	0.872	0.91	0.30	2.77
Other disorders of soft tissues	CD38	Wald ratio	1	0.03	0.17	0.874	1.03	0.74	1.42
Inguinal hernia	CD38	Wald ratio	1	0.02	0.11	0.875	1.02	0.82	1.26
Diseases of hair and hair follicles	CD38	Wald ratio	1	-0.03	0.18	0.875	0.97	0.68	1.38
Leukemia	CD38	Wald ratio	1	0.05	0.32	0.876	1.05	0.56	1.96
Respiratory failure, insufficiency, arrest	CD38	Wald ratio	1	0.04	0.26	0.877	1.04	0.63	1.72
Bipolar	CD38	Wald ratio	1	-0.06	0.40	0.878	0.94	0.43	2.05
Disorders of lacrimal system	CD38	Wald ratio	1	0.04	0.28	0.878	1.04	0.61	1.79

Constipation	CD38	Wald ratio	1	0.02	0.13	0.878	1.02	0.79	1.32
Poisoning by analgesics, antipyretics, and antirheumatics	CD38	Wald ratio	1	-0.02	0.16	0.879	0.98	0.72	1.33
Ulcer of esophagus	CD38	Wald ratio	1	0.03	0.18	0.881	1.03	0.72	1.47
Placenta previa and abruptio placenta	CD38	Wald ratio	1	-0.06	0.37	0.881	0.95	0.46	1.96
Benign neoplasm of unspecified sites	CD38	Wald ratio	1	0.04	0.26	0.884	1.04	0.62	1.74
Orthostatic hypotension	CD38	Wald ratio	1	0.05	0.35	0.885	1.05	0.53	2.10
Complications of surgical and medical procedures	CD38	Wald ratio	1	-0.02	0.14	0.886	0.98	0.75	1.28
Viral Enteritis	CD38	Wald ratio	1	-0.06	0.44	0.892	0.94	0.40	2.24
Lymphadenitis	CD38	Wald ratio	1	-0.03	0.25	0.893	0.97	0.59	1.58
Disorders of parathyroid gland	CD38	Wald ratio	1	-0.06	0.44	0.893	0.94	0.40	2.22
Other nonmalignant breast conditions	CD38	Wald ratio	1	-0.04	0.29	0.899	0.96	0.54	1.71
Convulsions	CD38	Wald ratio	1	-0.03	0.27	0.901	0.97	0.56	1.66
Intracranial hemorrhage	CD38	Wald ratio	1	-0.04	0.31	0.906	0.96	0.53	1.76
Intervertebral disc disorders	CD38	Wald ratio	1	-0.02	0.14	0.909	0.98	0.75	1.29
Disorders of adrenal glands	CD38	Wald ratio	1	0.06	0.51	0.909	1.06	0.39	2.88
Other upper respiratory disease	CD38	Wald ratio	1	0.02	0.20	0.909	1.02	0.69	1.52
Disease of tricuspid valve	CD38	Wald ratio	1	-0.04	0.40	0.914	0.96	0.44	2.08
Paroxysmal ventricular tachycardia	CD38	Wald ratio	1	-0.04	0.42	0.916	0.96	0.42	2.19
Nerve root and plexus disorders	CD38	Wald ratio	1	-0.04	0.38	0.916	0.96	0.45	2.03
Calculus of bile duct	CD38	Wald ratio	1	-0.03	0.25	0.918	0.97	0.59	1.60
Periapical abscess	CD38	Wald ratio	1	-0.04	0.38	0.918	0.96	0.46	2.01
Cervicalgia	CD38	Wald ratio	1	-0.03	0.31	0.918	0.97	0.53	1.76
Acquired absence of breast	CD38	Wald ratio	1	-0.03	0.27	0.921	0.97	0.58	1.64
Congenital anomalies of great vessels	CD38	Wald ratio	1	-0.03	0.31	0.921	0.97	0.53	1.77
Fracture of tibia and fibula	CD38	Wald ratio	1	0.03	0.28	0.921	1.03	0.59	1.78
Acute pharyngitis	CD38	Wald ratio	1	0.04	0.44	0.922	1.04	0.44	2.46

Other diseases of lung	CD38	Wald ratio	1	-0.05	0.46	0.923	0.96	0.39	2.37
Pernicious anemia	CD38	Wald ratio	1	0.05	0.47	0.923	1.05	0.42	2.64
Swelling of limb	CD38	Wald ratio	1	-0.02	0.19	0.924	0.98	0.68	1.43
Cervical intraepithelial neoplasia [CIN] [Cervical dysplasia]	CD38	Wald ratio	1	0.03	0.28	0.925	1.03	0.59	1.79
Abdominal pain	CD38	Wald ratio	1	-0.01	0.07	0.927	0.99	0.87	1.13
Overweight, obesity and other hyperalimentation	CD38	Wald ratio	1	0.01	0.13	0.929	1.01	0.79	1.30
Candidiasis	CD38	Wald ratio	1	-0.02	0.28	0.930	0.98	0.56	1.69
Corneal opacity and other disorders of cornea	CD38	Wald ratio	1	0.04	0.48	0.930	1.04	0.41	2.66
Other mental disorder	CD38	Wald ratio	1	0.01	0.08	0.934	1.01	0.86	1.18
Chronic airway obstruction	CD38	Wald ratio	1	-0.01	0.13	0.934	0.99	0.77	1.28
Other dyschromia	CD38	Wald ratio	1	-0.04	0.46	0.936	0.96	0.39	2.38
Epilepsy, recurrent seizures, convulsions	CD38	Wald ratio	1	0.01	0.18	0.938	1.01	0.71	1.45
Other forms of chronic heart disease	CD38	Wald ratio	1	-0.02	0.31	0.940	0.98	0.54	1.78
Malignant neoplasm of kidney, except pelvis	CD38	Wald ratio	1	-0.03	0.41	0.942	0.97	0.44	2.16
Thyrotoxicosis with or without goiter	CD38	Wald ratio	1	-0.02	0.30	0.943	0.98	0.54	1.77
Vitamin B-complex deficiencies	CD38	Wald ratio	1	-0.03	0.47	0.945	0.97	0.38	2.44
Other hypertrophic and atrophic conditions of skin	CD38	Wald ratio	1	0.01	0.19	0.947	1.01	0.70	1.46
Chronic tonsillitis and adenoiditis	CD38	Wald ratio	1	-0.02	0.39	0.949	0.98	0.46	2.08
Amblyopia	CD38	Wald ratio	1	0.03	0.56	0.951	1.03	0.35	3.08
Chronic ulcer of skin	CD38	Wald ratio	1	-0.02	0.29	0.954	0.98	0.56	1.73
Secondary malignant neoplasm of digestive systems	CD38	Wald ratio	1	0.02	0.33	0.954	1.02	0.53	1.95
Septicemia	CD38	Wald ratio	1	-0.01	0.20	0.955	0.99	0.66	1.47
Disorders of other cranial nerves	CD38	Wald ratio	1	-0.02	0.35	0.958	0.98	0.50	1.94
Symptoms of the muscles	CD38	Wald ratio	1	0.03	0.49	0.959	1.03	0.39	2.67
Ulceration of intestine	CD38	Wald ratio	1	-0.03	0.50	0.959	0.98	0.37	2.59
Early or threatened labor; hemorrhage in early pregnancy	CD38	Wald ratio	1	0.01	0.27	0.960	1.01	0.60	1.71

Hypertrophy of female genital organs	CD38	Wald ratio	1	0.02	0.36	0.961	1.02	0.50	2.08
Pain	CD38	Wald ratio	1	-0.02	0.46	0.961	0.98	0.39	2.43
Polyp of corpus uteri	CD38	Wald ratio	1	-0.01	0.15	0.963	0.99	0.74	1.33
Dyschromia and Vitiligo	CD38	Wald ratio	1	0.02	0.41	0.964	1.02	0.45	2.28
Antepartum hemorrhage, abruptio placentae, and placenta previa	CD38	Wald ratio	1	-0.02	0.45	0.964	0.98	0.41	2.35
Endometriosis	CD38	Wald ratio	1	-0.01	0.21	0.967	0.99	0.66	1.49
Strabismus and other disorders of binocular eye movements	CD38	Wald ratio	1	0.01	0.34	0.970	1.01	0.52	1.97
Viral infection	CD38	Wald ratio	1	-0.01	0.24	0.972	0.99	0.61	1.60
E. coli	CD38	Wald ratio	1	0.01	0.25	0.977	1.01	0.62	1.64
Congenital anomalies of urinary system	CD38	Wald ratio	1	0.01	0.44	0.982	1.01	0.42	2.40
Type 1 diabetes	CD38	Wald ratio	1	0.00	0.25	0.985	1.00	0.61	1.65
Other abnormality of urination	CD38	Wald ratio	1	0.01	0.30	0.986	1.01	0.56	1.79
Acute periodontitis	CD38	Wald ratio	1	0.01	0.50	0.986	1.01	0.38	2.69
Rheumatoid arthritis	CD38	Wald ratio	1	0.00	0.20	0.987	1.00	0.68	1.48
Purpura and other hemorrhagic conditions	CD38	Wald ratio	1	0.00	0.30	0.989	1.00	0.55	1.83
Early onset of delivery	CD38	Wald ratio	1	-0.01	0.53	0.989	0.99	0.35	2.79
Other cerebral degenerations	CD38	Wald ratio	1	0.01	0.39	0.989	1.01	0.47	2.15
Epilepsy	CD38	Wald ratio	1	0.00	0.43	0.996	1.00	0.43	2.31
Rheumatoid arthritis and other inflammatory polyarthropathies	CD38	Wald ratio	1	0.00	0.19	0.996	1.00	0.69	1.44

Supplementary Table 7: Phenome-wide MR results for brain DGKQ.

outcome	exposure	method	nsnp	b	se	pval	or	or_lci95	or_uci95
Dysmenorrhea	DGKQ	Wald ratio	1	-2.14	0.67	1.423E-03	0.12	0.03	0.44
Pain in joint	DGKQ	Wald ratio	1	0.95	0.32	2.633E-03	2.59	1.39	4.82
Internal derangement of knee	DGKQ	Wald ratio	1	0.69	0.23	3.191E-03	1.99	1.26	3.15
Anaphylactic shock NOS	DGKQ	Wald ratio	1	-3.31	1.20	5.683E-03	0.04	0.00	0.38
Other arthropathies	DGKQ	Wald ratio	1	0.41	0.16	8.836E-03	1.51	1.11	2.05
Ganglion and cyst of synovium, tendon, and bursa	DGKQ	Wald ratio	1	1.28	0.50	9.928E-03	3.61	1.36	9.58
Acute gastritis	DGKQ	Wald ratio	1	2.09	0.82	0.011	8.07	1.63	39.93
Other disorders of synovium, tendon, and bursa	DGKQ	Wald ratio	1	0.83	0.33	0.011	2.28	1.21	4.32
Arthropathy NOS	DGKQ	Wald ratio	1	0.41	0.16	0.011	1.50	1.10	2.06
Tinnitus	DGKQ	Wald ratio	1	-3.06	1.24	0.013	0.05	0.00	0.53
Unspecified diffuse connective tissue disease	DGKQ	Wald ratio	1	1.32	0.54	0.014	3.74	1.30	10.76
Unspecified monoarthritis	DGKQ	Wald ratio	1	0.57	0.23	0.015	1.77	1.12	2.80
Poisoning by antibiotics	DGKQ	Wald ratio	1	0.52	0.21	0.016	1.68	1.10	2.56
Congestive heart failure; nonhypertensive	DGKQ	Wald ratio	1	0.92	0.39	0.018	2.51	1.17	5.37
Calculus of kidney	DGKQ	Wald ratio	1	-1.18	0.50	0.018	0.31	0.12	0.81
Disorders of conjunctiva	DGKQ	Wald ratio	1	2.39	1.01	0.018	10.89	1.50	79.26
Nonspecific chest pain	DGKQ	Wald ratio	1	0.39	0.17	0.020	1.48	1.07	2.07
Acquired spondylolisthesis	DGKQ	Wald ratio	1	1.62	0.72	0.024	5.05	1.24	20.57
Symptoms of the muscles	DGKQ	Wald ratio	1	-2.38	1.06	0.025	0.09	0.01	0.74
Benign mammary dysplasias	DGKQ	Wald ratio	1	1.09	0.49	0.025	2.97	1.14	7.71
Allergy/adverse effect of penicillin	DGKQ	Wald ratio	1	0.50	0.23	0.030	1.65	1.05	2.58
Nausea and vomiting	DGKQ	Wald ratio	1	0.57	0.26	0.030	1.77	1.06	2.98
Disorder of skin and subcutaneous tissue NOS	DGKQ	Wald ratio	1	0.80	0.37	0.030	2.23	1.08	4.58
Coagulation defects	DGKQ	Wald ratio	1	-1.98	0.91	0.030	0.14	0.02	0.83

Asthma	DGKQ	Wald ratio	1	0.40	0.19	0.032	1.49	1.03	2.14
Displacement of intervertebral disc	DGKQ	Wald ratio	1	2.64	1.24	0.033	14.01	1.24	157.93
Malignant neoplasm of other and ill-defined sites within the digestive organs and peritoneum	DGKQ	Wald ratio	1	0.81	0.38	0.033	2.25	1.07	4.72
Other conditions of brain	DGKQ	Wald ratio	1	1.57	0.74	0.034	4.82	1.13	20.55
Disorders of menstruation and other abnormal bleeding from female genital tract	DGKQ	Wald ratio	1	-0.47	0.22	0.035	0.62	0.40	0.97
Glaucoma	DGKQ	Wald ratio	1	-0.89	0.42	0.035	0.41	0.18	0.94
Other specified benign mammary dysplasias	DGKQ	Wald ratio	1	2.22	1.06	0.036	9.23	1.16	73.50
Atherosclerosis of the extremities	DGKQ	Wald ratio	1	2.06	0.99	0.036	7.88	1.14	54.44
Dyspareunia	DGKQ	Wald ratio	1	-1.70	0.81	0.037	0.18	0.04	0.90
Staphylococcus infections	DGKQ	Wald ratio	1	1.04	0.50	0.038	2.83	1.06	7.54
Mechanical complication of unspecified genitourinary device, implant, and graft	DGKQ	Wald ratio	1	-1.64	0.80	0.039	0.19	0.04	0.92
Heartburn	DGKQ	Wald ratio	1	1.25	0.61	0.041	3.48	1.05	11.50
Secondary malignant neoplasm	DGKQ	Wald ratio	1	0.60	0.29	0.041	1.82	1.02	3.23
Hemorrhoids	DGKQ	Wald ratio	1	0.39	0.19	0.042	1.48	1.01	2.14
Symptoms concerning nutrition, metabolism, and development	DGKQ	Wald ratio	1	0.78	0.38	0.043	2.17	1.03	4.60
Other acquired musculoskeletal deformity	DGKQ	Wald ratio	1	1.28	0.64	0.044	3.61	1.03	12.62
Other tests	DGKQ	Wald ratio	1	0.73	0.37	0.045	2.08	1.02	4.27
Megaloblastic anemia	DGKQ	Wald ratio	1	-1.68	0.85	0.049	0.19	0.04	0.99
Diffuse diseases of connective tissue	DGKQ	Wald ratio	1	0.94	0.48	0.051	2.55	1.00	6.53
Uterine/Uterovaginal prolapse	DGKQ	Wald ratio	1	0.76	0.39	0.052	2.13	0.99	4.56
Gangrene	DGKQ	Wald ratio	1	2.32	1.19	0.052	10.13	0.98	104.46
Disorders of muscle, ligament, and fascia	DGKQ	Wald ratio	1	0.83	0.42	0.052	2.28	0.99	5.25
Secondary malignancy of brain/spine	DGKQ	Wald ratio	1	1.91	0.99	0.053	6.74	0.98	46.46

Poisoning/allergy of sulfonamides	DGKQ	Wald ratio	1	1.82	0.95	0.054	6.20	0.97	39.53
Peripheral enthesopathies and allied syndromes	DGKQ	Wald ratio	1	0.45	0.24	0.055	1.57	0.99	2.49
Prolapse of vaginal walls	DGKQ	Wald ratio	1	-0.63	0.33	0.057	0.53	0.28	1.02
Heart failure NOS	DGKQ	Wald ratio	1	0.82	0.43	0.059	2.27	0.97	5.32
Strabismus (not specified as paralytic)	DGKQ	Wald ratio	1	1.74	0.92	0.059	5.70	0.93	34.76
Pain and other symptoms associated with female genital organs	DGKQ	Wald ratio	1	-0.97	0.52	0.061	0.38	0.14	1.04
Cyst or abscess of Bartholin's gland	DGKQ	Wald ratio	1	1.90	1.02	0.062	6.68	0.91	49.06
Secondary malignant neoplasm of liver	DGKQ	Wald ratio	1	1.02	0.55	0.064	2.76	0.94	8.09
Abnormal findings on examination of urine	DGKQ	Wald ratio	1	-0.86	0.47	0.066	0.42	0.17	1.06
Diabetic retinopathy	DGKQ	Wald ratio	1	1.40	0.77	0.067	4.07	0.91	18.30
Pyelonephritis	DGKQ	Wald ratio	1	-1.40	0.77	0.068	0.25	0.05	1.11
Benign neoplasm of breast	DGKQ	Wald ratio	1	1.29	0.71	0.071	3.62	0.90	14.61
Subarachnoid hemorrhage	DGKQ	Wald ratio	1	1.75	0.98	0.074	5.77	0.84	39.38
Torus fracture	DGKQ	Wald ratio	1	1.21	0.69	0.077	3.36	0.88	12.87
Cataract	DGKQ	Wald ratio	1	0.37	0.21	0.077	1.44	0.96	2.17
Nonspecific abnormal findings on radiological and other examination of musculoskeletal system	DGKQ	Wald ratio	1	1.86	1.06	0.078	6.42	0.81	50.88
Paroxysmal ventricular tachycardia	DGKQ	Wald ratio	1	1.61	0.92	0.079	4.99	0.83	30.10
Varicose veins of lower extremity, symptomtic	DGKQ	Wald ratio	1	1.94	1.11	0.080	6.99	0.80	61.37
Genitourinary congenital anomalies	DGKQ	Wald ratio	1	1.22	0.70	0.080	3.40	0.86	13.40
Cancer of larynx, pharynx, nasal cavities	DGKQ	Wald ratio	1	-1.94	1.11	0.081	0.14	0.02	1.27
Fracture of tibia and fibula	DGKQ	Wald ratio	1	1.05	0.60	0.082	2.86	0.87	9.36
Symptoms and disorders of the joints	DGKQ	Wald ratio	1	0.80	0.46	0.083	2.23	0.90	5.54
Other deficiency anemia	DGKQ	Wald ratio	1	-1.44	0.83	0.083	0.24	0.05	1.21
Chronic pancreatitis	DGKQ	Wald ratio	1	-2.14	1.24	0.084	0.12	0.01	1.33
Senile cataract	DGKQ	Wald ratio	1	0.54	0.32	0.085	1.72	0.93	3.20

Aneurysm and dissection of heart	DGKQ	Wald ratio	1	-1.80	1.06	0.088	0.17	0.02	1.31
Syncope and collapse	DGKQ	Wald ratio	1	0.50	0.30	0.091	1.65	0.92	2.96
Nonspecific abnormal findings on radiological and other examination of other intrathoracic organs (echocardiogram, etc)	DGKQ	Wald ratio	1	1.98	1.17	0.092	7.24	0.73	72.23
Diseases of white blood cells	DGKQ	Wald ratio	1	-0.76	0.45	0.092	0.47	0.19	1.13
Irregular menstrual bleeding	DGKQ	Wald ratio	1	-0.78	0.46	0.092	0.46	0.19	1.14
Joint effusions	DGKQ	Wald ratio	1	1.24	0.74	0.095	3.44	0.81	14.65
Cholelithiasis with other cholecystitis	DGKQ	Wald ratio	1	-0.64	0.39	0.096	0.53	0.25	1.12
Fracture of clavicle or scapula	DGKQ	Wald ratio	1	1.19	0.72	0.096	3.30	0.81	13.44
Contracture of palmar fascia [Dupuytren's disease]	DGKQ	Wald ratio	1	0.80	0.48	0.097	2.23	0.86	5.73
Disease of tricuspid valve	DGKQ	Wald ratio	1	1.42	0.86	0.099	4.12	0.77	22.14
Congestive heart failure (CHF) NOS	DGKQ	Wald ratio	1	1.03	0.63	0.100	2.80	0.82	9.52
Other disorders of male genital organs	DGKQ	Wald ratio	1	0.87	0.53	0.102	2.38	0.84	6.70
Enthesopathy	DGKQ	Wald ratio	1	0.47	0.29	0.103	1.61	0.91	2.84
Other diseases of blood and blood-forming organs	DGKQ	Wald ratio	1	0.70	0.44	0.106	2.02	0.86	4.75
Burns	DGKQ	Wald ratio	1	-1.87	1.16	0.107	0.15	0.02	1.50
Urinary tract infection	DGKQ	Wald ratio	1	-0.41	0.25	0.108	0.67	0.41	1.09
Neuralgia, neuritis, and radiculitis NOS	DGKQ	Wald ratio	1	-1.31	0.81	0.108	0.27	0.05	1.34
Secondary malignancy of respiratory organs	DGKQ	Wald ratio	1	0.95	0.60	0.110	2.60	0.80	8.37
Lipoma of skin and subcutaneous tissue	DGKQ	Wald ratio	1	-0.66	0.42	0.111	0.52	0.23	1.16
Dysuria	DGKQ	Wald ratio	1	1.27	0.81	0.115	3.57	0.73	17.33
Cervical cancer	DGKQ	Wald ratio	1	-1.09	0.69	0.116	0.34	0.09	1.31
Secondary hypothyroidism	DGKQ	Wald ratio	1	-1.32	0.84	0.116	0.27	0.05	1.39
Secondary malignancy of bone	DGKQ	Wald ratio	1	0.94	0.60	0.117	2.57	0.79	8.37
Cellulitis and abscess of trunk	DGKQ	Wald ratio	1	-1.78	1.14	0.119	0.17	0.02	1.58
Acute periodontitis	DGKQ	Wald ratio	1	1.68	1.08	0.121	5.36	0.64	44.75

Pruritus and related conditions	DGKQ	Wald ratio	1	-1.54	1.00	0.123	0.22	0.03	1.52
Other venous embolism and thrombosis	DGKQ	Wald ratio	1	1.80	1.18	0.127	6.05	0.60	61.05
Other benign neoplasm of connective and other soft tissue	DGKQ	Wald ratio	1	-1.27	0.84	0.129	0.28	0.05	1.45
Congenital anomalies of genital organs	DGKQ	Wald ratio	1	1.57	1.04	0.129	4.82	0.63	36.74
Other disorders of cervical region	DGKQ	Wald ratio	1	-1.85	1.22	0.131	0.16	0.01	1.73
Fracture of lower limb	DGKQ	Wald ratio	1	0.50	0.33	0.131	1.65	0.86	3.17
Pulmonary heart disease	DGKQ	Wald ratio	1	0.65	0.43	0.134	1.91	0.82	4.46
Chronic pulmonary heart disease	DGKQ	Wald ratio	1	1.72	1.15	0.136	5.56	0.58	53.05
Diseases of pancreas	DGKQ	Wald ratio	1	-0.80	0.54	0.139	0.45	0.15	1.30
Inguinal hernia	DGKQ	Wald ratio	1	-0.35	0.24	0.141	0.71	0.44	1.12
Miscarriage; stillbirth	DGKQ	Wald ratio	1	0.60	0.41	0.142	1.82	0.82	4.05
Inflammatory bowel disease and other gastroenteritis and colitis	DGKQ	Wald ratio	1	0.61	0.42	0.147	1.85	0.81	4.22
Primary/intrinsic cardiomyopathies	DGKQ	Wald ratio	1	1.17	0.81	0.148	3.22	0.66	15.67
Cardiomyopathy	DGKQ	Wald ratio	1	1.15	0.80	0.149	3.15	0.66	14.99
Fasciitis	DGKQ	Wald ratio	1	0.66	0.46	0.150	1.94	0.79	4.77
Pernicious anemia	DGKQ	Wald ratio	1	-1.46	1.02	0.151	0.23	0.03	1.71
Other congenital musculoskeletal anomalies	DGKQ	Wald ratio	1	1.64	1.15	0.152	5.18	0.55	49.02
Epilepsy, recurrent seizures, convulsions	DGKQ	Wald ratio	1	0.57	0.40	0.153	1.76	0.81	3.83
Primary angle-closure glaucoma	DGKQ	Wald ratio	1	-1.50	1.05	0.155	0.22	0.03	1.76
Contusion	DGKQ	Wald ratio	1	-1.02	0.72	0.159	0.36	0.09	1.49
Vascular hamartomas and non-neoplastic nevi	DGKQ	Wald ratio	1	1.55	1.10	0.159	4.70	0.55	40.53
Postinflammatory pulmonary fibrosis	DGKQ	Wald ratio	1	-1.32	0.94	0.160	0.27	0.04	1.68
Nasal polyps	DGKQ	Wald ratio	1	-0.69	0.49	0.160	0.50	0.19	1.31
Osteoarthrosis	DGKQ	Wald ratio	1	0.25	0.18	0.161	1.29	0.91	1.82
Coronary atherosclerosis	DGKQ	Wald ratio	1	-0.30	0.22	0.162	0.74	0.48	1.13
Strabismus and other disorders of binocular eye movements	DGKQ	Wald ratio	1	1.02	0.74	0.164	2.78	0.66	11.77

Bacterial enteritis	DGKQ	Wald ratio	1	0.74	0.54	0.164	2.10	0.74	6.01
Cellulitis and abscess of arm/hand	DGKQ	Wald ratio	1	0.53	0.38	0.165	1.69	0.81	3.56
Other specified cardiac dysrhythmias	DGKQ	Wald ratio	1	0.69	0.49	0.166	1.98	0.75	5.23
Other headache syndromes	DGKQ	Wald ratio	1	0.44	0.32	0.166	1.56	0.83	2.92
Rupture of synovium	DGKQ	Wald ratio	1	1.58	1.14	0.166	4.87	0.52	45.95
Stiffness of joint	DGKQ	Wald ratio	1	1.55	1.12	0.169	4.70	0.52	42.59
Decreased white blood cell count	DGKQ	Wald ratio	1	-0.68	0.50	0.170	0.51	0.19	1.34
Neutropenia	DGKQ	Wald ratio	1	-0.68	0.50	0.170	0.51	0.19	1.34
Reflux esophagitis	DGKQ	Wald ratio	1	0.38	0.28	0.172	1.46	0.85	2.53
Other ill-defined and unknown causes of morbidity and mortality	DGKQ	Wald ratio	1	-0.31	0.23	0.173	0.73	0.47	1.15
Lipoma	DGKQ	Wald ratio	1	-0.48	0.35	0.174	0.62	0.31	1.24
Precordial pain	DGKQ	Wald ratio	1	0.64	0.47	0.175	1.89	0.75	4.76
Superficial cellulitis and abscess	DGKQ	Wald ratio	1	0.44	0.33	0.178	1.56	0.82	2.97
Bronchitis	DGKQ	Wald ratio	1	1.49	1.11	0.181	4.43	0.50	39.08
Diseases of sebaceous glands	DGKQ	Wald ratio	1	-0.40	0.30	0.182	0.67	0.37	1.21
Cancer of other lymphoid, histiocytic tissue	DGKQ	Wald ratio	1	0.79	0.59	0.182	2.19	0.69	6.96
Diseases of the larynx and vocal cords	DGKQ	Wald ratio	1	-0.72	0.54	0.186	0.49	0.17	1.42
Other disorders of bone and cartilage	DGKQ	Wald ratio	1	0.60	0.46	0.186	1.83	0.75	4.46
Irregular menstrual cycle/bleeding	DGKQ	Wald ratio	1	-0.31	0.24	0.189	0.73	0.46	1.17
Emphysema	DGKQ	Wald ratio	1	-0.89	0.68	0.189	0.41	0.11	1.55
Crystal arthropathies	DGKQ	Wald ratio	1	1.48	1.13	0.190	4.37	0.48	39.81
Hemorrhage of gastrointestinal tract	DGKQ	Wald ratio	1	-0.50	0.39	0.192	0.60	0.28	1.29
Other mental disorder	DGKQ	Wald ratio	1	0.23	0.18	0.192	1.26	0.89	1.79
Intracranial hemorrhage	DGKQ	Wald ratio	1	0.86	0.66	0.193	2.37	0.65	8.68
Back pain	DGKQ	Wald ratio	1	0.35	0.27	0.193	1.42	0.84	2.41
Viral warts & HPV	DGKQ	Wald ratio	1	-1.14	0.88	0.194	0.32	0.06	1.79

Cancer of brain and nervous system	DGKQ	Wald ratio	1	-1.57	1.21	0.195	0.21	0.02	2.23
Poisoning by other anti-infectives	DGKQ	Wald ratio	1	1.02	0.79	0.196	2.77	0.59	13.03
Malposition and malpresentation of fetus or obstruction	DGKQ	Wald ratio	1	-0.90	0.70	0.197	0.41	0.10	1.59
Symptoms involving female genital tract	DGKQ	Wald ratio	1	0.45	0.35	0.198	1.57	0.79	3.13
Palpitations	DGKQ	Wald ratio	1	-0.59	0.46	0.199	0.56	0.23	1.36
Disorders of uterus, NEC	DGKQ	Wald ratio	1	-0.62	0.49	0.201	0.54	0.21	1.39
Cyst of kidney, acquired	DGKQ	Wald ratio	1	1.01	0.79	0.201	2.74	0.59	12.80
Noninflammatory disorders of vulva and perineum	DGKQ	Wald ratio	1	0.97	0.76	0.202	2.65	0.59	11.84
Curvature of spine	DGKQ	Wald ratio	1	-1.06	0.83	0.202	0.35	0.07	1.77
Mucous polyp of cervix	DGKQ	Wald ratio	1	0.61	0.48	0.202	1.84	0.72	4.72
Nonspecific findings on examination of blood	DGKQ	Wald ratio	1	0.45	0.35	0.203	1.56	0.79	3.11
Diseases of hair and hair follicles	DGKQ	Wald ratio	1	-0.49	0.39	0.204	0.61	0.29	1.31
Diseases of esophagus	DGKQ	Wald ratio	1	0.20	0.16	0.207	1.22	0.89	1.68
Neoplasm of uncertain behavior	DGKQ	Wald ratio	1	0.93	0.74	0.211	2.53	0.59	10.87
Hypertrophy of female genital organs	DGKQ	Wald ratio	1	0.99	0.79	0.211	2.68	0.57	12.52
Esophagitis, GERD and related diseases	DGKQ	Wald ratio	1	0.21	0.17	0.214	1.23	0.89	1.71
Polyp of corpus uteri	DGKQ	Wald ratio	1	-0.40	0.32	0.216	0.67	0.36	1.26
Noninflammatory disorders of cervix	DGKQ	Wald ratio	1	0.65	0.53	0.219	1.91	0.68	5.36
Lymphadenitis	DGKQ	Wald ratio	1	0.67	0.55	0.221	1.95	0.67	5.69
Chondrocalcinosis	DGKQ	Wald ratio	1	1.44	1.18	0.223	4.22	0.42	42.69
Cellulitis and abscess of foot, toe	DGKQ	Wald ratio	1	0.46	0.38	0.223	1.59	0.75	3.35
Rash and other nonspecific skin eruption	DGKQ	Wald ratio	1	0.73	0.60	0.226	2.07	0.64	6.72
Other disorders of eyelids	DGKQ	Wald ratio	1	0.45	0.37	0.227	1.57	0.76	3.26
Synovitis and tenosynovitis	DGKQ	Wald ratio	1	0.64	0.53	0.234	1.89	0.66	5.38
Occlusion and stenosis of precerebral arteries	DGKQ	Wald ratio	1	-0.96	0.81	0.236	0.38	0.08	1.88
Nerve root and plexus disorders	DGKQ	Wald ratio	1	0.98	0.83	0.236	2.67	0.53	13.55

Fracture of foot	DGKQ	Wald ratio	1	0.78	0.66	0.237	2.17	0.60	7.84
Malignant neoplasm, other	DGKQ	Wald ratio	1	-0.27	0.22	0.237	0.77	0.49	1.19
Type 2 diabetes with ophthalmic manifestations	DGKQ	Wald ratio	1	0.92	0.78	0.238	2.51	0.54	11.54
Duodenitis	DGKQ	Wald ratio	1	0.39	0.33	0.238	1.47	0.78	2.79
Cardiac pacemaker in situ	DGKQ	Wald ratio	1	0.69	0.59	0.244	1.99	0.63	6.30
Other retinal disorders	DGKQ	Wald ratio	1	0.52	0.45	0.245	1.69	0.70	4.08
Calculus of ureter	DGKQ	Wald ratio	1	0.67	0.57	0.246	1.95	0.63	6.01
Urinary incontinence	DGKQ	Wald ratio	1	0.35	0.31	0.247	1.42	0.78	2.60
Urinary calculus	DGKQ	Wald ratio	1	-0.40	0.35	0.251	0.67	0.34	1.33
Fracture of ankle and foot	DGKQ	Wald ratio	1	0.67	0.58	0.252	1.95	0.62	6.10
Aortic aneurysm	DGKQ	Wald ratio	1	0.85	0.75	0.258	2.35	0.54	10.32
Secondary malignancy of lymph nodes	DGKQ	Wald ratio	1	0.44	0.39	0.258	1.55	0.73	3.29
Other complications of pregnancy NEC	DGKQ	Wald ratio	1	-0.69	0.61	0.259	0.50	0.15	1.66
Sebaceous cyst	DGKQ	Wald ratio	1	-0.34	0.30	0.260	0.71	0.39	1.29
Endometriosis	DGKQ	Wald ratio	1	-0.50	0.45	0.262	0.60	0.25	1.46
Ulcer of esophagus	DGKQ	Wald ratio	1	-0.44	0.39	0.262	0.64	0.30	1.39
Cellulitis and abscess of face/neck	DGKQ	Wald ratio	1	1.36	1.22	0.268	3.88	0.35	42.72
Other abnormal blood chemistry	DGKQ	Wald ratio	1	0.39	0.36	0.268	1.48	0.74	2.98
Bipolar	DGKQ	Wald ratio	1	-0.95	0.86	0.269	0.39	0.07	2.08
Suppurative and unspecified otitis media	DGKQ	Wald ratio	1	-1.05	0.96	0.273	0.35	0.05	2.28
Raynaud's syndrome	DGKQ	Wald ratio	1	0.90	0.83	0.274	2.47	0.49	12.49
Kyphoscoliosis and scoliosis	DGKQ	Wald ratio	1	-0.94	0.86	0.275	0.39	0.07	2.11
Anorexia	DGKQ	Wald ratio	1	-1.05	0.96	0.278	0.35	0.05	2.32
Pericarditis	DGKQ	Wald ratio	1	-0.85	0.78	0.280	0.43	0.09	1.99
Osteoarthrosis, generalized	DGKQ	Wald ratio	1	1.24	1.14	0.280	3.44	0.37	32.37
Ingrowing nail	DGKQ	Wald ratio	1	-0.97	0.90	0.280	0.38	0.06	2.21

Diseases of the oral soft tissues, excluding lesions specific for gingiva and tongue	DGKQ	Wald ratio	1	0.48	0.45	0.284	1.62	0.67	3.90
Gout and other crystal arthropathies	DGKQ	Wald ratio	1	0.49	0.46	0.286	1.64	0.66	4.04
Cellulitis and abscess of leg, except foot	DGKQ	Wald ratio	1	0.40	0.38	0.286	1.50	0.71	3.15
Open wounds of head; neck; and trunk	DGKQ	Wald ratio	1	-0.48	0.45	0.286	0.62	0.25	1.50
Cardiac pacemaker/device in situ	DGKQ	Wald ratio	1	0.60	0.57	0.287	1.83	0.60	5.53
Abdominal hernia	DGKQ	Wald ratio	1	-0.15	0.14	0.289	0.86	0.66	1.13
Other unspecified back disorders	DGKQ	Wald ratio	1	-0.74	0.70	0.291	0.48	0.12	1.88
Endocarditis	DGKQ	Wald ratio	1	1.12	1.06	0.292	3.07	0.38	24.70
Adverse effects of sedatives or other central nervous system depressants and anesthetics	DGKQ	Wald ratio	1	1.21	1.15	0.293	3.36	0.35	32.13
Fever of unknown origin	DGKQ	Wald ratio	1	0.47	0.45	0.293	1.60	0.67	3.82
Other cerebral degenerations	DGKQ	Wald ratio	1	-0.88	0.84	0.294	0.41	0.08	2.15
Ulcerative colitis	DGKQ	Wald ratio	1	0.52	0.50	0.299	1.68	0.63	4.50
Other signs and symptoms in breast	DGKQ	Wald ratio	1	1.09	1.06	0.301	2.98	0.38	23.63
Other nonmalignant breast conditions	DGKQ	Wald ratio	1	0.65	0.63	0.302	1.92	0.56	6.66
Liver abscess and sequelae of chronic liver disease	DGKQ	Wald ratio	1	-0.93	0.91	0.304	0.39	0.07	2.33
Irritable Bowel Syndrome	DGKQ	Wald ratio	1	0.39	0.38	0.310	1.48	0.70	3.12
Sarcoidosis	DGKQ	Wald ratio	1	-1.21	1.20	0.311	0.30	0.03	3.10
Menopausal and postmenopausal disorders	DGKQ	Wald ratio	1	0.28	0.28	0.313	1.32	0.77	2.29
Manlignant and unknown neoplasms of brain and nervous system	DGKQ	Wald ratio	1	-1.10	1.09	0.314	0.33	0.04	2.83
Chronic periodontitis	DGKQ	Wald ratio	1	-1.18	1.18	0.317	0.31	0.03	3.10
Stricture of artery	DGKQ	Wald ratio	1	0.95	0.95	0.318	2.58	0.40	16.52
Flatulence	DGKQ	Wald ratio	1	0.68	0.68	0.318	1.98	0.52	7.54
Pain in limb	DGKQ	Wald ratio	1	0.35	0.36	0.322	1.42	0.71	2.87
Acute pancreatitis	DGKQ	Wald ratio	1	-0.62	0.63	0.323	0.54	0.16	1.84

Stricture/obstruction of ureter	DGKQ	Wald ratio	1	-0.90	0.92		0.327	0.40	0.07	2.47
Degeneration of intervertebral disc	DGKQ	Wald ratio	1	-0.51	0.53		0.331	0.60	0.21	1.69
Abnormal involuntary movements	DGKQ	Wald ratio	1	0.91	0.94		0.331	2.49	0.40	15.60
Essential hypertension	DGKQ	Wald ratio	1	0.12	0.13		0.331	1.13	0.88	1.45
Ulcerative colitis (chronic)	DGKQ	Wald ratio	1	1.17	1.21		0.335	3.21	0.30	34.56
Hypertensive heart and/or renal disease	DGKQ	Wald ratio	1	-0.65	0.68		0.336	0.52	0.14	1.96
Chronic pharyngitis and nasopharyngitis	DGKQ	Wald ratio	1	0.87	0.90		0.337	2.38	0.41	13.89
Osteomyelitis	DGKQ	Wald ratio	1	-1.12	1.17		0.338	0.33	0.03	3.23
Chronic cystitis	DGKQ	Wald ratio	1	-0.89	0.93		0.341	0.41	0.07	2.56
Osteoarthritis; localized	DGKQ	Wald ratio	1	0.21	0.22		0.342	1.23	0.80	1.90
Noninflammatory disorders of vagina	DGKQ	Wald ratio	1	-0.63	0.67		0.342	0.53	0.14	1.96
Respiratory abnormalities	DGKQ	Wald ratio	1	1.07	1.13		0.344	2.92	0.32	26.89
Congenital anomalies of urinary system	DGKQ	Wald ratio	1	0.90	0.96		0.345	2.47	0.38	16.09
Myeloproliferative disease	DGKQ	Wald ratio	1	-0.84	0.89		0.346	0.43	0.08	2.47
Atherosclerosis	DGKQ	Wald ratio	1	0.73	0.77		0.347	2.07	0.46	9.41
Osteoarthrosis NOS	DGKQ	Wald ratio	1	0.24	0.26		0.347	1.28	0.77	2.12
Osteomyelitis, periostitis, and other infections involving bone	DGKQ	Wald ratio	1	-1.06	1.13		0.348	0.35	0.04	3.16
Stricture and stenosis of esophagus	DGKQ	Wald ratio	1	-0.60	0.65		0.349	0.55	0.15	1.94
Disturbance of skin sensation	DGKQ	Wald ratio	1	-0.48	0.52		0.350	0.62	0.22	1.70
Breast cancer	DGKQ	Wald ratio	1	0.24	0.26		0.350	1.27	0.77	2.12
Chronic tonsillitis and adenoiditis	DGKQ	Wald ratio	1	-0.78	0.83		0.352	0.46	0.09	2.36
Hypertension	DGKQ	Wald ratio	1	0.12	0.13		0.356	1.12	0.88	1.44
Regional enteritis	DGKQ	Wald ratio	1	0.62	0.67		0.359	1.86	0.50	6.94
Cystic mastopathy	DGKQ	Wald ratio	1	0.84	0.92		0.360	2.32	0.38	14.13
Neoplasm of unspecified nature of digestive system	DGKQ	Wald ratio	1	-0.78	0.86		0.364	0.46	0.09	2.47
Other upper respiratory disease	DGKQ	Wald ratio	1	-0.40	0.44		0.365	0.67	0.29	1.58

Poisoning by anticonvulsants and anti-Parkinsonism drugs	DGKQ	Wald ratio	1	1.12	1.24	0.365	3.06	0.27	34.53
Retinal detachments and defects	DGKQ	Wald ratio	1	0.45	0.50	0.366	1.56	0.59	4.13
Anxiety disorders	DGKQ	Wald ratio	1	0.31	0.34	0.369	1.36	0.70	2.66
Cardiac shunt/ heart septal defect	DGKQ	Wald ratio	1	-1.03	1.15	0.370	0.36	0.04	3.41
Alcoholic liver damage	DGKQ	Wald ratio	1	0.88	0.99	0.372	2.42	0.35	16.73
Agorophobia, social phobia, and panic disorder	DGKQ	Wald ratio	1	0.94	1.05	0.372	2.56	0.33	20.06
Hypertensive chronic kidney disease	DGKQ	Wald ratio	1	-0.64	0.71	0.372	0.53	0.13	2.14
Cholelithiasis with acute cholecystitis	DGKQ	Wald ratio	1	0.64	0.72	0.372	1.90	0.46	7.85
Cancer, suspected or other	DGKQ	Wald ratio	1	-0.20	0.22	0.373	0.82	0.53	1.26
Streptococcus infection	DGKQ	Wald ratio	1	0.61	0.69	0.373	1.85	0.48	7.12
Diverticulosis and diverticulitis	DGKQ	Wald ratio	1	-0.16	0.18	0.374	0.85	0.59	1.22
Viral hepatitis	DGKQ	Wald ratio	1	0.71	0.80	0.375	2.04	0.42	9.87
Diverticulosis	DGKQ	Wald ratio	1	-0.16	0.18	0.377	0.85	0.59	1.22
Effects radiation NOS	DGKQ	Wald ratio	1	-0.44	0.50	0.380	0.64	0.24	1.72
Primary open angle glaucoma	DGKQ	Wald ratio	1	-0.76	0.87	0.382	0.47	0.08	2.57
Pneumonitis due to inhalation of food or vomitus	DGKQ	Wald ratio	1	-1.01	1.16	0.382	0.36	0.04	3.51
Abnormal findings on exam of gastrointestinal tract/ abdominal area	DGKQ	Wald ratio	1	-0.60	0.69	0.385	0.55	0.14	2.12
Acidosis	DGKQ	Wald ratio	1	0.75	0.86	0.385	2.11	0.39	11.40
Benign neoplasm of lip, oral cavity, and pharynx	DGKQ	Wald ratio	1	0.77	0.89	0.385	2.17	0.38	12.40
Esophageal bleeding (varices/hemorrhage)	DGKQ	Wald ratio	1	0.59	0.68	0.387	1.80	0.47	6.88
Hallux valgus (Bunion)	DGKQ	Wald ratio	1	0.30	0.35	0.388	1.35	0.68	2.70
Other open wound of head and face	DGKQ	Wald ratio	1	-0.45	0.52	0.389	0.64	0.23	1.77
Postmenopausal atrophic vaginitis	DGKQ	Wald ratio	1	0.72	0.84	0.390	2.06	0.40	10.73
Breast cancer [female]	DGKQ	Wald ratio	1	0.22	0.26	0.391	1.25	0.75	2.09
Open-angle glaucoma	DGKQ	Wald ratio	1	-0.75	0.87	0.391	0.47	0.09	2.61
Paroxysmal tachycardia, unspecified	DGKQ	Wald ratio	1	0.43	0.50	0.391	1.53	0.58	4.05

Other disorders of the kidney and ureters	DGKQ	Wald ratio	1	-0.41	0.49	0.394	0.66	0.26	1.71
Personal history of diseases of digestive system	DGKQ	Wald ratio	1	0.20	0.23	0.395	1.22	0.77	1.93
Cough	DGKQ	Wald ratio	1	0.44	0.52	0.396	1.56	0.56	4.35
Postmenopausal bleeding	DGKQ	Wald ratio	1	0.25	0.30	0.397	1.29	0.72	2.33
Complication of internal orthopedic device	DGKQ	Wald ratio	1	0.42	0.50	0.397	1.53	0.57	4.06
Pathologic fracture	DGKQ	Wald ratio	1	-1.04	1.24	0.400	0.35	0.03	3.98
Acute appendicitis	DGKQ	Wald ratio	1	0.46	0.55	0.400	1.59	0.54	4.69
Allergic rhinitis	DGKQ	Wald ratio	1	0.72	0.86	0.402	2.05	0.38	10.99
Abnormal function study of cardiovascular system	DGKQ	Wald ratio	1	1.02	1.22	0.403	2.78	0.25	30.64
Disorders of adrenal glands	DGKQ	Wald ratio	1	-0.92	1.10	0.405	0.40	0.05	3.46
Cervical cancer and dysplasia	DGKQ	Wald ratio	1	-0.39	0.47	0.405	0.68	0.27	1.69
Gastritis and duodenitis	DGKQ	Wald ratio	1	0.15	0.18	0.407	1.16	0.82	1.63
Hemorrhage from gastrointestinal ulcer	DGKQ	Wald ratio	1	0.93	1.13	0.407	2.55	0.28	23.23
Diseases and other conditions of the tongue	DGKQ	Wald ratio	1	0.66	0.80	0.410	1.93	0.40	9.16
Nevus, non-neoplastic	DGKQ	Wald ratio	1	0.94	1.14	0.411	2.56	0.27	24.15
Perforation of tympanic membrane	DGKQ	Wald ratio	1	-0.71	0.87	0.412	0.49	0.09	2.68
stress incontinence, female	DGKQ	Wald ratio	1	0.31	0.37	0.413	1.36	0.65	2.82
Diseases of nail, NOS	DGKQ	Wald ratio	1	-0.64	0.78	0.413	0.53	0.11	2.44
Retinal vascular changes and abnomalities	DGKQ	Wald ratio	1	0.78	0.96	0.413	2.19	0.33	14.35
Benign neoplasm of ovary	DGKQ	Wald ratio	1	0.60	0.73	0.414	1.82	0.43	7.60
Hemorrhage or hematoma complicating a procedure	DGKQ	Wald ratio	1	0.32	0.39	0.414	1.37	0.64	2.92
Subjective visual disturbances	DGKQ	Wald ratio	1	-0.92	1.12	0.415	0.40	0.04	3.63
Dysphagia	DGKQ	Wald ratio	1	0.29	0.35	0.416	1.33	0.67	2.66
Other aneurysm	DGKQ	Wald ratio	1	0.54	0.66	0.416	1.71	0.47	6.23
Respiratory insufficiency	DGKQ	Wald ratio	1	0.54	0.67	0.417	1.72	0.46	6.40
Viral Enteritis	DGKQ	Wald ratio	1	0.77	0.95	0.417	2.17	0.33	14.01

Sicca syndrome	DGKQ	Wald ratio	1	-1.00	1.24	0.418	0.37	0.03	4.14
Blood in stool	DGKQ	Wald ratio	1	-0.44	0.55	0.418	0.64	0.22	1.88
Jaundice (not of newborn)	DGKQ	Wald ratio	1	0.75	0.93	0.421	2.12	0.34	13.26
Chronic liver disease and cirrhosis	DGKQ	Wald ratio	1	-0.42	0.52	0.421	0.66	0.24	1.83
Corneal opacity and other disorders of cornea	DGKQ	Wald ratio	1	0.83	1.03	0.422	2.29	0.30	17.38
Chemotherapy	DGKQ	Wald ratio	1	0.15	0.19	0.423	1.17	0.80	1.70
Noninfectious gastroenteritis	DGKQ	Wald ratio	1	-0.18	0.23	0.432	0.83	0.53	1.31
Cholesteatoma	DGKQ	Wald ratio	1	-0.89	1.13	0.432	0.41	0.04	3.78
Retinal detachment with retinal defect	DGKQ	Wald ratio	1	0.59	0.75	0.433	1.80	0.41	7.88
Benign neoplasm of colon	DGKQ	Wald ratio	1	-0.16	0.21	0.435	0.85	0.57	1.28
Late pregnancy and failed induction	DGKQ	Wald ratio	1	-0.62	0.80	0.437	0.54	0.11	2.59
Tachycardia NOS	DGKQ	Wald ratio	1	-0.46	0.60	0.438	0.63	0.19	2.03
Umbilical cord complications during labor and delivery	DGKQ	Wald ratio	1	-0.94	1.22	0.439	0.39	0.04	4.25
Simple and unspecified goiter	DGKQ	Wald ratio	1	-0.88	1.14	0.439	0.41	0.04	3.86
Superficial injury without mention of infection	DGKQ	Wald ratio	1	0.33	0.43	0.441	1.39	0.60	3.21
Disorders of mineral metabolism	DGKQ	Wald ratio	1	-0.46	0.61	0.449	0.63	0.19	2.08
Disorders of tooth development	DGKQ	Wald ratio	1	0.43	0.57	0.449	1.53	0.51	4.66
Chronic glomerulonephritis, NOS	DGKQ	Wald ratio	1	0.73	0.96	0.451	2.07	0.31	13.66
Multiple sclerosis	DGKQ	Wald ratio	1	-0.57	0.76	0.451	0.56	0.13	2.51
Cervicalgia	DGKQ	Wald ratio	1	0.50	0.66	0.453	1.64	0.45	6.02
Abnormal movement	DGKQ	Wald ratio	1	0.40	0.54	0.454	1.49	0.52	4.28
Varicose veins	DGKQ	Wald ratio	1	0.20	0.26	0.456	1.22	0.73	2.04
Diseases of the salivary glands	DGKQ	Wald ratio	1	0.78	1.05	0.457	2.18	0.28	17.00
Degeneration of macula and posterior pole of retina	DGKQ	Wald ratio	1	0.45	0.60	0.458	1.56	0.48	5.06
Angina pectoris	DGKQ	Wald ratio	1	0.17	0.24	0.463	1.19	0.75	1.88
Problems associated with amniotic cavity and membranes	DGKQ	Wald ratio	1	-0.56	0.76	0.465	0.57	0.13	2.56

Other anemias	DGKQ	Wald ratio	1	-0.18	0.25	0.468	0.83	0.50	1.37
Bacterial infection NOS	DGKQ	Wald ratio	1	0.18	0.26	0.470	1.20	0.73	1.99
Complications of cardiac/vascular device, implant, and graft	DGKQ	Wald ratio	1	0.48	0.66	0.470	1.61	0.44	5.89
Hearing loss	DGKQ	Wald ratio	1	-0.31	0.43	0.472	0.73	0.31	1.71
Visual disturbances	DGKQ	Wald ratio	1	0.35	0.49	0.474	1.42	0.54	3.71
Acute tonsillitis	DGKQ	Wald ratio	1	-0.81	1.13	0.475	0.45	0.05	4.07
Fracture of neck of femur	DGKQ	Wald ratio	1	0.47	0.66	0.476	1.60	0.44	5.81
Macular degeneration (senile) of retina NOS	DGKQ	Wald ratio	1	0.43	0.60	0.476	1.53	0.47	4.97
Gram negative septicemia	DGKQ	Wald ratio	1	0.69	0.97	0.477	1.99	0.30	13.36
Alcoholism	DGKQ	Wald ratio	1	0.21	0.30	0.478	1.24	0.69	2.24
Swelling of limb	DGKQ	Wald ratio	1	-0.29	0.41	0.478	0.75	0.33	1.68
Ventral hernia	DGKQ	Wald ratio	1	0.34	0.48	0.479	1.40	0.55	3.60
Viral infection	DGKQ	Wald ratio	1	0.37	0.53	0.480	1.45	0.52	4.09
Cholelithiasis	DGKQ	Wald ratio	1	-0.18	0.25	0.481	0.84	0.52	1.37
Atrioventricular block, complete	DGKQ	Wald ratio	1	0.81	1.16	0.481	2.26	0.23	21.75
Acquired toe deformities	DGKQ	Wald ratio	1	-0.28	0.40	0.483	0.76	0.35	1.65
Anal and rectal polyp	DGKQ	Wald ratio	1	-0.23	0.33	0.488	0.80	0.42	1.52
Musculoskeletal symptoms referable to limbs	DGKQ	Wald ratio	1	-0.26	0.38	0.491	0.77	0.36	1.62
Chronic bronchitis	DGKQ	Wald ratio	1	0.36	0.52	0.493	1.43	0.51	3.99
Ulceration of intestine	DGKQ	Wald ratio	1	0.74	1.08	0.493	2.09	0.25	17.29
Candidiasis	DGKQ	Wald ratio	1	0.42	0.61	0.494	1.51	0.46	4.98
Other non-epithelial cancer of skin	DGKQ	Wald ratio	1	0.19	0.27	0.496	1.21	0.70	2.07
Rheumatoid arthritis	DGKQ	Wald ratio	1	0.29	0.43	0.498	1.34	0.58	3.08
Anemia of chronic disease	DGKQ	Wald ratio	1	-0.71	1.05	0.499	0.49	0.06	3.87
Other chronic nonalcoholic liver disease	DGKQ	Wald ratio	1	-0.46	0.69	0.502	0.63	0.16	2.42
Tobacco use disorder	DGKQ	Wald ratio	1	0.14	0.21	0.503	1.15	0.76	1.74

Schizophrenia	DGKQ	Wald ratio	1	0.78	1.17	0.504	2.18	0.22	21.58
Nephritis; nephrosis; renal sclerosis	DGKQ	Wald ratio	1	0.48	0.72	0.504	1.62	0.39	6.63
Malignant neoplasm of rectum, rectosigmoid junction, and anus	DGKQ	Wald ratio	1	-0.41	0.61	0.504	0.66	0.20	2.21
Dental caries	DGKQ	Wald ratio	1	-0.34	0.51	0.504	0.71	0.26	1.93
Delirium dementia and amnestic and other cognitive disorders	DGKQ	Wald ratio	1	0.42	0.63	0.505	1.52	0.44	5.25
Varicose veins of lower extremity	DGKQ	Wald ratio	1	0.18	0.27	0.508	1.20	0.70	2.03
Abdominal aortic aneurysm	DGKQ	Wald ratio	1	0.62	0.94	0.509	1.86	0.30	11.62
Excessive or frequent menstruation	DGKQ	Wald ratio	1	-0.20	0.30	0.509	0.82	0.46	1.47
Cerebral ischemia	DGKQ	Wald ratio	1	-0.34	0.52	0.510	0.71	0.26	1.97
Other symptoms of respiratory system	DGKQ	Wald ratio	1	0.20	0.30	0.511	1.22	0.68	2.18
Other disorders of pancreatic internal secretion	DGKQ	Wald ratio	1	0.60	0.91	0.511	1.82	0.30	10.87
Late effects of cerebrovascular disease	DGKQ	Wald ratio	1	-0.51	0.79	0.514	0.60	0.13	2.80
Poisoning by psychotropic agents	DGKQ	Wald ratio	1	-0.42	0.64	0.515	0.66	0.19	2.30
Rheumatoid arthritis and other inflammatory polyarthropathies	DGKQ	Wald ratio	1	0.26	0.41	0.516	1.30	0.59	2.89
Cancer within the respiratory system	DGKQ	Wald ratio	1	0.35	0.54	0.517	1.42	0.49	4.11
Rheumatism, unspecified and fibrositis	DGKQ	Wald ratio	1	-0.57	0.88	0.517	0.56	0.10	3.18
Ankylosing spondylitis	DGKQ	Wald ratio	1	-0.73	1.12	0.518	0.48	0.05	4.36
Diseases of hard tissues of teeth	DGKQ	Wald ratio	1	-0.33	0.51	0.518	0.72	0.27	1.94
Myalgia and myositis unspecified	DGKQ	Wald ratio	1	-0.71	1.10	0.519	0.49	0.06	4.27
Diseases of spleen	DGKQ	Wald ratio	1	0.80	1.24	0.520	2.22	0.20	24.98
Fracture of humerus	DGKQ	Wald ratio	1	0.44	0.68	0.521	1.55	0.41	5.85
Non-Hodgkins lymphoma	DGKQ	Wald ratio	1	0.42	0.66	0.523	1.53	0.42	5.59
Hydronephrosis	DGKQ	Wald ratio	1	0.40	0.63	0.523	1.50	0.43	5.17
Other diseases of respiratory system, not elsewhere classified	DGKQ	Wald ratio	1	-0.18	0.29	0.523	0.83	0.47	1.47
Osteopenia or other disorder of bone and cartilage	DGKQ	Wald ratio	1	-0.62	0.98	0.524	0.54	0.08	3.65
Occlusion of cerebral arteries	DGKQ	Wald ratio	1	0.28	0.44	0.526	1.32	0.56	3.12

Pneumonia	DGKQ	Wald ratio	1	0.18	0.28	0.527	1.20	0.69	2.09
Noninfectious disorders of lymphatic channels	DGKQ	Wald ratio	1	-0.67	1.05	0.528	0.51	0.07	4.06
Osteoarthrosis, localized, primary	DGKQ	Wald ratio	1	0.19	0.30	0.529	1.21	0.67	2.18
Symptoms involving head and neck	DGKQ	Wald ratio	1	0.32	0.51	0.529	1.38	0.51	3.76
Other diseases of lung	DGKQ	Wald ratio	1	0.63	1.00	0.530	1.88	0.26	13.33
Disorders of sweat glands	DGKQ	Wald ratio	1	0.61	0.98	0.530	1.85	0.27	12.52
Disorders of iron metabolism	DGKQ	Wald ratio	1	-0.67	1.08	0.533	0.51	0.06	4.22
Acute upper respiratory infections of multiple or unspecified sites	DGKQ	Wald ratio	1	0.36	0.58	0.534	1.43	0.46	4.45
Functional digestive disorders	DGKQ	Wald ratio	1	0.12	0.20	0.534	1.13	0.77	1.66
Transient cerebral ischemia	DGKQ	Wald ratio	1	-0.38	0.60	0.535	0.69	0.21	2.25
Arterial embolism and thrombosis of lower extremity artery	DGKQ	Wald ratio	1	0.73	1.18	0.536	2.08	0.20	21.13
Fracture of radius and ulna	DGKQ	Wald ratio	1	-0.24	0.39	0.537	0.78	0.36	1.69
Large cell lymphoma	DGKQ	Wald ratio	1	0.72	1.17	0.538	2.05	0.21	20.18
Hypertension complicating pregnancy, childbirth, and the puerperium	DGKQ	Wald ratio	1	-0.52	0.85	0.539	0.59	0.11	3.16
Disorders of other cranial nerves	DGKQ	Wald ratio	1	0.46	0.75	0.539	1.59	0.36	6.89
Malignant neoplasm of female breast	DGKQ	Wald ratio	1	0.17	0.27	0.540	1.18	0.70	2.00
Disorders of the pituitary gland and its hypothalamic control	DGKQ	Wald ratio	1	-0.65	1.06	0.541	0.52	0.07	4.18
Blindness and low vision	DGKQ	Wald ratio	1	0.63	1.04	0.541	1.89	0.25	14.43
Urethral stricture (not specified as infectious)	DGKQ	Wald ratio	1	-0.30	0.49	0.541	0.74	0.29	1.93
Intestinal infection	DGKQ	Wald ratio	1	0.18	0.30	0.544	1.20	0.67	2.14
Obstructive chronic bronchitis	DGKQ	Wald ratio	1	0.33	0.54	0.545	1.39	0.48	4.05
Other disorders of arteries and arterioles	DGKQ	Wald ratio	1	0.46	0.77	0.545	1.59	0.35	7.17
Secondary malignant neoplasm of digestive systems	DGKQ	Wald ratio	1	0.43	0.72	0.546	1.54	0.38	6.27
Cerebral artery occlusion, with cerebral infarction	DGKQ	Wald ratio	1	0.44	0.72	0.547	1.55	0.37	6.41
Sleep disorders	DGKQ	Wald ratio	1	-0.24	0.39	0.547	0.79	0.37	1.70
Inflammation of eyelids	DGKQ	Wald ratio	1	-0.34	0.57	0.549	0.71	0.23	2.17

Type 2 diabetes with neurological manifestations	DGKQ	Wald ratio	1	0.70	1.17	0.549	2.01	0.20	19.80
Inflammation of the eye	DGKQ	Wald ratio	1	-0.30	0.50	0.550	0.74	0.28	1.96
Hemorrhage of rectum and anus	DGKQ	Wald ratio	1	0.15	0.24	0.550	1.16	0.72	1.87
Psychogenic and somatoform disorders	DGKQ	Wald ratio	1	0.72	1.21	0.550	2.06	0.19	22.20
Other disorders of biliary tract	DGKQ	Wald ratio	1	-0.52	0.87	0.551	0.60	0.11	3.27
Gout	DGKQ	Wald ratio	1	0.30	0.50	0.552	1.35	0.51	3.59
Chronic fatigue syndrome	DGKQ	Wald ratio	1	-0.68	1.15	0.552	0.51	0.05	4.79
Hypoglycemia	DGKQ	Wald ratio	1	0.54	0.91	0.555	1.72	0.29	10.30
Disorders of lacrimal system	DGKQ	Wald ratio	1	-0.35	0.60	0.556	0.70	0.22	2.26
Iron deficiency anemias, unspecified or not due to blood loss	DGKQ	Wald ratio	1	0.19	0.33	0.556	1.21	0.64	2.32
Purpura and other hemorrhagic conditions	DGKQ	Wald ratio	1	0.39	0.66	0.556	1.48	0.40	5.38
Cancer of bronchus; lung	DGKQ	Wald ratio	1	0.36	0.61	0.557	1.43	0.43	4.77
Paralytic ileus	DGKQ	Wald ratio	1	-0.72	1.22	0.558	0.49	0.04	5.38
Benign neoplasm of uterus	DGKQ	Wald ratio	1	0.16	0.28	0.560	1.18	0.68	2.04
Sleep apnea	DGKQ	Wald ratio	1	-0.25	0.42	0.561	0.78	0.34	1.80
Foreign body injury	DGKQ	Wald ratio	1	0.47	0.81	0.562	1.59	0.33	7.73
First degree AV block	DGKQ	Wald ratio	1	-0.52	0.89	0.563	0.60	0.10	3.43
Functional disorders of bladder	DGKQ	Wald ratio	1	0.40	0.69	0.563	1.49	0.39	5.72
Pilonidal cyst	DGKQ	Wald ratio	1	0.67	1.16	0.565	1.95	0.20	18.85
Heart valve replaced	DGKQ	Wald ratio	1	-0.42	0.72	0.565	0.66	0.16	2.73
Ill-defined descriptions and complications of heart disease	DGKQ	Wald ratio	1	0.57	0.99	0.565	1.77	0.25	12.26
Septal Deviations/Turbinate Hypertrophy	DGKQ	Wald ratio	1	0.23	0.40	0.566	1.26	0.57	2.78
Missed abortion/Hydatidiform mole	DGKQ	Wald ratio	1	0.46	0.80	0.566	1.58	0.33	7.62
Uterine leiomyoma	DGKQ	Wald ratio	1	0.16	0.28	0.567	1.18	0.67	2.06
Scar conditions and fibrosis of skin	DGKQ	Wald ratio	1	-0.33	0.58	0.567	0.72	0.23	2.22
Cancer of mouth	DGKQ	Wald ratio	1	-0.63	1.10	0.568	0.53	0.06	4.62

Other specified nonpsychotic and/or transient mental disorders	DGKQ	Wald ratio	1	-0.63	1.11	0.569	0.53	0.06	4.65
Periodontitis (acute or chronic)	DGKQ	Wald ratio	1	0.45	0.80	0.572	1.57	0.33	7.50
Erythematous conditions	DGKQ	Wald ratio	1	0.32	0.57	0.574	1.38	0.45	4.19
Digestive congenital anomalies	DGKQ	Wald ratio	1	0.59	1.05	0.574	1.81	0.23	14.21
Other derangement of joint	DGKQ	Wald ratio	1	-0.35	0.63	0.575	0.70	0.21	2.40
Symptoms affecting skin	DGKQ	Wald ratio	1	0.21	0.38	0.576	1.23	0.59	2.58
Other symptoms involving abdomen and pelvis	DGKQ	Wald ratio	1	0.27	0.49	0.580	1.31	0.50	3.46
Colon cancer	DGKQ	Wald ratio	1	0.28	0.51	0.580	1.33	0.49	3.62
Noninflammatory disorders of ovary, fallopian tube, and broad ligament	DGKQ	Wald ratio	1	-0.57	1.04	0.581	0.56	0.07	4.30
Alcohol-related disorders	DGKQ	Wald ratio	1	0.14	0.25	0.581	1.15	0.70	1.89
Benign neoplasm of skin	DGKQ	Wald ratio	1	0.18	0.32	0.581	1.20	0.63	2.26
Depression	DGKQ	Wald ratio	1	0.15	0.26	0.582	1.16	0.69	1.94
Diseases of the jaws	DGKQ	Wald ratio	1	-0.49	0.90	0.583	0.61	0.10	3.57
Other acquired deformities of limbs	DGKQ	Wald ratio	1	-0.38	0.69	0.584	0.69	0.18	2.65
Bursitis	DGKQ	Wald ratio	1	0.52	0.95	0.585	1.68	0.26	10.92
Melanomas of skin, dx or hx	DGKQ	Wald ratio	1	-0.30	0.54	0.586	0.74	0.26	2.16
Melanomas of skin	DGKQ	Wald ratio	1	-0.30	0.54	0.586	0.74	0.26	2.16
Hypotension NOS	DGKQ	Wald ratio	1	0.26	0.47	0.586	1.29	0.51	3.26
Aseptic necrosis of bone	DGKQ	Wald ratio	1	0.65	1.20	0.586	1.92	0.18	20.18
Inflammatory disease of cervix, vagina, and vulva	DGKQ	Wald ratio	1	0.29	0.53	0.587	1.33	0.47	3.75
Disorders of calcium/phosphorus metabolism	DGKQ	Wald ratio	1	-0.43	0.81	0.590	0.65	0.13	3.14
Cancer of stomach	DGKQ	Wald ratio	1	0.64	1.19	0.590	1.90	0.19	19.40
Pain	DGKQ	Wald ratio	1	-0.54	1.00	0.592	0.58	0.08	4.18
Other disorders of middle ear and mastoid	DGKQ	Wald ratio	1	-0.52	0.97	0.593	0.59	0.09	4.01
Hallux rigidus	DGKQ	Wald ratio	1	0.38	0.71	0.595	1.46	0.36	5.91
Arterial embolism and thrombosis	DGKQ	Wald ratio	1	0.49	0.92	0.596	1.63	0.27	9.96

Atrioventricular [AV] block	DGKQ	Wald ratio	1	-0.32	0.61	0.600	0.73	0.22	2.39
Cardiac arrest and ventricular fibrillation	DGKQ	Wald ratio	1	-0.43	0.83	0.601	0.65	0.13	3.29
Benign neoplasm of other endocrine glands and related structures	DGKQ	Wald ratio	1	0.49	0.94	0.603	1.63	0.26	10.37
Ulceration of the lower GI tract	DGKQ	Wald ratio	1	0.44	0.86	0.605	1.56	0.29	8.40
Cholelithiasis and cholecystitis	DGKQ	Wald ratio	1	-0.12	0.23	0.606	0.89	0.57	1.39
Retention of urine	DGKQ	Wald ratio	1	-0.18	0.35	0.611	0.84	0.42	1.65
Disorders of refraction and accommodation; blindness and low vision	DGKQ	Wald ratio	1	0.28	0.57	0.616	1.33	0.44	4.03
Intracranial hemorrhage (injury)	DGKQ	Wald ratio	1	-0.61	1.24	0.620	0.54	0.05	6.11
Antepartum hemorrhage, abruptio placentae, and placenta previa	DGKQ	Wald ratio	1	0.48	0.96	0.621	1.61	0.24	10.58
Bladder neck obstruction	DGKQ	Wald ratio	1	-0.31	0.63	0.623	0.73	0.21	2.53
Other disorders of gallbladder	DGKQ	Wald ratio	1	-0.37	0.75	0.624	0.69	0.16	2.99
Diplopia and disorders of binocular vision	DGKQ	Wald ratio	1	0.51	1.04	0.624	1.66	0.22	12.64
Labyrinthitis	DGKQ	Wald ratio	1	0.49	1.01	0.625	1.64	0.23	11.90
Hyperhidrosis	DGKQ	Wald ratio	1	0.56	1.15	0.625	1.75	0.18	16.59
Other forms of chronic heart disease	DGKQ	Wald ratio	1	0.32	0.66	0.627	1.38	0.38	5.04
Left bundle branch block	DGKQ	Wald ratio	1	-0.33	0.67	0.627	0.72	0.19	2.68
Type 1 diabetes	DGKQ	Wald ratio	1	0.27	0.55	0.628	1.30	0.45	3.81
Arrhythmia (cardiac) NOS	DGKQ	Wald ratio	1	0.45	0.92	0.628	1.56	0.26	9.51
Phlebitis and thrombophlebitis of lower extremities	DGKQ	Wald ratio	1	-0.23	0.47	0.629	0.80	0.32	2.01
Complication due to other implant and internal device	DGKQ	Wald ratio	1	0.24	0.49	0.630	1.27	0.48	3.33
Crushing or internal injury to organs	DGKQ	Wald ratio	1	0.42	0.88	0.630	1.53	0.27	8.56
Other disorders of urethra and urinary tract	DGKQ	Wald ratio	1	-0.21	0.44	0.631	0.81	0.34	1.92
Effects of other external causes	DGKQ	Wald ratio	1	0.21	0.43	0.632	1.23	0.53	2.88
Cardiomegaly	DGKQ	Wald ratio	1	0.27	0.56	0.633	1.30	0.44	3.87
Fracture of upper limb	DGKQ	Wald ratio	1	0.15	0.31	0.634	1.16	0.63	2.13
Complications of labor and delivery NEC	DGKQ	Wald ratio	1	0.28	0.59	0.634	1.32	0.42	4.19

Psoriasis and related disorders	DGKQ	Wald ratio	1	-0.28	0.59	0.635	0.76	0.24	2.39
Psoriasis vulgaris	DGKQ	Wald ratio	1	-0.32	0.68	0.636	0.72	0.19	2.75
Other disorders of intestine	DGKQ	Wald ratio	1	0.21	0.43	0.637	1.23	0.52	2.88
Sciatica	DGKQ	Wald ratio	1	0.27	0.58	0.639	1.31	0.42	4.05
Inflammatory diseases of female pelvic organs	DGKQ	Wald ratio	1	0.17	0.36	0.640	1.18	0.58	2.40
Vaginal enterocele, congenital or acquired	DGKQ	Wald ratio	1	0.50	1.07	0.643	1.64	0.20	13.47
Hammer toe (acquired)	DGKQ	Wald ratio	1	-0.30	0.64	0.643	0.74	0.21	2.60
Intestinal obstruction without mention of hernia	DGKQ	Wald ratio	1	-0.20	0.44	0.645	0.82	0.34	1.94
Other disorders of circulatory system	DGKQ	Wald ratio	1	0.10	0.23	0.645	1.11	0.71	1.73
Abnormal glucose	DGKQ	Wald ratio	1	0.49	1.07	0.646	1.63	0.20	13.20
Disturbances in tooth eruption	DGKQ	Wald ratio	1	0.26	0.58	0.647	1.30	0.42	4.03
Dislocation	DGKQ	Wald ratio	1	0.28	0.61	0.649	1.32	0.40	4.37
Cervicitis and endocervicitis	DGKQ	Wald ratio	1	-0.35	0.77	0.651	0.71	0.16	3.21
Atopic/contact dermatitis due to other or unspecified	DGKQ	Wald ratio	1	-0.27	0.61	0.656	0.76	0.23	2.52
Dizziness and giddiness (Light-headedness and vertigo)	DGKQ	Wald ratio	1	-0.18	0.42	0.656	0.83	0.37	1.88
Hyperpotassemia	DGKQ	Wald ratio	1	-0.40	0.90	0.659	0.67	0.12	3.91
Breast conditions, congenital or relating to hormones	DGKQ	Wald ratio	1	-0.39	0.91	0.665	0.67	0.11	4.03
Disorders of esophageal motility	DGKQ	Wald ratio	1	0.48	1.11	0.667	1.61	0.18	14.08
Anxiety disorder	DGKQ	Wald ratio	1	0.15	0.36	0.671	1.16	0.58	2.34
Pleurisy; pleural effusion	DGKQ	Wald ratio	1	0.15	0.35	0.673	1.16	0.58	2.30
Circulatory disease NEC	DGKQ	Wald ratio	1	0.10	0.23	0.673	1.10	0.71	1.72
Other biliary tract disease	DGKQ	Wald ratio	1	-0.19	0.45	0.673	0.83	0.34	2.01
Other diseases of respiratory system, NEC	DGKQ	Wald ratio	1	-0.13	0.30	0.673	0.88	0.49	1.58
Aortic valve disease	DGKQ	Wald ratio	1	0.33	0.79	0.678	1.39	0.30	6.51
Phlebitis and thrombophlebitis	DGKQ	Wald ratio	1	-0.19	0.45	0.679	0.83	0.34	2.01
Congenital anomalies of great vessels	DGKQ	Wald ratio	1	-0.27	0.66	0.684	0.76	0.21	2.80

Cancer of esophagus	DGKQ	Wald ratio	1	-0.42	1.04	0.684	0.65	0.08	5.05
Other disorders of metabolism	DGKQ	Wald ratio	1	-0.30	0.74	0.684	0.74	0.17	3.17
Disorders of lipoid metabolism	DGKQ	Wald ratio	1	0.07	0.16	0.685	1.07	0.77	1.48
Gastrointestinal hemorrhage	DGKQ	Wald ratio	1	-0.08	0.20	0.686	0.92	0.63	1.36
Glomerulonephritis	DGKQ	Wald ratio	1	0.35	0.87	0.686	1.42	0.26	7.87
Vascular insufficiency of intestine	DGKQ	Wald ratio	1	0.47	1.17	0.688	1.60	0.16	15.70
Other acute and subacute forms of ischemic heart disease	DGKQ	Wald ratio	1	0.33	0.82	0.689	1.39	0.28	6.94
Infection/inflammation of internal prosthetic device; implant; and graft	DGKQ	Wald ratio	1	0.22	0.56	0.689	1.25	0.42	3.75
Gingival and periodontal diseases	DGKQ	Wald ratio	1	0.27	0.67	0.689	1.31	0.35	4.85
Skin cancer	DGKQ	Wald ratio	1	0.10	0.25	0.692	1.10	0.68	1.80
Fracture of ribs	DGKQ	Wald ratio	1	0.39	1.00	0.696	1.48	0.21	10.60
Vitamin deficiency	DGKQ	Wald ratio	1	0.31	0.81	0.696	1.37	0.28	6.64
Hypovolemia	DGKQ	Wald ratio	1	-0.21	0.53	0.698	0.81	0.29	2.29
Calculus of lower urinary tract	DGKQ	Wald ratio	1	0.39	1.00	0.700	1.47	0.21	10.54
Irregular menstrual cycle	DGKQ	Wald ratio	1	-0.24	0.63	0.701	0.78	0.23	2.70
Pulmonary collapse; interstitial and compensatory emphysema	DGKQ	Wald ratio	1	0.24	0.62	0.702	1.27	0.37	4.31
Malaise and fatigue	DGKQ	Wald ratio	1	-0.18	0.48	0.703	0.83	0.32	2.14
Cancer of other female genital organs	DGKQ	Wald ratio	1	0.22	0.57	0.704	1.24	0.41	3.78
Cystitis	DGKQ	Wald ratio	1	-0.20	0.52	0.704	0.82	0.30	2.27
Abnormal findings on mammogram or breast exam	DGKQ	Wald ratio	1	0.27	0.71	0.705	1.31	0.33	5.22
Other dyspnea	DGKQ	Wald ratio	1	0.35	0.93	0.708	1.42	0.23	8.72
Intestinal malabsorption (non-celiac)	DGKQ	Wald ratio	1	0.23	0.62	0.708	1.26	0.38	4.21
Constipation	DGKQ	Wald ratio	1	-0.10	0.28	0.710	0.90	0.52	1.56
Peritoneal or intestinal adhesions	DGKQ	Wald ratio	1	-0.36	0.97	0.710	0.70	0.10	4.65
Protein-calorie malnutrition	DGKQ	Wald ratio	1	-0.32	0.86	0.711	0.73	0.14	3.91
Other intestinal obstruction	DGKQ	Wald ratio	1	-0.18	0.48	0.712	0.84	0.32	2.16

Nontoxic multinodular goiter	DGKQ	Wald ratio	1	0.40	1.08	0.713	1.49	0.18	12.22
Fracture of unspecified bones	DGKQ	Wald ratio	1	-0.28	0.77	0.713	0.75	0.17	3.43
Renal colic	DGKQ	Wald ratio	1	-0.23	0.62	0.714	0.80	0.24	2.68
Periapical abscess	DGKQ	Wald ratio	1	-0.30	0.81	0.716	0.74	0.15	3.67
Hypothyroidism NOS	DGKQ	Wald ratio	1	0.09	0.25	0.716	1.09	0.67	1.79
Fracture of patella	DGKQ	Wald ratio	1	-0.36	1.01	0.720	0.70	0.10	5.03
Osteoporosis, osteopenia and pathological fracture	DGKQ	Wald ratio	1	-0.12	0.33	0.720	0.89	0.47	1.69
Acid-base balance disorder	DGKQ	Wald ratio	1	0.29	0.82	0.721	1.34	0.27	6.68
Pelvic peritoneal adhesions, female (postoperative) (postinfection)	DGKQ	Wald ratio	1	0.20	0.56	0.721	1.22	0.41	3.66
Empyema and pneumothorax	DGKQ	Wald ratio	1	-0.29	0.81	0.725	0.75	0.15	3.71
Other hypertrophic and atrophic conditions of skin	DGKQ	Wald ratio	1	0.14	0.41	0.726	1.15	0.52	2.56
Spondylosis and allied disorders	DGKQ	Wald ratio	1	0.11	0.32	0.726	1.12	0.60	2.10
Hyperlipidemia	DGKQ	Wald ratio	1	0.06	0.16	0.728	1.06	0.77	1.46
Extrapyramidal disease and abnormal movement disorders	DGKQ	Wald ratio	1	0.32	0.93	0.731	1.38	0.22	8.62
Obesity	DGKQ	Wald ratio	1	0.10	0.28	0.732	1.10	0.64	1.90
Aphakia and other disorders of lens	DGKQ	Wald ratio	1	0.22	0.66	0.734	1.25	0.34	4.56
Polyp of female genital organs	DGKQ	Wald ratio	1	-0.09	0.28	0.737	0.91	0.53	1.57
Cancer of bladder	DGKQ	Wald ratio	1	0.19	0.57	0.742	1.21	0.39	3.71
Portal hypertension	DGKQ	Wald ratio	1	-0.40	1.21	0.743	0.67	0.06	7.23
Overweight, obesity and other hyperalimentation	DGKQ	Wald ratio	1	0.09	0.28	0.743	1.10	0.64	1.89
Nontoxic nodular goiter	DGKQ	Wald ratio	1	0.27	0.83	0.744	1.31	0.26	6.68
Acquired absence of breast	DGKQ	Wald ratio	1	-0.19	0.57	0.744	0.83	0.27	2.56
Symptoms involving skin and other integumentary tissue	DGKQ	Wald ratio	1	0.21	0.66	0.748	1.24	0.34	4.48
E. coli	DGKQ	Wald ratio	1	0.17	0.54	0.749	1.19	0.41	3.40
Degenerative skin conditions and other dermatoses	DGKQ	Wald ratio	1	-0.12	0.38	0.750	0.89	0.42	1.87
Injury, NOS	DGKQ	Wald ratio	1	-0.10	0.31	0.752	0.91	0.49	1.66

Placenta previa and abruptio placenta	DGKQ	Wald ratio	1	-0.25	0.80	0.753	0.78	0.16	3.73
Calculus of bile duct	DGKQ	Wald ratio	1	0.17	0.55	0.753	1.19	0.41	3.48
Meniere's disease	DGKQ	Wald ratio	1	-0.37	1.16	0.753	0.69	0.07	6.79
Diseases of lips	DGKQ	Wald ratio	1	0.33	1.07	0.754	1.40	0.17	11.39
Acute and chronic tonsillitis	DGKQ	Wald ratio	1	-0.19	0.63	0.756	0.82	0.24	2.80
Unspecified polyarthropathy or polyarthritis	DGKQ	Wald ratio	1	0.15	0.48	0.756	1.16	0.46	2.94
Septicemia	DGKQ	Wald ratio	1	0.14	0.44	0.757	1.15	0.48	2.73
Malignant neoplasm of testis	DGKQ	Wald ratio	1	0.16	0.52	0.758	1.17	0.42	3.25
Hypothyroidism	DGKQ	Wald ratio	1	0.07	0.24	0.759	1.08	0.67	1.74
Myocardial infarction	DGKQ	Wald ratio	1	-0.08	0.27	0.759	0.92	0.54	1.57
Ectropion or entropion	DGKQ	Wald ratio	1	-0.26	0.86	0.764	0.77	0.14	4.15
Lump or mass in breast	DGKQ	Wald ratio	1	0.21	0.72	0.765	1.24	0.30	5.07
Chronic ulcer of skin	DGKQ	Wald ratio	1	0.19	0.63	0.767	1.20	0.35	4.11
Benign neoplasm of brain and other parts of nervous system	DGKQ	Wald ratio	1	-0.29	0.97	0.768	0.75	0.11	5.06
Vertiginous syndromes and other disorders of vestibular system	DGKQ	Wald ratio	1	-0.11	0.36	0.770	0.90	0.44	1.82
Other abnormality of urination	DGKQ	Wald ratio	1	0.19	0.64	0.770	1.21	0.34	4.23
Hypertrophy of breast (Gynecomastia)	DGKQ	Wald ratio	1	-0.28	0.96	0.771	0.76	0.12	4.94
Known or suspected fetal abnormality affecting management of mother	DGKQ	Wald ratio	1	0.13	0.46	0.773	1.14	0.47	2.80
Other dyschromia	DGKQ	Wald ratio	1	-0.29	1.00	0.774	0.75	0.11	5.31
Cystitis and urethritis	DGKQ	Wald ratio	1	-0.15	0.51	0.774	0.86	0.32	2.33
Cardiac congenital anomalies	DGKQ	Wald ratio	1	-0.16	0.55	0.775	0.85	0.29	2.51
Other symptoms/disorders or the urinary system	DGKQ	Wald ratio	1	0.05	0.18	0.775	1.05	0.73	1.51
Hemoptysis	DGKQ	Wald ratio	1	-0.17	0.61	0.777	0.84	0.25	2.80
Malignant neoplasm of ovary and other uterine adnexa	DGKQ	Wald ratio	1	-0.17	0.61	0.781	0.84	0.26	2.79
Malignant neoplasm of ovary	DGKQ	Wald ratio	1	-0.17	0.61	0.781	0.84	0.25	2.81
Lymphoid leukemia	DGKQ	Wald ratio	1	0.32	1.16	0.781	1.38	0.14	13.49

Abdominal pain	DGKQ	Wald ratio	1	0.04	0.14	0.781	1.04	0.78	1.38
Carcinoma in situ of skin	DGKQ	Wald ratio	1	0.30	1.08	0.782	1.35	0.16	11.29
Other symptoms	DGKQ	Wald ratio	1	0.22	0.80	0.782	1.25	0.26	5.96
Psoriasis	DGKQ	Wald ratio	1	-0.16	0.59	0.784	0.85	0.27	2.72
Multiple myeloma	DGKQ	Wald ratio	1	0.33	1.19	0.784	1.38	0.13	14.20
Actinic keratosis	DGKQ	Wald ratio	1	-0.15	0.55	0.786	0.86	0.29	2.54
Fracture of vertebral column without mention of spinal cord injury	DGKQ	Wald ratio	1	0.21	0.76	0.786	1.23	0.28	5.45
Chronic airway obstruction	DGKQ	Wald ratio	1	-0.08	0.28	0.786	0.93	0.53	1.61
Traumatic arthropathy	DGKQ	Wald ratio	1	0.32	1.21	0.793	1.37	0.13	14.78
Cerebrovascular disease	DGKQ	Wald ratio	1	-0.08	0.30	0.793	0.92	0.51	1.68
Normal delivery	DGKQ	Wald ratio	1	-0.17	0.66	0.794	0.84	0.23	3.05
Early onset of delivery	DGKQ	Wald ratio	1	-0.29	1.13	0.795	0.74	0.08	6.87
Early or threatened labor; hemorrhage in early pregnancy	DGKQ	Wald ratio	1	0.15	0.57	0.797	1.16	0.38	3.57
Abnormal heart sounds	DGKQ	Wald ratio	1	-0.22	0.86	0.798	0.80	0.15	4.36
Phobia	DGKQ	Wald ratio	1	-0.32	1.25	0.799	0.73	0.06	8.40
Hyperparathyroidism	DGKQ	Wald ratio	1	-0.25	1.00	0.802	0.78	0.11	5.55
Other disorders of soft tissues	DGKQ	Wald ratio	1	-0.09	0.36	0.803	0.91	0.45	1.85
Benign neoplasm of brain, cranial nerves, meninges	DGKQ	Wald ratio	1	-0.25	1.01	0.803	0.78	0.11	5.60
Prolapse of vaginal vault after hysterectomy	DGKQ	Wald ratio	1	-0.30	1.24	0.805	0.74	0.07	8.30
Other disorders of tympanic membrane	DGKQ	Wald ratio	1	-0.19	0.76	0.805	0.83	0.19	3.67
Abnormal sputum	DGKQ	Wald ratio	1	-0.15	0.60	0.809	0.86	0.27	2.80
Epistaxis or throat hemorrhage	DGKQ	Wald ratio	1	-0.14	0.57	0.809	0.87	0.29	2.65
Injuries to the nervous system	DGKQ	Wald ratio	1	0.19	0.77	0.810	1.20	0.27	5.46
Intracerebral hemorrhage	DGKQ	Wald ratio	1	0.25	1.06	0.812	1.29	0.16	10.24
Nonspecific abnormal findings in stool contents	DGKQ	Wald ratio	1	-0.16	0.69	0.813	0.85	0.22	3.26
Peptic ulcer (excl. esophageal)	DGKQ	Wald ratio	1	0.08	0.33	0.815	1.08	0.57	2.06

Osteoporosis NOS	DGKQ	Wald ratio	1	-0.09	0.38	0.816	0.91	0.43	1.93
Appendicitis	DGKQ	Wald ratio	1	0.12	0.50	0.816	1.12	0.42	2.98
Hypercholesterolemia	DGKQ	Wald ratio	1	0.04	0.17	0.817	1.04	0.75	1.45
Spondylosis without myelopathy	DGKQ	Wald ratio	1	-0.09	0.40	0.818	0.91	0.42	1.99
Psoriatic arthropathy	DGKQ	Wald ratio	1	-0.24	1.05	0.819	0.79	0.10	6.14
GERD	DGKQ	Wald ratio	1	0.06	0.24	0.819	1.06	0.66	1.70
Open wounds of extremities	DGKQ	Wald ratio	1	-0.10	0.43	0.821	0.91	0.39	2.12
Vitamin B-complex deficiencies	DGKQ	Wald ratio	1	-0.23	1.02	0.823	0.80	0.11	5.86
Other disorders of peritoneum	DGKQ	Wald ratio	1	-0.11	0.49	0.823	0.90	0.34	2.34
Edema	DGKQ	Wald ratio	1	0.15	0.69	0.824	1.17	0.30	4.51
Pelvic inflammatory disease (PID)	DGKQ	Wald ratio	1	-0.20	0.92	0.825	0.82	0.14	4.91
Other inflammatory spondylopathies	DGKQ	Wald ratio	1	-0.15	0.68	0.825	0.86	0.22	3.28
Hypopotassemia	DGKQ	Wald ratio	1	-0.16	0.74	0.828	0.85	0.20	3.62
Aphasia/speech disturbance	DGKQ	Wald ratio	1	0.16	0.72	0.828	1.17	0.29	4.79
Celiac disease	DGKQ	Wald ratio	1	-0.14	0.65	0.829	0.87	0.24	3.13
Memory loss	DGKQ	Wald ratio	1	0.23	1.06	0.830	1.25	0.16	9.96
Malunion and nonunion of fracture	DGKQ	Wald ratio	1	0.17	0.79	0.832	1.18	0.25	5.53
Spinal stenosis	DGKQ	Wald ratio	1	0.10	0.46	0.833	1.10	0.44	2.73
Acute pharyngitis	DGKQ	Wald ratio	1	0.20	0.95	0.834	1.22	0.19	7.82
Cardiac dysrhythmias	DGKQ	Wald ratio	1	-0.04	0.19	0.837	0.96	0.66	1.39
Infertility, female	DGKQ	Wald ratio	1	0.16	0.77	0.838	1.17	0.26	5.34
Hemangioma and lymphangioma, any site	DGKQ	Wald ratio	1	-0.14	0.70	0.838	0.87	0.22	3.42
Diaphragmatic hernia	DGKQ	Wald ratio	1	-0.04	0.18	0.838	0.96	0.67	1.38
Seborrheic keratosis	DGKQ	Wald ratio	1	-0.10	0.51	0.839	0.90	0.34	2.43
Other disorders of eye	DGKQ	Wald ratio	1	0.09	0.45	0.839	1.10	0.45	2.65
Myopia	DGKQ	Wald ratio	1	0.16	0.79	0.840	1.17	0.25	5.53

Other and unspecified disorders of back	DGKQ	Wald ratio	1	-0.12	0.62	0.841	0.88	0.26	2.95
Shortness of breath	DGKQ	Wald ratio	1	0.07	0.37	0.845	1.07	0.52	2.21
Pancreatic cancer	DGKQ	Wald ratio	1	0.22	1.15	0.848	1.25	0.13	11.90
Other disorders of stomach and duodenum	DGKQ	Wald ratio	1	-0.09	0.48	0.851	0.91	0.35	2.36
Postoperative infection	DGKQ	Wald ratio	1	0.08	0.42	0.852	1.08	0.47	2.47
Bronchiectasis	DGKQ	Wald ratio	1	-0.12	0.65	0.853	0.89	0.25	3.15
Ovarian cyst	DGKQ	Wald ratio	1	0.08	0.41	0.855	1.08	0.48	2.42
Thyrotoxicosis with or without goiter	DGKQ	Wald ratio	1	-0.12	0.65	0.856	0.89	0.25	3.18
Amblyopia	DGKQ	Wald ratio	1	-0.22	1.20	0.856	0.80	0.08	8.45
Hemiplegia	DGKQ	Wald ratio	1	0.13	0.72	0.857	1.14	0.28	4.71
Peripheral vascular disease, unspecified	DGKQ	Wald ratio	1	0.10	0.56	0.859	1.10	0.37	3.30
Hyposmolality and/or hyponatremia	DGKQ	Wald ratio	1	0.12	0.66	0.859	1.12	0.31	4.07
Derangement of joint, non-traumatic	DGKQ	Wald ratio	1	0.09	0.52	0.861	1.10	0.39	3.04
Diseases of pulp and periapical tissues	DGKQ	Wald ratio	1	0.11	0.66	0.862	1.12	0.31	4.08
Swelling, mass, or lump in head and neck [Space-occupying lesion, intracranial NOS]	DGKQ	Wald ratio	1	0.16	0.93	0.862	1.17	0.19	7.22
Hypotension	DGKQ	Wald ratio	1	0.06	0.37	0.862	1.07	0.52	2.19
Elevated blood pressure reading without diagnosis of hypertension	DGKQ	Wald ratio	1	0.12	0.73	0.864	1.13	0.27	4.70
Convulsions	DGKQ	Wald ratio	1	0.10	0.59	0.866	1.11	0.35	3.54
Polymyalgia Rheumatica	DGKQ	Wald ratio	1	-0.14	0.83	0.866	0.87	0.17	4.40
Disorders of vitreous body	DGKQ	Wald ratio	1	0.13	0.76	0.866	1.14	0.26	5.00
Carditis	DGKQ	Wald ratio	1	-0.10	0.62	0.868	0.90	0.27	3.06
Dementias	DGKQ	Wald ratio	1	-0.15	0.90	0.872	0.86	0.15	5.06
Other disorders of liver	DGKQ	Wald ratio	1	-0.06	0.37	0.873	0.94	0.46	1.94
Other disorders of bladder	DGKQ	Wald ratio	1	-0.05	0.29	0.873	0.96	0.54	1.68
Frequency of urination and polyuria	DGKQ	Wald ratio	1	-0.07	0.44	0.873	0.93	0.39	2.22

Malignant neoplasm of uterus	DGKQ	Wald ratio	1	-0.13	0.79	0.874	0.88	0.19	4.11
Benign neoplasm of other parts of digestive system	DGKQ	Wald ratio	1	0.06	0.39	0.878	1.06	0.49	2.28
Obstetrical/birth trauma	DGKQ	Wald ratio	1	-0.07	0.43	0.878	0.94	0.40	2.17
Other and unspecified disc disorder	DGKQ	Wald ratio	1	-0.06	0.42	0.880	0.94	0.41	2.16
Obstruction of bile duct	DGKQ	Wald ratio	1	-0.15	1.00	0.880	0.86	0.12	6.16
Renal failure NOS	DGKQ	Wald ratio	1	-0.11	0.74	0.881	0.89	0.21	3.84
Facial nerve disorders [CN7]	DGKQ	Wald ratio	1	-0.14	0.94	0.882	0.87	0.14	5.52
Symptoms involving nervous and musculoskeletal systems	DGKQ	Wald ratio	1	-0.09	0.65	0.885	0.91	0.26	3.24
Migraine	DGKQ	Wald ratio	1	0.08	0.53	0.885	1.08	0.38	3.04
Epilepsy	DGKQ	Wald ratio	1	0.13	0.93	0.887	1.14	0.18	7.06
Type 2 diabetes	DGKQ	Wald ratio	1	0.03	0.22	0.889	1.03	0.67	1.59
Orthostatic hypotension	DGKQ	Wald ratio	1	-0.11	0.76	0.889	0.90	0.20	4.02
Rheumatic disease of the heart valves	DGKQ	Wald ratio	1	0.06	0.40	0.892	1.06	0.48	2.33
Premature beats	DGKQ	Wald ratio	1	0.16	1.20	0.892	1.18	0.11	12.37
Otitis media and Eustachian tube disorders	DGKQ	Wald ratio	1	-0.08	0.59	0.893	0.92	0.29	2.95
Peripheral vascular disease	DGKQ	Wald ratio	1	0.06	0.45	0.893	1.06	0.44	2.57
Malignant neoplasm of kidney, except pelvis	DGKQ	Wald ratio	1	0.12	0.88	0.894	1.13	0.20	6.34
Lymphoid leukemia, chronic	DGKQ	Wald ratio	1	-0.16	1.24	0.896	0.85	0.08	9.59
Prurigo and Lichen	DGKQ	Wald ratio	1	-0.13	1.00	0.898	0.88	0.12	6.28
Leukemia	DGKQ	Wald ratio	1	-0.09	0.69	0.900	0.92	0.24	3.53
Fetal distress and abnormal forces of labor	DGKQ	Wald ratio	1	0.08	0.62	0.901	1.08	0.32	3.64
Neurological disorders	DGKQ	Wald ratio	1	0.05	0.41	0.901	1.05	0.47	2.36
Cervical intraepithelial neoplasia [CIN] [Cervical dysplasia]	DGKQ	Wald ratio	1	0.08	0.62	0.901	1.08	0.32	3.60
Abnormal findings examination of lungs	DGKQ	Wald ratio	1	0.07	0.56	0.905	1.07	0.36	3.21
Disorders of external ear	DGKQ	Wald ratio	1	-0.09	0.74	0.906	0.92	0.22	3.88
Cardiac arrest	DGKQ	Wald ratio	1	-0.11	0.92	0.908	0.90	0.15	5.44

Genital prolapse	DGKQ	Wald ratio	1	0.03	0.27	0.909	1.03	0.61	1.74
Other specified osteoporosis	DGKQ	Wald ratio	1	0.14	1.21	0.909	1.15	0.11	12.35
Intervertebral disc disorders	DGKQ	Wald ratio	1	0.03	0.30	0.910	1.03	0.58	1.86
Peritonitis and retroperitoneal infections	DGKQ	Wald ratio	1	-0.11	0.94	0.910	0.90	0.14	5.65
Peritoneal adhesions (postoperative) (postinfection)	DGKQ	Wald ratio	1	-0.06	0.50	0.911	0.95	0.35	2.53
Opiates and related narcotics causing adverse effects in therapeutic use	DGKQ	Wald ratio	1	0.09	0.83	0.913	1.09	0.22	5.52
Other diseases of the teeth and supporting structures	DGKQ	Wald ratio	1	0.06	0.54	0.915	1.06	0.37	3.05
Nonrheumatic mitral valve disorders	DGKQ	Wald ratio	1	-0.06	0.52	0.916	0.95	0.34	2.64
Other local infections of skin and subcutaneous tissue	DGKQ	Wald ratio	1	0.04	0.42	0.916	1.04	0.46	2.36
Mood disorders	DGKQ	Wald ratio	1	0.03	0.26	0.916	1.03	0.62	1.70
Atrial fibrillation and flutter	DGKQ	Wald ratio	1	-0.02	0.24	0.918	0.98	0.61	1.57
Cardiac and circulatory congenital anomalies	DGKQ	Wald ratio	1	-0.05	0.53	0.920	0.95	0.33	2.69
Otitis media	DGKQ	Wald ratio	1	0.07	0.66	0.921	1.07	0.29	3.88
Pneumococcal pneumonia	DGKQ	Wald ratio	1	-0.04	0.37	0.923	0.97	0.47	1.98
Abnormality of gait	DGKQ	Wald ratio	1	-0.07	0.70	0.923	0.93	0.24	3.67
Anal and rectal conditions	DGKQ	Wald ratio	1	-0.02	0.23	0.924	0.98	0.62	1.54
Delirium due to conditions classified elsewhere	DGKQ	Wald ratio	1	-0.10	1.09	0.925	0.90	0.11	7.67
Abnormal results of function study of liver	DGKQ	Wald ratio	1	0.05	0.48	0.925	1.05	0.41	2.67
Symptoms involving digestive system	DGKQ	Wald ratio	1	0.02	0.23	0.925	1.02	0.66	1.59
Complications of surgical and medical procedures	DGKQ	Wald ratio	1	-0.03	0.29	0.926	0.97	0.55	1.73
Fracture of hand or wrist	DGKQ	Wald ratio	1	0.04	0.48	0.928	1.04	0.41	2.69
Inflammatory disease of breast	DGKQ	Wald ratio	1	-0.09	1.04	0.929	0.91	0.12	7.02
Complication of colostomy or enterostomy	DGKQ	Wald ratio	1	-0.11	1.25	0.931	0.90	0.08	10.35
Cancer of kidney and renal pelvis	DGKQ	Wald ratio	1	-0.07	0.86	0.932	0.93	0.17	5.05
Skull and face fracture and other intercranial injury	DGKQ	Wald ratio	1	0.04	0.52	0.933	1.04	0.38	2.88
Heart valve disorders	DGKQ	Wald ratio	1	-0.04	0.43	0.935	0.97	0.41	2.26

Right bundle branch block	DGKQ	Wald ratio	1	0.06	0.73	0.936	1.06	0.26	4.40
Cellulitis and abscess of fingers/toes	DGKQ	Wald ratio	1	0.09	1.17	0.937	1.10	0.11	10.95
Hemorrhage in early pregnancy	DGKQ	Wald ratio	1	-0.07	0.86	0.937	0.93	0.17	5.05
Bacterial pneumonia	DGKQ	Wald ratio	1	0.03	0.35	0.938	1.03	0.52	2.03
Epiphora	DGKQ	Wald ratio	1	-0.07	0.93	0.943	0.94	0.15	5.80
Endometrial hyperplasia	DGKQ	Wald ratio	1	0.06	0.83	0.943	1.06	0.21	5.43
Other paralytic syndromes	DGKQ	Wald ratio	1	0.08	1.09	0.944	1.08	0.13	9.09
Malignant neoplasm of bladder	DGKQ	Wald ratio	1	0.04	0.61	0.945	1.04	0.32	3.45
Carbuncle and furuncle	DGKQ	Wald ratio	1	-0.04	0.59	0.946	0.96	0.30	3.03
Bundle branch block	DGKQ	Wald ratio	1	-0.03	0.49	0.946	0.97	0.37	2.51
Hematuria	DGKQ	Wald ratio	1	-0.02	0.23	0.947	0.98	0.63	1.53
Unstable angina (intermediate coronary syndrome)	DGKQ	Wald ratio	1	-0.03	0.40	0.947	0.97	0.45	2.12
Altered mental status	DGKQ	Wald ratio	1	0.04	0.60	0.951	1.04	0.32	3.35
Ptosis of eyelid	DGKQ	Wald ratio	1	0.04	0.66	0.952	1.04	0.29	3.77
Poisoning by analgesics, antipyretics, and antirheumatics	DGKQ	Wald ratio	1	0.02	0.34	0.954	1.02	0.52	1.99
Ischemic Heart Disease	DGKQ	Wald ratio	1	-0.01	0.18	0.955	0.99	0.70	1.40
Polyarteritis nodosa and allied conditions	DGKQ	Wald ratio	1	0.05	0.97	0.956	1.06	0.16	7.09
Hemorrhage during pregnancy; childbirth and postpartum	DGKQ	Wald ratio	1	-0.04	0.65	0.956	0.97	0.27	3.44
Chronic sinusitis	DGKQ	Wald ratio	1	-0.03	0.55	0.957	0.97	0.33	2.86
Acquired foot deformities	DGKQ	Wald ratio	1	0.01	0.29	0.959	1.02	0.57	1.80
Disorders of fluid, electrolyte, and acid-base balance	DGKQ	Wald ratio	1	-0.02	0.33	0.960	0.98	0.52	1.87
Sepsis	DGKQ	Wald ratio	1	-0.03	0.53	0.961	0.97	0.35	2.74
Sepsis and SIRS	DGKQ	Wald ratio	1	-0.03	0.53	0.961	0.97	0.35	2.74
Electrolyte imbalance	DGKQ	Wald ratio	1	0.02	0.44	0.961	1.02	0.43	2.42
Other peripheral nerve disorders	DGKQ	Wald ratio	1	-0.01	0.26	0.962	0.99	0.60	1.64
Fracture of pelvis	DGKQ	Wald ratio	1	-0.05	1.04	0.964	0.95	0.12	7.34

Thrombocytopenia	DGKQ	Wald ratio	1	-0.03	0.71	0.964	0.97	0.24	3.87
Degenerative disease of the spinal cord	DGKQ	Wald ratio	1	-0.03	0.66	0.966	0.97	0.27	3.54
Other specified gastritis	DGKQ	Wald ratio	1	0.01	0.32	0.969	1.01	0.54	1.88
Voice disturbance	DGKQ	Wald ratio	1	0.03	0.85	0.969	1.03	0.20	5.44
Cardiac conduction disorders	DGKQ	Wald ratio	1	-0.01	0.34	0.969	0.99	0.50	1.93
Mitral valve disease	DGKQ	Wald ratio	1	-0.02	0.51	0.969	0.98	0.36	2.69
Colorectal cancer	DGKQ	Wald ratio	1	-0.02	0.42	0.969	0.98	0.43	2.24
Appendiceal conditions	DGKQ	Wald ratio	1	0.02	0.48	0.970	1.02	0.39	2.63
Inflammatory and toxic neuropathy	DGKQ	Wald ratio	1	0.03	0.75	0.970	1.03	0.23	4.52
Renal failure	DGKQ	Wald ratio	1	0.01	0.34	0.971	1.01	0.52	1.96
Iron deficiency anemias	DGKQ	Wald ratio	1	0.01	0.32	0.971	1.01	0.54	1.90
Adverse drug events and drug allergies	DGKQ	Wald ratio	1	-0.04	1.06	0.971	0.96	0.12	7.72
Benign neoplasm of unspecified sites	DGKQ	Wald ratio	1	0.02	0.57	0.972	1.02	0.33	3.12
Other chronic ischemic heart disease, unspecified	DGKQ	Wald ratio	1	-0.01	0.24	0.974	0.99	0.61	1.60
Paroxysmal supraventricular tachycardia	DGKQ	Wald ratio	1	0.02	0.58	0.976	1.02	0.33	3.17
Hematemesis	DGKQ	Wald ratio	1	-0.02	0.63	0.977	0.98	0.28	3.41
Decubitus ulcer	DGKQ	Wald ratio	1	0.02	0.93	0.981	1.02	0.17	6.27
Schizophrenia and other psychotic disorders	DGKQ	Wald ratio	1	0.02	0.96	0.984	1.02	0.16	6.67
Cholecystitis without cholelithiasis	DGKQ	Wald ratio	1	0.01	0.54	0.985	1.01	0.35	2.89
Acute renal failure	DGKQ	Wald ratio	1	-0.01	0.42	0.985	0.99	0.44	2.26
Cancer of urinary organs (incl. kidney and bladder)	DGKQ	Wald ratio	1	-0.01	0.44	0.986	0.99	0.42	2.34
Respiratory failure, insufficiency, arrest	DGKQ	Wald ratio	1	-0.01	0.56	0.988	0.99	0.33	2.95
Infection of the eye	DGKQ	Wald ratio	1	0.02	1.18	0.988	1.02	0.10	10.27
Femoral hernia	DGKQ	Wald ratio	1	-0.02	1.10	0.988	0.98	0.11	8.45
Diabetes mellitus	DGKQ	Wald ratio	1	0.00	0.21	0.989	1.00	0.66	1.52
Noninflammatory female genital disorders	DGKQ	Wald ratio	1	0.00	0.30	0.989	1.00	0.56	1.78

Osteoporosis	DGKQ	Wald ratio	1	0.00	0.36	0.990	1.00	0.50	2.02
Intestinal infection due to C. difficile	DGKQ	Wald ratio	1	-0.01	1.09	0.992	0.99	0.12	8.44
Ileostomy status	DGKQ	Wald ratio	1	-0.01	0.69	0.992	0.99	0.26	3.84
Chronic renal failure [CKD]	DGKQ	Wald ratio	1	0.00	0.55	0.993	1.00	0.34	2.91
Ascites (non malignant)	DGKQ	Wald ratio	1	0.00	0.71	0.995	1.00	0.25	4.03
Gastric ulcer	DGKQ	Wald ratio	1	0.00	0.44	0.995	1.00	0.42	2.36
Duodenal ulcer	DGKQ	Wald ratio	1	0.00	0.52	0.995	1.00	0.36	2.76
Otitis externa	DGKQ	Wald ratio	1	-0.01	1.22	0.997	0.99	0.09	10.95
Respiratory failure	DGKQ	Wald ratio	1	0.00	0.62	0.997	1.00	0.29	3.39
Dyschromia and Vitiligo	DGKQ	Wald ratio	1	0.00	0.89	0.997	1.00	0.17	5.72
Umbilical hernia	DGKQ	Wald ratio	1	0.00	0.46	0.998	1.00	0.40	2.48
Disorders of parathyroid gland	DGKQ	Wald ratio	1	0.00	0.95	0.999	1.00	0.16	6.39

Supplementary Table 8: Phenome-wide MR results for brain GPNMB.

outcome	exposure	method	nsnp	b	se	pval	or	or_lci95	or_uci95
Stricture of artery	GPNMB	Wald ratio	1	0.75	0.19	1.055E-04	2.11	1.45	3.08
Atherosclerosis of the extremities	GPNMB	Wald ratio	1	0.76	0.20	1.496E-04	2.14	1.44	3.16
Other disorders of arteries and arterioles	GPNMB	Wald ratio	1	0.58	0.16	2.133E-04	1.78	1.31	2.42
Peripheral vascular disease, unspecified	GPNMB	Wald ratio	1	0.38	0.11	8.369E-04	1.46	1.17	1.82
Atherosclerosis	GPNMB	Wald ratio	1	0.52	0.16	8.581E-04	1.69	1.24	2.30
Arterial embolism and thrombosis	GPNMB	Wald ratio	1	0.61	0.19	1.156E-03	1.84	1.27	2.66
Peripheral vascular disease	GPNMB	Wald ratio	1	0.28	0.09	2.343E-03	1.32	1.10	1.58
Arterial embolism and thrombosis of lower extremity artery	GPNMB	Wald ratio	1	0.71	0.24	3.101E-03	2.04	1.27	3.27
Cellulitis and abscess of foot, toe	GPNMB	Wald ratio	1	0.23	0.08	3.186E-03	1.26	1.08	1.46
Carcinoma in situ of skin	GPNMB	Wald ratio	1	0.65	0.22	3.270E-03	1.91	1.24	2.95
Cellulitis and abscess of leg, except foot	GPNMB	Wald ratio	1	0.23	0.08	3.519E-03	1.25	1.08	1.46
Postoperative infection	GPNMB	Wald ratio	1	0.25	0.09	3.688E-03	1.28	1.08	1.52
Atrioventricular block, complete	GPNMB	Wald ratio	1	0.67	0.24	4.472E-03	1.95	1.23	3.10
Cellulitis and abscess of arm/hand	GPNMB	Wald ratio	1	0.22	0.08	4.781E-03	1.24	1.07	1.45
Other inflammatory spondylopathies	GPNMB	Wald ratio	1	-0.39	0.14	5.338E-03	0.68	0.52	0.89
Lymphadenitis	GPNMB	Wald ratio	1	0.31	0.11	5.614E-03	1.36	1.09	1.69
Complications of labor and delivery NEC	GPNMB	Wald ratio	1	-0.31	0.12	8.836E-03	0.73	0.58	0.92
Intestinal obstruction without mention of hernia	GPNMB	Wald ratio	1	0.23	0.09	9.251E-03	1.26	1.06	1.51
Ascites (non malignant)	GPNMB	Wald ratio	1	0.36	0.14	0.012	1.44	1.09	1.91
Dysphagia	GPNMB	Wald ratio	1	-0.18	0.07	0.012	0.84	0.73	0.96
Other intestinal obstruction	GPNMB	Wald ratio	1	0.24	0.10	0.013	1.28	1.05	1.55
Other disorders of eye	GPNMB	Wald ratio	1	0.22	0.09	0.014	1.25	1.05	1.50
Other acquired musculoskeletal deformity	GPNMB	Wald ratio	1	0.32	0.13	0.015	1.37	1.06	1.77
Vertiginous syndromes and other disorders of vestibular system	GPNMB	Wald ratio	1	0.18	0.07	0.016	1.19	1.03	1.38

Bacterial enteritis	GPNMB	Wald ratio	1	0.26	0.11	0.017	1.30	1.05	1.60
stress incontinence, female	GPNMB	Wald ratio	1	-0.18	0.08	0.018	0.84	0.72	0.97
Musculoskeletal symptoms referable to limbs	GPNMB	Wald ratio	1	0.18	0.08	0.019	1.20	1.03	1.40
Bladder neck obstruction	GPNMB	Wald ratio	1	-0.30	0.13	0.019	0.74	0.57	0.95
Vascular insufficiency of intestine	GPNMB	Wald ratio	1	0.55	0.24	0.020	1.73	1.09	2.76
Functional disorders of bladder	GPNMB	Wald ratio	1	-0.32	0.14	0.022	0.73	0.55	0.95
Other symptoms	GPNMB	Wald ratio	1	0.37	0.16	0.022	1.45	1.05	1.99
Diseases of hard tissues of teeth	GPNMB	Wald ratio	1	-0.23	0.10	0.023	0.79	0.65	0.97
Endometriosis	GPNMB	Wald ratio	1	0.21	0.09	0.024	1.23	1.03	1.47
Symptoms involving female genital tract	GPNMB	Wald ratio	1	-0.16	0.07	0.024	0.85	0.74	0.98
Osteoporosis NOS	GPNMB	Wald ratio	1	-0.17	0.08	0.026	0.84	0.72	0.98
Superficial cellulitis and abscess	GPNMB	Wald ratio	1	0.15	0.07	0.026	1.16	1.02	1.32
Ulcerative colitis	GPNMB	Wald ratio	1	0.22	0.10	0.029	1.25	1.02	1.53
Aphakia and other disorders of lens	GPNMB	Wald ratio	1	0.29	0.13	0.030	1.34	1.03	1.74
Joint effusions	GPNMB	Wald ratio	1	-0.33	0.15	0.030	0.72	0.54	0.97
Dyspareunia	GPNMB	Wald ratio	1	-0.36	0.17	0.031	0.70	0.51	0.97
Hypertrophy of breast (Gynecomastia)	GPNMB	Wald ratio	1	0.42	0.20	0.033	1.52	1.04	2.22
Acquired spondylolisthesis	GPNMB	Wald ratio	1	0.31	0.15	0.033	1.36	1.03	1.81
Benign neoplasm of lip, oral cavity, and pharynx	GPNMB	Wald ratio	1	0.38	0.18	0.034	1.47	1.03	2.09
Dental caries	GPNMB	Wald ratio	1	-0.22	0.10	0.035	0.80	0.66	0.98
Other disorders of bladder	GPNMB	Wald ratio	1	-0.12	0.06	0.036	0.89	0.79	0.99
Poisoning by analgesics, antipyretics, and antirheumatics	GPNMB	Wald ratio	1	-0.14	0.07	0.044	0.87	0.76	1.00
Inflammatory diseases of female pelvic organs	GPNMB	Wald ratio	1	-0.15	0.07	0.045	0.86	0.75	1.00
Pain and other symptoms associated with female genital organs	GPNMB	Wald ratio	1	-0.20	0.11	0.051	0.81	0.66	1.00
Diplopia and disorders of binocular vision	GPNMB	Wald ratio	1	-0.41	0.21	0.052	0.66	0.44	1.00
Osteoporosis	GPNMB	Wald ratio	1	-0.14	0.07	0.053	0.87	0.75	1.00

Disorders of conjunctiva	GPNMB	Wald ratio	1	0.40	0.21	0.053	1.49	0.99	2.23
Other diseases of blood and blood-forming organs	GPNMB	Wald ratio	1	0.17	0.09	0.057	1.18	0.99	1.41
Spinal stenosis	GPNMB	Wald ratio	1	0.18	0.09	0.060	1.19	0.99	1.44
Pilonidal cyst	GPNMB	Wald ratio	1	-0.44	0.24	0.061	0.64	0.41	1.02
Hydronephrosis	GPNMB	Wald ratio	1	0.24	0.13	0.063	1.27	0.99	1.64
Rheumatoid arthritis and other inflammatory polyarthropathies	GPNMB	Wald ratio	1	-0.15	0.08	0.063	0.86	0.73	1.01
Poisoning/allergy of sulfonamides	GPNMB	Wald ratio	1	0.36	0.19	0.063	1.43	0.98	2.09
Aneurysm and dissection of heart	GPNMB	Wald ratio	1	0.40	0.21	0.064	1.49	0.98	2.27
Umbilical hernia	GPNMB	Wald ratio	1	-0.17	0.09	0.065	0.84	0.70	1.01
Epiphora	GPNMB	Wald ratio	1	-0.35	0.19	0.067	0.71	0.49	1.02
Other complications of pregnancy NEC	GPNMB	Wald ratio	1	0.23	0.13	0.067	1.26	0.98	1.61
Cardiac conduction disorders	GPNMB	Wald ratio	1	0.13	0.07	0.071	1.13	0.99	1.30
Swelling of limb	GPNMB	Wald ratio	1	0.15	0.08	0.076	1.16	0.98	1.37
Septicemia	GPNMB	Wald ratio	1	0.16	0.09	0.076	1.17	0.98	1.40
Constipation	GPNMB	Wald ratio	1	0.10	0.06	0.077	1.11	0.99	1.24
Other disorders of liver	GPNMB	Wald ratio	1	0.13	0.08	0.078	1.14	0.99	1.32
Breast conditions, congenital or relating to hormones	GPNMB	Wald ratio	1	0.32	0.19	0.080	1.38	0.96	1.99
Cholecystitis without cholelithiasis	GPNMB	Wald ratio	1	0.19	0.11	0.081	1.21	0.98	1.50
Blood in stool	GPNMB	Wald ratio	1	0.19	0.11	0.082	1.21	0.98	1.51
Intestinal infection	GPNMB	Wald ratio	1	0.10	0.06	0.083	1.11	0.99	1.25
Peritoneal or intestinal adhesions	GPNMB	Wald ratio	1	0.34	0.20	0.083	1.41	0.96	2.07
Disorders of vitreous body	GPNMB	Wald ratio	1	0.26	0.15	0.085	1.30	0.96	1.76
Torus fracture	GPNMB	Wald ratio	1	-0.24	0.14	0.085	0.79	0.60	1.03
Other disorders of the kidney and ureters	GPNMB	Wald ratio	1	0.17	0.10	0.088	1.18	0.98	1.44
Dizziness and giddiness (Light-headedness and vertigo)	GPNMB	Wald ratio	1	0.14	0.08	0.090	1.15	0.98	1.36
Other tests	GPNMB	Wald ratio	1	0.13	0.07	0.091	1.13	0.98	1.31

Erythematous conditions	GPNMB	Wald ratio	1	0.20	0.12	0.092	1.22	0.97	1.52
Diseases of hair and hair follicles	GPNMB	Wald ratio	1	0.13	0.08	0.092	1.14	0.98	1.33
Abnormal findings on mammogram or breast exam	GPNMB	Wald ratio	1	-0.24	0.14	0.093	0.79	0.59	1.04
Mechanical complication of unspecified genitourinary device, implant, and graft	GPNMB	Wald ratio	1	-0.27	0.16	0.095	0.76	0.56	1.05
Other peripheral nerve disorders	GPNMB	Wald ratio	1	-0.09	0.05	0.100	0.92	0.83	1.02
Acid-base balance disorder	GPNMB	Wald ratio	1	0.27	0.17	0.100	1.32	0.95	1.82
Benign neoplasm of breast	GPNMB	Wald ratio	1	0.24	0.14	0.102	1.27	0.95	1.68
Palpitations	GPNMB	Wald ratio	1	0.15	0.09	0.104	1.16	0.97	1.40
Pernicious anemia	GPNMB	Wald ratio	1	-0.33	0.21	0.107	0.72	0.48	1.08
Hyposmolality and/or hyponatremia	GPNMB	Wald ratio	1	0.21	0.13	0.107	1.24	0.95	1.61
Chemotherapy	GPNMB	Wald ratio	1	0.06	0.04	0.107	1.07	0.99	1.15
Diseases of sebaceous glands	GPNMB	Wald ratio	1	0.10	0.06	0.113	1.10	0.98	1.24
Other cerebral degenerations	GPNMB	Wald ratio	1	-0.27	0.17	0.113	0.76	0.55	1.07
Altered mental status	GPNMB	Wald ratio	1	0.19	0.12	0.114	1.21	0.96	1.54
Osteoarthritis; localized	GPNMB	Wald ratio	1	0.07	0.04	0.114	1.07	0.98	1.17
Other nonmalignant breast conditions	GPNMB	Wald ratio	1	0.20	0.13	0.115	1.23	0.95	1.58
Benign mammary dysplasias	GPNMB	Wald ratio	1	0.16	0.10	0.115	1.17	0.96	1.42
Pneumococcal pneumonia	GPNMB	Wald ratio	1	-0.12	0.07	0.116	0.89	0.77	1.03
Osteoarthrosis	GPNMB	Wald ratio	1	0.06	0.04	0.116	1.06	0.99	1.14
Acute renal failure	GPNMB	Wald ratio	1	0.13	0.09	0.116	1.14	0.97	1.35
Paroxysmal ventricular tachycardia	GPNMB	Wald ratio	1	-0.29	0.19	0.117	0.75	0.52	1.08
Actinic keratosis	GPNMB	Wald ratio	1	0.17	0.11	0.120	1.19	0.96	1.48
Hypopotassemia	GPNMB	Wald ratio	1	-0.23	0.15	0.122	0.79	0.59	1.06
Sebaceous cyst	GPNMB	Wald ratio	1	0.10	0.06	0.123	1.10	0.97	1.24
Acidosis	GPNMB	Wald ratio	1	0.27	0.18	0.124	1.31	0.93	1.84

Diverticulosis and diverticulitis	GPNMB	Wald ratio	1	0.06	0.04	0.126	1.06	0.98	1.14
Abnormal findings examination of lungs	GPNMB	Wald ratio	1	0.17	0.11	0.126	1.19	0.95	1.49
Nerve root and plexus disorders	GPNMB	Wald ratio	1	0.26	0.17	0.126	1.29	0.93	1.80
Atrioventricular [AV] block	GPNMB	Wald ratio	1	0.19	0.12	0.126	1.21	0.95	1.54
Diverticulosis	GPNMB	Wald ratio	1	0.06	0.04	0.128	1.06	0.98	1.14
Pain in joint	GPNMB	Wald ratio	1	-0.10	0.06	0.132	0.91	0.80	1.03
Epilepsy	GPNMB	Wald ratio	1	0.28	0.19	0.134	1.33	0.92	1.92
Hematuria	GPNMB	Wald ratio	1	-0.07	0.05	0.135	0.93	0.85	1.02
Leukemia	GPNMB	Wald ratio	1	0.21	0.14	0.137	1.23	0.94	1.62
Cholelithiasis with acute cholecystitis	GPNMB	Wald ratio	1	0.22	0.15	0.140	1.24	0.93	1.66
Anxiety disorder	GPNMB	Wald ratio	1	0.11	0.07	0.141	1.11	0.97	1.28
Bacterial pneumonia	GPNMB	Wald ratio	1	-0.10	0.07	0.141	0.90	0.78	1.04
Sicca syndrome	GPNMB	Wald ratio	1	-0.37	0.25	0.142	0.69	0.42	1.13
Heartburn	GPNMB	Wald ratio	1	0.18	0.12	0.144	1.20	0.94	1.53
Aortic aneurysm	GPNMB	Wald ratio	1	0.22	0.15	0.146	1.25	0.93	1.69
Noninflammatory disorders of vagina	GPNMB	Wald ratio	1	0.20	0.14	0.147	1.22	0.93	1.59
Sepsis	GPNMB	Wald ratio	1	0.16	0.11	0.148	1.17	0.95	1.44
Sepsis and SIRS	GPNMB	Wald ratio	1	0.16	0.11	0.148	1.17	0.95	1.44
Osteoporosis, osteopenia and pathological fracture	GPNMB	Wald ratio	1	-0.10	0.07	0.149	0.91	0.80	1.03
Vitamin B-complex deficiencies	GPNMB	Wald ratio	1	-0.30	0.21	0.150	0.74	0.49	1.11
Respiratory abnormalities	GPNMB	Wald ratio	1	0.33	0.23	0.150	1.39	0.89	2.19
Nausea and vomiting	GPNMB	Wald ratio	1	0.08	0.05	0.150	1.08	0.97	1.20
Disorders of muscle, ligament, and fascia	GPNMB	Wald ratio	1	-0.12	0.09	0.154	0.88	0.75	1.05
Genitourinary congenital anomalies	GPNMB	Wald ratio	1	-0.20	0.14	0.159	0.82	0.62	1.08
Congenital anomalies of genital organs	GPNMB	Wald ratio	1	-0.30	0.21	0.162	0.74	0.49	1.13
Other disorders of soft tissues	GPNMB	Wald ratio	1	0.10	0.07	0.162	1.11	0.96	1.28

Chronic sinusitis	GPNMB	Wald ratio	1	-0.16	0.11	0.166	0.86	0.69	1.07
Ileostomy status	GPNMB	Wald ratio	1	0.19	0.14	0.166	1.21	0.92	1.60
Complication of internal orthopedic device	GPNMB	Wald ratio	1	-0.14	0.10	0.166	0.87	0.71	1.06
Diseases of the oral soft tissues, excluding lesions specific for gingiva and tongue	GPNMB	Wald ratio	1	0.13	0.09	0.168	1.13	0.95	1.36
Other specified benign mammary dysplasias	GPNMB	Wald ratio	1	0.30	0.22	0.168	1.35	0.88	2.05
Pain	GPNMB	Wald ratio	1	-0.28	0.20	0.169	0.75	0.51	1.13
Cervical intraepithelial neoplasia [CIN] [Cervical dysplasia]	GPNMB	Wald ratio	1	0.17	0.13	0.173	1.19	0.93	1.52
Gastritis and duodenitis	GPNMB	Wald ratio	1	0.05	0.04	0.175	1.05	0.98	1.13
Shortness of breath	GPNMB	Wald ratio	1	0.10	0.08	0.175	1.11	0.96	1.28
Glomerulonephritis	GPNMB	Wald ratio	1	0.24	0.18	0.175	1.27	0.90	1.80
Benign neoplasm of unspecified sites	GPNMB	Wald ratio	1	0.16	0.12	0.176	1.17	0.93	1.47
Nephritis; nephrosis; renal sclerosis	GPNMB	Wald ratio	1	0.20	0.15	0.177	1.22	0.91	1.62
Pathologic fracture	GPNMB	Wald ratio	1	-0.34	0.25	0.181	0.71	0.44	1.17
Unspecified diffuse connective tissue disease	GPNMB	Wald ratio	1	0.15	0.11	0.181	1.16	0.93	1.44
Benign neoplasm of ovary	GPNMB	Wald ratio	1	-0.20	0.15	0.181	0.82	0.61	1.10
Fracture of foot	GPNMB	Wald ratio	1	0.18	0.13	0.182	1.19	0.92	1.55
Pericarditis	GPNMB	Wald ratio	1	-0.21	0.16	0.184	0.81	0.59	1.11
Lump or mass in breast	GPNMB	Wald ratio	1	-0.19	0.15	0.186	0.82	0.62	1.10
Other chronic ischemic heart disease, unspecified	GPNMB	Wald ratio	1	0.07	0.05	0.186	1.07	0.97	1.18
Osteopenia or other disorder of bone and cartilage	GPNMB	Wald ratio	1	0.26	0.20	0.187	1.30	0.88	1.92
Right bundle branch block	GPNMB	Wald ratio	1	0.19	0.15	0.188	1.21	0.91	1.62
Myopia	GPNMB	Wald ratio	1	-0.21	0.16	0.188	0.81	0.59	1.11
Other abnormal blood chemistry	GPNMB	Wald ratio	1	-0.10	0.07	0.188	0.91	0.79	1.05
Rheumatoid arthritis	GPNMB	Wald ratio	1	-0.11	0.09	0.189	0.89	0.75	1.06
Poisoning by psychotropic agents	GPNMB	Wald ratio	1	0.17	0.13	0.191	1.19	0.92	1.53

Acquired toe deformities	GPNMB	Wald ratio	1	-0.11	0.08	0.191	0.90	0.77	1.05
Inflammatory disease of breast	GPNMB	Wald ratio	1	0.28	0.21	0.192	1.32	0.87	2.00
Diseases of esophagus	GPNMB	Wald ratio	1	0.04	0.03	0.193	1.04	0.98	1.11
Esophagitis, GERD and related diseases	GPNMB	Wald ratio	1	0.04	0.03	0.195	1.05	0.98	1.12
Secondary malignant neoplasm of digestive systems	GPNMB	Wald ratio	1	0.19	0.15	0.195	1.21	0.91	1.61
Nonspecific findings on examination of blood	GPNMB	Wald ratio	1	-0.09	0.07	0.202	0.91	0.79	1.05
Abdominal aortic aneurysm	GPNMB	Wald ratio	1	0.24	0.19	0.203	1.27	0.88	1.85
Contusion	GPNMB	Wald ratio	1	-0.19	0.15	0.203	0.83	0.62	1.11
Pain in limb	GPNMB	Wald ratio	1	0.09	0.07	0.206	1.10	0.95	1.26
Cancer of urinary organs (incl. kidney and bladder)	GPNMB	Wald ratio	1	-0.11	0.09	0.206	0.89	0.75	1.06
Retinal vascular changes and abnomalities	GPNMB	Wald ratio	1	-0.25	0.20	0.207	0.78	0.53	1.15
Polyp of corpus uteri	GPNMB	Wald ratio	1	-0.08	0.07	0.209	0.92	0.81	1.05
Cancer of bladder	GPNMB	Wald ratio	1	-0.15	0.12	0.211	0.86	0.69	1.09
Malignant neoplasm of female breast	GPNMB	Wald ratio	1	0.07	0.05	0.212	1.07	0.96	1.19
Hallux rigidus	GPNMB	Wald ratio	1	-0.18	0.14	0.214	0.84	0.63	1.11
Sciatica	GPNMB	Wald ratio	1	-0.14	0.12	0.218	0.87	0.69	1.09
Cancer, suspected or other	GPNMB	Wald ratio	1	-0.06	0.04	0.219	0.95	0.87	1.03
Fracture of radius and ulna	GPNMB	Wald ratio	1	-0.10	0.08	0.220	0.91	0.78	1.06
Nonrheumatic mitral valve disorders	GPNMB	Wald ratio	1	0.13	0.11	0.223	1.14	0.92	1.40
Fever of unknown origin	GPNMB	Wald ratio	1	0.11	0.09	0.225	1.12	0.93	1.33
Complication of colostomy or enterostomy	GPNMB	Wald ratio	1	0.31	0.25	0.225	1.36	0.83	2.24
Abnormal results of function study of liver	GPNMB	Wald ratio	1	0.12	0.10	0.226	1.12	0.93	1.36
Other and unspecified disorders of back	GPNMB	Wald ratio	1	-0.15	0.13	0.231	0.86	0.67	1.10
Anorexia	GPNMB	Wald ratio	1	0.23	0.20	0.232	1.26	0.86	1.86
Disorder of skin and subcutaneous tissue NOS	GPNMB	Wald ratio	1	-0.09	0.07	0.232	0.91	0.79	1.06
Neoplasm of unspecified nature of digestive system	GPNMB	Wald ratio	1	-0.21	0.17	0.233	0.81	0.58	1.14

Disorders of menstruation and other abnormal bleeding from female genital tract	GPNMB	Wald ratio	1	-0.05	0.05	0.233	0.95	0.87	1.04
Megaloblastic anemia	GPNMB	Wald ratio	1	-0.20	0.17	0.238	0.82	0.58	1.15
Simple and unspecified goiter	GPNMB	Wald ratio	1	0.27	0.23	0.239	1.31	0.83	2.07
Pneumonia	GPNMB	Wald ratio	1	-0.07	0.06	0.239	0.93	0.83	1.05
Chronic pancreatitis	GPNMB	Wald ratio	1	0.29	0.25	0.243	1.34	0.82	2.19
Infection of the eye	GPNMB	Wald ratio	1	-0.28	0.24	0.244	0.76	0.47	1.21
Lymphoid leukemia, chronic	GPNMB	Wald ratio	1	0.29	0.25	0.245	1.34	0.82	2.20
Abdominal pain	GPNMB	Wald ratio	1	-0.03	0.03	0.247	0.97	0.91	1.02
Chronic renal failure [CKD]	GPNMB	Wald ratio	1	0.13	0.11	0.248	1.14	0.91	1.41
Peritonitis and retroperitoneal infections	GPNMB	Wald ratio	1	0.22	0.19	0.248	1.25	0.86	1.81
Other deficiency anemia	GPNMB	Wald ratio	1	-0.20	0.17	0.249	0.82	0.59	1.15
Irregular menstrual cycle/bleeding	GPNMB	Wald ratio	1	-0.06	0.05	0.249	0.95	0.86	1.04
Endocarditis	GPNMB	Wald ratio	1	0.25	0.22	0.251	1.28	0.84	1.96
Pelvic peritoneal adhesions, female (postoperative) (postinfection)	GPNMB	Wald ratio	1	-0.13	0.11	0.251	0.88	0.70	1.10
Anxiety disorders	GPNMB	Wald ratio	1	0.08	0.07	0.251	1.08	0.94	1.24
Skull and face fracture and other intercranial injury	GPNMB	Wald ratio	1	-0.12	0.11	0.252	0.89	0.72	1.09
Other disorders of urethra and urinary tract	GPNMB	Wald ratio	1	0.10	0.09	0.252	1.11	0.93	1.32
Coronary atherosclerosis	GPNMB	Wald ratio	1	-0.05	0.04	0.253	0.95	0.87	1.04
Problems associated with amniotic cavity and membranes	GPNMB	Wald ratio	1	0.18	0.16	0.256	1.20	0.88	1.63
Other derangement of joint	GPNMB	Wald ratio	1	-0.14	0.13	0.259	0.87	0.67	1.11
Late pregnancy and failed induction	GPNMB	Wald ratio	1	-0.19	0.16	0.261	0.83	0.60	1.15
Vitamin deficiency	GPNMB	Wald ratio	1	-0.18	0.16	0.261	0.83	0.60	1.15
Other paralytic syndromes	GPNMB	Wald ratio	1	-0.25	0.22	0.262	0.78	0.51	1.20
Disturbances in tooth eruption	GPNMB	Wald ratio	1	-0.13	0.12	0.262	0.88	0.70	1.10
Malignant neoplasm of bladder	GPNMB	Wald ratio	1	-0.14	0.12	0.262	0.87	0.68	1.11

Congenital anomalies of great vessels	GPNMB	Wald ratio	1	0.15	0.13	0.263	1.16	0.89	1.51
Malignant neoplasm of uterus	GPNMB	Wald ratio	1	-0.18	0.16	0.264	0.84	0.61	1.14
Heart valve replaced	GPNMB	Wald ratio	1	0.16	0.15	0.265	1.18	0.88	1.57
Cyst of kidney, acquired	GPNMB	Wald ratio	1	0.18	0.16	0.266	1.20	0.87	1.64
Mood disorders	GPNMB	Wald ratio	1	0.06	0.05	0.266	1.06	0.96	1.17
Cancer of mouth	GPNMB	Wald ratio	1	0.25	0.22	0.267	1.28	0.83	1.99
Other disorders of stomach and duodenum	GPNMB	Wald ratio	1	0.11	0.10	0.268	1.12	0.92	1.35
Fracture of tibia and fibula	GPNMB	Wald ratio	1	-0.13	0.12	0.273	0.87	0.69	1.11
Renal failure	GPNMB	Wald ratio	1	0.07	0.07	0.275	1.08	0.94	1.23
Dementias	GPNMB	Wald ratio	1	0.20	0.18	0.279	1.22	0.85	1.75
Multiple myeloma	GPNMB	Wald ratio	1	0.26	0.24	0.280	1.30	0.81	2.08
Candidiasis	GPNMB	Wald ratio	1	-0.13	0.12	0.280	0.88	0.69	1.11
Phlebitis and thrombophlebitis of lower extremities	GPNMB	Wald ratio	1	-0.10	0.10	0.281	0.90	0.75	1.09
Pruritus and related conditions	GPNMB	Wald ratio	1	-0.22	0.20	0.282	0.80	0.54	1.20
Cancer within the respiratory system	GPNMB	Wald ratio	1	0.12	0.11	0.282	1.13	0.91	1.40
Stiffness of joint	GPNMB	Wald ratio	1	0.25	0.23	0.283	1.28	0.82	2.00
Other disorders of gallbladder	GPNMB	Wald ratio	1	0.16	0.15	0.285	1.18	0.87	1.58
Postmenopausal atrophic vaginitis	GPNMB	Wald ratio	1	0.18	0.17	0.285	1.20	0.86	1.68
Cancer of larynx, pharynx, nasal cavities	GPNMB	Wald ratio	1	0.24	0.23	0.288	1.27	0.82	1.98
Disorders of tooth development	GPNMB	Wald ratio	1	-0.12	0.11	0.290	0.89	0.71	1.11
Inflammatory disease of cervix, vagina, and vulva	GPNMB	Wald ratio	1	-0.11	0.11	0.290	0.89	0.72	1.10
Disorders of iron metabolism	GPNMB	Wald ratio	1	-0.23	0.22	0.294	0.79	0.52	1.22
Urethral stricture (not specified as infectious)	GPNMB	Wald ratio	1	0.10	0.10	0.295	1.11	0.91	1.35
Bacterial infection NOS	GPNMB	Wald ratio	1	0.05	0.05	0.296	1.06	0.95	1.17
Dysuria	GPNMB	Wald ratio	1	0.17	0.16	0.299	1.19	0.86	1.64
Cardiac pacemaker/device in situ	GPNMB	Wald ratio	1	0.12	0.11	0.299	1.13	0.90	1.41

Hemiplegia	GPNMB	Wald ratio	1	0.15	0.15	0.300	1.16	0.87	1.55
Traumatic arthropathy	GPNMB	Wald ratio	1	-0.26	0.25	0.301	0.77	0.48	1.26
Polyp of female genital organs	GPNMB	Wald ratio	1	-0.06	0.06	0.302	0.94	0.85	1.05
Chronic glomerulonephritis, NOS	GPNMB	Wald ratio	1	0.20	0.20	0.305	1.22	0.83	1.79
Diffuse diseases of connective tissue	GPNMB	Wald ratio	1	0.10	0.10	0.306	1.11	0.91	1.34
Pelvic inflammatory disease (PID)	GPNMB	Wald ratio	1	-0.19	0.19	0.307	0.83	0.57	1.19
Malignant neoplasm of testis	GPNMB	Wald ratio	1	-0.11	0.11	0.308	0.90	0.73	1.10
Obstruction of bile duct	GPNMB	Wald ratio	1	0.21	0.20	0.309	1.23	0.82	1.84
Gingival and periodontal diseases	GPNMB	Wald ratio	1	0.14	0.14	0.312	1.15	0.88	1.50
Breast cancer [female]	GPNMB	Wald ratio	1	0.05	0.05	0.313	1.06	0.95	1.17
Phlebitis and thrombophlebitis	GPNMB	Wald ratio	1	-0.09	0.09	0.313	0.91	0.76	1.09
Cancer of brain and nervous system	GPNMB	Wald ratio	1	0.25	0.25	0.313	1.28	0.79	2.08
Delirium dementia and amnestic and other cognitive disorders	GPNMB	Wald ratio	1	0.13	0.13	0.314	1.14	0.88	1.46
Intracranial hemorrhage (injury)	GPNMB	Wald ratio	1	-0.25	0.25	0.316	0.78	0.47	1.27
Cervicitis and endocervicitis	GPNMB	Wald ratio	1	-0.16	0.16	0.316	0.85	0.63	1.16
Alcohol-related disorders	GPNMB	Wald ratio	1	0.05	0.05	0.317	1.05	0.95	1.17
Viral warts & HPV	GPNMB	Wald ratio	1	-0.18	0.18	0.323	0.84	0.59	1.19
Bundle branch block	GPNMB	Wald ratio	1	0.10	0.10	0.323	1.10	0.91	1.34
Opiates and related narcotics causing adverse effects in therapeutic use	GPNMB	Wald ratio	1	-0.17	0.17	0.324	0.85	0.61	1.18
Raynaud's syndrome	GPNMB	Wald ratio	1	0.17	0.17	0.325	1.18	0.85	1.64
Convulsions	GPNMB	Wald ratio	1	-0.12	0.12	0.327	0.89	0.70	1.13
Fetal distress and abnormal forces of labor	GPNMB	Wald ratio	1	-0.12	0.13	0.328	0.88	0.69	1.13
Strabismus (not specified as paralytic)	GPNMB	Wald ratio	1	-0.18	0.19	0.330	0.83	0.58	1.20
Inflammatory bowel disease and other gastroenteritis and colitis	GPNMB	Wald ratio	1	0.08	0.09	0.331	1.09	0.92	1.29
Fracture of clavicle or scapula	GPNMB	Wald ratio	1	0.14	0.15	0.332	1.15	0.87	1.53
Osteoarthrosis NOS	GPNMB	Wald ratio	1	0.05	0.05	0.332	1.05	0.95	1.17

Other specified osteoporosis	GPNMB	Wald ratio	1	-0.24	0.25	0.332	0.79	0.49	1.28
Paroxysmal tachycardia, unspecified	GPNMB	Wald ratio	1	-0.10	0.10	0.333	0.91	0.74	1.11
Atrial fibrillation and flutter	GPNMB	Wald ratio	1	-0.05	0.05	0.333	0.95	0.86	1.05
Other congenital musculoskeletal anomalies	GPNMB	Wald ratio	1	0.23	0.23	0.335	1.25	0.79	1.98
Other disorders of eyelids	GPNMB	Wald ratio	1	-0.07	0.08	0.335	0.93	0.80	1.08
Respiratory failure	GPNMB	Wald ratio	1	0.12	0.13	0.337	1.13	0.88	1.45
Suppurative and unspecified otitis media	GPNMB	Wald ratio	1	-0.19	0.19	0.337	0.83	0.57	1.22
Rupture of synovium	GPNMB	Wald ratio	1	-0.22	0.23	0.337	0.80	0.51	1.26
Atopic/contact dermatitis due to other or unspecified	GPNMB	Wald ratio	1	0.12	0.12	0.338	1.13	0.88	1.44
Labyrinthitis	GPNMB	Wald ratio	1	0.20	0.21	0.338	1.22	0.81	1.82
Cardiac pacemaker in situ	GPNMB	Wald ratio	1	0.11	0.12	0.340	1.12	0.89	1.42
Cholelithiasis	GPNMB	Wald ratio	1	-0.05	0.05	0.341	0.95	0.86	1.05
Ingrowing nail	GPNMB	Wald ratio	1	0.17	0.18	0.342	1.19	0.83	1.70
Osteoarthrosis, localized, primary	GPNMB	Wald ratio	1	0.06	0.06	0.343	1.06	0.94	1.20
Visual disturbances	GPNMB	Wald ratio	1	-0.09	0.10	0.343	0.91	0.75	1.11
Depression	GPNMB	Wald ratio	1	0.05	0.05	0.343	1.05	0.95	1.17
Chronic tonsillitis and adenoiditis	GPNMB	Wald ratio	1	0.16	0.17	0.344	1.17	0.84	1.64
Otitis media and Eustachian tube disorders	GPNMB	Wald ratio	1	-0.11	0.12	0.348	0.89	0.71	1.13
Disorders of the pituitary gland and its hypothalamic control	GPNMB	Wald ratio	1	0.20	0.22	0.351	1.22	0.80	1.87
Periapical abscess	GPNMB	Wald ratio	1	-0.15	0.17	0.352	0.86	0.62	1.19
Benign neoplasm of uterus	GPNMB	Wald ratio	1	0.05	0.06	0.353	1.05	0.94	1.18
Abnormal sputum	GPNMB	Wald ratio	1	-0.11	0.12	0.365	0.90	0.70	1.14
Other biliary tract disease	GPNMB	Wald ratio	1	0.08	0.09	0.365	1.09	0.91	1.30
Arthropathy NOS	GPNMB	Wald ratio	1	0.03	0.03	0.367	1.03	0.97	1.10
Respiratory failure, insufficiency, arrest	GPNMB	Wald ratio	1	0.10	0.11	0.369	1.11	0.89	1.38
Injuries to the nervous system	GPNMB	Wald ratio	1	-0.14	0.16	0.370	0.87	0.64	1.18

Type 2 diabetes with neurological manifestations	GPNMB	Wald ratio	1	0.21	0.24	0.370	1.24	0.78	1.97
Other open wound of head and face	GPNMB	Wald ratio	1	-0.09	0.11	0.370	0.91	0.74	1.12
Miscarriage; stillbirth	GPNMB	Wald ratio	1	-0.07	0.08	0.371	0.93	0.79	1.09
Symptoms concerning nutrition, metabolism, and development	GPNMB	Wald ratio	1	-0.07	0.08	0.371	0.93	0.80	1.09
Degenerative disease of the spinal cord	GPNMB	Wald ratio	1	-0.12	0.13	0.372	0.89	0.68	1.15
Other specified gastritis	GPNMB	Wald ratio	1	0.06	0.06	0.373	1.06	0.93	1.20
Placenta previa and abruptio placenta	GPNMB	Wald ratio	1	0.15	0.16	0.375	1.16	0.84	1.60
Diabetic retinopathy	GPNMB	Wald ratio	1	0.14	0.16	0.375	1.15	0.85	1.56
Degenerative skin conditions and other dermatoses	GPNMB	Wald ratio	1	0.07	0.08	0.380	1.07	0.92	1.25
Heart valve disorders	GPNMB	Wald ratio	1	0.08	0.09	0.384	1.08	0.91	1.28
Inflammation of the eye	GPNMB	Wald ratio	1	0.09	0.10	0.384	1.09	0.90	1.33
Type 1 diabetes	GPNMB	Wald ratio	1	0.10	0.11	0.385	1.10	0.89	1.37
Delirium due to conditions classified elsewhere	GPNMB	Wald ratio	1	0.19	0.22	0.386	1.21	0.78	1.87
Other disorders of biliary tract	GPNMB	Wald ratio	1	0.15	0.18	0.388	1.16	0.82	1.65
Abnormal findings on exam of gastrointestinal tract/ abdominal area	GPNMB	Wald ratio	1	0.12	0.14	0.393	1.13	0.86	1.48
Symptoms of the muscles	GPNMB	Wald ratio	1	0.18	0.22	0.396	1.20	0.79	1.83
Fracture of upper limb	GPNMB	Wald ratio	1	-0.05	0.06	0.399	0.95	0.84	1.07
Breast cancer	GPNMB	Wald ratio	1	0.04	0.05	0.399	1.05	0.94	1.16
Functional digestive disorders	GPNMB	Wald ratio	1	0.03	0.04	0.400	1.03	0.96	1.12
Alcoholic liver damage	GPNMB	Wald ratio	1	0.17	0.20	0.400	1.18	0.80	1.75
Fasciitis	GPNMB	Wald ratio	1	-0.08	0.09	0.401	0.92	0.77	1.11
Chronic airway obstruction	GPNMB	Wald ratio	1	0.05	0.06	0.403	1.05	0.94	1.17
Disorders of lacrimal system	GPNMB	Wald ratio	1	-0.10	0.12	0.404	0.90	0.71	1.15
Abnormal findings on examination of urine	GPNMB	Wald ratio	1	0.08	0.10	0.406	1.08	0.90	1.31
Multiple sclerosis	GPNMB	Wald ratio	1	0.13	0.16	0.406	1.14	0.84	1.54
Adverse drug events and drug allergies	GPNMB	Wald ratio	1	0.18	0.22	0.407	1.20	0.78	1.83

Mitral valve disease	GPNMB	Wald ratio	1	0.09	0.10	0.408	1.09	0.89	1.34
Chronic liver disease and cirrhosis	GPNMB	Wald ratio	1	0.09	0.11	0.409	1.09	0.89	1.34
Disorders of esophageal motility	GPNMB	Wald ratio	1	0.19	0.23	0.409	1.20	0.77	1.87
Irregular menstrual bleeding	GPNMB	Wald ratio	1	-0.08	0.09	0.411	0.93	0.77	1.11
Back pain	GPNMB	Wald ratio	1	0.05	0.06	0.411	1.05	0.94	1.17
Fracture of ribs	GPNMB	Wald ratio	1	-0.17	0.20	0.414	0.85	0.57	1.26
Gram negative septicemia	GPNMB	Wald ratio	1	0.16	0.20	0.415	1.17	0.80	1.73
Cerebral ischemia	GPNMB	Wald ratio	1	0.09	0.11	0.416	1.09	0.89	1.34
Fracture of ankle and foot	GPNMB	Wald ratio	1	0.10	0.12	0.416	1.10	0.87	1.39
Primary/intrinsic cardiomyopathies	GPNMB	Wald ratio	1	-0.13	0.16	0.416	0.88	0.63	1.21
Viral infection	GPNMB	Wald ratio	1	0.09	0.11	0.418	1.09	0.88	1.35
Alcoholism	GPNMB	Wald ratio	1	0.05	0.06	0.419	1.05	0.93	1.19
Gastric ulcer	GPNMB	Wald ratio	1	0.07	0.09	0.420	1.08	0.90	1.28
Otitis media	GPNMB	Wald ratio	1	-0.11	0.13	0.420	0.90	0.69	1.17
Other symptoms of respiratory system	GPNMB	Wald ratio	1	0.05	0.06	0.421	1.05	0.93	1.18
Fracture of patella	GPNMB	Wald ratio	1	-0.16	0.21	0.422	0.85	0.57	1.27
Diseases of pulp and periapical tissues	GPNMB	Wald ratio	1	-0.11	0.13	0.423	0.90	0.69	1.17
Postinflammatory pulmonary fibrosis	GPNMB	Wald ratio	1	0.15	0.19	0.424	1.16	0.80	1.69
Fracture of lower limb	GPNMB	Wald ratio	1	-0.05	0.07	0.426	0.95	0.83	1.08
Myalgia and myositis unspecified	GPNMB	Wald ratio	1	-0.18	0.22	0.427	0.84	0.54	1.30
Calculus of ureter	GPNMB	Wald ratio	1	0.09	0.12	0.427	1.10	0.87	1.38
Other disorders of pancreatic internal secretion	GPNMB	Wald ratio	1	0.15	0.19	0.427	1.16	0.81	1.67
Unspecified polyarthropathy or polyarthritis	GPNMB	Wald ratio	1	0.08	0.10	0.427	1.08	0.89	1.31
Cervical cancer	GPNMB	Wald ratio	1	-0.11	0.14	0.428	0.89	0.68	1.18
Secondary malignant neoplasm of liver	GPNMB	Wald ratio	1	-0.09	0.11	0.431	0.92	0.74	1.14
Congestive heart failure (CHF) NOS	GPNMB	Wald ratio	1	-0.10	0.13	0.433	0.90	0.70	1.16

Other arthropathies	GPNMB	Wald ratio	1	0.03	0.03	0.434	1.03	0.96	1.09
Corneal opacity and other disorders of cornea	GPNMB	Wald ratio	1	-0.16	0.21	0.434	0.85	0.56	1.28
Other forms of chronic heart disease	GPNMB	Wald ratio	1	-0.10	0.13	0.437	0.90	0.69	1.17
Orthostatic hypotension	GPNMB	Wald ratio	1	-0.12	0.16	0.437	0.89	0.65	1.20
Elevated blood pressure reading without diagnosis of hypertension	GPNMB	Wald ratio	1	-0.11	0.15	0.437	0.89	0.67	1.19
Malignant neoplasm, other	GPNMB	Wald ratio	1	-0.04	0.05	0.439	0.97	0.88	1.06
Disturbance of skin sensation	GPNMB	Wald ratio	1	0.08	0.11	0.440	1.08	0.88	1.33
Cardiac and circulatory congenital anomalies	GPNMB	Wald ratio	1	0.08	0.11	0.443	1.09	0.88	1.34
Primary open angle glaucoma	GPNMB	Wald ratio	1	0.13	0.18	0.446	1.14	0.81	1.62
Swelling, mass, or lump in head and neck [Space-occupying lesion, intracranial NOS]	GPNMB	Wald ratio	1	-0.14	0.19	0.450	0.87	0.60	1.25
Intracranial hemorrhage	GPNMB	Wald ratio	1	0.10	0.13	0.450	1.11	0.85	1.44
Diseases of lips	GPNMB	Wald ratio	1	0.16	0.22	0.451	1.18	0.77	1.81
Intracerebral hemorrhage	GPNMB	Wald ratio	1	0.16	0.22	0.451	1.18	0.77	1.79
Cervicalgia	GPNMB	Wald ratio	1	0.10	0.13	0.452	1.11	0.85	1.44
Memory loss	GPNMB	Wald ratio	1	0.16	0.22	0.456	1.17	0.77	1.79
Migraine	GPNMB	Wald ratio	1	-0.08	0.11	0.457	0.92	0.75	1.14
Nonspecific abnormal findings on radiological and other examination of other intrathoracic organs (echocardiogram, etc)	GPNMB	Wald ratio	1	-0.18	0.24	0.457	0.84	0.52	1.34
Hemorrhoids	GPNMB	Wald ratio	1	-0.03	0.04	0.458	0.97	0.90	1.05
Cardiomyopathy	GPNMB	Wald ratio	1	-0.12	0.16	0.460	0.89	0.65	1.22
Scar conditions and fibrosis of skin	GPNMB	Wald ratio	1	0.09	0.12	0.460	1.09	0.87	1.37
Hypertrophy of female genital organs	GPNMB	Wald ratio	1	-0.12	0.16	0.461	0.89	0.65	1.22
Other and unspecified disc disorder	GPNMB	Wald ratio	1	0.06	0.09	0.464	1.07	0.90	1.26
Diaphragmatic hernia	GPNMB	Wald ratio	1	0.03	0.04	0.466	1.03	0.96	1.10
Cholelithiasis with other cholecystitis	GPNMB	Wald ratio	1	-0.06	0.08	0.469	0.94	0.81	1.10

Abnormal function study of cardiovascular system	GPNMB	Wald ratio	1	0.18	0.25	0.470	1.20	0.74	1.95
Decreased white blood cell count	GPNMB	Wald ratio	1	0.07	0.10	0.470	1.08	0.88	1.31
Neutropenia	GPNMB	Wald ratio	1	0.07	0.10	0.470	1.08	0.88	1.31
Diseases of the jaws	GPNMB	Wald ratio	1	-0.13	0.18	0.474	0.88	0.61	1.26
Osteomyelitis	GPNMB	Wald ratio	1	-0.17	0.24	0.475	0.84	0.53	1.35
Other diseases of respiratory system, NEC	GPNMB	Wald ratio	1	-0.04	0.06	0.475	0.96	0.85	1.08
Uterine leiomyoma	GPNMB	Wald ratio	1	0.04	0.06	0.475	1.04	0.93	1.17
Overweight, obesity and other hyperalimentation	GPNMB	Wald ratio	1	0.04	0.06	0.476	1.04	0.93	1.16
Anemia of chronic disease	GPNMB	Wald ratio	1	-0.15	0.21	0.478	0.86	0.56	1.31
Open-angle glaucoma	GPNMB	Wald ratio	1	0.13	0.18	0.479	1.13	0.80	1.60
Epilepsy, recurrent seizures, convulsions	GPNMB	Wald ratio	1	-0.06	0.08	0.479	0.94	0.81	1.11
Hammer toe (acquired)	GPNMB	Wald ratio	1	-0.09	0.13	0.480	0.91	0.71	1.18
Regional enteritis	GPNMB	Wald ratio	1	-0.10	0.14	0.481	0.91	0.69	1.19
Other specified nonpsychotic and/or transient mental disorders	GPNMB	Wald ratio	1	-0.16	0.22	0.483	0.85	0.55	1.33
Obesity	GPNMB	Wald ratio	1	0.04	0.06	0.483	1.04	0.93	1.16
Noninfectious gastroenteritis	GPNMB	Wald ratio	1	0.03	0.05	0.484	1.03	0.94	1.13
Hypoglycemia	GPNMB	Wald ratio	1	0.13	0.19	0.485	1.14	0.79	1.64
Epistaxis or throat hemorrhage	GPNMB	Wald ratio	1	0.08	0.12	0.486	1.08	0.86	1.36
Other retinal disorders	GPNMB	Wald ratio	1	0.06	0.09	0.487	1.07	0.89	1.28
Schizophrenia and other psychotic disorders	GPNMB	Wald ratio	1	-0.14	0.20	0.488	0.87	0.60	1.28
Ankylosing spondylitis	GPNMB	Wald ratio	1	-0.16	0.23	0.488	0.85	0.55	1.34
Excessive or frequent menstruation	GPNMB	Wald ratio	1	-0.04	0.06	0.488	0.96	0.85	1.08
Prolapse of vaginal vault after hysterectomy	GPNMB	Wald ratio	1	-0.17	0.25	0.488	0.84	0.51	1.37
Polymyalgia Rheumatica	GPNMB	Wald ratio	1	0.12	0.17	0.489	1.12	0.81	1.56
Ill-defined descriptions and complications of heart disease	GPNMB	Wald ratio	1	0.14	0.20	0.490	1.15	0.77	1.70
Symptoms involving head and neck	GPNMB	Wald ratio	1	-0.07	0.10	0.490	0.93	0.76	1.14

Peripheral enthesopathies and allied syndromes	GPNMB	Wald ratio	1	-0.03	0.05	0.491	0.97	0.88	1.06
Benign neoplasm of colon	GPNMB	Wald ratio	1	0.03	0.04	0.492	1.03	0.95	1.12
Congestive heart failure; nonhypertensive	GPNMB	Wald ratio	1	-0.05	0.08	0.493	0.95	0.81	1.11
Ulceration of the lower GI tract	GPNMB	Wald ratio	1	0.12	0.17	0.494	1.13	0.80	1.59
Cancer of bronchus; lung	GPNMB	Wald ratio	1	0.09	0.12	0.494	1.09	0.85	1.39
Other disorders of intestine	GPNMB	Wald ratio	1	-0.06	0.09	0.497	0.94	0.79	1.12
Appendicitis	GPNMB	Wald ratio	1	0.07	0.10	0.498	1.07	0.88	1.31
Infection/inflammation of internal prosthetic device; implant; and graft	GPNMB	Wald ratio	1	0.08	0.11	0.499	1.08	0.86	1.35
Obstetrical/birth trauma	GPNMB	Wald ratio	1	0.06	0.09	0.501	1.06	0.89	1.26
Arrhythmia (cardiac) NOS	GPNMB	Wald ratio	1	0.13	0.19	0.502	1.13	0.79	1.64
Degeneration of intervertebral disc	GPNMB	Wald ratio	1	0.07	0.11	0.503	1.07	0.87	1.33
Neurological disorders	GPNMB	Wald ratio	1	0.06	0.08	0.504	1.06	0.90	1.25
Lymphoid leukemia	GPNMB	Wald ratio	1	0.16	0.24	0.505	1.17	0.74	1.86
Rheumatic disease of the heart valves	GPNMB	Wald ratio	1	0.05	0.08	0.506	1.06	0.90	1.24
Staphylococcus infections	GPNMB	Wald ratio	1	0.07	0.10	0.507	1.07	0.88	1.31
Nasal polyps	GPNMB	Wald ratio	1	-0.07	0.10	0.510	0.94	0.77	1.14
Antepartum hemorrhage, abruptio placentae, and placenta previa	GPNMB	Wald ratio	1	-0.13	0.20	0.510	0.88	0.60	1.29
Nontoxic nodular goiter	GPNMB	Wald ratio	1	0.11	0.17	0.511	1.12	0.80	1.56
Burns	GPNMB	Wald ratio	1	-0.15	0.24	0.511	0.86	0.54	1.36
Respiratory insufficiency	GPNMB	Wald ratio	1	0.09	0.14	0.514	1.09	0.84	1.43
Unspecified monoarthritis	GPNMB	Wald ratio	1	0.03	0.05	0.516	1.03	0.94	1.13
Intervertebral disc disorders	GPNMB	Wald ratio	1	0.04	0.06	0.517	1.04	0.92	1.17
Primary angle-closure glaucoma	GPNMB	Wald ratio	1	-0.14	0.21	0.520	0.87	0.57	1.33
Renal failure NOS	GPNMB	Wald ratio	1	-0.10	0.15	0.520	0.91	0.67	1.22
Complication due to other implant and internal device	GPNMB	Wald ratio	1	0.06	0.10	0.520	1.07	0.88	1.30
Diseases of white blood cells	GPNMB	Wald ratio	1	0.06	0.09	0.521	1.06	0.89	1.27

Poisoning by other anti-infectives	GPNMB	Wald ratio	1	0.10	0.16	0.523	1.11	0.81	1.52
Subarachnoid hemorrhage	GPNMB	Wald ratio	1	-0.13	0.20	0.524	0.88	0.60	1.30
Digestive congenital anomalies	GPNMB	Wald ratio	1	0.14	0.21	0.525	1.15	0.75	1.74
Osteoarthrosis, generalized	GPNMB	Wald ratio	1	-0.15	0.23	0.529	0.86	0.55	1.36
Other chronic nonalcoholic liver disease	GPNMB	Wald ratio	1	0.09	0.14	0.529	1.09	0.83	1.44
Acute upper respiratory infections of multiple or unspecified sites	GPNMB	Wald ratio	1	0.07	0.12	0.530	1.08	0.86	1.36
Acquired foot deformities	GPNMB	Wald ratio	1	-0.04	0.06	0.531	0.96	0.86	1.08
Portal hypertension	GPNMB	Wald ratio	1	0.15	0.25	0.533	1.17	0.72	1.89
Cardiac shunt/ heart septal defect	GPNMB	Wald ratio	1	-0.14	0.23	0.537	0.87	0.55	1.37
Other signs and symptoms in breast	GPNMB	Wald ratio	1	0.13	0.22	0.539	1.14	0.75	1.74
Hematemesis	GPNMB	Wald ratio	1	-0.08	0.13	0.540	0.92	0.72	1.19
Cardiac congenital anomalies	GPNMB	Wald ratio	1	0.07	0.11	0.542	1.07	0.86	1.33
Pleurisy; pleural effusion	GPNMB	Wald ratio	1	0.04	0.07	0.543	1.04	0.91	1.20
Precordial pain	GPNMB	Wald ratio	1	-0.06	0.10	0.544	0.94	0.78	1.14
Chronic cystitis	GPNMB	Wald ratio	1	0.11	0.19	0.546	1.12	0.77	1.63
Nonspecific chest pain	GPNMB	Wald ratio	1	0.02	0.03	0.546	1.02	0.95	1.09
Myocardial infarction	GPNMB	Wald ratio	1	-0.03	0.06	0.551	0.97	0.87	1.08
Septal Deviations/Turbinate Hypertrophy	GPNMB	Wald ratio	1	0.05	0.08	0.551	1.05	0.89	1.23
Chronic ulcer of skin	GPNMB	Wald ratio	1	0.08	0.13	0.553	1.08	0.84	1.38
Aseptic necrosis of bone	GPNMB	Wald ratio	1	0.14	0.24	0.554	1.16	0.72	1.86
Retinal detachments and defects	GPNMB	Wald ratio	1	-0.06	0.10	0.555	0.94	0.77	1.15
Frequency of urination and polyuria	GPNMB	Wald ratio	1	0.05	0.09	0.556	1.05	0.88	1.26
Other headache syndromes	GPNMB	Wald ratio	1	0.04	0.07	0.556	1.04	0.91	1.18
Cardiac dysrhythmias	GPNMB	Wald ratio	1	-0.02	0.04	0.558	0.98	0.91	1.05
Carditis	GPNMB	Wald ratio	1	-0.07	0.13	0.561	0.93	0.72	1.19
Derangement of joint, non-traumatic	GPNMB	Wald ratio	1	-0.06	0.11	0.562	0.94	0.76	1.16

Hyperparathyroidism	GPNMB	Wald ratio	1	-0.12	0.20	0.562	0.89	0.60	1.32
Diabetes mellitus	GPNMB	Wald ratio	1	0.02	0.04	0.567	1.03	0.94	1.12
Foreign body injury	GPNMB	Wald ratio	1	-0.09	0.16	0.571	0.91	0.66	1.26
Non-Hodgkins lymphoma	GPNMB	Wald ratio	1	-0.08	0.13	0.577	0.93	0.71	1.21
Cataract	GPNMB	Wald ratio	1	-0.02	0.04	0.577	0.98	0.90	1.06
Ptosis of eyelid	GPNMB	Wald ratio	1	-0.07	0.13	0.579	0.93	0.72	1.21
Chronic periodontitis	GPNMB	Wald ratio	1	-0.13	0.24	0.582	0.88	0.55	1.40
Secondary malignant neoplasm	GPNMB	Wald ratio	1	0.03	0.06	0.582	1.03	0.92	1.16
Bronchiectasis	GPNMB	Wald ratio	1	-0.07	0.13	0.585	0.93	0.72	1.20
Other disorders of circulatory system	GPNMB	Wald ratio	1	0.03	0.05	0.585	1.03	0.94	1.12
Early or threatened labor; hemorrhage in early pregnancy	GPNMB	Wald ratio	1	0.06	0.12	0.587	1.07	0.85	1.34
Symptoms and disorders of the joints	GPNMB	Wald ratio	1	-0.05	0.09	0.588	0.95	0.79	1.14
Pneumonitis due to inhalation of food or vomitus	GPNMB	Wald ratio	1	0.13	0.23	0.590	1.14	0.72	1.80
Genital prolapse	GPNMB	Wald ratio	1	-0.03	0.05	0.593	0.97	0.87	1.08
Strabismus and other disorders of binocular eye movements	GPNMB	Wald ratio	1	-0.08	0.15	0.595	0.92	0.69	1.24
Other upper respiratory disease	GPNMB	Wald ratio	1	-0.05	0.09	0.595	0.95	0.80	1.14
Acute pharyngitis	GPNMB	Wald ratio	1	-0.10	0.19	0.597	0.90	0.62	1.32
Polyarteritis nodosa and allied conditions	GPNMB	Wald ratio	1	-0.10	0.20	0.600	0.90	0.61	1.33
Symptoms involving skin and other integumentary tissue	GPNMB	Wald ratio	1	-0.07	0.13	0.601	0.93	0.72	1.21
Ulceration of intestine	GPNMB	Wald ratio	1	0.11	0.22	0.605	1.12	0.73	1.72
Other local infections of skin and subcutaneous tissue	GPNMB	Wald ratio	1	-0.04	0.08	0.606	0.96	0.81	1.13
Inflammation of eyelids	GPNMB	Wald ratio	1	0.06	0.12	0.606	1.06	0.85	1.33
Premature beats	GPNMB	Wald ratio	1	0.13	0.25	0.606	1.13	0.70	1.83
Inflammatory and toxic neuropathy	GPNMB	Wald ratio	1	-0.08	0.15	0.608	0.92	0.68	1.25
Stricture/obstruction of ureter	GPNMB	Wald ratio	1	-0.10	0.19	0.610	0.91	0.63	1.31
Other dyschromia	GPNMB	Wald ratio	1	-0.10	0.20	0.610	0.90	0.61	1.34

Psoriatic arthropathy	GPNMB	Wald ratio	1	0.11	0.21	0.612	1.11	0.73	1.69
Other acquired deformities of limbs	GPNMB	Wald ratio	1	0.07	0.14	0.612	1.07	0.82	1.41
Cardiac arrest	GPNMB	Wald ratio	1	0.09	0.19	0.613	1.10	0.76	1.58
Amblyopia	GPNMB	Wald ratio	1	-0.12	0.25	0.614	0.88	0.55	1.43
Appendiceal conditions	GPNMB	Wald ratio	1	0.05	0.10	0.614	1.05	0.87	1.27
Abnormal movement	GPNMB	Wald ratio	1	-0.05	0.11	0.616	0.95	0.76	1.17
Hallux valgus (Bunion)	GPNMB	Wald ratio	1	-0.04	0.07	0.618	0.96	0.84	1.11
Cardiomegaly	GPNMB	Wald ratio	1	0.06	0.11	0.618	1.06	0.85	1.32
Hypovolemia	GPNMB	Wald ratio	1	-0.05	0.11	0.618	0.95	0.77	1.17
Diseases of nail, NOS	GPNMB	Wald ratio	1	0.08	0.16	0.619	1.08	0.79	1.48
Other conditions of brain	GPNMB	Wald ratio	1	-0.07	0.15	0.620	0.93	0.69	1.25
Hypothyroidism	GPNMB	Wald ratio	1	0.02	0.05	0.620	1.02	0.93	1.13
Disorders of calcium/phosphorus metabolism	GPNMB	Wald ratio	1	0.08	0.16	0.627	1.08	0.79	1.49
Pulmonary heart disease	GPNMB	Wald ratio	1	0.04	0.09	0.628	1.04	0.88	1.24
Streptococcus infection	GPNMB	Wald ratio	1	-0.07	0.14	0.629	0.93	0.71	1.23
Protein-calorie malnutrition	GPNMB	Wald ratio	1	-0.08	0.17	0.630	0.92	0.65	1.29
Injury, NOS	GPNMB	Wald ratio	1	-0.03	0.06	0.630	0.97	0.86	1.10
Cancer of kidney and renal pelvis	GPNMB	Wald ratio	1	-0.08	0.18	0.631	0.92	0.65	1.30
Unstable angina (intermediate coronary syndrome)	GPNMB	Wald ratio	1	-0.04	0.08	0.636	0.96	0.82	1.13
Manlignant and unknown neoplasms of brain and nervous system	GPNMB	Wald ratio	1	0.11	0.22	0.637	1.11	0.72	1.72
Acute pancreatitis	GPNMB	Wald ratio	1	0.06	0.13	0.637	1.06	0.83	1.37
Liver abscess and sequelae of chronic liver disease	GPNMB	Wald ratio	1	0.09	0.18	0.637	1.09	0.76	1.57
Symptoms affecting skin	GPNMB	Wald ratio	1	0.04	0.08	0.638	1.04	0.89	1.20
Flatulence	GPNMB	Wald ratio	1	0.06	0.14	0.640	1.07	0.81	1.40
Viral hepatitis	GPNMB	Wald ratio	1	0.08	0.16	0.641	1.08	0.78	1.49

Malignant neoplasm of other and ill-defined sites within the digestive organs and peritoneum	GPNMB	Wald ratio	1	-0.04	0.08	0.641	0.96	0.83	1.12
Type 2 diabetes with ophthalmic manifestations	GPNMB	Wald ratio	1	0.07	0.16	0.643	1.08	0.79	1.47
Cancer of other lymphoid, histiocytic tissue	GPNMB	Wald ratio	1	0.06	0.12	0.643	1.06	0.84	1.34
Ectropion or entropion	GPNMB	Wald ratio	1	0.08	0.17	0.645	1.08	0.77	1.52
Irregular menstrual cycle	GPNMB	Wald ratio	1	-0.06	0.13	0.646	0.94	0.73	1.21
Abnormal glucose	GPNMB	Wald ratio	1	0.10	0.22	0.646	1.10	0.72	1.69
Bronchitis	GPNMB	Wald ratio	1	0.10	0.23	0.646	1.11	0.71	1.73
Other anemias	GPNMB	Wald ratio	1	-0.02	0.05	0.647	0.98	0.88	1.08
Other hypertrophic and atrophic conditions of skin	GPNMB	Wald ratio	1	0.04	0.08	0.648	1.04	0.88	1.22
Other disorders of male genital organs	GPNMB	Wald ratio	1	-0.05	0.11	0.649	0.95	0.77	1.18
Acute tonsillitis	GPNMB	Wald ratio	1	0.10	0.23	0.649	1.11	0.71	1.74
Hypertension	GPNMB	Wald ratio	1	0.01	0.03	0.649	1.01	0.96	1.06
Pancreatic cancer	GPNMB	Wald ratio	1	0.11	0.23	0.653	1.11	0.70	1.76
Carbuncle and furuncle	GPNMB	Wald ratio	1	0.05	0.12	0.654	1.05	0.84	1.33
Circulatory disease NEC	GPNMB	Wald ratio	1	0.02	0.05	0.654	1.02	0.93	1.12
Benign neoplasm of other parts of digestive system	GPNMB	Wald ratio	1	-0.04	0.08	0.654	0.97	0.83	1.13
Crystal arthropathies	GPNMB	Wald ratio	1	0.10	0.23	0.654	1.11	0.71	1.74
Colorectal cancer	GPNMB	Wald ratio	1	-0.04	0.09	0.655	0.96	0.81	1.14
Complications of cardiac/vascular device, implant, and graft	GPNMB	Wald ratio	1	-0.06	0.13	0.655	0.94	0.72	1.23
Dislocation	GPNMB	Wald ratio	1	0.06	0.12	0.655	1.06	0.83	1.35
Disorders of sweat glands	GPNMB	Wald ratio	1	-0.09	0.20	0.657	0.92	0.62	1.35
Acute appendicitis	GPNMB	Wald ratio	1	0.05	0.11	0.658	1.05	0.84	1.31
Essential hypertension	GPNMB	Wald ratio	1	0.01	0.03	0.662	1.01	0.96	1.06
Adverse effects of sedatives or other central nervous system depressants and anesthetics	GPNMB	Wald ratio	1	0.10	0.23	0.662	1.11	0.70	1.76

Hemoptysis	GPNMB	Wald ratio	1	-0.05	0.13	0.662	0.95	0.74	1.21
Cyst or abscess of Bartholin's gland	GPNMB	Wald ratio	1	0.09	0.21	0.663	1.09	0.73	1.64
Colon cancer	GPNMB	Wald ratio	1	-0.05	0.10	0.663	0.96	0.78	1.17
Benign neoplasm of brain and other parts of nervous system	GPNMB	Wald ratio	1	0.09	0.20	0.665	1.09	0.74	1.61
Fracture of humerus	GPNMB	Wald ratio	1	-0.06	0.14	0.665	0.94	0.72	1.23
Noninflammatory disorders of vulva and perineum	GPNMB	Wald ratio	1	-0.07	0.16	0.665	0.94	0.69	1.27
Bursitis	GPNMB	Wald ratio	1	-0.08	0.19	0.668	0.92	0.63	1.35
Displacement of intervertebral disc	GPNMB	Wald ratio	1	-0.11	0.25	0.670	0.90	0.55	1.47
Other diseases of respiratory system, not elsewhere classified	GPNMB	Wald ratio	1	-0.03	0.06	0.672	0.98	0.87	1.09
Malposition and malpresentation of fetus or obstruction	GPNMB	Wald ratio	1	0.06	0.14	0.672	1.06	0.80	1.40
Aphasia/speech disturbance	GPNMB	Wald ratio	1	-0.06	0.15	0.673	0.94	0.71	1.25
Intestinal malabsorption (non-celiac)	GPNMB	Wald ratio	1	-0.05	0.13	0.673	0.95	0.74	1.21
Umbilical cord complications during labor and delivery	GPNMB	Wald ratio	1	-0.11	0.25	0.673	0.90	0.55	1.47
Dysmenorrhea	GPNMB	Wald ratio	1	0.06	0.14	0.674	1.06	0.81	1.38
Benign neoplasm of brain, cranial nerves, meninges	GPNMB	Wald ratio	1	0.09	0.20	0.675	1.09	0.73	1.63
Other unspecified back disorders	GPNMB	Wald ratio	1	-0.06	0.14	0.677	0.94	0.71	1.24
Esophageal bleeding (varices/hemorrhage)	GPNMB	Wald ratio	1	-0.06	0.14	0.677	0.94	0.72	1.24
Schizophrenia	GPNMB	Wald ratio	1	-0.10	0.24	0.680	0.91	0.57	1.45
Noninflammatory female genital disorders	GPNMB	Wald ratio	1	0.02	0.06	0.681	1.03	0.91	1.15
Nonspecific abnormal findings on radiological and other examination of musculoskeletal system	GPNMB	Wald ratio	1	-0.09	0.21	0.683	0.92	0.60	1.40
Hypothyroidism NOS	GPNMB	Wald ratio	1	0.02	0.05	0.685	1.02	0.92	1.13
Anaphylactic shock NOS	GPNMB	Wald ratio	1	-0.10	0.24	0.686	0.91	0.56	1.46
Diseases of pancreas	GPNMB	Wald ratio	1	0.04	0.11	0.687	1.05	0.84	1.30
Cervical cancer and dysplasia	GPNMB	Wald ratio	1	0.04	0.09	0.687	1.04	0.86	1.25
Urinary calculus	GPNMB	Wald ratio	1	-0.03	0.07	0.691	0.97	0.85	1.12

Stricture and stenosis of esophagus	GPNMB	Wald ratio	1	-0.05	0.13		0.693	0.95	0.73	1.23
Fracture of hand or wrist	GPNMB	Wald ratio	1	-0.04	0.10		0.694	0.96	0.79	1.17
Other mental disorder	GPNMB	Wald ratio	1	0.01	0.04		0.695	1.01	0.94	1.09
GERD	GPNMB	Wald ratio	1	0.02	0.05		0.695	1.02	0.93	1.12
Voice disturbance	GPNMB	Wald ratio	1	0.07	0.17		0.696	1.07	0.76	1.50
Cardiac arrest and ventricular fibrillation	GPNMB	Wald ratio	1	0.07	0.17		0.696	1.07	0.77	1.49
Rash and other nonspecific skin eruption	GPNMB	Wald ratio	1	-0.05	0.12		0.697	0.95	0.75	1.21
Psoriasis	GPNMB	Wald ratio	1	0.05	0.12		0.698	1.05	0.83	1.33
Spondylosis and allied disorders	GPNMB	Wald ratio	1	0.03	0.07		0.699	1.03	0.90	1.17
Cancer of esophagus	GPNMB	Wald ratio	1	-0.08	0.21		0.700	0.92	0.61	1.40
Symptoms involving digestive system	GPNMB	Wald ratio	1	0.02	0.05		0.703	1.02	0.93	1.11
Viral Enteritis	GPNMB	Wald ratio	1	-0.07	0.19		0.703	0.93	0.64	1.36
Iron deficiency anemias, unspecified or not due to blood loss	GPNMB	Wald ratio	1	0.03	0.07		0.703	1.03	0.90	1.17
Disorders of mineral metabolism	GPNMB	Wald ratio	1	-0.05	0.12		0.703	0.95	0.75	1.22
Subjective visual disturbances	GPNMB	Wald ratio	1	0.09	0.23		0.708	1.09	0.70	1.71
Benign neoplasm of skin	GPNMB	Wald ratio	1	-0.02	0.07		0.708	0.98	0.86	1.11
Occlusion and stenosis of precerebral arteries	GPNMB	Wald ratio	1	0.06	0.17		0.709	1.06	0.77	1.47
Effects radiation NOS	GPNMB	Wald ratio	1	-0.04	0.10		0.710	0.96	0.79	1.18
Malignant neoplasm of ovary	GPNMB	Wald ratio	1	0.05	0.12		0.710	1.05	0.82	1.34
Cystitis and urethritis	GPNMB	Wald ratio	1	-0.04	0.10		0.711	0.96	0.79	1.18
Secondary malignancy of lymph nodes	GPNMB	Wald ratio	1	0.03	0.08		0.713	1.03	0.88	1.20
Other benign neoplasm of connective and other soft tissue	GPNMB	Wald ratio	1	-0.06	0.17		0.713	0.94	0.67	1.31
Secondary malignancy of bone	GPNMB	Wald ratio	1	-0.04	0.12		0.714	0.96	0.75	1.22
Decubitus ulcer	GPNMB	Wald ratio	1	0.07	0.19		0.714	1.07	0.74	1.55
Endometrial hyperplasia	GPNMB	Wald ratio	1	0.06	0.17		0.715	1.06	0.76	1.48
Calculus of kidney	GPNMB	Wald ratio	1	-0.04	0.10		0.718	0.96	0.79	1.18

Urinary tract infection	GPNMB	Wald ratio	1	0.02	0.05	0.719	1.02	0.92	1.13
Gangrene	GPNMB	Wald ratio	1	-0.09	0.24	0.719	0.92	0.57	1.47
Congenital anomalies of urinary system	GPNMB	Wald ratio	1	-0.07	0.19	0.720	0.93	0.64	1.37
Reflux esophagitis	GPNMB	Wald ratio	1	-0.02	0.06	0.720	0.98	0.88	1.10
Bipolar	GPNMB	Wald ratio	1	0.06	0.18	0.721	1.06	0.76	1.50
Secondary hypothyroidism	GPNMB	Wald ratio	1	0.06	0.17	0.722	1.06	0.76	1.49
Periodontitis (acute or chronic)	GPNMB	Wald ratio	1	-0.06	0.16	0.726	0.94	0.69	1.30
Malignant neoplasm of ovary and other uterine adnexa	GPNMB	Wald ratio	1	0.04	0.12	0.726	1.04	0.82	1.33
Hemorrhage of gastrointestinal tract	GPNMB	Wald ratio	1	-0.03	0.08	0.727	0.97	0.83	1.14
Acquired absence of breast	GPNMB	Wald ratio	1	0.04	0.12	0.727	1.04	0.83	1.31
Paralytic ileus	GPNMB	Wald ratio	1	0.09	0.25	0.729	1.09	0.67	1.77
Ulcer of esophagus	GPNMB	Wald ratio	1	0.03	0.08	0.729	1.03	0.88	1.20
Malaise and fatigue	GPNMB	Wald ratio	1	-0.03	0.10	0.730	0.97	0.80	1.17
Osteomyelitis, periostitis, and other infections involving bone	GPNMB	Wald ratio	1	-0.08	0.23	0.730	0.92	0.59	1.45
Disorders of other cranial nerves	GPNMB	Wald ratio	1	0.05	0.15	0.732	1.05	0.78	1.42
Glaucoma	GPNMB	Wald ratio	1	0.03	0.09	0.732	1.03	0.87	1.22
Abnormal heart sounds	GPNMB	Wald ratio	1	-0.06	0.18	0.732	0.94	0.67	1.33
Contracture of palmar fascia [Dupuytren's disease]	GPNMB	Wald ratio	1	-0.03	0.10	0.732	0.97	0.80	1.17
Ischemic Heart Disease	GPNMB	Wald ratio	1	-0.01	0.04	0.732	0.99	0.92	1.06
Diseases of spleen	GPNMB	Wald ratio	1	-0.09	0.25	0.733	0.92	0.56	1.50
Renal colic	GPNMB	Wald ratio	1	0.04	0.13	0.733	1.04	0.82	1.34
Malignant neoplasm of kidney, except pelvis	GPNMB	Wald ratio	1	-0.06	0.18	0.734	0.94	0.66	1.34
Effects of other external causes	GPNMB	Wald ratio	1	0.03	0.09	0.735	1.03	0.87	1.22
Agorophobia, social phobia, and panic disorder	GPNMB	Wald ratio	1	-0.07	0.21	0.737	0.93	0.61	1.42
Aortic valve disease	GPNMB	Wald ratio	1	-0.05	0.16	0.737	0.95	0.69	1.30
Nevus, non-neoplastic	GPNMB	Wald ratio	1	0.08	0.23	0.738	1.08	0.68	1.71

Cough	GPNMB	Wald ratio	1	0.04	0.11	0.740	1.04	0.84	1.28
Skin cancer	GPNMB	Wald ratio	1	0.02	0.05	0.742	1.02	0.92	1.12
Iron deficiency anemias	GPNMB	Wald ratio	1	0.02	0.07	0.744	1.02	0.90	1.16
Synovitis and tenosynovitis	GPNMB	Wald ratio	1	-0.04	0.11	0.746	0.97	0.78	1.20
Dyschromia and Vitiligo	GPNMB	Wald ratio	1	-0.06	0.18	0.748	0.94	0.66	1.35
Hemorrhage of rectum and anus	GPNMB	Wald ratio	1	0.02	0.05	0.750	1.02	0.92	1.12
Nonspecific abnormal findings in stool contents	GPNMB	Wald ratio	1	-0.04	0.14	0.750	0.96	0.73	1.26
Enthesopathy	GPNMB	Wald ratio	1	0.02	0.06	0.751	1.02	0.91	1.14
Personal history of diseases of digestive system	GPNMB	Wald ratio	1	0.02	0.05	0.752	1.02	0.92	1.11
Abnormality of gait	GPNMB	Wald ratio	1	-0.04	0.14	0.752	0.96	0.72	1.26
Mucous polyp of cervix	GPNMB	Wald ratio	1	-0.03	0.10	0.756	0.97	0.80	1.17
Vascular hamartomas and non-neoplastic nevi	GPNMB	Wald ratio	1	0.07	0.22	0.757	1.07	0.69	1.66
Phobia	GPNMB	Wald ratio	1	0.08	0.25	0.758	1.08	0.66	1.77
Seborrheic keratosis	GPNMB	Wald ratio	1	-0.03	0.10	0.764	0.97	0.79	1.19
Symptoms involving nervous and musculoskeletal systems	GPNMB	Wald ratio	1	0.04	0.13	0.765	1.04	0.80	1.35
Diseases of the larynx and vocal cords	GPNMB	Wald ratio	1	-0.03	0.11	0.766	0.97	0.78	1.20
Cholelithiasis and cholecystitis	GPNMB	Wald ratio	1	-0.01	0.05	0.767	0.99	0.90	1.08
Internal derangement of knee	GPNMB	Wald ratio	1	-0.01	0.05	0.768	0.99	0.90	1.08
Ovarian cyst	GPNMB	Wald ratio	1	0.02	0.08	0.769	1.02	0.87	1.21
Disorders of uterus, NEC	GPNMB	Wald ratio	1	-0.03	0.10	0.771	0.97	0.80	1.18
Disorders of lipoid metabolism	GPNMB	Wald ratio	1	0.01	0.03	0.775	1.01	0.95	1.08
Infertility, female	GPNMB	Wald ratio	1	-0.05	0.16	0.775	0.96	0.70	1.30
Sarcoidosis	GPNMB	Wald ratio	1	-0.07	0.24	0.778	0.93	0.58	1.50
Intestinal infection due to C. difficile	GPNMB	Wald ratio	1	-0.06	0.22	0.779	0.94	0.61	1.45
Tobacco use disorder	GPNMB	Wald ratio	1	0.01	0.04	0.780	1.01	0.93	1.10
Pyelonephritis	GPNMB	Wald ratio	1	0.04	0.16	0.780	1.04	0.77	1.42

Other disorders of cervical region	GPNMB	Wald ratio	1	0.07	0.25	0.781	1.07	0.66	1.75
Cancer of other female genital organs	GPNMB	Wald ratio	1	0.03	0.12	0.782	1.03	0.82	1.30
Cerebral artery occlusion, with cerebral infarction	GPNMB	Wald ratio	1	-0.04	0.15	0.784	0.96	0.72	1.28
Hyperlipidemia	GPNMB	Wald ratio	1	0.01	0.03	0.787	1.01	0.95	1.08
Acute periodontitis	GPNMB	Wald ratio	1	-0.06	0.22	0.787	0.94	0.61	1.45
Acute gastritis	GPNMB	Wald ratio	1	-0.04	0.17	0.792	0.96	0.69	1.32
Asthma	GPNMB	Wald ratio	1	0.01	0.04	0.793	1.01	0.94	1.09
Poisoning by anticonvulsants and anti-Parkinsonism drugs	GPNMB	Wald ratio	1	0.07	0.25	0.794	1.07	0.65	1.75
Secondary malignancy of brain/spine	GPNMB	Wald ratio	1	-0.05	0.20	0.794	0.95	0.64	1.41
Inguinal hernia	GPNMB	Wald ratio	1	-0.01	0.05	0.794	0.99	0.90	1.08
Other disorders of metabolism	GPNMB	Wald ratio	1	-0.04	0.15	0.797	0.96	0.72	1.29
Occlusion of cerebral arteries	GPNMB	Wald ratio	1	-0.02	0.09	0.797	0.98	0.82	1.16
Late effects of cerebrovascular disease	GPNMB	Wald ratio	1	-0.04	0.16	0.799	0.96	0.70	1.31
Diseases and other conditions of the tongue	GPNMB	Wald ratio	1	0.04	0.16	0.799	1.04	0.76	1.43
Retinal detachment with retinal defect	GPNMB	Wald ratio	1	-0.04	0.15	0.800	0.96	0.71	1.30
Fracture of neck of femur	GPNMB	Wald ratio	1	0.03	0.13	0.800	1.03	0.80	1.35
Other acute and subacute forms of ischemic heart disease	GPNMB	Wald ratio	1	-0.04	0.17	0.801	0.96	0.69	1.33
Acute and chronic tonsillitis	GPNMB	Wald ratio	1	0.03	0.13	0.802	1.03	0.80	1.32
Left bundle branch block	GPNMB	Wald ratio	1	-0.03	0.14	0.803	0.97	0.74	1.26
Hyperpotassemia	GPNMB	Wald ratio	1	-0.05	0.18	0.803	0.96	0.67	1.37
First degree AV block	GPNMB	Wald ratio	1	-0.04	0.18	0.805	0.96	0.67	1.36
Jaundice (not of newborn)	GPNMB	Wald ratio	1	0.05	0.19	0.809	1.05	0.72	1.52
Large cell lymphoma	GPNMB	Wald ratio	1	0.06	0.24	0.809	1.06	0.67	1.69
Other symptoms involving abdomen and pelvis	GPNMB	Wald ratio	1	-0.02	0.10	0.809	0.98	0.80	1.19
Spondylosis without myelopathy	GPNMB	Wald ratio	1	0.02	0.08	0.809	1.02	0.87	1.20
Noninfectious disorders of lymphatic channels	GPNMB	Wald ratio	1	-0.05	0.21	0.810	0.95	0.62	1.45

Menopausal and postmenopausal disorders	GPNMB	Wald ratio	1	0.01	0.06	0.810	1.01	0.91	1.13
Sleep apnea	GPNMB	Wald ratio	1	0.02	0.09	0.812	1.02	0.86	1.21
Electrolyte imbalance	GPNMB	Wald ratio	1	0.02	0.09	0.813	1.02	0.86	1.22
Duodenitis	GPNMB	Wald ratio	1	-0.02	0.07	0.813	0.98	0.86	1.12
Secondary malignancy of respiratory organs	GPNMB	Wald ratio	1	0.03	0.12	0.816	1.03	0.81	1.31
Allergic rhinitis	GPNMB	Wald ratio	1	-0.04	0.17	0.818	0.96	0.68	1.35
Fracture of unspecified bones	GPNMB	Wald ratio	1	-0.04	0.16	0.820	0.96	0.71	1.31
Normal delivery	GPNMB	Wald ratio	1	-0.03	0.13	0.821	0.97	0.75	1.26
Emphysema	GPNMB	Wald ratio	1	0.03	0.14	0.823	1.03	0.79	1.35
Hemorrhage or hematoma complicating a procedure	GPNMB	Wald ratio	1	0.02	0.08	0.824	1.02	0.87	1.19
Missed abortion/Hydatidiform mole	GPNMB	Wald ratio	1	0.04	0.16	0.825	1.04	0.75	1.43
Chondrocalcinosis	GPNMB	Wald ratio	1	0.05	0.24	0.828	1.05	0.66	1.69
Hemorrhage from gastrointestinal ulcer	GPNMB	Wald ratio	1	0.05	0.23	0.829	1.05	0.67	1.65
Senile cataract	GPNMB	Wald ratio	1	0.01	0.06	0.830	1.01	0.89	1.15
Disorders of refraction and accommodation; blindness and low vision	GPNMB	Wald ratio	1	-0.02	0.12	0.832	0.98	0.78	1.22
Hemangioma and lymphangioma, any site	GPNMB	Wald ratio	1	-0.03	0.14	0.834	0.97	0.73	1.28
Other disorders of middle ear and mastoid	GPNMB	Wald ratio	1	-0.04	0.20	0.835	0.96	0.65	1.41
Paroxysmal supraventricular tachycardia	GPNMB	Wald ratio	1	-0.02	0.12	0.835	0.98	0.77	1.23
Lipoma	GPNMB	Wald ratio	1	0.01	0.07	0.836	1.02	0.88	1.17
Other disorders of bone and cartilage	GPNMB	Wald ratio	1	-0.02	0.09	0.837	0.98	0.82	1.18
Other aneurysm	GPNMB	Wald ratio	1	0.03	0.13	0.838	1.03	0.79	1.34
Pulmonary collapse; interstitial and compensatory emphysema	GPNMB	Wald ratio	1	0.03	0.13	0.839	1.03	0.80	1.32
Thyrotoxicosis with or without goiter	GPNMB	Wald ratio	1	0.03	0.13	0.841	1.03	0.79	1.33
Vaginal enterocele, congenital or acquired	GPNMB	Wald ratio	1	-0.04	0.22	0.845	0.96	0.63	1.47
Hypotension NOS	GPNMB	Wald ratio	1	0.02	0.10	0.847	1.02	0.84	1.23
Empyema and pneumothorax	GPNMB	Wald ratio	1	-0.03	0.17	0.847	0.97	0.70	1.34

Allergy/adverse effect of penicillin	GPNMB	Wald ratio	1	0.01	0.05	0.849	1.01	0.92	1.10
Ulcerative colitis (chronic)	GPNMB	Wald ratio	1	0.05	0.25	0.850	1.05	0.65	1.69
Cellulitis and abscess of fingers/toes	GPNMB	Wald ratio	1	-0.04	0.24	0.851	0.96	0.60	1.53
Nontoxic multinodular goiter	GPNMB	Wald ratio	1	-0.04	0.22	0.853	0.96	0.63	1.47
Kyphoscoliosis and scoliosis	GPNMB	Wald ratio	1	0.03	0.17	0.855	1.03	0.73	1.45
Myeloproliferative disease	GPNMB	Wald ratio	1	0.03	0.18	0.855	1.03	0.73	1.47
Gout and other crystal arthropathies	GPNMB	Wald ratio	1	0.02	0.09	0.856	1.02	0.85	1.22
Hemorrhage in early pregnancy	GPNMB	Wald ratio	1	0.03	0.18	0.857	1.03	0.73	1.46
Celiac disease	GPNMB	Wald ratio	1	-0.02	0.13	0.858	0.98	0.75	1.27
Prolapse of vaginal walls	GPNMB	Wald ratio	1	0.01	0.07	0.859	1.01	0.89	1.16
Irritable Bowel Syndrome	GPNMB	Wald ratio	1	-0.01	0.08	0.859	0.99	0.85	1.15
Calculus of lower urinary tract	GPNMB	Wald ratio	1	-0.04	0.20	0.862	0.97	0.65	1.44
Disorders of adrenal glands	GPNMB	Wald ratio	1	0.04	0.22	0.863	1.04	0.67	1.61
Other abnormality of urination	GPNMB	Wald ratio	1	-0.02	0.13	0.865	0.98	0.76	1.26
Calculus of bile duct	GPNMB	Wald ratio	1	0.02	0.11	0.865	1.02	0.82	1.27
Cellulitis and abscess of face/neck	GPNMB	Wald ratio	1	-0.04	0.25	0.865	0.96	0.59	1.56
Superficial injury without mention of infection	GPNMB	Wald ratio	1	0.01	0.09	0.866	1.01	0.86	1.20
Psoriasis vulgaris	GPNMB	Wald ratio	1	-0.02	0.14	0.870	0.98	0.74	1.28
Hyperhidrosis	GPNMB	Wald ratio	1	-0.04	0.23	0.870	0.96	0.61	1.52
Fracture of pelvis	GPNMB	Wald ratio	1	-0.03	0.21	0.871	0.97	0.64	1.46
Hypertensive heart and/or renal disease	GPNMB	Wald ratio	1	0.02	0.14	0.871	1.02	0.78	1.34
Fracture of vertebral column without mention of spinal cord injury	GPNMB	Wald ratio	1	-0.02	0.15	0.872	0.98	0.72	1.32
Open wounds of head; neck; and trunk	GPNMB	Wald ratio	1	0.01	0.09	0.873	1.01	0.85	1.22
Malunion and nonunion of fracture	GPNMB	Wald ratio	1	-0.03	0.16	0.874	0.97	0.71	1.33
Complications of surgical and medical procedures	GPNMB	Wald ratio	1	-0.01	0.06	0.874	0.99	0.88	1.11
Chronic pulmonary heart disease	GPNMB	Wald ratio	1	-0.04	0.23	0.875	0.96	0.61	1.53

Otitis externa	GPNMB	Wald ratio	1	-0.04	0.25	0.877	0.96	0.59	1.57
Cystic mastopathy	GPNMB	Wald ratio	1	0.03	0.19	0.879	1.03	0.71	1.49
Malignant neoplasm of rectum, rectosigmoid junction, and anus	GPNMB	Wald ratio	1	0.02	0.13	0.880	1.02	0.80	1.30
Cholesteatoma	GPNMB	Wald ratio	1	0.03	0.23	0.881	1.04	0.66	1.63
Psoriasis and related disorders	GPNMB	Wald ratio	1	0.02	0.12	0.884	1.02	0.81	1.29
Urinary incontinence	GPNMB	Wald ratio	1	-0.01	0.06	0.886	0.99	0.88	1.12
Thrombocytopenia	GPNMB	Wald ratio	1	-0.02	0.14	0.888	0.98	0.74	1.30
E. coli	GPNMB	Wald ratio	1	-0.01	0.11	0.891	0.99	0.80	1.22
Hypotension	GPNMB	Wald ratio	1	-0.01	0.07	0.894	0.99	0.86	1.15
Other dyspnea	GPNMB	Wald ratio	1	0.03	0.19	0.894	1.03	0.71	1.48
Early onset of delivery	GPNMB	Wald ratio	1	-0.03	0.23	0.896	0.97	0.61	1.53
Other symptoms/disorders or the urinary system	GPNMB	Wald ratio	1	0.00	0.04	0.900	1.00	0.93	1.08
Other diseases of lung	GPNMB	Wald ratio	1	0.03	0.20	0.902	1.03	0.69	1.53
Peptic ulcer (excl. esophageal)	GPNMB	Wald ratio	1	-0.01	0.07	0.902	0.99	0.87	1.13
Disease of tricuspid valve	GPNMB	Wald ratio	1	-0.02	0.17	0.904	0.98	0.70	1.38
Perforation of tympanic membrane	GPNMB	Wald ratio	1	-0.02	0.18	0.904	0.98	0.69	1.38
Blindness and low vision	GPNMB	Wald ratio	1	0.02	0.21	0.907	1.02	0.68	1.55
Hypertensive chronic kidney disease	GPNMB	Wald ratio	1	-0.02	0.15	0.907	0.98	0.74	1.31
Chronic bronchitis	GPNMB	Wald ratio	1	-0.01	0.11	0.908	0.99	0.80	1.22
Varicose veins of lower extremity, symptomtic	GPNMB	Wald ratio	1	0.03	0.23	0.909	1.03	0.66	1.60
Disorders of external ear	GPNMB	Wald ratio	1	0.02	0.15	0.912	1.02	0.76	1.36
Cellulitis and abscess of trunk	GPNMB	Wald ratio	1	0.03	0.23	0.912	1.03	0.65	1.62
Other disorders of synovium, tendon, and bursa	GPNMB	Wald ratio	1	-0.01	0.07	0.913	0.99	0.87	1.13
Neuralgia, neuritis, and radiculitis NOS	GPNMB	Wald ratio	1	-0.02	0.17	0.914	0.98	0.71	1.36
Chronic fatigue syndrome	GPNMB	Wald ratio	1	0.02	0.23	0.915	1.03	0.65	1.62
Cystitis	GPNMB	Wald ratio	1	-0.01	0.11	0.915	0.99	0.80	1.22

Facial nerve disorders [CN7]	GPNMB	Wald ratio	1	-0.02	0.19	0.916	0.98	0.67	1.43
Ganglion and cyst of synovium, tendon, and bursa	GPNMB	Wald ratio	1	0.01	0.10	0.916	1.01	0.83	1.23
Heart failure NOS	GPNMB	Wald ratio	1	-0.01	0.09	0.916	0.99	0.83	1.18
Peritoneal adhesions (postoperative) (postinfection)	GPNMB	Wald ratio	1	-0.01	0.10	0.918	0.99	0.81	1.21
Ventral hernia	GPNMB	Wald ratio	1	-0.01	0.10	0.918	0.99	0.82	1.20
Melanomas of skin, dx or hx	GPNMB	Wald ratio	1	-0.01	0.11	0.918	0.99	0.80	1.23
Melanomas of skin	GPNMB	Wald ratio	1	-0.01	0.11	0.918	0.99	0.80	1.23
Anal and rectal conditions	GPNMB	Wald ratio	1	0.00	0.05	0.918	1.00	0.92	1.10
Rheumatism, unspecified and fibrositis	GPNMB	Wald ratio	1	0.02	0.18	0.919	1.02	0.72	1.45
Diseases of the salivary glands	GPNMB	Wald ratio	1	-0.02	0.21	0.920	0.98	0.64	1.49
Other ill-defined and unknown causes of morbidity and mortality	GPNMB	Wald ratio	1	0.00	0.05	0.920	1.00	0.92	1.10
Cerebrovascular disease	GPNMB	Wald ratio	1	0.01	0.06	0.921	1.01	0.89	1.14
Other non-epithelial cancer of skin	GPNMB	Wald ratio	1	0.01	0.06	0.923	1.01	0.90	1.12
Neoplasm of uncertain behavior	GPNMB	Wald ratio	1	-0.01	0.15	0.925	0.99	0.73	1.33
Abdominal hernia	GPNMB	Wald ratio	1	0.00	0.03	0.925	1.00	0.95	1.06
Retention of urine	GPNMB	Wald ratio	1	-0.01	0.07	0.926	0.99	0.87	1.14
Obstructive chronic bronchitis	GPNMB	Wald ratio	1	0.01	0.11	0.926	1.01	0.81	1.26
Chronic pharyngitis and nasopharyngitis	GPNMB	Wald ratio	1	0.02	0.18	0.929	1.02	0.71	1.46
Poisoning by antibiotics	GPNMB	Wald ratio	1	0.00	0.04	0.929	1.00	0.92	1.09
Benign neoplasm of other endocrine glands and related structures	GPNMB	Wald ratio	1	0.02	0.19	0.930	1.02	0.70	1.48
Anal and rectal polyp	GPNMB	Wald ratio	1	0.01	0.07	0.930	1.01	0.88	1.15
Type 2 diabetes	GPNMB	Wald ratio	1	0.00	0.04	0.933	1.00	0.92	1.10
Other venous embolism and thrombosis	GPNMB	Wald ratio	1	0.02	0.24	0.933	1.02	0.64	1.63
Other specified cardiac dysrhythmias	GPNMB	Wald ratio	1	-0.01	0.10	0.938	0.99	0.81	1.21
Varicose veins of lower extremity	GPNMB	Wald ratio	1	0.00	0.06	0.939	1.00	0.90	1.12
Crushing or internal injury to organs	GPNMB	Wald ratio	1	-0.01	0.18	0.940	0.99	0.69	1.40

Tinnitus	GPNMB	Wald ratio	1	0.02	0.25	0.944	1.02	0.62	1.66
Angina pectoris	GPNMB	Wald ratio	1	0.00	0.05	0.945	1.00	0.91	1.09
Degeneration of macula and posterior pole of retina	GPNMB	Wald ratio	1	-0.01	0.12	0.948	0.99	0.78	1.26
Noninflammatory disorders of ovary, fallopian tube, and broad ligament	GPNMB	Wald ratio	1	0.01	0.21	0.950	1.01	0.67	1.53
Extrapyramidal disease and abnormal movement disorders	GPNMB	Wald ratio	1	0.01	0.19	0.950	1.01	0.70	1.47
Cancer of stomach	GPNMB	Wald ratio	1	0.01	0.24	0.952	1.01	0.63	1.63
Coagulation defects	GPNMB	Wald ratio	1	-0.01	0.19	0.953	0.99	0.69	1.42
Uterine/Uterovaginal prolapse	GPNMB	Wald ratio	1	0.00	0.08	0.953	1.00	0.86	1.17
Syncope and collapse	GPNMB	Wald ratio	1	0.00	0.06	0.958	1.00	0.89	1.12
Other diseases of the teeth and supporting structures	GPNMB	Wald ratio	1	0.01	0.11	0.959	1.01	0.81	1.25
Prurigo and Lichen	GPNMB	Wald ratio	1	-0.01	0.20	0.961	0.99	0.66	1.48
Macular degeneration (senile) of retina NOS	GPNMB	Wald ratio	1	-0.01	0.12	0.962	0.99	0.78	1.26
Femoral hernia	GPNMB	Wald ratio	1	0.01	0.22	0.963	1.01	0.65	1.56
Transient cerebral ischemia	GPNMB	Wald ratio	1	-0.01	0.12	0.964	0.99	0.78	1.27
Other disorders of tympanic membrane	GPNMB	Wald ratio	1	-0.01	0.15	0.965	0.99	0.73	1.34
Disorders of parathyroid gland	GPNMB	Wald ratio	1	-0.01	0.19	0.968	0.99	0.68	1.45
Noninflammatory disorders of cervix	GPNMB	Wald ratio	1	0.00	0.11	0.970	1.00	0.81	1.24
Tachycardia NOS	GPNMB	Wald ratio	1	0.00	0.12	0.972	1.00	0.78	1.26
Hearing loss	GPNMB	Wald ratio	1	0.00	0.09	0.972	1.00	0.84	1.19
Hypertension complicating pregnancy, childbirth, and the puerperium	GPNMB	Wald ratio	1	0.01	0.17	0.972	1.01	0.71	1.42
Meniere's disease	GPNMB	Wald ratio	1	0.01	0.24	0.973	1.01	0.63	1.60
Postmenopausal bleeding	GPNMB	Wald ratio	1	0.00	0.06	0.975	1.00	0.89	1.13
Gout	GPNMB	Wald ratio	1	0.00	0.10	0.976	1.00	0.82	1.22
Curvature of spine	GPNMB	Wald ratio	1	0.00	0.17	0.976	1.00	0.71	1.39
Lipoma of skin and subcutaneous tissue	GPNMB	Wald ratio	1	0.00	0.08	0.977	1.00	0.85	1.18
Hypercholesterolemia	GPNMB	Wald ratio	1	0.00	0.03	0.977	1.00	0.94	1.07

Duodenal ulcer	GPNMB	Wald ratio	1	0.00	0.11	0.978	1.00	0.82	1.23
Open wounds of extremities	GPNMB	Wald ratio	1	0.00	0.09	0.979	1.00	0.84	1.19
Psychogenic and somatoform disorders	GPNMB	Wald ratio	1	-0.01	0.25	0.980	0.99	0.61	1.61
Varicose veins	GPNMB	Wald ratio	1	0.00	0.05	0.983	1.00	0.90	1.11
Hemorrhage during pregnancy; childbirth and postpartum	GPNMB	Wald ratio	1	0.00	0.13	0.987	1.00	0.77	1.30
Edema	GPNMB	Wald ratio	1	0.00	0.14	0.987	1.00	0.76	1.32
Other disorders of peritoneum	GPNMB	Wald ratio	1	0.00	0.10	0.987	1.00	0.82	1.21
Gastrointestinal hemorrhage	GPNMB	Wald ratio	1	0.00	0.04	0.988	1.00	0.93	1.08
Known or suspected fetal abnormality affecting management of mother	GPNMB	Wald ratio	1	0.00	0.09	0.988	1.00	0.83	1.20
Disorders of fluid, electrolyte, and acid-base balance	GPNMB	Wald ratio	1	0.00	0.07	0.989	1.00	0.88	1.14
Purpura and other hemorrhagic conditions	GPNMB	Wald ratio	1	0.00	0.13	0.990	1.00	0.77	1.30
Sleep disorders	GPNMB	Wald ratio	1	0.00	0.08	0.996	1.00	0.86	1.17
Abnormal involuntary movements	GPNMB	Wald ratio	1	0.00	0.19	0.998	1.00	0.69	1.45

Supplementary Table 9 The details of studies for QTLs and Parkinson's disease via MR.

Study	PD risk GWAS	Blood eQTL	Blood pQTL (number of proteins)	CSF pQTL (number of proteins)	Brain eQTL (number of genes)	Brain pQTL (number of proteins)	Methods
**Our study**	Nalls (2019)[1]	The eQTLGen[6]	Sun (2018)[5]	Yang (2021)[4]	The PsychENCODE[3, 10]	The ROS/MAP[2]	Two-sample MR analysis, Colocalization, Steiger filtering analysis,fine mapping, Protein Interactor, phenome-wide MR
Ge (2023)	Nalls (2019)[1]	/	Sun (2018)[5]	/	/	The ROS/MAP[2]	Two-sample MR analysis, Colocalization, drugbility and safety analysis
Storm (2021)[11]	Nalls (2014)[12] / Nalls (2019)[1]	The eQTLGen[6]	Sun (2018)[5]	/	The PsychENCODE[3, 10]	Hillary (2019)[13]	Two-sample MR analysis, Colocalization
Png (2021)[14]	Pankratz (2011)[15]	/	Png (2021)[14]	/	/	/	Two-sample MR analysis, Colocalization
Cui (2021)	The PD cohort in the UK Biobank (The details are unknown)	Only CD4 and CD8 (The details are unknown)	/	/	The brain eQTL dataset (The details are unknown)	/	Summary data-based MR
Yang (2021)[4]	Nalls (2019)[1]	/	Yang (2021)[4]	Yang (2021)[4]	/	Yang (2021)[4]	MR, Colocalization
Demis (2021)[16]	Chang (2017)[17]	/	/	/	The GTEx version 7 data and the UK Brain Expression Consortium Braineac data set contains data*	/	MR, Colocalization, TWAS, Weighted Gene Co-expression Network Analysis, Protein Interactor Networks
Baird (2021)[18]	Nalls (2014)[12]	/	/	/	The GTEx brain meta-analysis[19]	/	two-sample MR, Colocalization, Steiger filtering analysis
Nalls (2019)[1]	Nalls (2019)[1]	The eQTLGen[6]	/	/	Qi (2018)[19]	/	summary-based MR
Bandres-Ciga(2019)[20]	Bandres-Ciga(2019)[20]	Zhu (2016)[21]	/	/	Zhu (2016)[21]	/	Two-sample MR

* Data source: https://www.gtexportal.org/home/ and http://www.braineac.org/.

Supplementary Table 10 List of datasets used in this study

Data source	Dataset	Cohort/Project	Sample size
Nalls et al. (2019)[1]	PD GWAS	IPDGC	482730 (induvials)
Wingo et al. (2021)[2]	Brain pQTL	ROS/MAP	376 (induvials)
Robins et al. (2021)	Brain pQTL	Banner	152 (induvials)
Akbarian et al. (2015)[3]	Brain eQTL	PsychENCODE	1866 (induvials)
Sieberts et al (2020)	Brain eQTL	CMC, AMP-AD	1433 (induvials)
Yang et al. (2021)[4]	CSF pQTL	NA	713 (proteins)
Sun et al. (2018)[5]	Blood pQTL	NA	2994 (proteins)
Võsa et al. (2018)[6]	Blood eQTL	eQTLGen	31684 (induvials)

Supplementary Table 11 Known PD causative/risk genes

Gene name	Locus	Inheritance	Disease onset	Clinical phenotype
LRRK2	12q12	AD/AR	Late	Typical
SNCA	4q22.1	AD	Early	Typical
VPS35	16q11.2	AD	Late	Atypical
ATXN2	12q24.12	AD	Early	Typical
GCH1	14q22.2	AD	Early	Typical
DNAJC13	3q22.1	AD	Late	Typical
TMEM230	20p13-p12	AD	Late	Typical
UCHL1	4p13	AD	Early	Typical
RIC3	11p15.4	AD	Early or late	Typical
HTRA2	2q13.1	AD	Late	Typical
GIGYF2	2q37.1	AD	Early	Typical
CHCHD2	7p11.2	AD	Early or late	Typical
EIF4G1	3q27.1	AD	Late	Typical
PTRHD1	2p23.3	AR	Early	Atypical
POXDL	7q32.3	AR	Juvenile	Typical
PRKN	6q26	AR	Juvenile or early	Typical
PINK	1p36.12	AR	Juvenile or early	Typical
DJ1	1p36.23	AR	Juvenile or early	Typical
ATP13A2	1p36.13	AR	Juvenile	Atypical
PLA2G6	22q13.1	AR	Juvenile or early	Atypical
FBXO7	22q12.3	AR	Juvenile or early	Atypical

DNAJC6	1q31.3	AR	Juvenile or early	Atypical or typical
SPG11	15q21.1	AR	Juvenile	Atypical
SYNJ1	21q22.11	AR	Early	Atypical
VPS13C	15q22.2	AR	Early	Atypical
RAB38B	Xq28	XLD	Early for men, late for women	Atypical or typical
GBA	1q22	AD	Early or late	Typical

The genes were sumarrzied in Lunati A et al (2018)[7]. AD: Autosomal dominant; AR: Autosomal recessive; XLD: X-linked

Supplementary Table 12 List of PD drug and the corresponding drug targets

Drug name	Target	Gene Name	Uniprot ID	Pharmacological Action
levodopa	Dopamine D1 receptor	*DRD1*	P21728	Yes
	Dopamine D5 receptor	*DRD5*	P21918	Yes
	Dopamine D2 receptor	*DRD2*	P14416	Yes
	Dopamine D3 receptor	*DRD3*	P35462	Yes
	Dopamine D4 receptor	*DRD4*	P21917	Yes
Carbidopa	Aromatic-L-amino-acid decarboxylase	*DDC*	P20711	Yes
pramipexole	Dopamine D3 receptor	*DRD3*	P35462	Yes
	Dopamine D2 receptor	*DRD2*	P14416	Yes
	Dopamine D4 receptor	*DRD4*	P21917	Yes
	5-hydroxytryptamine receptor 1A	*HTR1A*	P08908	Unknown
	Alpha-2A adrenergic receptor	*ADRA2A*	P08913	Unknown
ropinirole	Dopamine D3 receptor	*DRD3*	P35462	Yes
	Dopamine D2 receptor	*DRD2*	P14416	Yes
	Dopamine D4 receptor	*DRD4*	P21917	Unknown
	Alpha-1A adrenergic receptor	*ADRA1A*	P35348	Unknown
	Alpha-1B adrenergic receptor	*ADRA1B*	P35368	Unknown
	Alpha-1D adrenergic receptor	*ADRA1D*	P25100	Unknown
	Alpha-2A adrenergic receptor	*ADRA2A*	P08913	Unknown
	Alpha-2B adrenergic receptor	*ADRA2B*	P18089	Unknown
	Alpha-2C adrenergic receptor	*ADRA2C*	P18825	Unknown
rotigotine	Dopamine D3 receptor	*DRD3*	P35462	Yes
	Dopamine D2 receptor	*DRD2*	P14416	Yes
	Dopamine D4 receptor	*DRD4*	P21917	Yes

	Dopamine D5 receptor	*DRD5*	P21918	Unknown
	Dopamine D1 receptor	*DRD1*	P21728	Unknown
	5-hydroxytryptamine receptor 1A	*HTR1A*	P08908	Unknown
	Alpha-2B adrenergic receptor	*ADRA2B*	P18089	Unknown
apomorphine	Dopamine D3 receptor	*DRD3*	P35462	Yes
	Dopamine D2 receptor	*DRD2*	P14416	Yes
	Dopamine D4 receptor	*DRD4*	P21917	Yes
	Dopamine D5 receptor	*DRD5*	P21918	Unknown
	Dopamine D1 receptor	*DRD1*	P21728	Unknown
	Alpha-2B adrenergic receptor	*ADRA2B*	P18089	Unknown
	Alpha-2C adrenergic receptor	*ADRA2C*	P18825	Unknown
	Alpha-2A adrenergic receptor	*ADRA2A*	P08913	Unknown
	5-hydroxytryptamine receptor 1A	*HTR1A*	P08908	Unknown
	5-hydroxytryptamine receptor 2A	*HTR2A*	P28223	Unknown
	5-hydroxytryptamine receptor 2B	*HTR2B*	P41595	Unknown
	5-hydroxytryptamine receptor 2C	*HTR2C*	P28335	Unknown
	5-hydroxytryptamine receptor 1D	*HTR1D*	P28221	Unknown
	5-hydroxytryptamine receptor 1B	*HTR1B*	P28222	Unknown
Selegiline	Amine oxidase [flavin-containing] B	*MAOB*	P27338	Yes
Rasagiline	Amine oxidase [flavin-containing] B	*MAOB*	P27338	Yes
	Apoptosis regulator Bcl-2	*BCL2*	P10415	Yes
Safinamide	Amine oxidase [flavin-containing] B	*MAOB*	P27338	Yes
Zonisamide	Sodium channel protein type 1 subunit alpha	*SCN1A*	P35498	Yes
	Sodium channel protein type 2 subunit alpha	*SCN2A*	Q99250	Yes

	Sodium channel protein type 3 subunit alpha	*SCN3A*	Q9NY46	Yes
	Sodium channel protein type 4 subunit alpha	*SCN4A*	P35499	Yes
	Sodium channel protein type 5 subunit alpha	*SCN5A*	Q14524	Yes
	Sodium channel protein type 9 subunit alpha	*SCN9A*	Q15858	Yes
	Sodium channel protein type 11 subunit alpha	*SCN11A*	Q9UI33	Yes
	Sodium channel subunit beta-1	*SCN1B*	Q07699	Yes
	Sodium channel subunit beta-2	*SCN2B*	O60939	Yes
	Sodium channel subunit beta-3	*SCN3B*	Q9NY72	Yes
	Sodium channel subunit beta-4	*SCN4B*	Q8IWT1	Yes
	Voltage-dependent T-type calcium channel subunit alpha-1G	*CACNA1G*	O43497	Yes
	Voltage-dependent T-type calcium channel subunit alpha-1H	*CACNA1H*	O95180	Yes
	Voltage-dependent T-type calcium channel subunit alpha-1I	*CACNA1I*	Q9P0X4	Yes
	Carbonic anhydrase 1	*CA1*	P00915	Unknown
	Carbonic anhydrase 2	*CA2*	P00918	Unknown
	Carbonic anhydrase 3	*CA3*	P07451	Unknown
	Carbonic anhydrase 4	*CA4*	P22748	Unknown
	Carbonic anhydrase 5A, mitochondrial	*CA5A*	P35218	Unknown
	Carbonic anhydrase 5B, mitochondrial	*CA5B*	Q9Y2D0	Unknown

	Carbonic anhydrase 6	*CA6*	P23280	Unknown
	Carbonic anhydrase 7	*CA7*	P43166	Unknown
	Carbonic anhydrase-related protein	*CA8*	P35219	Unknown
	Carbonic anhydrase 9	*CA9*	Q16790	Unknown
	Carbonic anhydrase-related protein 10	*CA10*	Q9NS85	Unknown
	Carbonic anhydrase-related protein 11	*CA11*	O75493	Unknown
	Carbonic anhydrase 12	*CA12*	O43570	Unknown
	Carbonic anhydrase 13	*CA13*	Q8N1Q1	Unknown
	Carbonic anhydrase 14	*CA14*	Q9ULX7	Unknown
	Amine oxidase [flavin-containing] B	*MAOB*	P27338	Unknown
	Amine oxidase [flavin-containing] A	*MAOA*	P21397	Unknown
Entacapone	Catechol O-methyltransferase	*COMT*	P21964	Yes
Opicapone	Catechol O-methyltransferase	*COMT*	P21964	Yes
Tolcapone	Catechol O-methyltransferase	*COMT*	P21964	Yes
trihexyphenidyl	Muscarinic acetylcholine receptor M1	*CHRM1*	P11229	Yes
	Muscarinic acetylcholine receptor M2	*CHRM2*	P08172	Unknown
	Muscarinic acetylcholine receptor M3	*CHRM3*	P20309	Unknown
	Muscarinic acetylcholine receptor M4	*CHRM4*	P08173	Unknown
	Muscarinic acetylcholine receptor M5	*CHRM5*	P08912	Unknown
benztropine	Muscarinic acetylcholine receptor M1	*CHRM1*	P11229	Yes
	Sodium-dependent dopamine transporter	*SLC6A3*	Q01959	Yes
	Histamine H1 receptor	*HRH1*	P35367	Unknown
Amantadine	Matrix protein 2	*M*	P21430	Yes
	Glutamate receptor ionotropic, NMDA 3A	*GRIN3A*	Q8TCU5	Yes
	Dopamine D2 receptor	*DRD2*	P14416	Yes

	Neuronal acetylcholine receptor subunit alpha-7	*CHRNA7*	P36544	Unknown
	Neuronal acetylcholine receptor subunit alpha-4	*CHRNA4*	P43681	Unknown
	Neuronal acetylcholine receptor subunit alpha-3	*CHRNA3*	P32297	Unknown
Istradefylline	Adenosine receptor A2a	*ADORA2A*	P29274	Yes
Clozapine	Dopamine D2 receptor	*DRD2*	P14416	Yes
	5-hydroxytryptamine receptor 2A	*HTR2A*	P28223	Yes
	Dopamine D1 receptor	*DRD1*	P21728	Unknown
	Dopamine D3 receptor	*DRD3*	P35462	Unknown
	Dopamine D4 receptor	*DRD4*	P21917	Unknown
	5-hydroxytryptamine receptor 1A	*HTR1A*	P08908	Unknown
	5-hydroxytryptamine receptor 1B	*HTR1B*	P28222	Unknown
	5-hydroxytryptamine receptor 1D	*HTR1D*	P28221	Unknown
	5-hydroxytryptamine receptor 1E	*HTR1E*	P28566	Unknown
	5-hydroxytryptamine receptor 3A	*HTR3A*	P46098	Unknown
	5-hydroxytryptamine receptor 2C	*HTR2C*	P28335	Unknown
	5-hydroxytryptamine receptor 6	*HTR6*	P50406	Unknown
	5-hydroxytryptamine receptor 7	*HTR7*	P34969	Unknown
	Neuron-specific vesicular protein calcyon	*CALY*	Q9NYX4	Unknown
	Glutathione S-transferase P	*GSTP1*	P09211	Unknown

Pharmacologic Agents Used for Motor Symptoms in Parkinson Disease were summarized by Armstrong, M. J. et, al[8]. Targets are distilled from DrugBank database[9]. In this database, if a therapeutic action is driven by the activity of the drug towards that target, the pharmacological action tag will be "yes"; if the activity of the drug is not related to the therapeutic activity, the

pharmacological tag will be "no"; and lastly if the activity of the drug towards the target has not been established to be related to the therapeutic effect, the pharmacological tag will be "unknown". Targets with the tag of pharmacological action is "Yes" or "Unknown" were extracted.

Supplementary Table 13: Phenotypes used for phenome-wide MR.

PheCode	cases	controls	excluded controls	Phenotype Description	Phenotype Category
008.5	2737	399970	6254	Bacterial enteritis	infectious diseases
008.52	650	399970	8341	Intestinal infection due to C. difficile	infectious diseases
008.6	862	399970	8129	Viral Enteritis	infectious diseases
008	8991	399970	0	Intestinal infection	infectious diseases
038.1	832	393897	14232	Gram negative septicemia	infectious diseases
038	4005	393897	11059	Septicemia	infectious diseases
041.1	3149	393897	11915	Staphylococcus infections	infectious diseases
041.2	1644	393897	13420	Streptococcus infection	infectious diseases
041.4	2744	393897	12320	E. coli	infectious diseases
041	12187	393897	2877	Bacterial infection NOS	infectious diseases
070	1215	403316	4430	Viral hepatitis	infectious diseases
078	1007	403316	4638	Viral warts & HPV	infectious diseases
079	2806	403316	2839	Viral infection	infectious diseases
080	4489	402343	2129	Postoperative infection	infectious diseases
081	2485	402343	4133	Infection/inflammation of internal prosthetic device; implant; and graft	infectious diseases
1000	579	408382	0	Burns	injuries & poisonings
1001	1210	407751	0	Foreign body injury	injuries & poisonings
1002	5512	403449	0	Symptoms concerning nutrition, metabolism, and development	symptoms
1005	1222	407739	0	Other symptoms	symptoms
1008	1007	407954	0	Crushing or internal injury to organs	injuries & poisonings
1009	8440	400521	0	Injury, NOS	injuries & poisonings

1010	5972	402989	0	Other tests	symptoms
1011	9140	399821	0	Complications of surgical and medical procedures	injuries & poisonings
1015	4230	404731	0	Effects of other external causes	symptoms
1019	16398	392563	0	Other ill-defined and unknown causes of morbidity and mortality	symptoms
112	2134	406301	526	Candidiasis	infectious diseases
145	643	406821	1497	Cancer of mouth	neoplasms
149	628	406821	1512	Cancer of larynx, pharynx, nasal cavities	neoplasms
150	720	393372	14869	Cancer of esophagus	neoplasms
151	554	393372	15035	Cancer of stomach	neoplasms
153.2	3051	382756	23154	Colon cancer	neoplasms
153.3	2095	382756	24110	Malignant neoplasm of rectum, rectosigmoid junction, and anus	neoplasms
153	4562	382756	21643	Colorectal cancer	neoplasms
157	589	393372	15000	Pancreatic cancer	neoplasms
158	1056	393372	14533	Neoplasm of unspecified nature of digestive system	neoplasms
159	5584	393372	10005	Malignant neoplasm of other and ill-defined sites within the digestive organs and peritoneum	neoplasms
165.1	2101	406226	634	Cancer of bronchus; lung	neoplasms
165	2700	406226	35	Cancer within the respiratory system	neoplasms
172.1	2691	395071	11199	Melanomas of skin, dx or hx	neoplasms
172.11	2691	395071	11199	Melanomas of skin	neoplasms
172.2	11149	395071	2741	Other non-epithelial cancer of skin	neoplasms
172.3	667	395071	13223	Carcinoma in situ of skin	neoplasms
172	13752	395071	138	Skin cancer	neoplasms
174.1	12671	388549	7741	Breast cancer [female]	neoplasms

174.11	11874	388549	8538	Malignant neoplasm of female breast	neoplasms
174	12898	388549	7514	Breast cancer	neoplasms
175	2415	387566	18980	Acquired absence of breast	neoplasms
180.1	1659	381902	25400	Cervical cancer	neoplasms
180.3	2090	381902	24969	Cervical intraepithelial neoplasia [CIN] [Cervical dysplasia]	neoplasms
180	3653	381902	23406	Cervical cancer and dysplasia	neoplasms
182	1284	381967	25710	Malignant neoplasm of uterus	neoplasms
184.1	2127	389695	17139	Malignant neoplasm of ovary and other uterine adnexa	neoplasms
184.11	2103	389695	17163	Malignant neoplasm of ovary	neoplasms
184	2463	389695	16803	Cancer of other female genital organs	neoplasms
187.2	2981	401788	4192	Malignant neoplasm of testis	neoplasms
189.1	1045	404796	3120	Cancer of kidney and renal pelvis	neoplasms
189.11	1002	404796	3163	Malignant neoplasm of kidney, except pelvis	neoplasms
189.2	2427	404796	1738	Cancer of bladder	neoplasms
189.21	2146	404796	2019	Malignant neoplasm of bladder	neoplasms
189	4165	404796	0	Cancer of urinary organs (incl. kidney and bladder)	neoplasms
191.1	531	407239	1191	Cancer of brain and nervous system	neoplasms
191	655	407239	1067	Manlignant and unknown neoplasms of brain and nervous system	neoplasms
195.1	15979	370604	22378	Malignant neoplasm, other	neoplasms
195	16725	370604	21632	Cancer, suspected or other	neoplasms
197	21798	370604	16559	Chemotherapy	neoplasms
198.1	5379	370604	32978	Secondary malignancy of lymph nodes	neoplasms
198.2	2211	370604	36146	Secondary malignancy of respiratory organs	neoplasms
198.3	1519	370604	36838	Secondary malignant neoplasm of digestive systems	neoplasms

198.4	2638	370604	35719	Secondary malignant neoplasm of liver	neoplasms
198.5	806	370604	37551	Secondary malignancy of brain/spine	neoplasms
198.6	2151	370604	36206	Secondary malignancy of bone	neoplasms
198	9483	370604	28874	Secondary malignant neoplasm	neoplasms
199	1412	370604	36945	Neoplasm of uncertain behavior	neoplasms
200	995	404466	3500	Myeloproliferative disease	neoplasms
202.2	1793	404466	2702	Non-Hodgkins lymphoma	neoplasms
202.24	573	404466	3922	Large cell lymphoma	neoplasms
202	2270	404466	2225	Cancer of other lymphoid, histiocytic tissue	neoplasms
204.1	578	404466	3917	Lymphoid leukemia	neoplasms
204.12	506	404466	3989	Lymphoid leukemia, chronic	neoplasms
204.4	552	404466	3943	Multiple myeloma	neoplasms
204	1661	404466	2834	Leukemia	neoplasms
208	20204	386011	2746	Benign neoplasm of colon	neoplasms
210	984	406821	1156	Benign neoplasm of lip, oral cavity, and pharynx	neoplasms
211	5280	395301	8380	Benign neoplasm of other parts of digestive system	neoplasms
214.1	4611	401613	2737	Lipoma of skin and subcutaneous tissue	neoplasms
214	6271	401613	1077	Lipoma	neoplasms
215	1110	401613	6238	Other benign neoplasm of connective and other soft tissue	neoplasms
216	7722	400618	621	Benign neoplasm of skin	neoplasms
217.1	597	400618	7746	Nevus, non-neoplastic	neoplasms
217	649	400618	7694	Vascular hamartomas and non-neoplastic nevi	neoplasms
218.1	10345	391653	6963	Uterine leiomyoma	neoplasms
218	10610	391653	6698	Benign neoplasm of uterus	neoplasms
220	1482	380325	27154	Benign neoplasm of ovary	neoplasms

225.1	774	407239	948	Benign neoplasm of brain, cranial nerves, meninges	neoplasms
225	828	407239	894	Benign neoplasm of brain and other parts of nervous system	neoplasms
227	876	407399	686	Benign neoplasm of other endocrine glands and related structures	neoplasms
228	1603	407358	0	Hemangioma and lymphangioma, any site	neoplasms
229	2402	406559	0	Benign neoplasm of unspecified sites	neoplasms
240	602	391429	16930	Simple and unspecified goiter	endocrine/metabolic
241.2	680	391429	16852	Nontoxic multinodular goiter	endocrine/metabolic
241	1143	391429	16389	Nontoxic nodular goiter	endocrine/metabolic
242	1860	391429	15672	Thyrotoxicosis with or without goiter	endocrine/metabolic
244.1	1117	391429	16415	Secondary hypothyroidism	endocrine/metabolic
244.4	14171	391429	3361	Hypothyroidism NOS	endocrine/metabolic
244	14871	391429	2661	Hypothyroidism	endocrine/metabolic
250.1	2660	388756	17545	Type 1 diabetes	endocrine/metabolic
250.2	18945	388756	1260	Type 2 diabetes	endocrine/metabolic
250.23	1298	388756	18907	Type 2 diabetes with ophthalmic manifestations	endocrine/metabolic
250.24	575	388756	19630	Type 2 diabetes with neurological manifestations	endocrine/metabolic
250.4	685	388756	19520	Abnormal glucose	endocrine/metabolic
250.7	1339	396859	10763	Diabetic retinopathy	endocrine/metabolic
250	20203	388756	2	Diabetes mellitus	endocrine/metabolic
251.1	939	386319	21703	Hypoglycemia	endocrine/metabolic
251	943	405386	2632	Other disorders of pancreatic internal secretion	endocrine/metabolic
252.1	781	405386	2794	Hyperparathyroidism	endocrine/metabolic
252	877	405386	2698	Disorders of parathyroid gland	endocrine/metabolic

253	693	405386	2882	Disorders of the pituitary gland and its hypothalamic control	endocrine/metabolic
255	642	405386	2933	Disorders of adrenal glands	endocrine/metabolic
260.6	835	406492	1634	Anorexia	endocrine/metabolic
260	1057	406492	1412	Protein-calorie malnutrition	endocrine/metabolic
261.2	754	406492	1715	Vitamin B-complex deficiencies	endocrine/metabolic
261	1208	406492	1261	Vitamin deficiency	endocrine/metabolic
272.1	35844	373034	83	Hyperlipidemia	endocrine/metabolic
272.11	33242	373034	2685	Hypercholesterolemia	endocrine/metabolic
272	35927	373034	0	Disorders of lipoid metabolism	endocrine/metabolic
274.1	3195	405198	568	Gout	endocrine/metabolic
274.2	616	405198	3147	Crystal arthropathies	endocrine/metabolic
274.21	560	405198	3203	Chondrocalcinosis	endocrine/metabolic
274	3763	405198	0	Gout and other crystal arthropathies	endocrine/metabolic
275.1	669	406834	1458	Disorders of iron metabolism	hematopoietic
275.5	1204	406834	923	Disorders of calcium/phosphorus metabolism	endocrine/metabolic
275	2127	406834	0	Disorders of mineral metabolism	endocrine/metabolic
276.1	4123	401506	3332	Electrolyte imbalance	endocrine/metabolic
276.12	1826	401506	5629	Hyposmolality and/or hyponatremia	endocrine/metabolic
276.13	975	401506	6480	Hyperpotassemia	endocrine/metabolic
276.14	1430	401506	6025	Hypopotassemia	endocrine/metabolic
276.4	1161	401506	6294	Acid-base balance disorder	endocrine/metabolic
276.41	1055	401506	6400	Acidosis	endocrine/metabolic
276.5	2834	401506	4621	Hypovolemia	endocrine/metabolic
276	7455	401506	0	Disorders of fluid, electrolyte, and acid-base balance	endocrine/metabolic
277	1424	407537	0	Other disorders of metabolism	endocrine/metabolic

278.1	10799	397993	169	Obesity	endocrine/metabolic
278	10968	397993	0	Overweight, obesity and other hyperalimentation	endocrine/metabolic
280.1	7414	390026	11521	Iron deficiency anemias, unspecified or not due to blood loss	hematopoietic
280	7787	390026	11148	Iron deficiency anemias	hematopoietic
281.1	1076	390026	17859	Megaloblastic anemia	hematopoietic
281.11	754	390026	18181	Pernicious anemia	hematopoietic
281	1133	390026	17802	Other deficiency anemia	hematopoietic
285.2	702	390026	18233	Anemia of chronic disease	hematopoietic
285	12256	390026	6679	Other anemias	hematopoietic
286	941	406281	1739	Coagulation defects	hematopoietic
287.3	1563	406281	1117	Thrombocytopenia	hematopoietic
287	1791	406281	889	Purpura and other hemorrhagic conditions	hematopoietic
288.1	3184	401375	4402	Decreased white blood cell count	hematopoietic
288.11	3184	401375	4402	Neutropenia	hematopoietic
288	3788	401375	3798	Diseases of white blood cells	hematopoietic
289.4	2622	401375	4964	Lymphadenitis	hematopoietic
289.5	515	401375	7071	Diseases of spleen	hematopoietic
289	4177	401375	3409	Other diseases of blood and blood-forming organs	hematopoietic
290.1	956	402383	5622	Dementias	mental disorders
290.2	654	402383	5924	Delirium due to conditions classified elsewhere	mental disorders
290	1970	402383	4608	Delirium dementia and amnestic and other cognitive disorders	mental disorders
291	641	402383	5937	Other specified nonpsychotic and/or transient mental disorders	mental disorders
292.1	1514	402383	5064	Aphasia/speech disturbance	mental disorders

292.3	700	402383	5878	Memory loss	mental disorders
292.4	2189	402383	4389	Altered mental status	mental disorders
292	4655	402383	1923	Neurological disorders	mental disorders
293.1	907	405975	2079	Swelling, mass, or lump in head and neck [Space-occupying lesion, intracranial NOS]	mental disorders
293	2986	405975	0	Symptoms involving head and neck	mental disorders
295.1	571	365476	42914	Schizophrenia	mental disorders
295	850	365476	42635	Schizophrenia and other psychotic disorders	mental disorders
296.1	1064	365476	42421	Bipolar	mental disorders
296.2	11901	365476	31584	Depression	mental disorders
296	12560	365476	30925	Mood disorders	mental disorders
300.1	6375	365476	37110	Anxiety disorder	mental disorders
300.12	709	365476	42776	Agorophobia, social phobia, and panic disorder	mental disorders
300.13	503	365476	42982	Phobia	mental disorders
300	6939	365476	36546	Anxiety disorders	mental disorders
303	529	365476	42956	Psychogenic and somatoform disorders	mental disorders
306	28791	365476	14694	Other mental disorder	mental disorders
317.1	8968	379355	20638	Alcoholism	mental disorders
317.11	802	379355	28804	Alcoholic liver damage	mental disorders
317	12922	379355	16684	Alcohol-related disorders	mental disorders
318	19780	379355	9826	Tobacco use disorder	mental disorders
327.3	4471	403723	767	Sleep apnea	neurological
327	5238	403723	0	Sleep disorders	neurological
331	1112	395209	12640	Other cerebral degenerations	neurological
333	891	395209	12861	Extrapyramidal disease and abnormal movement disorders	neurological

334	1789	395209	11963	Degenerative disease of the spinal cord	neurological
335	1356	395209	12396	Multiple sclerosis	neurological
338	771	408190	0	Pain	symptoms
339	7891	398780	2290	Other headache syndromes	neurological
340	2870	398780	7311	Migraine	neurological
342	1500	395209	12252	Hemiplegia	neurological
344	660	395209	13092	Other paralytic syndromes	neurological
345.1	901	395209	12851	Epilepsy	neurological
345.3	2232	395209	11520	Convulsions	neurological
345	5087	395209	8665	Epilepsy, recurrent seizures, convulsions	neurological
348	1426	395209	12326	Other conditions of brain	neurological
350.1	888	406217	1856	Abnormal involuntary movements	neurological
350.2	1601	406217	1143	Abnormality of gait	neurological
350	2744	406217	0	Abnormal movement	neurological
351	12592	394067	2302	Other peripheral nerve disorders	neurological
352.2	880	394067	14014	Facial nerve disorders [CN7]	neurological
352	1393	394067	13501	Disorders of other cranial nerves	neurological
353	1139	394067	13755	Nerve root and plexus disorders	neurological
357	1368	406852	741	Inflammatory and toxic neuropathy	neurological
361.1	1392	397761	9808	Retinal detachment with retinal defect	sense organs
361	3263	397761	7937	Retinal detachments and defects	sense organs
362.2	2191	396859	9911	Degeneration of macula and posterior pole of retina	sense organs
362.29	2188	396859	9914	Macular degeneration (senile) of retina NOS	sense organs
362.4	849	396859	11253	Retinal vascular changes and abnomalities	sense organs
362	3867	396859	8235	Other retinal disorders	sense organs
364	732	397761	10468	Corneal opacity and other disorders of cornea	sense organs

365.1	1043	397761	10157	Open-angle glaucoma	sense organs
365.11	1037	397761	10163	Primary open angle glaucoma	sense organs
365.2	705	397761	10495	Primary angle-closure glaucoma	sense organs
365	4462	397761	6738	Glaucoma	sense organs
366.2	8369	388609	11983	Senile cataract	sense organs
366	20352	388609	0	Cataract	sense organs
367.1	1257	406530	1174	Myopia	sense organs
367.9	723	406530	1708	Blindness and low vision	sense organs
367	2431	406530	0	Disorders of refraction and accommodation; blindness and low vision	sense organs
368.1	538	405654	2769	Amblyopia	sense organs
368.2	732	405654	2575	Diplopia and disorders of binocular vision	sense organs
368.9	615	405654	2692	Subjective visual disturbances	sense organs
368	3307	405654	0	Visual disturbances	sense organs
369	560	399306	9095	Infection of the eye	sense organs
371.3	2396	399306	7259	Inflammation of eyelids	sense organs
371	3174	399306	6481	Inflammation of the eye	sense organs
372	765	399306	8890	Disorders of conjunctiva	sense organs
374.1	1068	399306	8587	Ectropion or entropion	sense organs
374.3	1834	399306	7821	Ptosis of eyelid	sense organs
374	5726	399306	3929	Other disorders of eyelids	sense organs
375.2	899	401245	6817	Epiphora	sense organs
375	2218	401245	5498	Disorders of lacrimal system	sense organs
378.1	918	401245	6798	Strabismus (not specified as paralytic)	sense organs
378	1442	401245	6274	Strabismus and other disorders of binocular eye movements	sense organs

379.2	1372	401245	6344	Disorders of vitreous body	sense organs
379.3	1825	401245	5891	Aphakia and other disorders of lens	sense organs
379	3910	401245	3806	Other disorders of eye	sense organs
380.1	524	407510	927	Otitis externa	sense organs
380	1451	407510	0	Disorders of external ear	sense organs
381.1	1824	404888	2249	Otitis media	sense organs
381.11	856	404888	3217	Suppurative and unspecified otitis media	sense organs
381	2259	404888	1814	Otitis media and Eustachian tube disorders	sense organs
384.4	1044	404888	3029	Perforation of tympanic membrane	sense organs
384	1364	404888	2709	Other disorders of tympanic membrane	sense organs
385.3	608	404888	3465	Cholesteatoma	sense organs
385	828	404888	3245	Other disorders of middle ear and mastoid	sense organs
386.1	578	402827	5556	Meniere's disease	sense organs
386.3	767	402827	5367	Labyrinthitis	sense organs
386.9	4611	402827	1523	Dizziness and giddiness (Light-headedness and vertigo)	sense organs
386	6134	402827	0	Vertiginous syndromes and other disorders of vestibular system	sense organs
389.4	515	404562	3884	Tinnitus	sense organs
389	4256	404562	143	Hearing loss	sense organs
394.2	2985	402421	3555	Mitral valve disease	circulatory system
394.3	1260	402421	5280	Aortic valve disease	circulatory system
394.7	1058	402421	5482	Disease of tricuspid valve	circulatory system
394	4895	402421	1645	Rheumatic disease of the heart valves	circulatory system
395.1	2892	402421	3648	Nonrheumatic mitral valve disorders	circulatory system
395.6	1499	402421	5041	Heart valve replaced	circulatory system
395	4239	402421	2301	Heart valve disorders	circulatory system

396	1049	402421	5491	Abnormal heart sounds	circulatory system
401.1	77723	330366	872	Essential hypertension	circulatory system
401.2	1719	330366	76876	Hypertensive heart and/or renal disease	circulatory system
401.22	1548	330366	77047	Hypertensive chronic kidney disease	circulatory system
401	77977	330366	618	Hypertension	circulatory system
402	1487	330366	77108	Elevated blood pressure reading without diagnosis of hypertension	circulatory system
411.1	5181	377103	26677	Unstable angina (intermediate coronary syndrome)	circulatory system
411.2	11703	377103	20155	Myocardial infarction	circulatory system
411.3	16175	377103	15683	Angina pectoris	circulatory system
411.4	20023	377103	11835	Coronary atherosclerosis	circulatory system
411.41	698	377103	31160	Aneurysm and dissection of heart	circulatory system
411.8	14921	377103	16937	Other chronic ischemic heart disease, unspecified	circulatory system
411.9	1169	377103	30689	Other acute and subacute forms of ischemic heart disease	circulatory system
411	31355	377103	503	Ischemic Heart Disease	circulatory system
414	1796	377103	30062	Other forms of chronic heart disease	circulatory system
415.2	590	402375	5996	Chronic pulmonary heart disease	circulatory system
415	4257	402375	2329	Pulmonary heart disease	circulatory system
416	2573	402375	4013	Cardiomegaly	circulatory system
418.1	3582	377532	27847	Precordial pain	circulatory system
418	31429	377532	0	Nonspecific chest pain	circulatory system
420.2	1273	405779	1909	Pericarditis	circulatory system
420.3	686	405779	2496	Endocarditis	circulatory system
420	2020	405779	1162	Carditis	circulatory system
425.1	1208	405779	1974	Primary/intrinsic cardiomyopathies	circulatory system
425	1247	405779	1935	Cardiomyopathy	circulatory system

426.2	2125	380919	25917	Atrioventricular [AV] block	circulatory system
426.21	988	380919	27054	First degree AV block	circulatory system
426.24	587	380919	27455	Atrioventricular block, complete	circulatory system
426.3	3353	380919	24689	Bundle branch block	circulatory system
426.31	1498	380919	26544	Right bundle branch block	circulatory system
426.32	1757	380919	26285	Left bundle branch block	circulatory system
426.9	2487	380919	25555	Cardiac pacemaker/device in situ	circulatory system
426.91	2283	380919	25759	Cardiac pacemaker in situ	circulatory system
426	6959	380919	21083	Cardiac conduction disorders	circulatory system
427.1	3225	380919	24817	Paroxysmal tachycardia, unspecified	circulatory system
427.11	2359	380919	25683	Paroxysmal supraventricular tachycardia	circulatory system
427.12	938	380919	27104	Paroxysmal ventricular tachycardia	circulatory system
427.2	14820	380919	13222	Atrial fibrillation and flutter	circulatory system
427.3	3236	380919	24806	Other specified cardiac dysrhythmias	circulatory system
427.4	1137	380919	26905	Cardiac arrest and ventricular fibrillation	circulatory system
427.42	927	380919	27115	Cardiac arrest	circulatory system
427.5	922	380919	27120	Arrhythmia (cardiac) NOS	circulatory system
427.6	536	380919	27506	Premature beats	circulatory system
427.7	2193	380919	25849	Tachycardia NOS	circulatory system
427.9	3832	380919	24210	Palpitations	circulatory system
427	24681	380919	3361	Cardiac dysrhythmias	circulatory system
428.1	2031	402834	4096	Congestive heart failure (CHF) NOS	circulatory system
428.2	4269	402834	1858	Heart failure NOS	circulatory system
428	5415	402834	712	Congestive heart failure; nonhypertensive	circulatory system
429.2	524	402834	5603	Abnormal function study of cardiovascular system	circulatory system

429	796	402834	5331	Ill-defined descriptions and complications of heart disease	circulatory system
430.1	812	399017	9132	Subarachnoid hemorrhage	circulatory system
430.2	700	399017	9244	Intracerebral hemorrhage	circulatory system
430	1796	399017	8148	Intracranial hemorrhage	circulatory system
433.1	1185	399017	8759	Occlusion and stenosis of precerebral arteries	circulatory system
433.2	4134	399017	5810	Occlusion of cerebral arteries	circulatory system
433.21	1501	399017	8443	Cerebral artery occlusion, with cerebral infarction	circulatory system
433.3	2920	399017	7024	Cerebral ischemia	circulatory system
433.31	2146	399017	7798	Transient cerebral ischemia	circulatory system
433.8	1263	399017	8681	Late effects of cerebrovascular disease	circulatory system
433	8742	399017	1202	Cerebrovascular disease	circulatory system
440.2	811	400595	7555	Atherosclerosis of the extremities	circulatory system
440	1324	400595	7042	Atherosclerosis	circulatory system
441	576	400595	7790	Vascular insufficiency of intestine	circulatory system
442.1	1374	400595	6992	Aortic aneurysm	circulatory system
442.11	903	400595	7463	Abdominal aortic aneurysm	circulatory system
442	1808	400595	6558	Other aneurysm	circulatory system
443.1	1148	400595	7218	Raynaud's syndrome	circulatory system
443.9	2566	400595	5800	Peripheral vascular disease, unspecified	circulatory system
443	3927	400595	4439	Peripheral vascular disease	circulatory system
444.1	557	400595	7809	Arterial embolism and thrombosis of lower extremity artery	circulatory system
444	921	400595	7445	Arterial embolism and thrombosis	circulatory system
446	828	400595	7538	Polyarteritis nodosa and allied conditions	circulatory system
447.1	873	400595	7493	Stricture of artery	circulatory system

447	1333	400595	7033	Other disorders of arteries and arterioles	circulatory system
450	705	408256	0	Noninfectious disorders of lymphatic channels	circulatory system
451.2	3587	369592	35782	Phlebitis and thrombophlebitis of lower extremities	circulatory system
451	3900	369592	35469	Phlebitis and thrombophlebitis	circulatory system
452	558	369592	38811	Other venous embolism and thrombosis	circulatory system
454.1	11697	369592	27672	Varicose veins of lower extremity	circulatory system
454.11	633	369592	38736	Varicose veins of lower extremity, symptomtic	circulatory system
454	12172	369592	27197	Varicose veins	circulatory system
455	23896	369592	15473	Hemorrhoids	circulatory system
458.1	1347	387905	19709	Orthostatic hypotension	circulatory system
458.9	3518	387905	17538	Hypotension NOS	circulatory system
458	5827	387905	15229	Hypotension	circulatory system
459.9	16366	387905	4690	Circulatory disease NEC	circulatory system
459	16544	387905	4512	Other disorders of circulatory system	circulatory system
465.2	870	406447	1644	Acute pharyngitis	respiratory
465	2335	406447	179	Acute upper respiratory infections of multiple or unspecified sites	respiratory
470	4939	390045	13977	Septal Deviations/Turbinate Hypertrophy	respiratory
471	3311	390045	15605	Nasal polyps	respiratory
472	966	390045	17950	Chronic pharyngitis and nasopharyngitis	respiratory
473.4	1086	390045	17830	Voice disturbance	respiratory
473	2630	390045	16286	Diseases of the larynx and vocal cords	respiratory
474.1	614	390045	18302	Acute tonsillitis	respiratory
474.2	1126	390045	17790	Chronic tonsillitis and adenoiditis	respiratory
474	2013	390045	16903	Acute and chronic tonsillitis	respiratory
475	2602	390045	16314	Chronic sinusitis	respiratory

476	1060	390045	17856	Allergic rhinitis	respiratory
477	2456	390045	16460	Epistaxis or throat hemorrhage	respiratory
479	4101	390045	14815	Other upper respiratory disease	respiratory
480.1	6710	398538	3713	Bacterial pneumonia	respiratory
480.11	5951	398538	4472	Pneumococcal pneumonia	respiratory
480	10059	398538	364	Pneumonia	respiratory
495	26332	375505	7124	Asthma	respiratory
496.1	1727	375505	31729	Emphysema	respiratory
496.2	2934	375505	30522	Chronic bronchitis	respiratory
496.21	2698	375505	30758	Obstructive chronic bronchitis	respiratory
496.3	1882	375505	31574	Bronchiectasis	respiratory
496	10502	375505	22954	Chronic airway obstruction	respiratory
497	631	375505	32825	Bronchitis	respiratory
501	586	397411	10964	Pneumonitis due to inhalation of food or vomitus	respiratory
502	887	397411	10663	Postinflammatory pulmonary fibrosis	respiratory
506	1174	397411	10376	Empyema and pneumothorax	respiratory
507	6448	397411	5102	Pleurisy; pleural effusion	respiratory
508	2005	397411	9545	Pulmonary collapse; interstitial and compensatory emphysema	respiratory
509.1	2018	397411	9532	Respiratory failure	respiratory
509.2	1749	397411	9801	Respiratory insufficiency	respiratory
509	2565	397411	8985	Respiratory failure, insufficiency, arrest	respiratory
510	781	408180	0	Other diseases of lung	respiratory
512.7	5884	399833	3244	Shortness of breath	respiratory
512.8	2884	399833	6244	Cough	respiratory
512.9	912	399833	8216	Other dyspnea	respiratory

512	9128	399833	0	Other symptoms of respiratory system	respiratory
513	611	408350	0	Respiratory abnormalities	respiratory
514	2500	406461	0	Abnormal findings examination of lungs	respiratory
516.1	2073	406794	94	Hemoptysis	respiratory
516	2167	406794	0	Abnormal sputum	respiratory
519.8	8844	399525	592	Other diseases of respiratory system, NEC	respiratory
519	9436	399525	0	Other diseases of respiratory system, not elsewhere classified	respiratory
520.2	2364	398136	8461	Disturbances in tooth eruption	digestive
520	2449	398136	8376	Disorders of tooth development	digestive
521.1	3051	398136	7774	Dental caries	digestive
521	3091	398136	7734	Diseases of hard tissues of teeth	digestive
522.5	1177	398136	9648	Periapical abscess	digestive
522	1795	398136	9030	Diseases of pulp and periapical tissues	digestive
523.3	1222	398136	9603	Periodontitis (acute or chronic)	digestive
523.31	668	398136	10157	Acute periodontitis	digestive
523.32	561	398136	10264	Chronic periodontitis	digestive
523	1742	398136	9083	Gingival and periodontal diseases	digestive
525	2689	398136	8136	Other diseases of the teeth and supporting structures	digestive
526	960	398136	9865	Diseases of the jaws	digestive
527	710	403323	4928	Diseases of the salivary glands	digestive
528.5	684	403323	4954	Diseases of lips	digestive
528	3939	403323	1699	Diseases of the oral soft tissues, excluding lesions specific for gingiva and tongue	digestive
529	1236	403323	4402	Diseases and other conditions of the tongue	digestive
530.1	32108	369275	7578	Esophagitis, GERD and related diseases	digestive

530.11	14223	369275	25463	GERD	digestive
530.12	5243	369275	34443	Ulcer of esophagus	digestive
530.14	10551	369275	29135	Reflux esophagitis	digestive
530.2	1672	369275	38014	Esophageal bleeding (varices/hemorrhage)	digestive
530.3	1874	369275	37812	Stricture and stenosis of esophagus	digestive
530.5	639	369275	39047	Disorders of esophageal motility	digestive
530.9	2100	369275	37586	Heartburn	digestive
530	35852	369275	3834	Diseases of esophagus	digestive
531.1	617	401525	6819	Hemorrhage from gastrointestinal ulcer	digestive
531.2	4109	401525	3327	Gastric ulcer	digestive
531.3	3002	401525	4434	Duodenal ulcer	digestive
531	7436	401525	0	Peptic ulcer (excl. esophageal)	digestive
532	6482	369275	33204	Dysphagia	digestive
535.1	1184	378124	29653	Acute gastritis	digestive
535.6	7655	378124	23182	Duodenitis	digestive
535.8	8147	378124	22690	Other specified gastritis	digestive
535	28941	378124	1896	Gastritis and duodenitis	digestive
537	3404	378124	27433	Other disorders of stomach and duodenum	digestive
540.1	3217	405552	192	Appendicitis	digestive
540.11	2608	405552	801	Acute appendicitis	digestive
540	3409	405552	0	Appendiceal conditions	digestive
550.1	15995	361617	31349	Inguinal hernia	digestive
550.2	27126	361617	20218	Diaphragmatic hernia	digestive
550.3	651	361617	46693	Femoral hernia	digestive
550.4	3727	361617	43617	Umbilical hernia	digestive
550.5	3448	361617	43896	Ventral hernia	digestive

550	47344	361617	0	Abdominal hernia	digestive
555.1	1743	334783	72435	Regional enteritis	digestive
555.2	3195	334783	70983	Ulcerative colitis	digestive
555.21	539	334783	73639	Ulcerative colitis (chronic)	digestive
555	4528	334783	69650	Inflammatory bowel disease and other gastroenteritis and colitis	digestive
556.1	674	334783	73504	Ulceration of intestine	digestive
556	1063	334783	73115	Ulceration of the lower GI tract	digestive
557.1	1855	334783	72323	Celiac disease	digestive
557	2103	334783	72075	Intestinal malabsorption (non-celiac)	digestive
558	15747	334783	58431	Noninfectious gastroenteritis	digestive
559	1660	334783	72518	Ileostomy status	digestive
560.1	522	334783	73656	Paralytic ileus	digestive
560.3	832	334783	73346	Peritoneal or intestinal adhesions	digestive
560.4	3346	334783	70832	Other intestinal obstruction	digestive
560	3994	334783	70184	Intestinal obstruction without mention of hernia	digestive
561.2	1689	334783	72489	Flatulence	digestive
561	15977	334783	58201	Symptoms involving digestive system	digestive
562.1	27268	334783	46910	Diverticulosis	digestive
562	27311	334783	46867	Diverticulosis and diverticulitis	digestive
563	10442	334783	63736	Constipation	digestive
564.1	5548	334783	68630	Irritable Bowel Syndrome	digestive
564.8	1650	334783	72528	Abnormal findings on exam of gastrointestinal tract/ abdominal area	digestive
564.9	15392	334783	58786	Personal history of diseases of digestive system	digestive
564	22138	334783	52040	Functional digestive disorders	digestive

565.1	7408	387338	14215	Anal and rectal polyp	digestive
565	14997	387338	6626	Anal and rectal conditions	digestive
567	887	387338	20736	Peritonitis and retroperitoneal infections	digestive
568.1	3108	387338	18515	Peritoneal adhesions (postoperative) (postinfection)	digestive
568	3308	387338	18315	Other disorders of peritoneum	digestive
569	4200	387338	17423	Other disorders of intestine	digestive
571.5	1664	400055	7242	Other chronic nonalcoholic liver disease	digestive
571.8	942	400055	7964	Liver abscess and sequelae of chronic liver disease	digestive
571.81	529	400055	8377	Portal hypertension	digestive
571	2895	400055	6011	Chronic liver disease and cirrhosis	digestive
572	1547	400055	7359	Ascites (non malignant)	digestive
573.5	897	400055	8009	Jaundice (not of newborn)	digestive
573.7	3479	400055	5427	Abnormal results of function study of liver	digestive
573	5847	400055	3059	Other disorders of liver	digestive
574.1	13777	391307	3877	Cholelithiasis	digestive
574.11	1513	391307	16141	Cholelithiasis with acute cholecystitis	digestive
574.12	5472	391307	12182	Cholelithiasis with other cholecystitis	digestive
574.2	2634	391307	15020	Calculus of bile duct	digestive
574.3	2761	391307	14893	Cholecystitis without cholelithiasis	digestive
574	16225	391307	1429	Cholelithiasis and cholecystitis	digestive
575.2	777	391307	16877	Obstruction of bile duct	digestive
575.7	1412	391307	16242	Other disorders of gallbladder	digestive
575.8	1040	391307	16614	Other disorders of biliary tract	digestive
575	3892	391307	13762	Other biliary tract disease	digestive
577.1	1986	406271	704	Acute pancreatitis	digestive
577.2	514	406271	2176	Chronic pancreatitis	digestive

577	2690	406271	0	Diseases of pancreas	digestive
578.1	1961	385157	21843	Hematemesis	digestive
578.2	2639	385157	21165	Blood in stool	digestive
578.8	13222	385157	10582	Hemorrhage of rectum and anus	digestive
578.9	5229	385157	18575	Hemorrhage of gastrointestinal tract	digestive
578	21137	385157	2667	Gastrointestinal hemorrhage	digestive
579.8	1659	385157	22145	Nonspecific abnormal findings in stool contents	digestive
579	3195	385157	20609	Other symptoms involving abdomen and pelvis	digestive
580.1	1033	397602	10326	Glomerulonephritis	genitourinary
580.14	845	397602	10514	Chronic glomerulonephritis, NOS	genitourinary
580	1522	397602	9837	Nephritis; nephrosis; renal sclerosis	genitourinary
585.1	4521	397602	6838	Acute renal failure	genitourinary
585.2	1412	397602	9947	Renal failure NOS	genitourinary
585.3	2629	397602	8730	Chronic renal failure [CKD]	genitourinary
585	6985	397602	4374	Renal failure	genitourinary
586.2	1260	397602	10099	Cyst of kidney, acquired	genitourinary
586.4	913	397602	10446	Stricture/obstruction of ureter	genitourinary
586	3362	397602	7997	Other disorders of the kidney and ureters	genitourinary
590	1324	379936	27701	Pyelonephritis	genitourinary
591	12491	379936	16534	Urinary tract infection	genitourinary
592.1	2948	379936	26077	Cystitis	genitourinary
592.12	892	379936	28133	Chronic cystitis	genitourinary
592	3088	379936	25937	Cystitis and urethritis	genitourinary
593	16409	379936	12616	Hematuria	genitourinary
594.1	3191	401005	4765	Calculus of kidney	genitourinary
594.2	778	401005	7178	Calculus of lower urinary tract	genitourinary

594.3	2417	401005	5539	Calculus of ureter	genitourinary
594.8	2055	401005	5901	Renal colic	genitourinary
594	6643	401005	1313	Urinary calculus	genitourinary
595	1951	401005	6005	Hydronephrosis	genitourinary
596.1	1980	394699	12282	Bladder neck obstruction	genitourinary
596.5	1664	394699	12598	Functional disorders of bladder	genitourinary
596	9933	394699	4329	Other disorders of bladder	genitourinary
597.1	3331	394699	10931	Urethral stricture (not specified as infectious)	genitourinary
597	4069	394699	10193	Other disorders of urethra and urinary tract	genitourinary
598	3547	405414	0	Abnormal findings on examination of urine	genitourinary
599.2	6755	384930	17276	Retention of urine	genitourinary
599.3	1199	384930	22832	Dysuria	genitourinary
599.4	8856	384930	15175	Urinary incontinence	genitourinary
599.5	4037	384930	19994	Frequency of urination and polyuria	genitourinary
599.9	1926	384930	22105	Other abnormality of urination	genitourinary
599	24031	384930	0	Other symptoms/disorders or the urinary system	genitourinary
608	2833	389094	17034	Other disorders of male genital organs	genitourinary
610.1	928	401746	6287	Cystic mastopathy	genitourinary
610.4	1553	401746	5662	Benign neoplasm of breast	genitourinary
610.8	696	401746	6519	Other specified benign mammary dysplasias	genitourinary
610	3379	401746	3836	Benign mammary dysplasias	genitourinary
611.3	1525	401746	5690	Lump or mass in breast	genitourinary
611	1580	401746	5635	Abnormal findings on mammogram or breast exam	genitourinary
612.2	854	401746	6361	Hypertrophy of breast (Gynecomastia)	genitourinary
612	942	401746	6273	Breast conditions, congenital or relating to hormones	genitourinary
613.1	723	406978	1260	Inflammatory disease of breast	genitourinary

613.7	704	406978	1279	Other signs and symptoms in breast	genitourinary
613	1983	406978	0	Other nonmalignant breast conditions	genitourinary
614.1	2546	399757	6658	Pelvic peritoneal adhesions, female (postoperative) (postinfection)	genitourinary
614.3	936	399757	8268	Pelvic inflammatory disease (PID)	genitourinary
614.5	2864	399757	6340	Inflammatory disease of cervix, vagina, and vulva	genitourinary
614.51	1323	399757	7881	Cervicitis and endocervicitis	genitourinary
614.53	758	399757	8446	Cyst or abscess of Bartholin's gland	genitourinary
614	6204	399757	3000	Inflammatory diseases of female pelvic organs	genitourinary
615	4053	399757	5151	Endometriosis	genitourinary
618.1	7462	396730	4769	Prolapse of vaginal walls	genitourinary
618.2	5428	396730	6803	Uterine/Uterovaginal prolapse	genitourinary
618.5	513	396730	11718	Prolapse of vaginal vault after hysterectomy	genitourinary
618.6	681	396730	11550	Vaginal enterocele, congenital or acquired	genitourinary
618	11966	396730	265	Genital prolapse	genitourinary
619.1	730	399629	8602	Noninflammatory disorders of ovary, fallopian tube, and broad ligament	genitourinary
619.2	3336	399629	5996	Disorders of uterus, NEC	genitourinary
619.3	2884	399629	6448	Noninflammatory disorders of cervix	genitourinary
619.4	1776	399629	7556	Noninflammatory disorders of vagina	genitourinary
619.5	1355	399629	7977	Noninflammatory disorders of vulva and perineum	genitourinary
619	9332	399629	0	Noninflammatory female genital disorders	genitourinary
621	1133	396384	11444	Endometrial hyperplasia	genitourinary
622.1	7910	396384	4667	Polyp of corpus uteri	genitourinary
622.2	3450	396384	9127	Mucous polyp of cervix	genitourinary
622	10881	396384	1696	Polyp of female genital organs	genitourinary

623	1275	396384	11302	Hypertrophy of female genital organs	genitourinary
624.9	5924	399509	3528	stress incontinence, female	genitourinary
624	6688	399509	2764	Symptoms involving female genital tract	genitourinary
625.1	1192	399509	8260	Dyspareunia	genitourinary
625	3003	399509	6449	Pain and other symptoms associated with female genital organs	genitourinary
626.1	15880	377857	15224	Irregular menstrual cycle/bleeding	genitourinary
626.12	9820	377857	21284	Excessive or frequent menstruation	genitourinary
626.13	1989	377857	29115	Irregular menstrual cycle	genitourinary
626.14	3817	377857	27287	Irregular menstrual bleeding	genitourinary
626.2	1787	377857	29317	Dysmenorrhea	genitourinary
626.8	1370	377857	29734	Infertility, female	genitourinary
626	18580	377857	12524	Disorders of menstruation and other abnormal bleeding from female genital tract	genitourinary
627.1	9109	377857	21995	Postmenopausal bleeding	genitourinary
627.3	1115	377857	29989	Postmenopausal atrophic vaginitis	genitourinary
627	10699	377857	20405	Menopausal and postmenopausal disorders	genitourinary
628	4777	377857	26327	Ovarian cyst	genitourinary
634.1	1251	400946	6764	Missed abortion/Hydatidiform mole	pregnancy complications
634	5463	400946	2552	Miscarriage; stillbirth	pregnancy complications
635.2	866	400946	7149	Antepartum hemorrhage, abruptio placentae, and placenta previa	pregnancy complications
635.3	1274	400946	6741	Placenta previa and abruptio placenta	pregnancy complications
635	2005	400946	6010	Hemorrhage during pregnancy; childbirth and postpartum	pregnancy complications
636.2	617	400946	7398	Early onset of delivery	pregnancy complications

636.3	1083	400946	6932	Hemorrhage in early pregnancy	pregnancy complications
636	2558	400946	5457	Early or threatened labor; hemorrhage in early pregnancy	pregnancy complications
642	1114	407847	0	Hypertension complicating pregnancy, childbirth, and the puerperium	pregnancy complications
645	1256	407705	0	Late pregnancy and failed induction	pregnancy complications
646	2235	406726	0	Other complications of pregnancy NEC	pregnancy complications
650	1928	407033	0	Normal delivery	pregnancy complications
652	1720	405994	1247	Malposition and malpresentation of fetus or obstruction	digestive
653	1404	405994	1563	Problems associated with amniotic cavity and membranes	pregnancy complications
655	4325	404636	0	Known or suspected fetal abnormality affecting management of mother	pregnancy complications
661	2272	401037	5652	Fetal distress and abnormal forces of labor	pregnancy complications
663	533	401037	7391	Umbilical cord complications during labor and delivery	pregnancy complications
665	5335	401037	2589	Obstetrical/birth trauma	pregnancy complications
669	2559	401037	5365	Complications of labor and delivery NEC	pregnancy complications
681.1	566	397635	10760	Cellulitis and abscess of fingers/toes	dermatologic
681.2	524	397635	10802	Cellulitis and abscess of face/neck	dermatologic
681.3	5539	397635	5787	Cellulitis and abscess of arm/hand	dermatologic
681.5	5547	397635	5779	Cellulitis and abscess of leg, except foot	dermatologic
681.6	5502	397635	5824	Cellulitis and abscess of foot, toe	dermatologic
681.7	602	397635	10724	Cellulitis and abscess of trunk	dermatologic
681	7451	397635	3875	Superficial cellulitis and abscess	dermatologic
686.1	2302	397635	9024	Carbuncle and furuncle	dermatologic
686.3	587	397635	10739	Pilonidal cyst	dermatologic
686	4520	397635	6806	Other local infections of skin and subcutaneous tissue	dermatologic

687.1	2157	403407	3397	Rash and other nonspecific skin eruption	dermatologic
687.4	2900	403407	2654	Disturbance of skin sensation	dermatologic
687	5554	403407	0	Symptoms affecting skin	dermatologic
689	5782	403179	0	Disorder of skin and subcutaneous tissue NOS	dermatologic
694.2	783	402672	5506	Other dyschromia	dermatologic
694	987	402672	5302	Dyschromia and Vitiligo	dermatologic
695.7	779	402672	5510	Prurigo and Lichen	dermatologic
695	2420	402672	3869	Erythematous conditions	dermatologic
696.4	2237	398199	8525	Psoriasis	dermatologic
696.41	1684	398199	9078	Psoriasis vulgaris	dermatologic
696.42	708	398199	10054	Psoriatic arthropathy	dermatologic
696	2293	398199	8469	Psoriasis and related disorders	dermatologic
697	548	402672	5741	Sarcoidosis	dermatologic
698	783	408178	0	Pruritus and related conditions	dermatologic
701.2	2356	403875	2730	Scar conditions and fibrosis of skin	dermatologic
701	4804	403875	282	Other hypertrophic and atrophic conditions of skin	dermatologic
702.1	2594	403439	2928	Actinic keratosis	dermatologic
702.2	3092	403439	2430	Seborrheic keratosis	dermatologic
702	5522	398746	4693	Degenerative skin conditions and other dermatoses	dermatologic
703.1	970	402357	5634	Ingrowing nail	dermatologic
703	1287	402357	5317	Diseases of nail, NOS	dermatologic
704	5344	402357	1260	Diseases of hair and hair follicles	dermatologic
705.8	592	399255	9114	Hyperhidrosis	dermatologic
705	817	399255	8889	Disorders of sweat glands	dermatologic
706.2	8876	399255	830	Sebaceous cyst	dermatologic
706	8948	399255	758	Diseases of sebaceous glands	dermatologic

707.1	913	406973	1075	Decubitus ulcer	dermatologic
707	1988	406973	0	Chronic ulcer of skin	dermatologic
709.2	513	399404	9044	Sicca syndrome	dermatologic
709.7	2720	399404	6837	Unspecified diffuse connective tissue disease	dermatologic
709	3463	399404	6094	Diffuse diseases of connective tissue	dermatologic
710.1	565	365819	42577	Osteomyelitis	musculoskeletal
710	612	365819	42530	Osteomyelitis, periostitis, and other infections involving bone	musculoskeletal
714.1	4412	365085	39464	Rheumatoid arthritis	musculoskeletal
714	4879	365085	38997	Rheumatoid arthritis and other inflammatory polyarthropathies	musculoskeletal
715.2	620	365085	43256	Ankylosing spondylitis	musculoskeletal
715	1671	365085	42205	Other inflammatory spondylopathies	musculoskeletal
716.1	3535	365819	39607	Unspecified polyarthropathy or polyarthritis	musculoskeletal
716.2	15790	365819	27352	Unspecified monoarthritis	musculoskeletal
716.9	37043	365819	6099	Arthropathy NOS	musculoskeletal
716	38715	365819	4427	Other arthropathies	musculoskeletal
717	1152	407809	0	Polymyalgia Rheumatica	musculoskeletal
720	3733	391917	13311	Spinal stenosis	musculoskeletal
721.1	5077	391917	11967	Spondylosis without myelopathy	musculoskeletal
721	7930	391917	9114	Spondylosis and allied disorders	musculoskeletal
722.1	513	391917	16531	Displacement of intervertebral disc	musculoskeletal
722.6	2846	391917	14198	Degeneration of intervertebral disc	musculoskeletal
722.9	4434	391917	12610	Other and unspecified disc disorder	musculoskeletal
722	9241	391917	7803	Intervertebral disc disorders	musculoskeletal
723	521	391917	16523	Other disorders of cervical region	musculoskeletal

724.9	1617	391917	15427	Other unspecified back disorders	musculoskeletal
724	2077	391917	14967	Other and unspecified disorders of back	musculoskeletal
726.1	9668	378711	20582	Enthesopathy	musculoskeletal
726.3	858	378711	29392	Bursitis	musculoskeletal
726	14983	378711	15267	Peripheral enthesopathies and allied syndromes	musculoskeletal
727.1	2754	378711	27496	Synovitis and tenosynovitis	musculoskeletal
727.4	3185	378711	27065	Ganglion and cyst of synovium, tendon, and bursa	musculoskeletal
727.5	593	378711	29657	Rupture of synovium	musculoskeletal
727	7629	378711	22621	Other disorders of synovium, tendon, and bursa	musculoskeletal
728.7	3843	378711	26407	Fasciitis	musculoskeletal
728.71	3503	378711	26747	Contracture of palmar fascia [Dupuytren's disease]	musculoskeletal
728	4488	378711	25762	Disorders of muscle, ligament, and fascia	musculoskeletal
729.1	1010	378711	29240	Rheumatism, unspecified and fibrositis	musculoskeletal
729	6170	378711	24080	Other disorders of soft tissues	musculoskeletal
733.4	542	391041	17378	Aseptic necrosis of bone	musculoskeletal
733.8	1268	391041	16652	Malunion and nonunion of fracture	musculoskeletal
733	3773	391041	14147	Other disorders of bone and cartilage	musculoskeletal
735.2	5144	394914	8903	Acquired toe deformities	musculoskeletal
735.21	1939	394914	12108	Hammer toe (acquired)	musculoskeletal
735.23	1561	394914	12486	Hallux rigidus	musculoskeletal
735.3	6699	394914	7348	Hallux valgus (Bunion)	musculoskeletal
735	9865	394914	4182	Acquired foot deformities	musculoskeletal
736	1649	394914	12398	Other acquired deformities of limbs	musculoskeletal
737.3	1063	394914	12984	Kyphoscoliosis and scoliosis	musculoskeletal
737	1134	394914	12913	Curvature of spine	musculoskeletal
738.4	1521	394914	12526	Acquired spondylolisthesis	musculoskeletal

738	1921	394914	12126	Other acquired musculoskeletal deformity	musculoskeletal
740.1	17691	380522	10748	Osteoarthritis; localized	musculoskeletal
740.11	9069	380522	19370	Osteoarthrosis, localized, primary	musculoskeletal
740.2	599	380522	27840	Osteoarthrosis, generalized	musculoskeletal
740.9	12436	380522	16003	Osteoarthrosis NOS	musculoskeletal
740	28439	380522	0	Osteoarthrosis	musculoskeletal
741.2	615	402633	5713	Stiffness of joint	musculoskeletal
741.4	1425	402633	4903	Joint effusions	musculoskeletal
741	3634	402633	2694	Symptoms and disorders of the joints	musculoskeletal
742.9	2000	402633	4328	Other derangement of joint	musculoskeletal
742	2913	402633	3415	Derangement of joint, non-traumatic	musculoskeletal
743.1	6484	401279	1198	Osteoporosis	musculoskeletal
743.11	5622	401279	2060	Osteoporosis NOS	musculoskeletal
743.13	532	401279	7150	Other specified osteoporosis	musculoskeletal
743.2	514	401279	7168	Pathologic fracture	musculoskeletal
743.9	820	401279	6862	Osteopenia or other disorder of bone and cartilage	musculoskeletal
743	7682	401279	0	Osteoporosis, osteopenia and pathological fracture	musculoskeletal
745	8037	400924	0	Pain in joint	musculoskeletal
747.1	2618	406165	178	Cardiac congenital anomalies	congenital anomalies
747.11	586	406165	2210	Cardiac shunt/ heart septal defect	congenital anomalies
747.13	1799	406165	997	Congenital anomalies of great vessels	congenital anomalies
747	2796	406165	0	Cardiac and circulatory congenital anomalies	congenital anomalies
750	703	406730	1528	Digestive congenital anomalies	congenital anomalies
751.1	732	406730	1499	Congenital anomalies of genital organs	congenital anomalies
751.2	855	406730	1376	Congenital anomalies of urinary system	congenital anomalies
751	1596	406730	635	Genitourinary congenital anomalies	congenital anomalies

756	594	407831	536	Other congenital musculoskeletal anomalies	congenital anomalies
760	11274	397687	0	Back pain	symptoms
761	1796	407165	0	Cervicalgia	symptoms
764	2383	405481	1097	Sciatica	symptoms
766	1181	405481	2299	Neuralgia, neuritis, and radiculitis NOS	symptoms
770	642	408319	0	Myalgia and myositis unspecified	symptoms
771.1	4663	403468	830	Swelling of limb	symptoms
771	5493	403468	0	Musculoskeletal symptoms referable to limbs	symptoms
772	698	408263	0	Symptoms of the muscles	symptoms
773	6327	402634	0	Pain in limb	symptoms
781	1886	407075	0	Symptoms involving nervous and musculoskeletal systems	symptoms
782.3	1648	407145	168	Edema	symptoms
782	1816	407145	0	Symptoms involving skin and other integumentary tissue	symptoms
783	3940	405021	0	Fever of unknown origin	symptoms
785	41316	367645	0	Abdominal pain	symptoms
788	9163	399798	0	Syncope and collapse	symptoms
789	11706	397255	0	Nausea and vomiting	symptoms
790.6	6413	402342	206	Other abnormal blood chemistry	symptoms
790	6619	402342	0	Nonspecific findings on examination of blood	symptoms
791	550	408411	0	Gangrene	symptoms
793.2	567	408263	131	Nonspecific abnormal findings on radiological and other examination of other intrathoracic organs (echocardiogram, etc)	circulatory system
793	698	408263	0	Nonspecific abnormal findings on radiological and other examination of musculoskeletal system	injuries & poisonings

798.1	593	405532	2836	Chronic fatigue syndrome	symptoms
798	3429	405532	0	Malaise and fatigue	symptoms
800.1	1814	387765	19382	Fracture of neck of femur	injuries & poisonings
800.3	2162	387765	19034	Fracture of tibia and fibula	injuries & poisonings
800.4	768	387765	20428	Fracture of patella	injuries & poisonings
800	7251	387765	13945	Fracture of lower limb	injuries & poisonings
801.1	1842	387765	19354	Fracture of foot	injuries & poisonings
801	2339	387765	18857	Fracture of ankle and foot	injuries & poisonings
802	722	387765	20474	Fracture of pelvis	injuries & poisonings
803.1	1692	387765	19504	Fracture of humerus	injuries & poisonings
803.2	5246	387765	15950	Fracture of radius and ulna	injuries & poisonings
803.3	1521	387765	19675	Fracture of clavicle or scapula	injuries & poisonings
803	8521	387765	12675	Fracture of upper limb	injuries & poisonings
804	3357	387765	17839	Fracture of hand or wrist	injuries & poisonings
805	1352	387765	19844	Fracture of vertebral column without mention of spinal cord injury	injuries & poisonings
807	775	387765	20421	Fracture of ribs	injuries & poisonings
809	1304	387765	19892	Fracture of unspecified bones	injuries & poisonings
818	508	405554	2899	Intracranial hemorrhage (injury)	injuries & poisonings
819	2957	405554	450	Skull and face fracture and other intercranial injury	injuries & poisonings
823	1674	385157	22130	Torus fracture	injuries & poisonings
830	2094	391457	15410	Dislocation	injuries & poisonings
835	15430	391457	2074	Internal derangement of knee	injuries & poisonings
836	528	391457	16976	Traumatic arthropathy	injuries & poisonings
850	5329	394929	8703	Hemorrhage or hematoma complicating a procedure	injuries & poisonings
853	502	394929	13530	Complication of colostomy or enterostomy	injuries & poisonings

854	1789	394929	12243	Complications of cardiac/vascular device, implant, and graft	injuries & poisonings
857	1241	394929	12791	Mechanical complication of unspecified genitourinary device, implant, and graft	injuries & poisonings
858	3167	394929	10865	Complication of internal orthopedic device	injuries & poisonings
859	3265	394929	10767	Complication due to other implant and internal device	injuries & poisonings
870.3	2919	400426	5616	Other open wound of head and face	injuries & poisonings
870	3837	400426	4698	Open wounds of head; neck; and trunk	injuries & poisonings
871	4219	400426	4316	Open wounds of extremities	injuries & poisonings
907	1315	407496	150	Injuries to the nervous system	injuries & poisonings
915	4289	403595	1077	Superficial injury without mention of infection	injuries & poisonings
916	1486	407475	0	Contusion	injuries & poisonings
939	2110	404817	2034	Atopic/contact dermatitis due to other or unspecified	dermatologic
946	548	404817	3596	Anaphylactic shock NOS	injuries & poisonings
960.2	16090	381797	11074	Allergy/adverse effect of penicillin	injuries & poisonings
960	18430	381797	8734	Poisoning by antibiotics	injuries & poisonings
961.1	874	381797	26290	Poisoning/allergy of sulfonamides	injuries & poisonings
961	1256	381797	25908	Poisoning by other anti-infectives	injuries & poisonings
965.1	1150	381797	26014	Opiates and related narcotics causing adverse effects in therapeutic use	injuries & poisonings
965	6910	381797	20254	Poisoning by analgesics, antipyretics, and antirheumatics	injuries & poisonings
966	509	381797	26655	Poisoning by anticonvulsants and anti-Parkinsonism drugs	injuries & poisonings
967	588	381797	26576	Adverse effects of sedatives or other central nervous system depressants and anesthetics	injuries & poisonings
969	1940	381797	25224	Poisoning by psychotropic agents	injuries & poisonings

979	695	381797	26469	Adverse drug events and drug allergies	injuries & poisonings
990	3130	403295	2536	Effects radiation NOS	injuries & poisonings
994.2	2811	406150	0	Sepsis	infectious diseases
994	2811	406150	0	Sepsis and SIRS	infectious diseases

Supplementary Figure 1 The Pearson correlation analysis among different tissues.

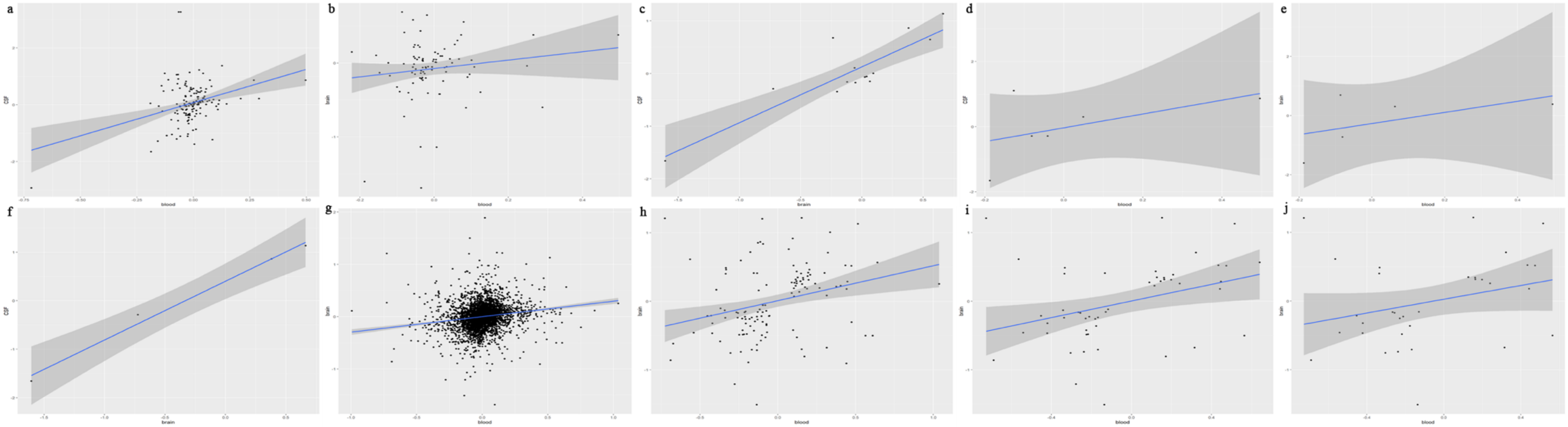

a: The correlation analysis for the MR effects between brain proteins and CSF proteins (No p-value threshold, Pearson correlation = 0.8745, p-value =4.28e-05, number of genes =14). **b**: The correlation analysis for the MR effects between brain proteins and CSF proteins when limited p-value threshold to 0.05(Pearson correlation = 0.9946, p-value =0.0053, number of genes =4). **c-d**: **c,**The MR effects between blood proteins and CSF proteins showed a robust positive correlation at no p-value threshold (Pearson correlation = 0.3311, p-value =4.19e-05, number of genes =147), **d**,while increasing the p-value threshold to p<0.05 led to no correlation (p-value =0.2835, number of genes =6); **e-f:** the MR effects between blood proteins and brain proteins showed no correlation both at no p-value threshold (**e**) and 0.05 threshold (**f**). **g-j:** At the transcriptional level, we found a robust positive correlation between the brain and blood MR effects (**g**, No p value threshold, Pearson correlation = 0.167, p-value <2.2e-16, number of genes =4283). Increasing the p-value threshold resulted in a still robust and higher correlation (**h**, p<0.05 threshold: Pearson correlation= 0.286, p-value =0.001, number of genes = 126; **i**, p<0.01 threshold: Pearson correlation= 0.349, p-value =0.012, number of genes = 50; **j,** p<0.005 threshold: Pearson correlation= 0.287, p-value =0.005, number of genes = 34) between brain and blood eQTL.

Supplementary Figure 2 The Protein-protein interaction network using proteins suggestive to be causal for PD from brain.

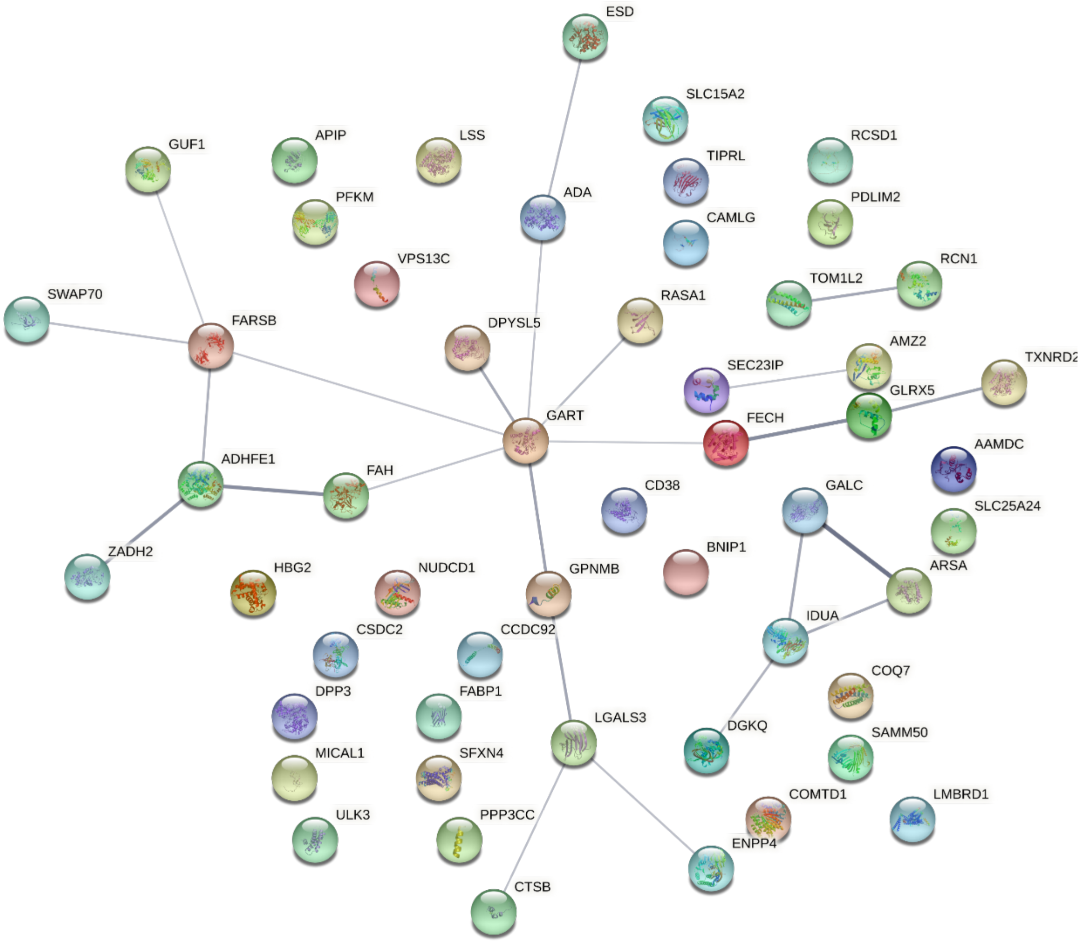

Note:
number of nodes:
53
number of edges:
24
average node degree:
0.906
avg. local clustering coefficient:
0.308
expected number of edges:
9
PPI enrichment p-value:
3.43e-05

Supplementary Figure 3 The Protein-protein interaction network using proteins suggestive to be causal for PD from CSF.

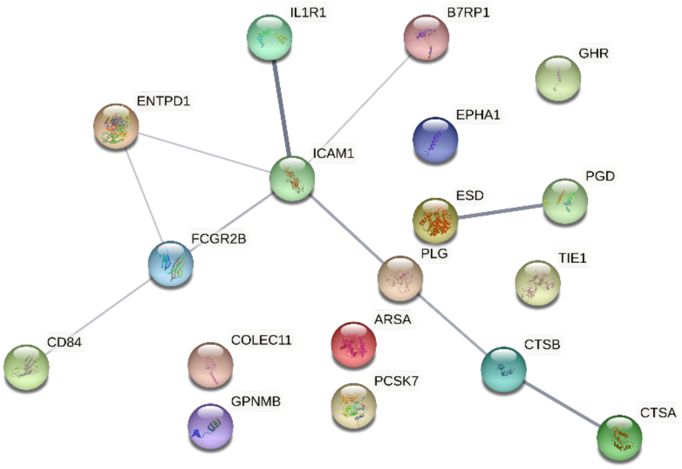

Note:
number of nodes:
18
number of edges:
10
average node degree:
1.11
avg. local clustering coefficient:
0.413
expected number of edges:
2
PPI enrichment p-value:
4.48e-05

Supplementary Figure 4 The Protein-protein interaction network using proteins suggestive to be causal for PD from blood.

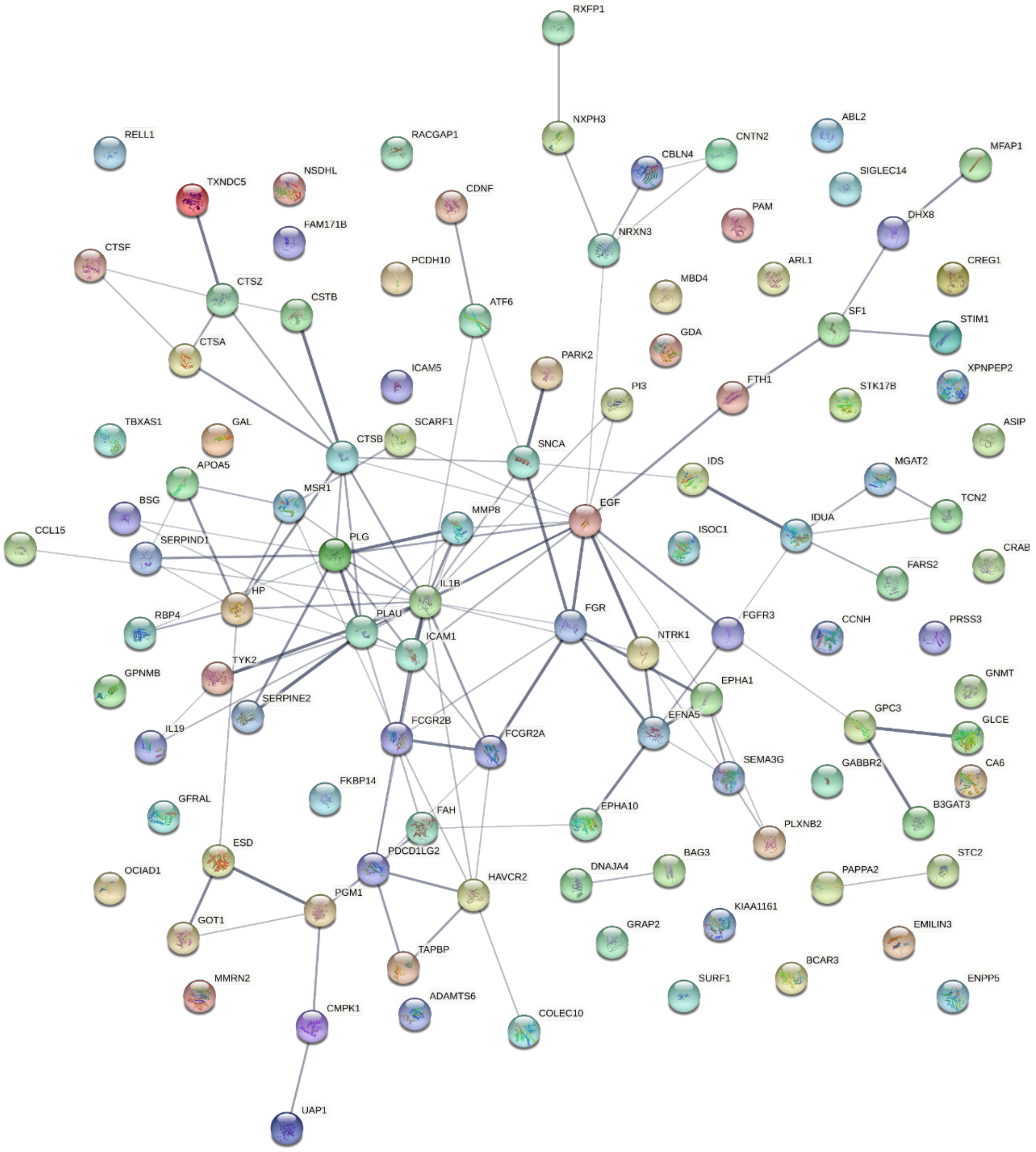

Note:
number of nodes:107
number of edges:127
average node degree:2.37
avg. local clustering coefficient:0.378
expected number of edges:57
PPI enrichment p-value:1.44e-15

Supplementary Figure 5 The Protein-protein interaction network using MR-identified proteins passed multiple correction.

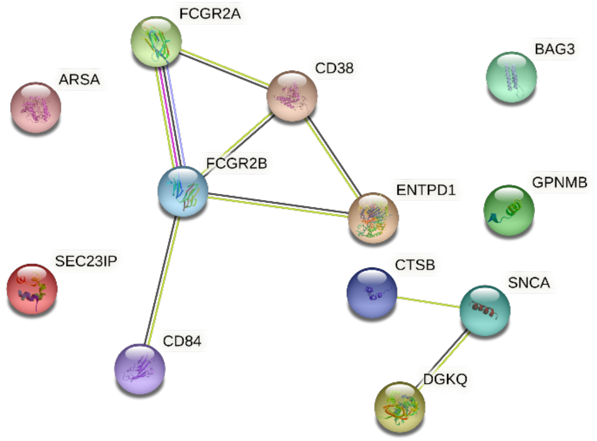

Note:
number of nodes:
12
number of edges:
8
average node degree:
1.33
avg. local clustering coefficient:
0.5
expected number of edges:
1
PPI enrichment p-value:
0.000108

Supplementary Figure 6: Cell type specific expression analysis.

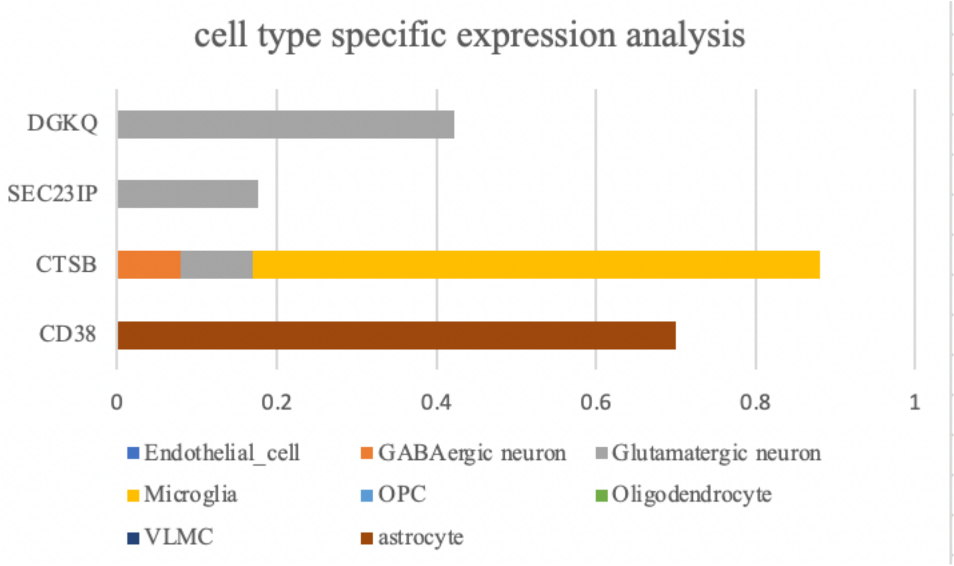

Supplementary Figure 7: Results of phenome-wide MR.

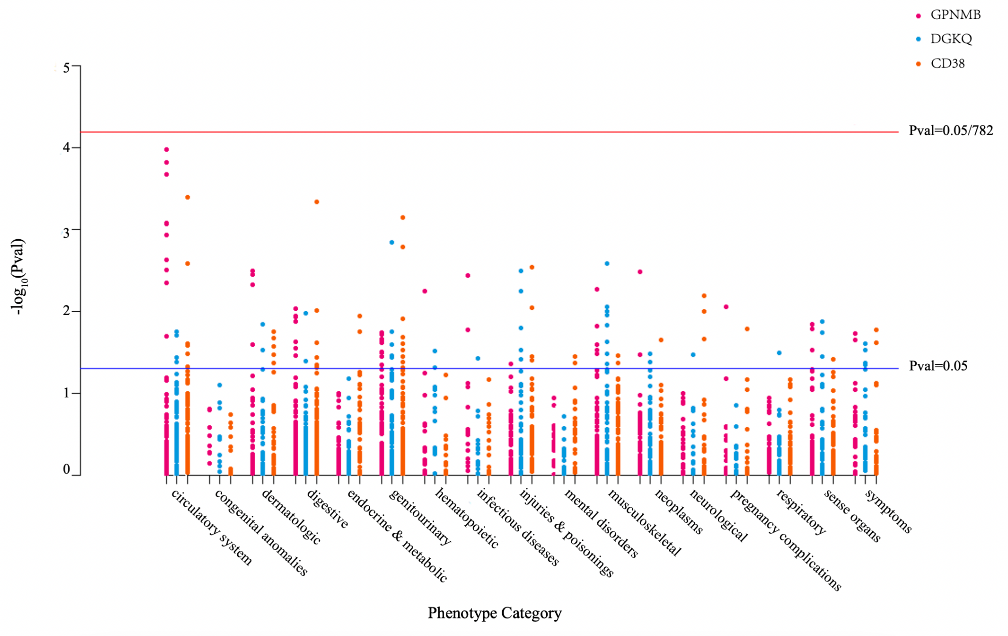

As our previous study, in order to assess the potential side effects of therapeutic targets, we utilized therapeutic target gene expression in the brain as the exposure factor and summary statistics of diseases in the UK Biobank cohort (n≤408 961) as the outcomes for conducting phenome-wide MR. Causal effects are considered statistically significant when $p<0.05/782$ (after applying Bonferroni correction).

Supplementary References:

1. Nalls MA, Blauwendraat C, Vallerga CL, Heilbron K, Bandres-Ciga S, Chang D, et al. Identification of novel risk loci, causal insights, and heritable risk for Parkinson's disease: a meta-analysis of genome-wide association studies. Lancet Neurol 2019; 18 (12): 1091-1102. doi: 10.1016/S1474-4422(19)30320-5.
2. Wingo AP, Liu Y, Gerasimov ES, Gockley J, Logsdon BA, Duong DM, et al. Integrating human brain proteomes with genome-wide association data implicates new proteins in Alzheimer's disease pathogenesis. Nat Genet 2021; 53 (2): 143-146. doi: 10.1038/s41588-020-00773-z.
3. Akbarian S, Liu C, Knowles JA, Vaccarino FM, Farnham PJ, Crawford GE, et al. The PsychENCODE project. Nat Neurosci 2015; 18 (12): 1707-1712. doi: 10.1038/nn.4156.
4. Yang C, Farias FHG, Ibanez L, Suhy A, Sadler B, Fernandez MV, et al. Genomic atlas of the proteome from brain, CSF and plasma prioritizes proteins implicated in neurological disorders. Nat Neurosci 2021; 24 (9): 1302-1312. doi: 10.1038/s41593-021-00886-6.
5. Sun BB, Maranville JC, Peters JE, Stacey D, Staley JR, Blackshaw J, et al. Genomic atlas of the human plasma proteome. Nature 2018; 558 (7708): 73-79. doi: 10.1038/s41586-018-0175-2.
6. Vosa U, Claringbould A, Westra HJ, Bonder MJ, Deelen P, Zeng B, et al. Large-scale cis- and trans-eQTL analyses identify thousands of genetic loci and polygenic scores that regulate blood gene expression. Nat Genet 2021; 53 (9): 1300-1310. doi: 10.1038/s41588-021-00913-z.
7. Lunati A, Lesage S, Brice A. The genetic landscape of Parkinson's disease. Rev Neurol (Paris) 2018; 174 (9): 628-643. doi: 10.1016/j.neurol.2018.08.004.
8. Armstrong MJ, Okun MS. Diagnosis and Treatment of Parkinson Disease: A Review. JAMA 2020; 323 (6): 548-560. doi: 10.1001/jama.2019.22360.
9. Wishart DS, Feunang YD, Guo AC, Lo EJ, Marcu A, Grant JR, et al. DrugBank 5.0: a major update to the DrugBank database for 2018. Nucleic Acids Res 2018; 46 (D1): D1074-D1082. doi: 10.1093/nar/gkx1037.
10. Wang D, Liu S, Warrell J, Won H, Shi X, Navarro FCP, et al. Comprehensive functional genomic resource and integrative model for the human brain. Science 2018; 362 (6420): doi: 10.1126/science.aat8464.
11. Storm CS, Kia DA, Almramhi MM, Bandres-Ciga S, Finan C, International Parkinson's Disease Genomics C, et al. Finding genetically-supported drug targets for Parkinson's disease using Mendelian randomization of the druggable genome. Nat Commun 2021; 12 (1): 7342. doi: 10.1038/s41467-021-26280-1.
12. Nalls MA, Pankratz N, Lill CM, Do CB, Hernandez DG, Saad M, et al. Large-scale meta-analysis of genome-wide association data identifies six new risk loci for Parkinson's disease. Nat Genet 2014; 46 (9): 989-993. doi: 10.1038/ng.3043.
13. Hillary RF, McCartney DL, Harris SE, Stevenson AJ, Seeboth A, Zhang Q, et al. Genome and epigenome wide studies of neurological protein biomarkers in the Lothian Birth Cohort 1936. Nat Commun 2019; 10 (1): 3160. doi: 10.1038/s41467-019-11177-x.

14. Png G, Barysenka A, Repetto L, Navarro P, Shen X, Pietzner M, et al. Mapping the serum proteome to neurological diseases using whole genome sequencing. Nat Commun 2021; 12 (1): 7042. doi: 10.1038/s41467-021-27387-1.

15. Pankratz N, Dumitriu A, Hetrick KN, Sun M, Latourelle JC, Wilk JB, et al. Copy number variation in familial Parkinson disease. PLoS One 2011; 6 (8): e20988. doi: 10.1371/journal.pone.0020988.

16. Kia DA, Zhang D, Guelfi S, Manzoni C, Hubbard L, Reynolds RH, et al. Identification of Candidate Parkinson Disease Genes by Integrating Genome-Wide Association Study, Expression, and Epigenetic Data Sets. JAMA Neurol 2021; 78 (4): 464-472. doi: 10.1001/jamaneurol.2020.5257.

17. Chang D, Nalls MA, Hallgrímsdóttir IB, Hunkapiller J, van der Brug M, Cai F, et al. A meta-analysis of genome-wide association studies identifies 17 new Parkinson's disease risk loci. Nat Genet 2017; 49 (10): 1511-1516. doi: 10.1038/ng.3955.

18. Baird DA, Liu JZ, Zheng J, Sieberts SK, Perumal T, Elsworth B, et al. Identifying drug targets for neurological and psychiatric disease via genetics and the brain transcriptome. PLoS Genet 2021; 17 (1): e1009224. doi: 10.1371/journal.pgen.1009224.

19. Qi T, Wu Y, Zeng J, Zhang F, Xue A, Jiang L, et al. Identifying gene targets for brain-related traits using transcriptomic and methylomic data from blood. Nat Commun 2018; 9 (1): 2282. doi: 10.1038/s41467-018-04558-1.

20. Bandres-Ciga S, Ahmed S, Sabir MS, Blauwendraat C, Adarmes-Gomez AD, Bernal-Bernal I, et al. The Genetic Architecture of Parkinson Disease in Spain: Characterizing Population-Specific Risk, Differential Haplotype Structures, and Providing Etiologic Insight. Mov Disord 2019; 34 (12): 1851-1863. doi: 10.1002/mds.27864.

21. Zhu Z, Zhang F, Hu H, Bakshi A, Robinson MR, Powell JE, et al. Integration of summary data from GWAS and eQTL studies predicts complex trait gene targets. Nat Genet 2016; 48 (5): 481-487. doi: 10.1038/ng.3538.
